# NMR-Chemical-Shift-Driven Protocol Reveals the Cofactor-Bound, Complete Structure of Dynamic Intermediates of the Catalytic Cycle of Oncogenic KRAS G12C Protein and the Significance of the Mg^2+^ Ion

**DOI:** 10.3390/ijms241512101

**Published:** 2023-07-28

**Authors:** Márton Gadanecz, Zsolt Fazekas, Gyula Pálfy, Dóra Karancsiné Menyhárd, András Perczel

**Affiliations:** 1 Laboratory of Structural Chemistry and Biology, Institute of Chemistry, Eötvös Loránd University, Pázmány Péter stny. 1/A, H-1117 Budapest, Hungary; marton.gadanecz@ttk.elte.hu (M.G.); dora.k.menyhard@ttk.elte.hu (D.K.M.); 2 Hevesy György PhD School of Chemistry, Eötvös Loránd University, Pázmány Péter stny. 1/A, H-1117 Budapest, Hungary; 3 ELKH-ELTE Protein Modeling Research Group, Eötvös Loránd Research Network (ELKH), Pázmány Péter stny. 1/A, H-1117 Budapest, Hungary; 4 Department of Biology, Institute of Biochemistry, ETH Zürich, 8093 Zürich, Switzerland

**Keywords:** protein structure determination, protein dynamics, NMR spectroscopy, molecular dynamics simulation, KRAS, ligand binding

## Abstract

In this work, catalytically significant states of the oncogenic G12C variant of KRAS, those of Mg^2+^-free and Mg^2+^-bound GDP-loaded forms, have been determined using CS-Rosetta software and NMR-data-driven molecular dynamics simulations. There are several Mg^2+^-bound G12C KRAS/GDP structures deposited in the Protein Data Bank (PDB), so this system was used as a reference, while the structure of the Mg^2+^-free but GDP-bound state of the RAS cycle has not been determined previously. Due to the high flexibility of the Switch-I and Switch-II regions, which also happen to be the catalytically most significant segments, only chemical shift information could be collected for the most important regions of both systems. CS-Rosetta was used to derive an “NMR ensemble” based on the measured chemical shifts, which, however, did not contain the nonprotein components of the complex. We developed a torsional restraint set for backbone torsions based on the CS-Rosetta ensembles for MD simulations, overriding the force-field-based parametrization in the presence of the reinserted cofactors. This protocol (csdMD) resulted in complete models for both systems that also retained the structural features and heterogeneity defined by the measured chemical shifts and allowed a detailed comparison of the Mg^2+^-bound and Mg^2+^-free states of G12C KRAS/GDP.

## 1. Introduction

Protein structure determination plays a crucial role in understanding biological functions, and it also empowers the exploration of novel strategies to regulate pathological processes. The primary approaches for comprehending protein function involve gathering sufficient information through experimental techniques or various forms of computational modelling. The prediction of the tertiary and quaternary structure of proteins from only sequential information was a major challenge until the introduction of the artificial-intelligence-based methods, AlphaFold [1] and RoseTTAfold [2]. However, as M. L. Hekkelman et al. emphasizes [3], these methods are unprecedently reliable for domain structures, but the predictions for flexible parts are less accurate. Furthermore, the predicted structures do not include small molecules, ligands, and cofactors, which are typically associated with proteins. When considering experimental techniques providing atomic resolution, three methods must be mentioned: X-ray crystallography, cryoelectron microscopy (cryo-EM), and nuclear magnetic resonance (NMR) spectroscopy. NMR spectroscopy is the only one of these methods which is capable of studying both the structure and inherent dynamic behavior of macromolecules in solution [4]. The primary observables of NMR spectroscopy are the chemical shifts, which are highly reproducible and sensitive parameters. They depend on the local magnetic field and provide insight into covalent structural features, noncovalent structure, solvent interactions, ionization constants, ring orientations, hydrogen bond interactions, and other structure-based features [5]. It is possible to obtain long-range distance information from paramagnetic relaxation (PRE) experiments, torsion angles from J-couplings, and relative orientations of interatomic vectors from residual dipolar coupling (RDC) measurements [6,7,8]. The most commonly used NMR approach to determine structural information in the form of interatomic distances is to perform nuclear Overhauser effect (NOE) measurements. However, the assignment of the NOE cross-peaks can be difficult because of spectral overlap and resonance frequency degeneracy. If a sufficient number of NOE cross-peaks can be observed, software packages such as CYANA [9], UNIO [10], ARIA [11], and XPLOR-NIH [12] are able to automate the structure solution process, convert the NOE information into ambiguous distance restraints, and calculate the protein structure. To further automate this process, the ARTINA [13,14] workflow has been developed, using deep-learning neural networks. ARTINA is able to perform the peak picking, resonance assignment using FLYA [15], chemical-shift-based torsional restraints generation by TALOS-N [16], NOE cross-peak assignment, ambiguous distance restraints generation, and structure calculation by CYANA’s simulated annealing protocol [9] without any user intervention, using only the sequence, a collection of high-quality processed spectra and potentially other different types of inputs (partial or full assignments, predicted structures, lower/upper limit restraints, etc.). Unfortunately, NOE measurements of the flexible regions of proteins, where, for example, conformational exchange occurs on a micro- to millisecond time scale of motions, can be problematic due to line broadening, and it can be difficult to obtain structural information. In these regions of a protein, distance-restraints-based structure determination software can only rely on its force field or structure prediction approach.

The NMR chemical shifts of protein atoms depend strongly on the local backbone geometry, carrying information about the secondary structure, sidechain conformations, and dynamics. This phenomenon facilitates the determination of the peptide backbone (ϕ, ψ and ω) and sidechain (χ_1_) dihedral (or torsion) angles directly from the backbone resonances, which are usually accessible at an early stage of NMR studies. Thus, in theory, chemical shifts combined with a library of fragments of known 3D structures can be sufficient to determine the structure of a protein [8,17,18,19]. Chemical-Shift-Rosetta (CS-Rosetta) [20] is a method dedicated to this task; it uses a high-resolution protein structure database from PISCES [21] (a nonhomologous subset of PDB) supplemented with secondary structure assignments derived from DSSP [22] and chemical shifts predicted by SPARTA [23]. As a first step of the structure determination, a fragment library is built using chemical shifts similarity, secondary structure likeliness, and sequential homology. The fragment library of three- and nine-residue-long fragments contains many possible backbone conformations to drive the structure building process. Fragments are selected, assembled, and refined using Rosetta’s Metropolis Monte Carlo method and energy functions [18,24]. In conformationally flexible regions, where NMR-based information is not accessible, CS-Rosetta reverts to the standard Rosetta approach of using homology-based information to predict the possible backbone conformations [8].

Nearly 80% of all PDB entries (queried in September of 2018) contain some kind of small molecule bound to proteins or nucleic acids [25], which clearly conveys the importance of the nonprotein components of protein models. Despite this, most of the above-mentioned software tools are unable to incorporate any nonprotein components into their model-building protocols. Here, we present a workflow that allows the incorporation of chemical shift data via CS-Rosetta to generate a primary structural ensemble, which is further refined using molecular dynamics (MD) simulations, but which retains the fold (and backbone) information present in the CS-Rosetta ensemble—replacing the backbone restraints of the force field with those derived from the Rosetta approach.

**Figure 1:**
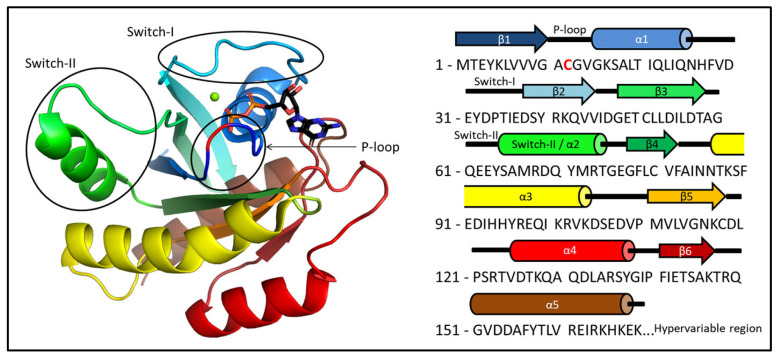
The 3D structure of KRAS-G12C with both GDP and Mg^2+^ ion with the primary sequence of the oncogenic mutant protein. The P-loop (residues 10–17), Switch-I (residues 28–40), and Switch-II (residues 60–76) regions are circled. The position of the mutation within the P-loop (G12C) is colored red.

We chose to demonstrate the structure-solving power of this chemical-shift-based protocol in the case of the resting state and Mg^2+^-free form of the G12C variant of KRAS, both a physiologically and therapeutically significant protein whose function is carried out by the rearrangement of two highly flexible regions called Switch-I and Switch-II. Due to the conformational heterogeneity of the switches, the experimental study of KRAS is quite challenging, with crystal structures often based on a weak electron density in these regions (or omitting them) and NMR datasets that are also most uncertain or broadened in these catalytically critical segments. About 20–30% of all human oncogenic diseases are initiated by mutations in one of the three RAS genes: KRAS, HRAS, and NRAS [26]. RAS proteins are membrane-bound small GTPases that play a key role in several signal transduction pathways as molecular switches, alternating between the GDP-bound OFF and the GTP-bound ON states, regulating cell growth, proliferation, and survival [27]. RAS proteins bind Mg^2+^ as a cofactor in both of the active and resting forms, which is released—together with the nucleotide—during the GDP/GTP nucleotide exchange step of the catalytic cycle [28]. Mutations in critical positions, mainly in the so-called P-loop (Figure 1) (the KRAS-G12C mutant is one of the most studied examples), lead to a shift towards the active form and consequently to the excessive activation of the signal transduction pathways by interfering with the binding of the assisting protein, GAP, which enhances the GTP→GDP hydrolysis step of the catalytic cycle [29]. This step results in the “switching OFF” of the growth signal and the return to the resting GDP-bound state. Therapeutic targeting of oncogenic RAS proteins is made extremely difficult by the apparent lack of structural differences between either the GDP- or the GTP-bound forms of mutant variants and the wild-type protein [30]. However, the dynamics and internal interaction networks of the oncogenic variants were shown to differ from those of the wild-type one [31] indicating that intermediates between the resting and active states may carry mutation-state-dependent structural differences.

The GDP/GTP exchange step is catalyzed by another helper protein, GEF (guanosine exchange factor), which—upon complex formation with RASs—invades the nucleotide-binding site and forces the GDP release. Following the dissociation of the GEF, the vacated nucleotide-binding site is loaded with GTP, which is available in plasma at an order of magnitude greater concentration than GDP: the intracellular concentration of GTP ranges from 100 to 200 μM, whereas the concentration of GDP ranges from 10 to 20 μM [32]. The assistance of the GAP and GEF is made necessary by the very poor intrinsic hydrolytic and exchange capacity of RAS proteins but also provides a means of further controlling the activation process. We have previously shown that the Mg^2+^-free state of KRAS is best characterized as an intermediate between the GDP-bound resting state and the completely nucleotide-free (and Mg^2+^-free) KRAS:GEF complex state [31]. It has also been shown that the removal of the Mg^2+^ ion enhances intrinsic—assistance-free—GDP release [33], therefore the Mg^2+^-free state can safely be assumed to be a transiently appearing intermediate of the nucleotide release step of the catalytic cycle. Since both the nucleotide release capacity and its dependence on the presence/absence of the Mg^2+^ ion are mutation-state sensitive, determining the structure of the Mg^2+^-free forms of oncogenic mutants might expose structural differences, which would allow a mutation-specific targeting of these transient states.

## 2. Results

### 2.1. Ab Initio Structure Models of the Mg^2+^-Bound KRAS-G12C/GDP by Chemical-Shift-Rosetta 

**Figure 2:**
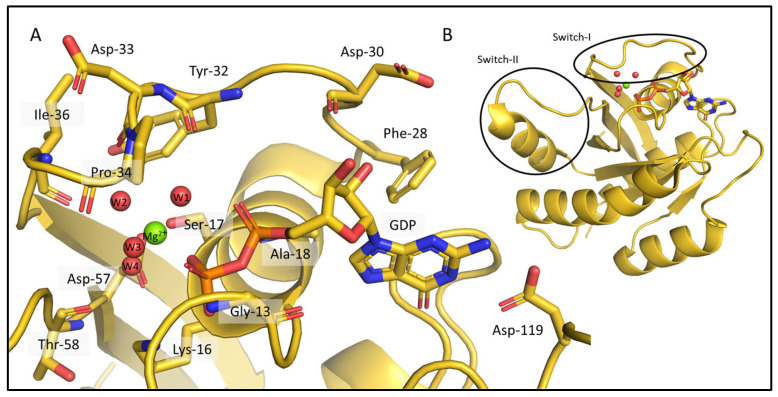
Interactions between the KRAS apo-protein and its nonprotein components, a GDP ligand and a Mg^2+^ cofactor in the crystal structure 4OBE (color: gold). (**A**) The critical amino acids are shown around the GDP and the Mg^2+^ ion. The binding pocket of KRAS and all the residues coordinating the nucleotide and the ion are represented as sticks, Mg^2+^ is shown as a green sphere, the waters in the coordination sphere of the ion are red spheres and all the mentioned parts of the structure are labelled. The list of the interactions between the molecules and the ion is given in Table S1. (**B**) The full 3D structure of KRas-G12C/GDP-Mg^2+^ (PDB ID: 4OBE).

Despite the large number of crystal structures in the PDB representing different catalytic states and variants of KRAS, NMR-derived structures exist. In fact, the only ab initio determined NMR structure of the few wild-type protein (PDB ID: 7KYZ) does not contain either the GDP, or the Mg^2+^ ion, despite their presence in the sample used for the NMR data acquisition. According to the KRAS crystal structures (e.g., PDB ID: 4OBE, wild-type KRAS, shown in Figure 2) numerous interactions are formed between the apo-protein, the GDP ligand, and the Mg^2+^ cofactor (Figure 2A). The sidechains of Lys-16, Phe-28, and Asp-119, and the backbone of Gly-13, Ala-18, and Asp-30 typically coordinate the nucleotide. The Mg^2+^ ion is bound directly by the sidechain of Ser-17 and the β-phosphate of GDP, and by water-mediated interactions that ultimately connect it to the main chain of Tyr-32, Asp-33, Pro-34, Ile-36, Asp-57, and Thr-58 (Table S1). Since the latter are part of either the Switch-I (spanning residues 28–40) or the Switch-II (residues 60–76) regions, the most flexible segments of the protein, the listed interactions are not necessarily always all present in solution.

The challenges that we aim to address in this work are nicely demonstrated by the NMR-based ensemble deposited in the PDB (7KYZ), which was derived using the PONDEROSA and CYANA programs for automated peak picking, structure solution, and refinement [34] (using 2140 distance restraints, 138 hydrogen bond restraints, and 238 dihedral angle restraints), neither of which supports the inclusion of nonprotein components into the built models. Switch-I residues such as Phe-28, Asp-30, Tyr-32, Asp-33, and Pro-34 were found to be quite flexible as expected. However, the fact that the model does not contain the nucleotide and the Mg^2+^ ion may actually account for some of the structural heterogeneity of this segment. The sidechains of the Lys-16, Phe-28, and Asp-119 residues are in conformations incompatible with the binding of the GDP ligand (which is coordinated to the nucleotide-binding site in a rather well-conserved mode). In addition, in some of the models, the sidechain of Tyr-32 also protrudes into the binding space of the nucleotide. In only about half of the models are the sidechains of Ser-16 and Asp-57 in a suitable conformation to coordinate the Mg^2+^ ion; in the rest, they point in another direction (Figure S1). These observations highlight the importance of developing methods that allow cofactors, ligands, or substrates to be included in NMR structural ensembles, even if additional information regarding their position cannot be obtained, simply because their presence limits the conformational pool from which the protein can be sampled. Since there is no significant difference in the structure of the wild-type and oncogenic mutant KRAS proteins, a similar problem will arise when determining the structure of mutants such as G12C by NMR.

**Figure 3:**
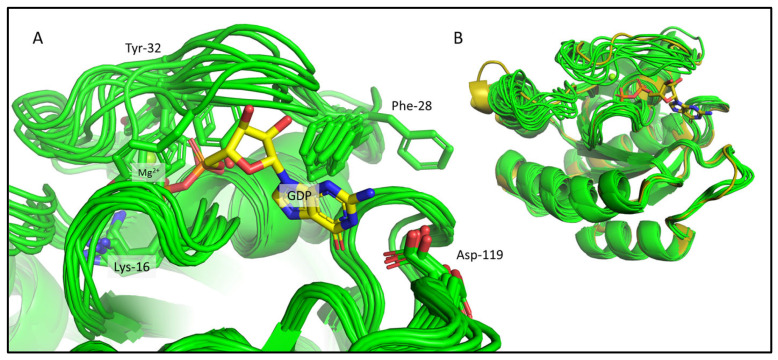
The CS-Rosetta ensemble shown for KRas-G12C. (**A**) The binding pocket of the best 10 CS-Rosetta models (light green) with some of the important residues (Lys-16, Phe-28, Tyr-32, Asp-119) whose sidechains are shown as sticks and are labelled. The GDP and the Mg^2+^ are also shown from the 4OBE (gold sticks and green sphere), but they are not present in the CS-Rosetta ensemble. (**B**) Structural alignment of the best 10 CS-Rosetta models (light green) with the 4OBE reference crystal structure (gold).

We carried out ab initio model building of the oncogenic mutant KRAS-G12C (Mg^2+^- and GDP-bound forms) using CS-Rosetta, a method relying on chemical shift data only. As CS-Rosetta does not support the inclusion of nonprotein components either, the ensemble it provides carries problems that are similar to those of the 7KYZ ensemble, but to a lesser extent. In well-scored 3D models, both Lys-16 and Asp-119 residues are usually in the right conformation to form H-bonds with GDP, with Phe-28 in the correct position to form π-π interactions in most cases. Ser-17 and Asp-57 are in positions that allow for the coordination of the Mg^2+^ ion. However, in about half of the best 10 3D models (Figure 3A), Tyr-32 is placed in a position where it would clash with the GDP instead of pointing inward to the pocket outlined by the Switch-I loop. In the crystal structures of the GDP/Mg^2+^-bound resting state, the Tyr-32 sidechain participates in an H-bond with Tyr-40, forming a Tyr-gate over Switch-I (the Tyr-32 OH–Tyr-40 OH distances in the 4OBE models are 3.2 and 2.9 Å, in chain A and B, respectively). This is a catalytically significant interaction that obstructs the access to the recognition and binding site of the downstream effectors (e.g., RAF) that KRAS activates, when it is in the GTP-bound activated state [35]. At this point, it is important to emphasize that CS-Rosetta models are built using chemical-shift-based backbone dihedral angles, while the sidechains conformations are derived from a rotamer library subsequently minimized. On the other hand, the backbone carbonyl oxygen atom of Tyr-32 should coordinate one of the waters in the coordination sphere of Mg^2+^ (“W1” on Figure 2)—and the flipping of its sidechain means that its backbone atoms are also unavailable for this interaction. Similarly, the entire Switch-I region of the CS-Rosetta models also needs to be refined, as it is too close to the nucleotide ligand in some of the 3D structures.

### 2.2. Chemical-Shift-Driven MD Refinement—Reinsertion of the Nonprotein Components in the Case of the KRAS-G12C/GDP-Mg^2+^ System

To address the problems described above, we introduced an additional refinement step to the CS-Rosetta NMR structure determination process, where we successfully reinserted the GDP and Mg^2+^ into the model, using chemical-shift-driven molecular dynamics simulations (csdMD). During the CS-Rosetta ab initio structure determination, the program uses a fragment library constructed from sequence and chemical shifts (N, HN, C, Cα, Cβ, and Hα). This library contains numerous potential ϕ (phi angle: C_i−1_–N_i_–Cα_i_–C_i_), ψ (psi angle: N_i_–Cα_i_–C_i_–N_i+1_) and ω (omega angle: Cα_i_–C_i_–N_i+1_–Cα_i+1_) backbone dihedral angles for each residue. The robustness of the CS-Rosetta structure determination method stems from the huge number of calculated models, but only the best few thousandths of the models are considered as results. In Figure S2C, we can see that the number of structures calculated in CS-Rosetta (10,000) was sufficient for both systems, since the models converged, that is, the best models were also structurally similar (as reflected in their low RMSD values). The obtained CS-Rosetta ensemble was used to define energy terms concerning ϕ and ψ dihedral angles for the csdMD simulations, since these are the most direct information from the NMR measurements. The ω dihedral angles were excluded because the planar peptide bond makes them nearly constant. First, we defined the potential energy function (PEF) from the probability of occurrence of the dihedral angles ϕ and ψ. These data were weighted with the CS-Rosetta scores, so that the contribution of less likely structures (based on the CS-Rosetta score) was smaller. The negative derivative of the PEF is the force acting on these angles during the csdMD refinement, meaning that these forces pull the backbone to conformations similar to those found in the best CS-Rosetta models. The force was scaled to the same order of magnitude as the original GROMACS energy terms. In this way, the backbone dihedral angles sampled by the CS-Rosetta ensemble and their weighted distribution, reflecting the measured chemical shifts, replaced the force-field-derived torsional angles and constraints along the entire length of the protein backbone in the MD refinement step. The detailed description of this process can be found in the methods, and the Python3 scripts to perform it are publicly available on GitHub (https://github.com/fazekaszs/ensemble_to_gromacs, accessed on 24 July 2023).

**Figure 4:**
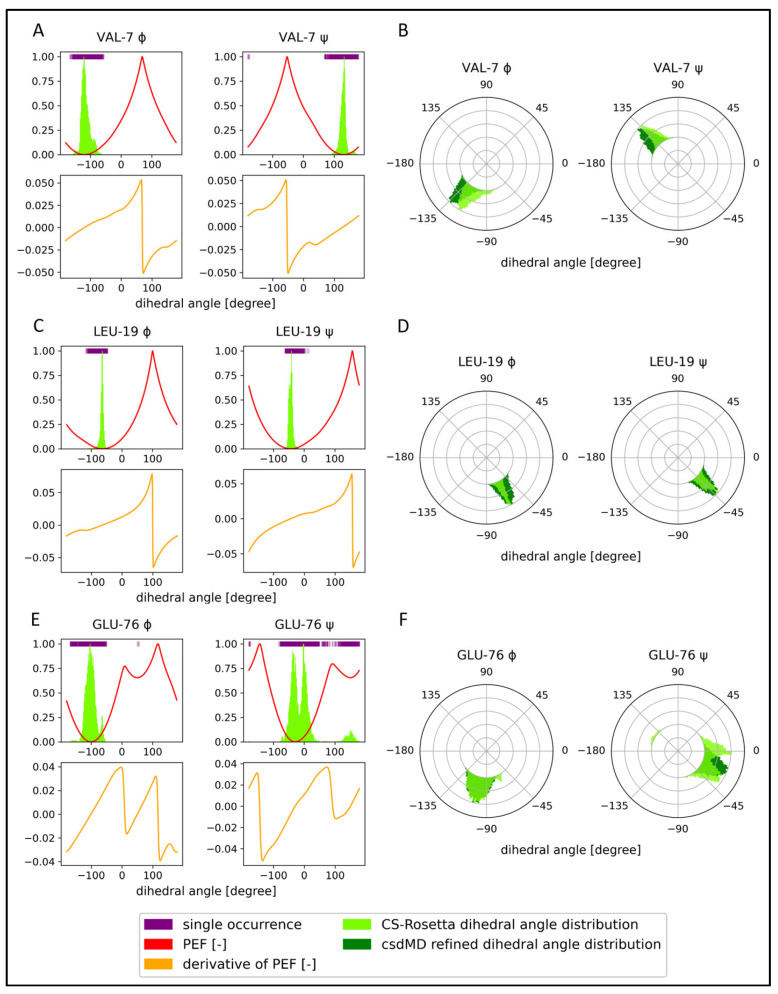
Comparison of the unrefined and refined ensembles of KRAS-G12C/GDP-Mg^2+^ angle distributions, supplemented with the PEF figures and derivatives. Three residues from a β-sheet (Val-7), an α-helix (Leu-19), and a loop (Glu-76) are shown. The top figures (**A**,**C**,**E**) show the dihedral angle distribution of the CS-Rosetta ensemble as a light green histogram, single dihedral angle occurrences as purple lines at the top of the figures, and the potential energy function (PEF) calculated from the histograms with a red line. The lower figures (**A**,**C**,**E**) with orange lines are the first derivatives of the PEF. The (**B**,**D**,**F**) diagrams are the ϕ and ψ dihedral angle distributions on a polar coordinate system, where the CS-Rosetta data before refinement are light green and the csdMD refined data are dark green.

Here, a 1000 ns long csdMD simulation was performed on the NMR data obtained for the KRAS-G12C/GDP-Mg^2+^ system, starting from the appropriately prepared best CS-Rosetta model containing the manually reinserted nucleotide and Mg^2+^ ion. The trajectory was clustered and analyzed in the 500–1000 ns time range. The distributions of the CS-Rosetta scores are shown in Figure S2A,B. In Figure 4A,C,E, some examples for the potential energy curves derived from the dihedral angle distributions can be seen. Similar diagrams for all the residues can be found in the Supplementary Materials.

Examples of the dihedral angle distribution from the CS-Rosetta ensemble before and after the csdMD refinement are shown in Figure 4B,D,F, on a polar coordinate system. All the dihedral angle figures are shown in the Supplementary Materials. We can clearly see the difference between the flexibility of different regions. The three examples shown here are those of Val-7 in the β1-strand, Leu-19, part of the α1-helix, and Glu-76 from a loop. Comparing the ensembles before and after csdMD refinement, some general differences can be observed. The unrefined ensembles often show multimodal distributions. The reason for this could be that the dihedral angles of CS-Rosetta are derived from a fragment library of crystal structures and that the molecules in the crystalline phase have a lower mobility than in the solution. So intermediate orientations or arrangements of flexible segments are scarcely sampled by CS-Rosetta, reflecting only the well-distinguished most stable conformations in the form of separate distribution peaks (Figure 4F).

It is an interesting phenomenon that in some regions the unrefined models show a greater structural heterogeneity compared to the csdMD refined data (Supplementary Materials: angle figures). In the first set of models, in the absence of the nucleotide and the ion, many conformations seem to be accessible for the Switch-I region, which would not be the case in the presence of the GDP and Mg^2+^. Amino acids in this loop, next to the nucleotide and the ion-binding site, such as Glu-31–Tyr-32–Asp-33–Pro-34–Thr-35–Ile-36–Glu-37, show bi- and multimodal dihedral angle distributions in the unrefined ensemble. In the refined models, only one mode occurs at these sequence positions, because of the interactions formed with the nonprotein components.

**Figure 5:**
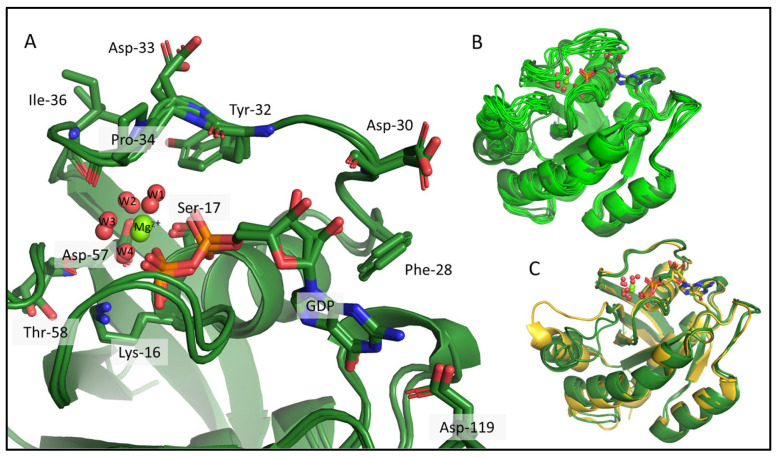
KRAS-G12C/GDP-Mg^2+^ csdMD refined cluster centers from 500 to 1000 ns are shown, which represent 98% of the snapshots. (**A**) Interactions between KRAS-G12C and its nonprotein moieties, the GDP, and the Mg^2+^ ion, where sidechains of some important amino acids are shown as sticks and are labelled. Mg^2+^ is shown as a green sphere, and the 4 water molecules in its coordination sphere are red spheres. (**B**) Aligned models before (light green) and after refinement (dark green). (**C**) Superimposed 3D models of our refined cluster centers (dark green) and the 4OBE reference crystal structure (gold).

During the csdMD refinement, the inserted nucleotide and Mg^2+^ ion remained stably bound in a manner similar to that seen in the crystal structures, despite the fact that no constraints were applied to restrict their movement: the RMSD calculated for the Mg^2+^ ion (after fitting the backbone of the non-Switch regions of the protein, using the 500–1000 ns segment of the trajectory) with respect to its position on the crystal structure (4OBE) was 0.87 ± 0.20 Å and 0.60 ± 0.19 Å for the centroid of the GDP nucleotide. Looking at the cluster centers in Figure 5A, we can see that all the interactions between KRAS and the nonprotein components previously described (Table S1) in the crystal structures are present in the refined models. In Figure 5B, we can compare the ensembles before and after the csdMD refinement. The csdMD refined models show slightly less mobility in the Switch-I and Switch-II regions, because the nucleotide and the ion that are now present in the model interact with residues in these regions and fix them. The only significant difference is the tilt angle of Switch-II, which also varies between the crystal structures. We also ran the MD simulation for refinement with the unmodified AMBER-ff99SBildnp* force field, which resulted in a more flexible system, meaning that the proposed CS-Rosetta-based torsion angle energy terms during the csdMD refinement effectively restricted the conformational space into the region that was compatible with the measured chemical shifts (the RMSF and RMSD data are shown in Figures S4 and S5). There were some differences in the flexibility of some specific regions, e.g., the first half of Switch-I (Figure S4E,G). This part of Switch-I is the closest to the GDP ligand and becomes more ordered in the csdMD refined models. This region was quite similar in different crystal structures, supporting our results.

At this point, we claim to have determined the structure of KRAS-G12C/GDP-Mg^2+^ (a system for which the crystal structure is also available) using our new method combining CS-Rosetta and GROMACS molecular dynamics simulations—two free and open-source software suites—directly from NMR chemical shifts.

### 2.3. Comparison of the CS-Rosetta Ensembles of KRAS-G12C/GDP-Mg^2+^ and the Mg^2+^-Free KRAS-G12C/GDP System

**Figure 6:**
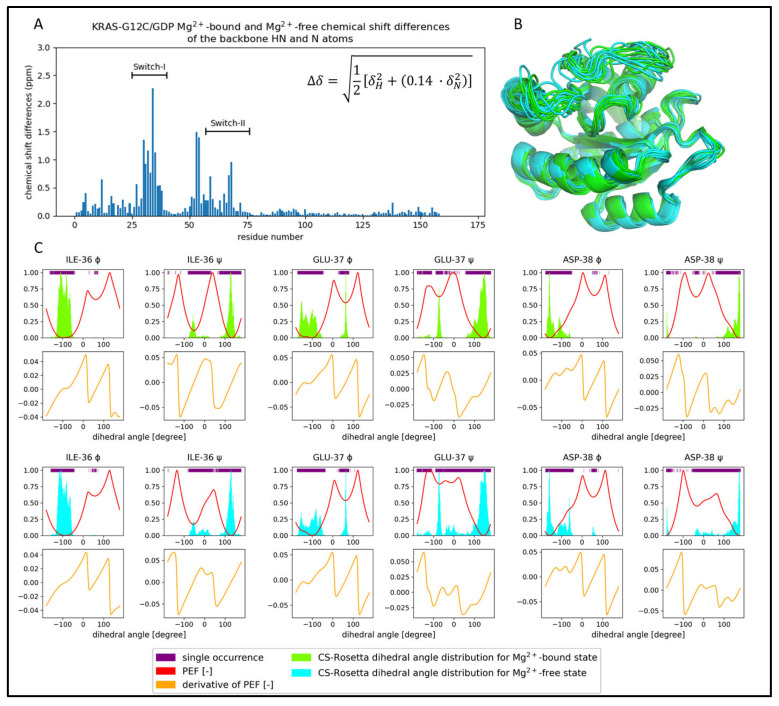
Comparison of the Mg^2+^-bound (light green) and Mg^2+^-free (cyan) KRAS-G12C/GDP of the CS-Rosetta ensembles. (**A**) Chemical shift differences of the backbone HN and N atoms scaled by the equation shown in the bar diagram. (**B**) The best 10 CS-Rosetta models are presented. It is important to note that both the GDP and Mg^2+^ ion are missing in these models. (**C**) Dihedral angle distributions and PEF figures are shown for some of the residues of different backbone flexibility.

Using the same methodology, we also determined the CS-Rosetta NMR-based ensemble of the Mg^2+^-free form of the KRAS-G12C/GDP protein. To do this, we completed our previously published NMR assignment of the Mg^2+^-free GDP-bound KRAS-G12C [36] with new data derived from further NMR measurements (4D NOESY and 4D TOCSY measurements) [37]. Figure 6A (and Figure S3) shows that there are significant differences between the measured chemical shifts of the two proteins in the Switch-I and Switch-II regions. The amino acids of Switch-I that differ the most (the largest difference is at position 37), are part of the ligand- and cofactor-binding site. We can also see large differences at the N-terminus of Switch-II, (specifically positions 58 and 59), where the Asp-57 and Thr-58 are located, which play a part in the coordination of the Mg^2+^ ion (Table S1, Figure 2A). Observing the largest chemical shift differences in the case of the residues located close to the Mg^2+^-ion-binding site is understandable: its missing charge clearly explains the large difference between the chemical environments these residues experience. However, a chemical shift change was found to be small for Ser-17, which is in direct contact with Mg^2+^ in the resting state structure and moderate in the case of Asp-33 and Ile-36, which anchor waters that form the coordination sphere of the ion. On the other hand, several residues that are not in contact with the ion in the resting state experienced a chemical shift change upon the loss of Mg^2+^. This indicates that the removal of the Mg^2+^ affects a wider region of the protein, beyond the immediate environment of the ion.

The CS-Rosetta ensembles of the two systems are similar (Figure 6B), but if we examine the dihedral angle distributions (Figure 6C and Supplementary Materials: angle figures), we can notice some characteristic deviations close to the ion-binding site. The largest deviations are found at positions Ile-36, Glu-37, and Asp-38 in Switch-I, where we see a decidedly greater structural heterogeneity than in the case of the Mg^2+^-bound form. As it can be seen in Figure 6C, this is coupled with the fact that the ψ dihedral angles at these positions show wider distributions in the Mg^2+^-free form. The difference of heterogeneity between the two CS-Rosetta ensembles stays hidden by only looking at the very best models, but it becomes visible if we examine a larger fraction (Figure S4A,B, where the RMSF values are shown).

### 2.4. Comparison of the csdMD Refined Ensembles of KRAS-G12C/GDP-Mg^2+^ and the Mg^2+^-Free KRAS-G12C/GDP Systems

**Figure 7:**
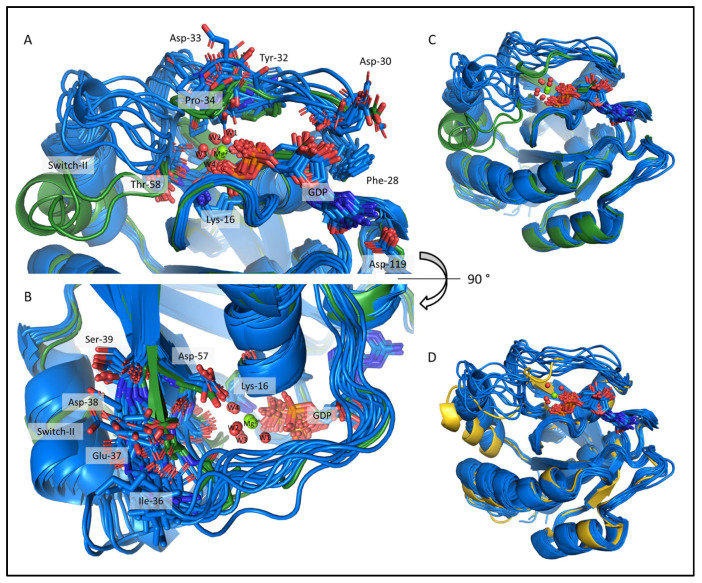
The csdMD refined structures of the Mg^2+^-bound and Mg^2+^-free KRAS-G12C proteins, shown in dark green and dark blue, respectively. The figures show the center structures of the clusters, representing 98% of the trajectory between 500 and 1000 ns. Two clusters were sufficient to describe the ensemble of the resting state KRAS-G12C/GDP-Mg^2+^, while 12 structures were required to describe the more flexible Mg^2+^-free state (RMS deviations were calculated for the extended Switch-I/II main chain plus Cβ, with a 1 Å cut-off). (**A**,**B**) The nucleotide-binging pocket and the Switch-I region from a front and top view. The sidechains of some important residues are shown as sticks. (**C**) Superimposed cluster centers of the investigated systems. (**D**) Aligned models of the KRAS-G12C/GDP Mg^2+^-free (dark blue) and the KRAS-G12C/GDP-Mg^2+^ 4OBE crystal structure (gold).

After the reinsertion of the GDP into the KRAS-G12C by the csdMD refinement, we can re-examine the impact of losing the Mg^2+^ ion (PDB files can be found in the Supplementary Materials, where 50 models represent the equilibrium part of the trajectories (500–1000 ns) including the probability of occurrence). The most apparent difference is the increased flexibility of the Mg^2+^-free state (Figure 7, RMS fluctuation plot in Figure S4C, and RMSD plots in Figure S5), which was expected since the ion acts as structure-ordering and hub-connecting residues of the P-loop (residues 10–17) and the two switch regions (residues 28–40 and 60–76) through its coordination sphere. This difference in flexibility was already present in the CS-Rosetta ensembles; however, the csdMD refinement provided a clearer and more clear-cut, structured representation of it. In the csdMD refined ensemble, the second half of Switch-I (Tyr-32, Asp-33, Pro-34, Ile-36) becomes more flexible while its N-terminal (Phe-28, Asp-30) remains well ordered. The disorder caused by the absence of the cofactor also extends to the posterior part of Switch-I, resulting in a shortening of the β2-strand of the central β-sheet of KRAS at Asp-38 and Ser-39, when compared to the cofactor-containing structure (Figure 7B and the RMSF values are shown in Figure S4E,F). We already proposed this shortening of the β2-strand and the coupled loosening of the end-segments of the Switch-regions in a previous work, where we investigated the dynamical consequences of Mg^2+^ ion loss based on NMR measurements [36].

The lack of the Mg^2+^ ion’s interaction network allows the GDP to move more frequently alongside the Switch-I region. The RMSD calculated for the centroid of the GDP nucleotide (after fitting the backbone of the non-Switch regions of the protein) with respect to its position on the crystal structure (4OBE) was 0.95 ± 0.27 Å, reflecting a 1.5-fold increase in flexibility. The Mg^2+^-free GDP-bound state is the first step of the nucleotide exchange process [28], and the increase in mobility may contribute to the GDP release. The enhanced flexibility also facilitates the binding of SOS (Son on Sevenless) as GEF, which accelerates the GDP/GTP exchange [38].

The difference in the tilt angle of Switch-II is also notable between the two sets of models. In the Mg^2+^-bound structures, Asp-57 and Thr-58 interact with the ion that fixes the entire Switch-II segment. Because of this, the α2-helix is closer to the α3-helix (see Figure 1) in our G12C KRAS-/GDP-Mg^2+^ models. In the Mg^2+^-free structure, these interactions are absent, allowing for Switch-II to fluctuate and to tilt to a steeper angle of inclination. This finding may be of significance given that the currently used small covalent inhibitors targeting G12C KRAS bind in the Switch-II pocket between helices α2 and α3 adjacent to the P-loop. Thus, the transient restructuring of this region during the nucleotide exchange step of the catalysis may provide additional protein surfaces to target—in the hope of arresting the catalytic activation of mutant variants.

In Figure S4A,B, we can observe the RMSF values of the energetically best models of the CS-Rosetta ensembles. The lowest-scoring structures exhibit similar levels of mobility, but when we examine larger portions of the ensembles, the differences become apparent. This indicates that our csdMD simulation is an effective way to represent CS-Rosetta ensembles and further investigate the structural features and dynamics of the studied systems.

### 2.5. Structure Determination of the Mg^2+^-Free and GDP-Bound KRAS-G12C by ARTINA

For comparison purposes, we also determined the NMR structure of the Mg^2+^-free form using a traditional NOE-based approach. It should be noted, however, that very few NOE cross-peaks could be assigned to the Switch regions (Figure S6C), partly due to their flexibility, and also since their nearest neighbor is the GDP nucleotide itself. We used a recently developed artificial-intelligence-based method, ARTINA [13], which created a self-generated input from the supplied various multidimensional NMR spectra and also relied on structural models derived by AlphaFold [1] (see Section 4 for details; the pdb file and information of the ARTINA calculations can be found in the Supplementary Material). In the resultant structure ensemble (Figure S6), the Switch-I and Switch-II regions were found to be in highly heterogeneous, open conformations. However, we suspect that this is mostly due to the lack of sufficient experimental NOE distance restraints, since the residues of the nucleotide-binding pocket are shown in conformations that do not allow forming the expected interactions with the GDP nucleotide (Table S1), even though it was present in the protein during data acquisition. The shortening of the β2-strand is evident in this ensemble as well as in the csdMD-refined Mg^2+^-free models, contributing to the enhanced mobility of the Switch-I region. Quality metrics were determined for the structures calculated with ARTINA and the csdMD refined models using the validation server of MolProbity [39,40], which showed that the csdMD method derived better quality models in every aspect (Table S2).

Thus, the open-conformation and structural heterogeneity of the Switch-I region of the ARTINA ensemble seems to be the joint result of the absence of the relevant NOE cross-peaks and of the GDP ligand during the model-building process. These findings reaffirm the importance of chemical-shift-based structure solution protocols and underline that protein models derived using methods that do not account for the nonprotein components must be further refined by the reinsertion of all ligands, cofactors, or ions.

## 3. Discussion

Recent advancements in NMR data processing and structure determination, such as the introduction of automated structure solution protocols such as ARTINA, have enabled the elucidation of protein structural information with minimal user intervention within a few days of data acquisition. However, despite these developments, certain structural and dynamical properties of the proteins, which might significantly influence biological function, remain hidden. Here, we introduced a protocol that is applicable to the incorporation of protein or nonprotein components that are difficult to measure, or regions where NOE cross-peaks cannot be obtained, due to inherent backbone-flexibility-induced fast relaxations and/or spectral overlap. Since the inserted cofactors as well as the flexible and pliable regions often correspond to active sites or significant loci of conformational transitions, the method we described supports a more complete structural analysis of proteins. Using this chemical-shift-based approach, relevant structural ensembles can be obtained, but potentially, the structure determination can be complemented with further NMR-based structural data, such as distance restraints.

Applying the csdMD method introduced here allowed the objective model building of two physiologically relevant systems: that of the resting state of the oncogenic KRAS-G12C and its Mg^2+^-free variant. A crystal structure exists for the former; however, that of the Mg^2+^-free state is a newly refined 3D model. We found that the loss of the Mg^2+^ ion did not lead to a drastic restructuring of the protein, but it significantly increased the conformational freedom of the Switch-I region. The increased flexibility of Switch-I upon the Mg^2+^ ion loss was also shown experimentally by fast-dynamics NMR measurements in our previous work [36], supporting our new structural ensemble. This segment of the protein also shifts, opening the nucleotide-binding pocket to the solvent, suggesting that GDP release from this state would be considerably enhanced, in accordance with experimental findings [33].

The CS-Rosetta algorithm transforms the measured chemical shifts into an ensemble of probable structures. We extracted the information pertaining to the backbone conformation in the form of weighted ϕ and ψ dihedral angle distributions that we enforced in the subsequent csdMD refinement step, which also allowed for the completion of our models. The presence of the nonprotein components, as well as the increased conformational sampling capacity of csdMD refinement, led to a more meaningful set of structures that uncovered catalytically significant differences between the two states of KRAS-G12C.

The notion of combining Rosetta modelling and MD simulation for iterative structure refinement has previously been introduced by Lindert S. et al. [41,42,43]. In a subsequent publication including chemical shift data into the MD simulation steps themselves was also introduced [43]. In this work, short MD simulation steps (1 ns) were inserted between those of the Rosetta refinement, allowing a quick relaxation of the Rosetta-derived structures, then correcting the steps taken based on the classical force field toward arrangements that comply with the measured chemical shifts. For this purpose, Lindert et al. applied PLUMED [44] to redirect the simulation based on the difference between the experimentally determined chemical shifts and those calculated using the instantaneous, force-field-derived conformation of each MD step [43]. Our csdMD protocol, on the other hand, creates a potential energy surface that incorporates chemical-shift-based structural information, and molecular dynamics is used as an efficient sampling method for the thorough analysis (1000 ns, in this work) of this experiment-based conformational space. Replacing the dihedral energy terms of the classical force field by the Rosetta-score-weighted dihedral angle distribution of the Rosetta ensemble, eliminates the need for the continuous recalculation of the chemical shifts of the generated conformers, making this method computationally less demanding. However, more importantly, our approach generates an equilibrated ensemble that satisfies the experimentally determined chemical shifts and thus provides not only the most probable structures but the coupled conformational heterogeneity and dynamic properties of the studied system.

## 4. Materials and Methods

### 4.1. Protein Expression and Purification

The expression and purification of the ^13^C-^15^N-labelled KRAS (1-169) G12C protein was performed by using a methodology published previously [45]. For the cofactor-free protein, the Mg^2+^ ion was removed during size exclusion chromatography by adding 10 mM EDTA instead of 10 mM MgCl_2_ to the PBS elution buffer.

### 4.2. NMR Spectroscopy

NMR samples contained 0.7–1 mM KRAS, 10 mM MgCl_2_ for the Mg^2+^-bound samples or 10 mM EDTA for the Mg^2+^-free samples, 3 mM NaN_3_ in PBS, 7% D_2_O, 1% DSS at pH = 7.4. For the Mg^2+^-bound KRAS structure calculations, our previous assignment was used, deposited in the Biological Magnetic Resonance Data Bank (BMRB) database (entry: 27646). To complete our previous assignment of Mg^2+^-free KRAS-G12C/GDP [36], further 4D spectra were recorded according to the method described earlier [37]. Four-dimensional HC(CC-TOCSY(CO))NH with nonuniform sampling (1% of the total number of points) was recorded on a Bruker Avance III HD 700 MHz spectrometer (Bruker Daltonics GmbH & Co. KG, Bremen, Germany) (700.17 MHz for ^1^H, 176.06 MHz for ^13^C, 70.95 MHz for ^15^N) equipped with a ^1^H{^31^P/^13^C/^15^N} QCI cryoprobe and 4D ^13^C, ^15^N-edited SOFAST-HMQC-NOESY-SOFAST-HMQC (HCNH) with nonuniform sampling (1.48% of the total number of points) was recorded on a Bruker Avance NEO 900 MHz spectrometer (900.3 MHz for ^1^H, 226.38 MHz for ^13^C, 91.23 MHz for ^15^N) equipped with a ^1^H{^13^C/^15^N} TCI cryoprobe. The chemical shift assignment derived from the 4D spectra was used for the CS-Rosetta structure determination. For the structure calculation by ARTINA our previously recorded 3D spectra were used (namely 3D BEST-TROSY-HNCO, BEST-TROSY-HN(CA)CO, BEST-TROSY-HNCA, BEST-TROSY-HN(CO)CA, BEST-TROSY-HNCACB, BEST-TROSY-HN(CO)CACB, CCH-TOCSY, ^15^N-NOESY-HSQC) [23] recorded on a Bruker Avance Neo 700 MHz spectrometer (700.25 MHz for ^1^H, 176.09 MHz for ^13^C, 70.96 MHz for ^15^N) equipped with a 5 mm Prodigy H&F-C/N-D, a z-gradient probe head, and further recorded spectra (2D ^1^H,^15^N-HSQC, ^1^H,^13^C-HSQC, 3D ^15^N-TOCSY-HSQC, ^13^C-HCCH-TOCSY, ^13^C-NOESY-HSQC, and CC(CO)NH) were recorded on a Bruker Avance III 700 MHz spectrometer (700.05 MHz for ^1^H, 176.03 MHz for ^13^C, 70.94 MHz for ^15^N) equipped with a 5 mm Prodigy TCI H&F-C/N-D, z-gradient probe head. Measurements were performed at 298 K. The temperature was calibrated against a standard methanol solution. ^1^H chemical shifts were referenced with respect to the ^1^H resonance of the internal DSS, whereas ^13^C and ^15^N-chemical shifts were referenced indirectly using the corresponding gyromagnetic ratios according to IUPAC convention. All spectra were processed with the Bruker TOPSPIN 3.6 and TOPSPIN 4.1.

### 4.3. CS-Rosetta Structure Determination

The 4D spectra were assigned by using 4D-CHAINS [37] and our previous backbone assignment [36] to obtain a full assignment including sidechains of Mg^2+^-free KRas-G12C/GDP (BMRB entry: 52009). CS-Rosetta version 3.6 and Rosetta release version 2021.16 were applied. A fragment library was built using the sequence and the chemical shifts, which contained many possible conformations for a given set of degrees of freedom. The library contained homologous protein structures as well, such as multiple nucleotide-binding proteins. The fragments were assembled to a nativelike protein structure—which was missing the GDP and the Mg^2+^ ion—using the standard ab initio relax protocol of Chemical-Shift-Rosetta [20] based solely on sequence and chemical shifts. Ten thousand all-atom models were calculated.

### 4.4. csdMD Model Refinement

For the csdMD simulations, CS-Rosetta ensemble biased backbone dihedral-angle potential energy functions (PEFs) were built. First, based on the distribution of the residues’ ϕ or ψ angles inside the ensembles, continuous probability density functions (PDFs) were calculated using the kernel density estimation method. The chosen kernel was a power-raised cosine kernel, shown in Equation (1):

(1) $$\;K\left(\alpha ,\alpha_{ij}\right)=\cos^{k}\left(∆\left(\alpha ,\alpha_{ij}\right)\cdot \frac{\pi }{360{}^{\circ}}\right)$$

*K* is the kernel value at *α*, *α_ij_* is the *i*th angle-value (ϕ or ψ) in the ensemble’s *j*th structure, *k* determines the width of the kernel, and Δ is the distance of two angles on a circular topology (Equation (2)):

(2) $$∆\left(\alpha ,\alpha_{ij}\right)=\left\{\begin{array}{c} \left|\alpha -\alpha_{ij}\right|,\;\;\;\;\;\;\;\;\;\;\;\;\;\;\mathrm{if}\;\left|\alpha -\alpha_{ij}\right|<180{}^{\circ}\\ 360{}^{\circ}-\left|\alpha -\alpha_{ij}\right|,\;\;\;\;\;\;\;\mathrm{else}\;\;\;\;\;\;\;\;\;\;\;\;\;\; \end{array}\right.$$

Equation (2) ensures that Δ is between 0° and 180°. The parameter *k* is directly related to the kernel width *w* (Equation (3)):

(3) $$k=1+\frac{1}{{tan}^{2}\left(\frac{w}{2}\right)}$$

We calculated the width using Equation (4), which is as follows:

(4) $$w=\frac{2\pi }{\sqrt[{3}]{N}}$$

The value of the width depends on *N*, the number of structures inside the ensemble, similarly as in Scott’s normal reference rule, ref. [46] or in the Freedman–Diaconis rule [47]. The PDF of each *i*th angle was constructed from the individual kernels (Equation (5)):

(5) $$P_{i}\left(\alpha \right)=\frac{1}{C}\sum_{j=1}^{N}c_{j}\cdot K\left(\alpha ,\alpha_{ij}\right)$$

where *C* is the sum of all weights *c_j_*, and *c_j_* is a weight determined from the corresponding structure’s total Rosetta score *s_j_* (Equation (6)):

(6) $$c_{j}=exp\left(-\frac{s_{j}}{s_{ref}}\right)$$

The reference score *s_ref_* was chosen as 10, which seemed to be the appropriate magnitude to scale down the score differences between the good, low-scoring, similar models, but large enough to reduce the contribution of the bad, high-scoring models, with high RMSD. From the PDFs, knowledge-based PEFs were calculated assuming a Boltzmann distribution (Equation (7)):

(7) $$E_{i}\left(\alpha \right)=-f_{i}\cdot ln\left(P_{i}\left(\alpha \right)\right)$$

The force-scaling factors *f_i_* were chosen so that they rescaled the standard deviation of the PEFs (to 14.77 and 5.30 times for the ϕ and ψ angles, respectively) as large as the standard deviation of the corresponding dihedral-angle potential term in the AMBER-ff99SBildnp* force field.

The initial models for the csdMD simulations were built from the best CS-Rosetta models. The best models (2-168) were extended by adding the missing first and last residues of the sequence (1-169) and optimizing their conformation to avoid clashes using Schrödinger Maestro 2022.4 software (Schrödinger, LLC, New York, NY, USA, 2021), while freezing the rest of the molecule. When a sidechain (in our case only Tyr-32) was in a clashing conformation when the nonprotein components were reinserted, i.e., occupying the nucleotide-binding pocket, it was rotated out of this cavity using the rotamer library. The nonprotein moieties were added based on the crystal structure of 4OBE. The models were solvated in water using the OPC water model [48], and the systems were neutralized with sodium and chloride ions at physiological salt concentration (0.15 M). The simulations were carried out using GROMACS 2022.2 [49] at 350 K using the AMBER-ff99SBildnp* force field [50] with the parametrization of Steinbrecher et al. for the phosphate moieties [51].

### 4.5. ARTINA Calculations

For the ARTINA [13] structure determination of Mg^2+^-free KRAS-G12C/GDP the following spectra were used as an input: 2D ^1^H,^15^N-HSQC and ^1^H,^13^C-HSQC, 3D BEST-TROSY-HNCO, BEST-TROSY-HN(CA)CO, BEST-TROSY-HNCA, BEST-TROSY-HN(CO)CA, BEST-TROSY-HNCACB, BEST-TROSY-HN(CO)CACB, CC(CO)NH, CCH-TOCSY, ^15^N-NOESY-HSQC, ^15^N-TOCSY-HSQC, ^13^C-HCCH-TOCSY, and ^13^C-NOESY-HSQC. AlphaFold [1] was used to generate a structure prediction based on the residues’ sequence, and this structure was also used as an input for ARTINA. The calculation was performed using the NMRtist platform [14]. The total number of distance restraints in the final calculation was 2200, and the number of long-distance NOE cross-peaks (where the given amino acids were more than three residues apart) was 637.

## Data Availability

The NMR chemical shift assignments are available in the BMRB (https://bmrb.io/, accessed on 24 July 2023) under entries 27646 and 52009. The Python3 code for the structure refinement was uploaded to GitHub (https://github.com/fazekaszs/ensemble_to_gromacs, accessed on 24 July 2023).

## Supplementary Materials

The following supporting information can be downloaded at: https://www.mdpi.com/article/10.3390/ijms241512101/s1.

## Abbreviations

BMRB	Biological Magnetic Resonance Data Bank
csdMD	Chemical-shift-driven MD
CS-Rosetta	Chemical-Shift-Rosetta
χ_1_	Chi-1 dihedral (or torsion) angle
GDP	Guanosine diphosphate
GEF	Guanosine exchange factor
GTP	Guanosine triphosphate
MD	Molecular dynamics
NMR	Nuclear magnetic resonance
NOE	Nuclear Overhauser effect
ω	Omega dihedral (or torsion) angle
ϕ	Phi dihedral (or torsion) angle
PEF	Potential energy function
PDB	Protein Data Bank
ψ	Psi dihedral (or torsion) angle
RMSD	Root-mean-square deviation
RMSF	Root-mean-square fluctuation
SOS	Son of Sevenless
