# Supplementary figures and images for: NMR-Chemical-Shift-Driven Protocol Reveals the Cofactor-Bound, Complete Structure of Dynamic Intermediates of the Catalytic Cycle of Oncogenic KRAS G12C Protein and the Significance of the Mg2+ Ion (part 2 of 2)
Source: Int J Mol Sci. 2023 Jul 28;24(15):12101. doi: 10.3390/ijms241512101 (PMC10418480; doi:10.3390/ijms241512101)

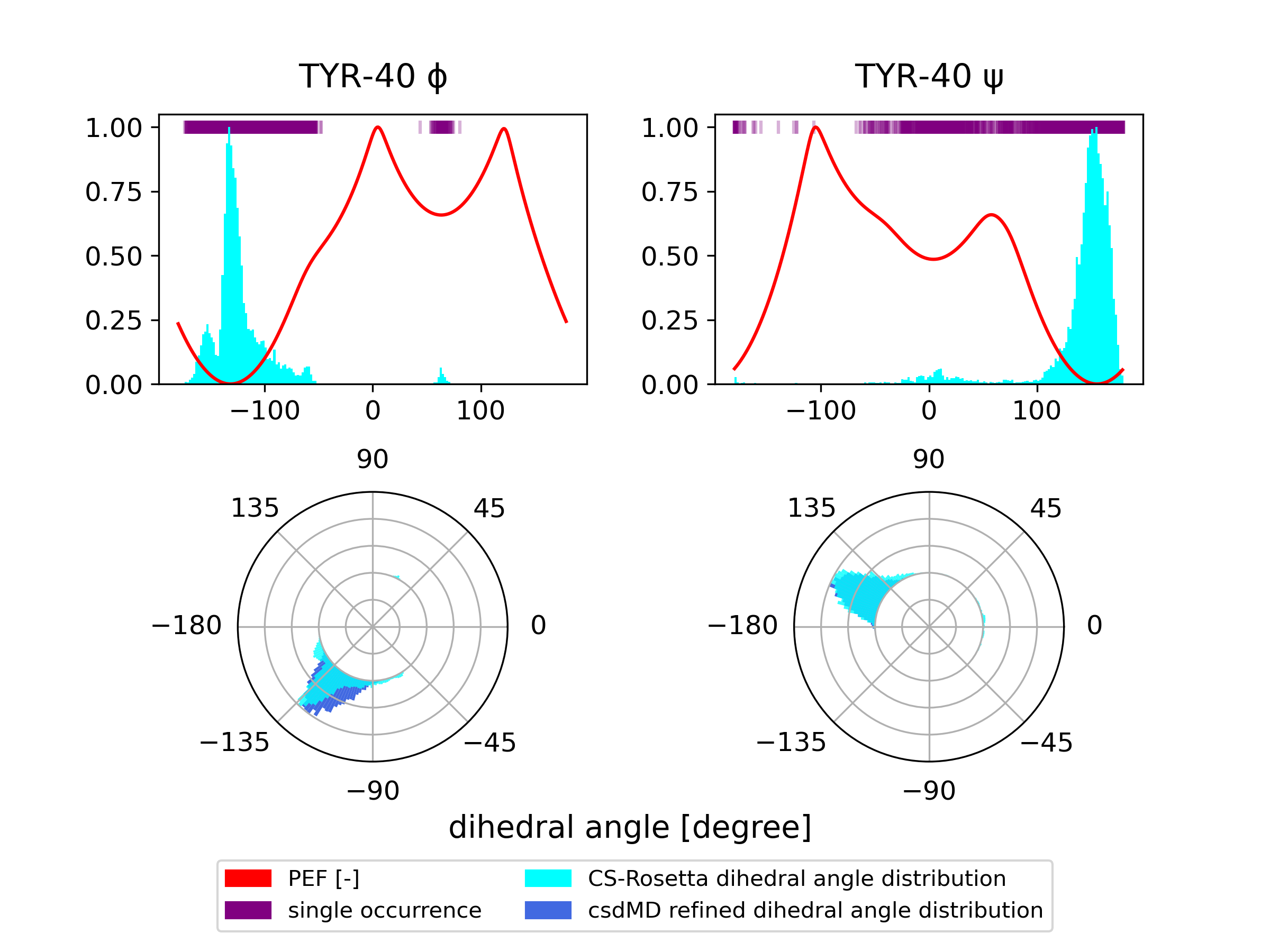

Supplement: Supplementary file 1 [file ijms-24-12101-s001.zip › KRAS-G12C-GDP-Mg-free_angle_figures/40-TYR.png]

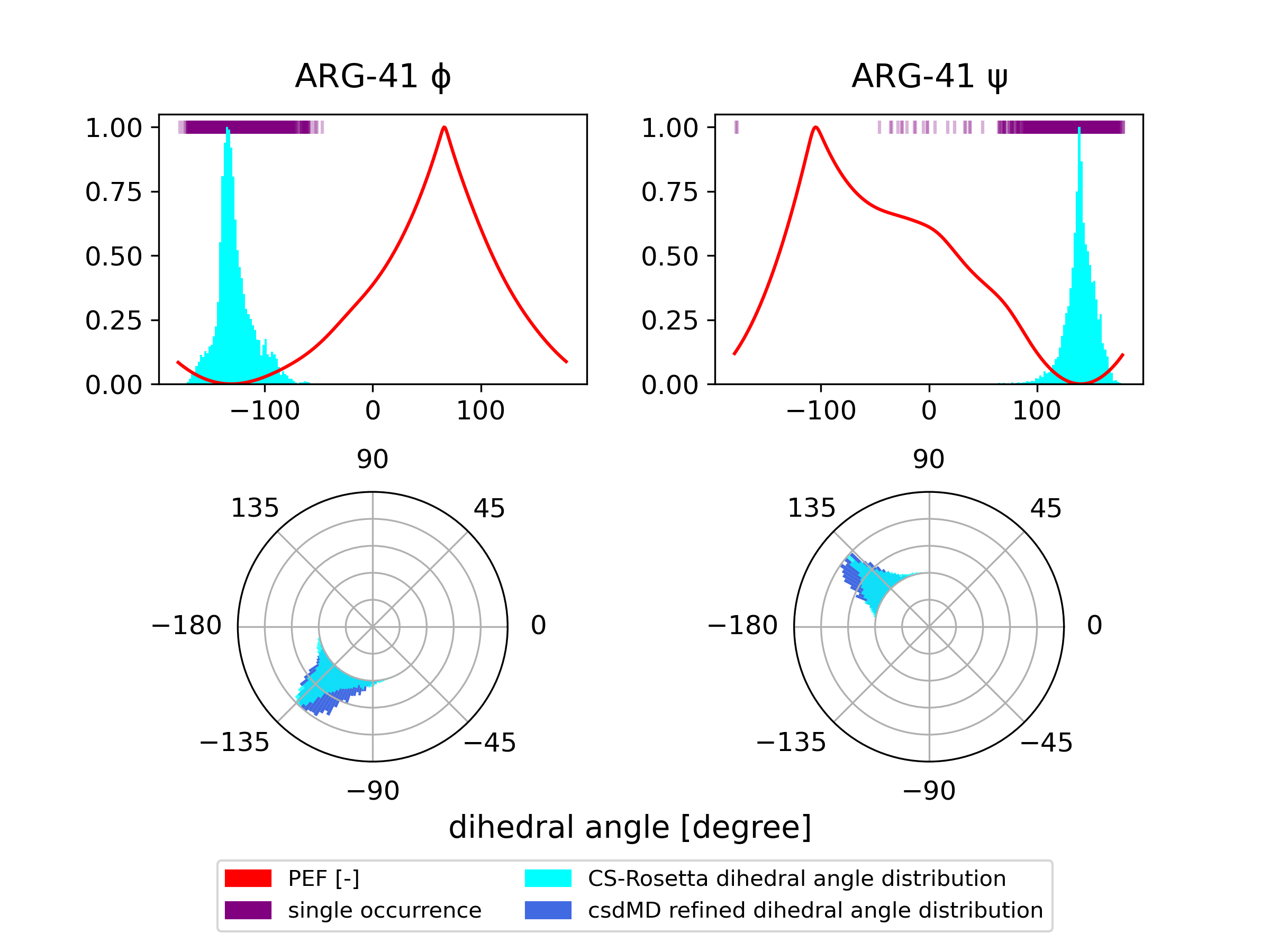

Supplement: Supplementary file 1 [file ijms-24-12101-s001.zip › KRAS-G12C-GDP-Mg-free_angle_figures/41-ARG.png]

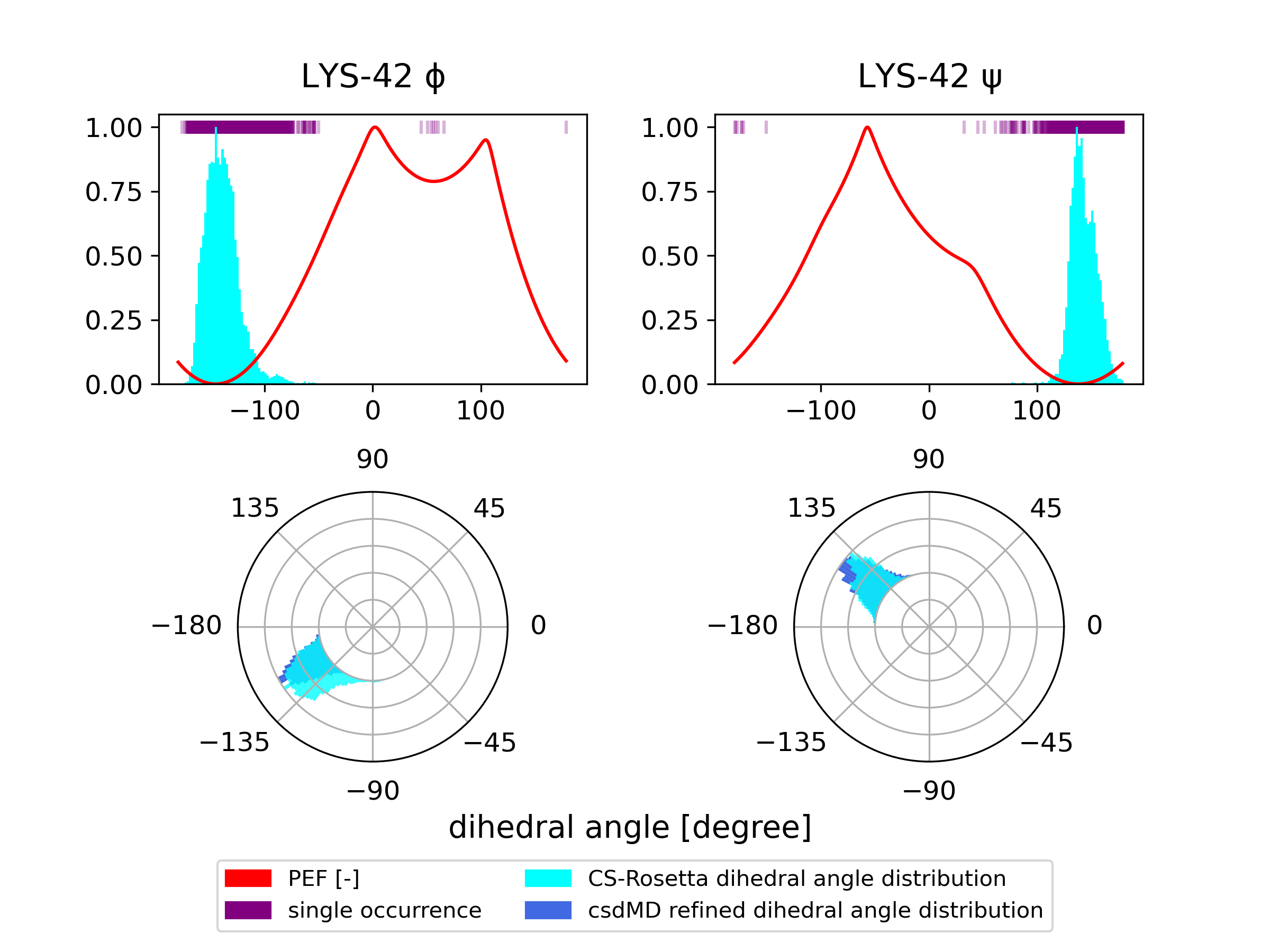

Supplement: Supplementary file 1 [file ijms-24-12101-s001.zip › KRAS-G12C-GDP-Mg-free_angle_figures/42-LYS.png]

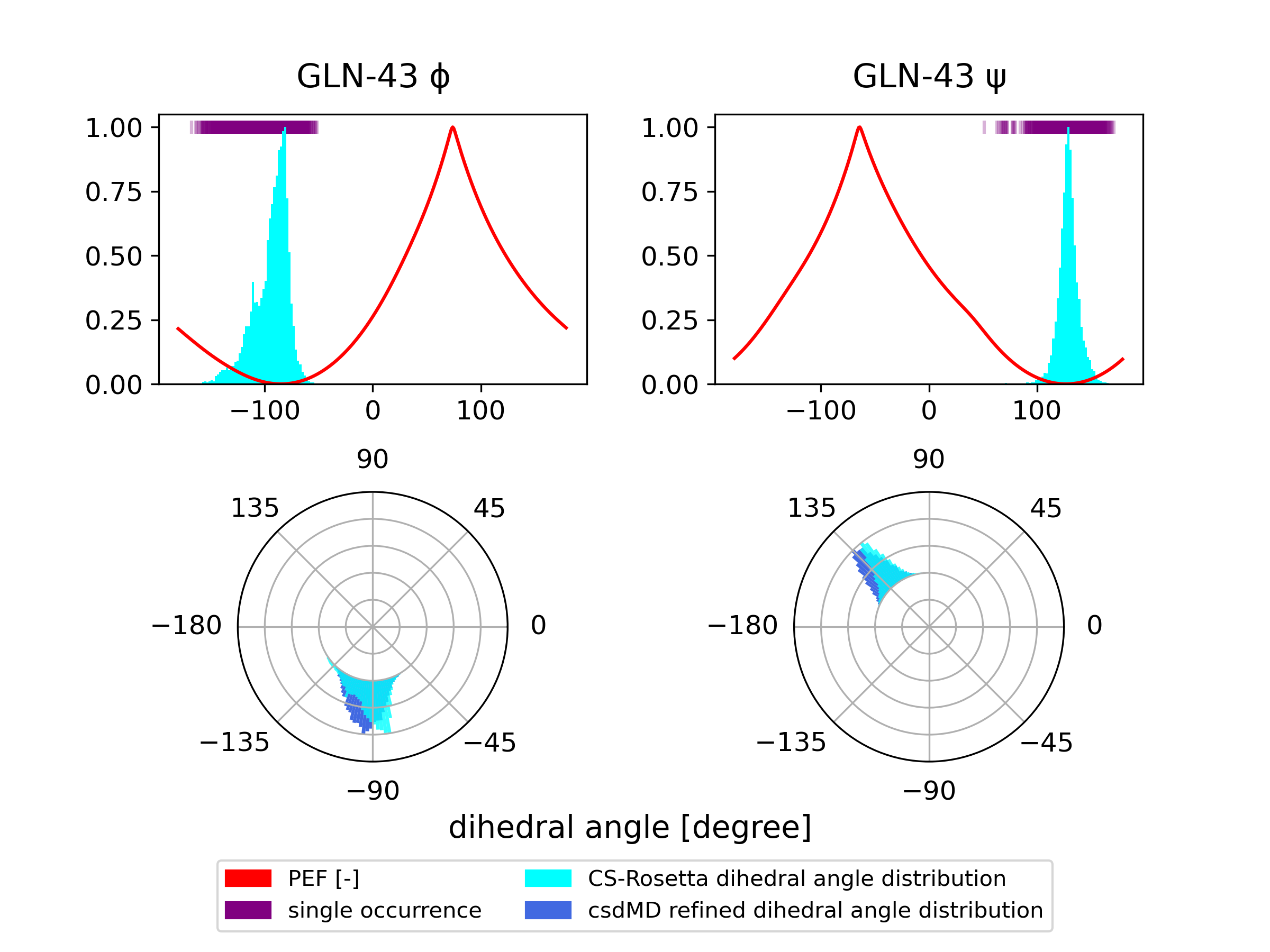

Supplement: Supplementary file 1 [file ijms-24-12101-s001.zip › KRAS-G12C-GDP-Mg-free_angle_figures/43-GLN.png]

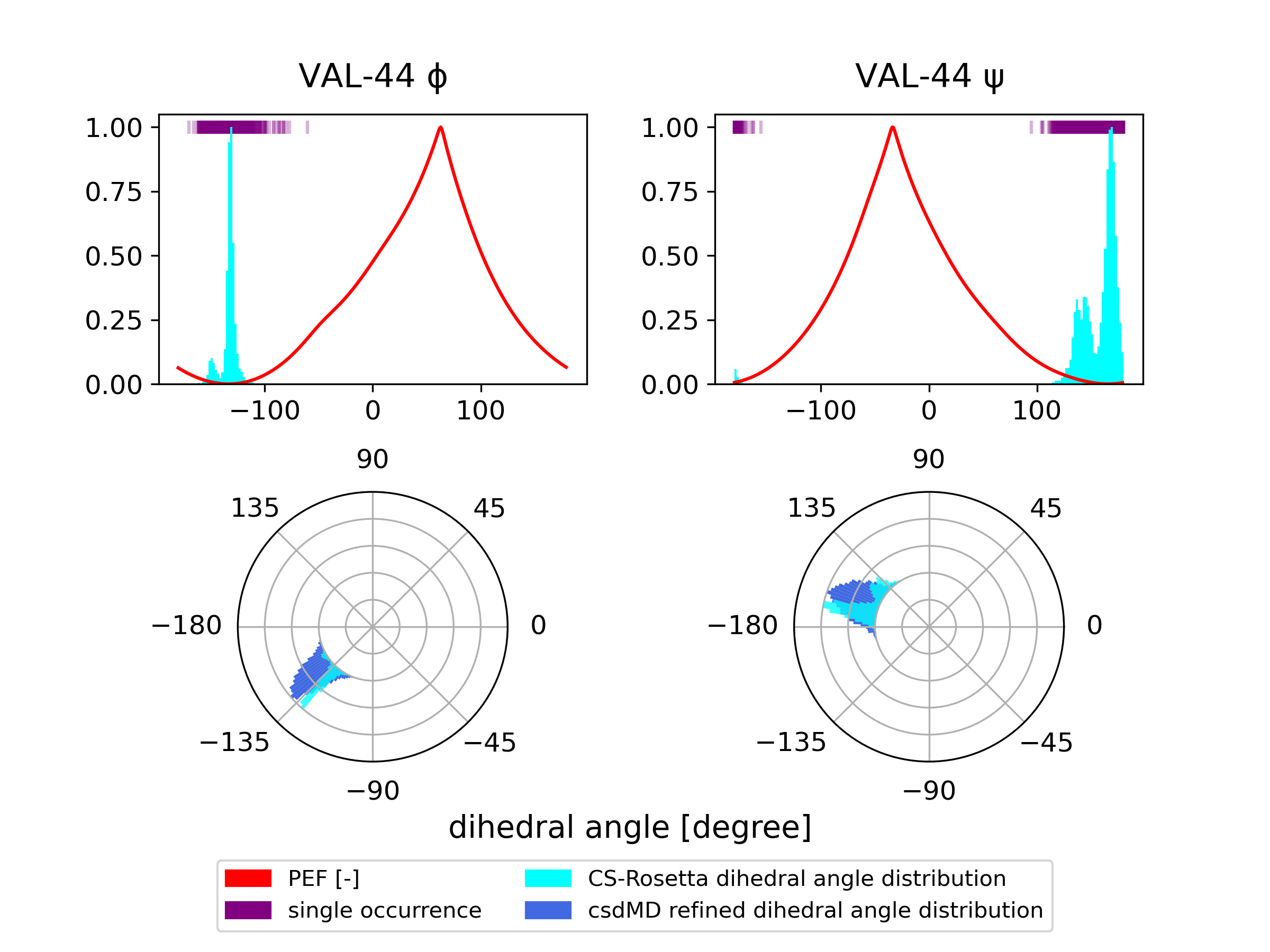

Supplement: Supplementary file 1 [file ijms-24-12101-s001.zip › KRAS-G12C-GDP-Mg-free_angle_figures/44-VAL.png]

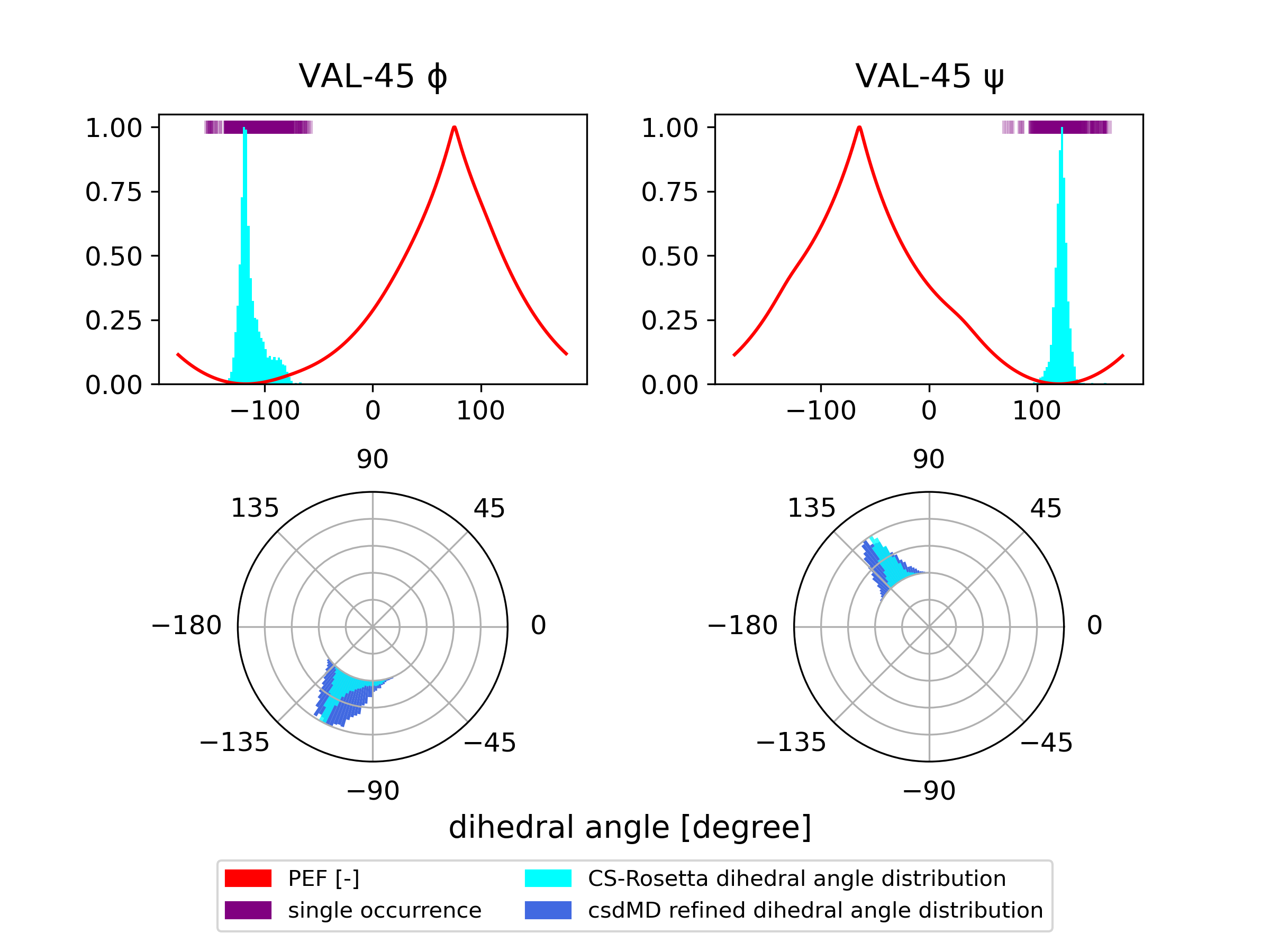

Supplement: Supplementary file 1 [file ijms-24-12101-s001.zip › KRAS-G12C-GDP-Mg-free_angle_figures/45-VAL.png]

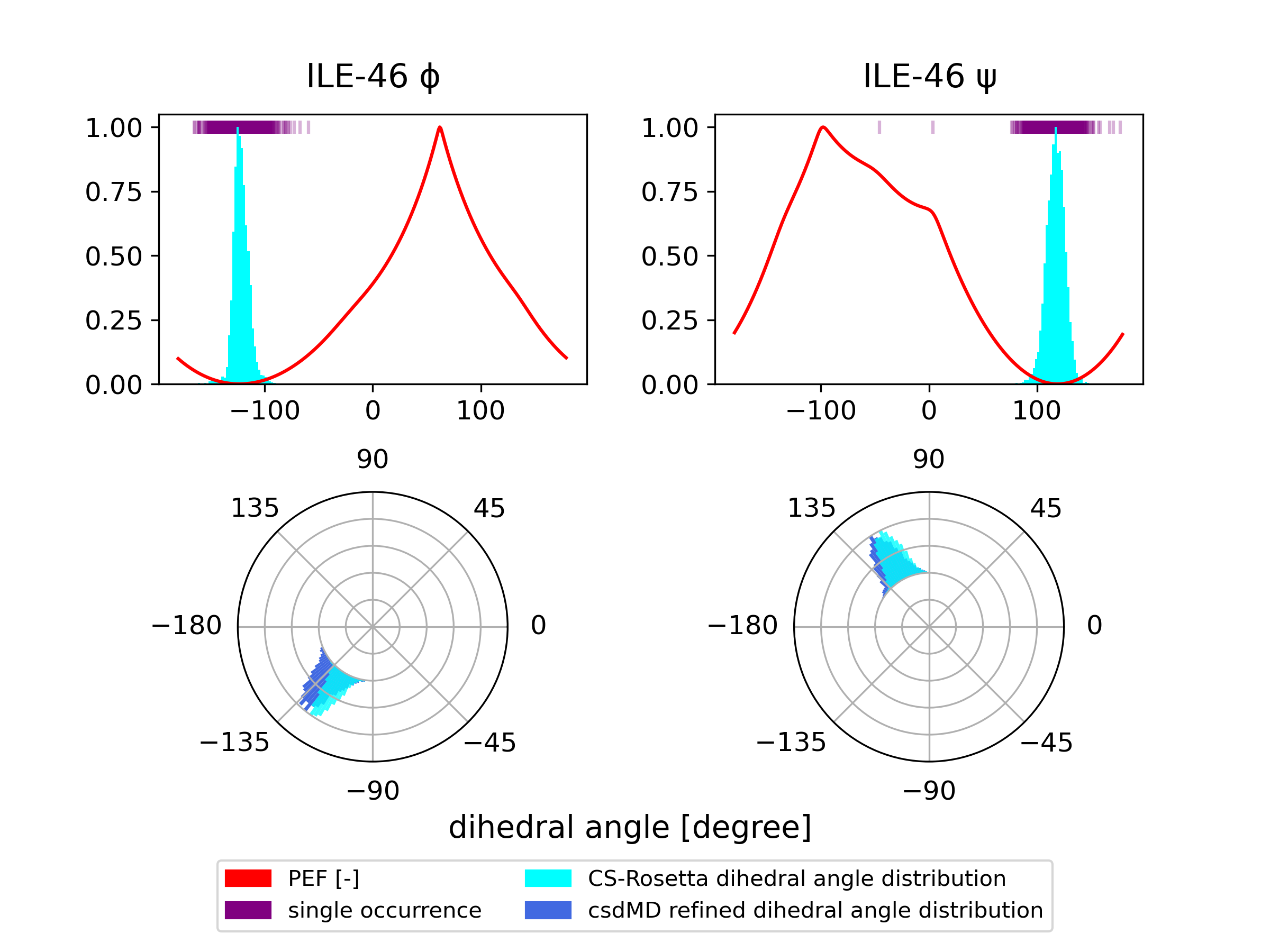

Supplement: Supplementary file 1 [file ijms-24-12101-s001.zip › KRAS-G12C-GDP-Mg-free_angle_figures/46-ILE.png]

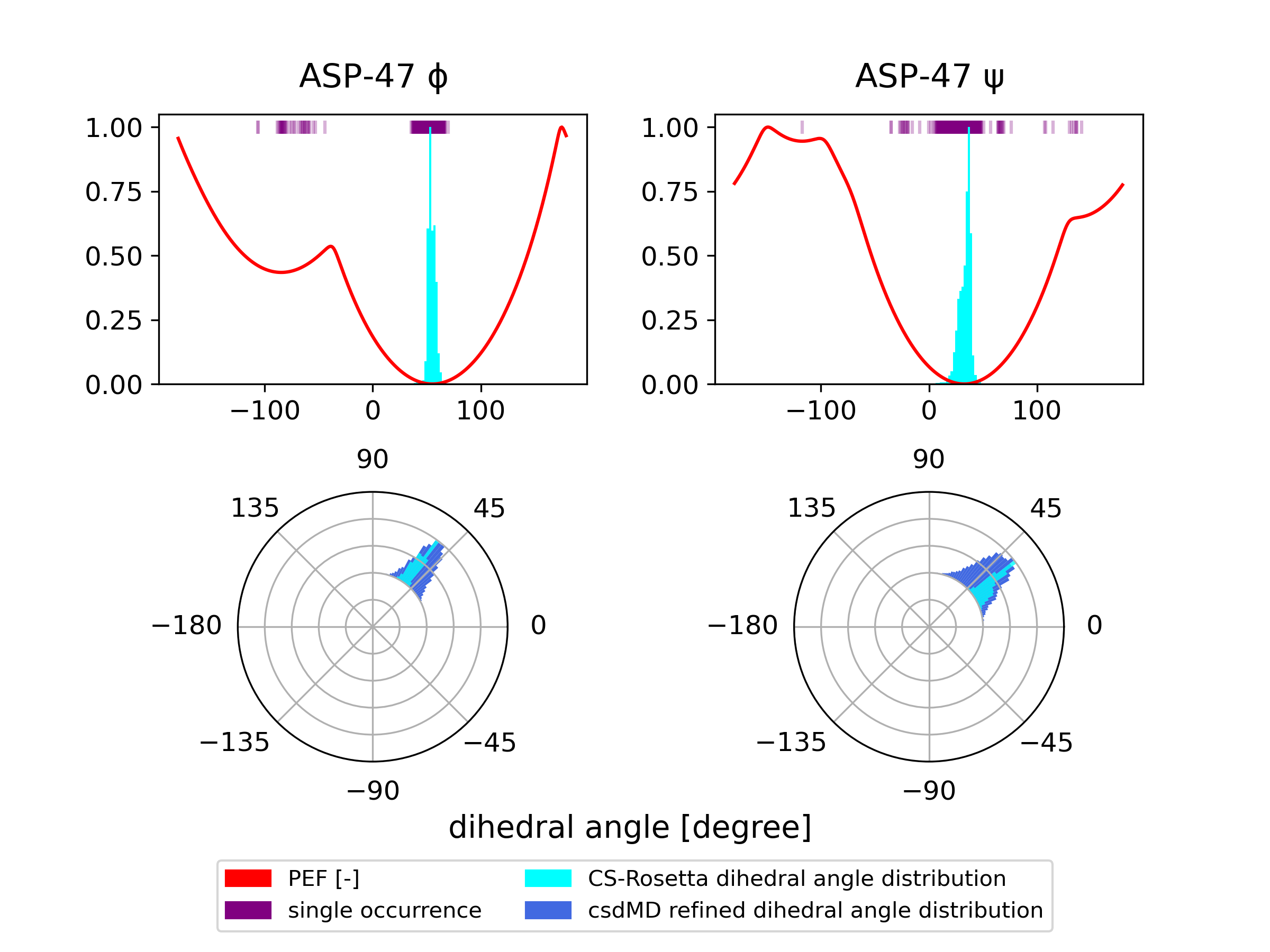

Supplement: Supplementary file 1 [file ijms-24-12101-s001.zip › KRAS-G12C-GDP-Mg-free_angle_figures/47-ASP.png]

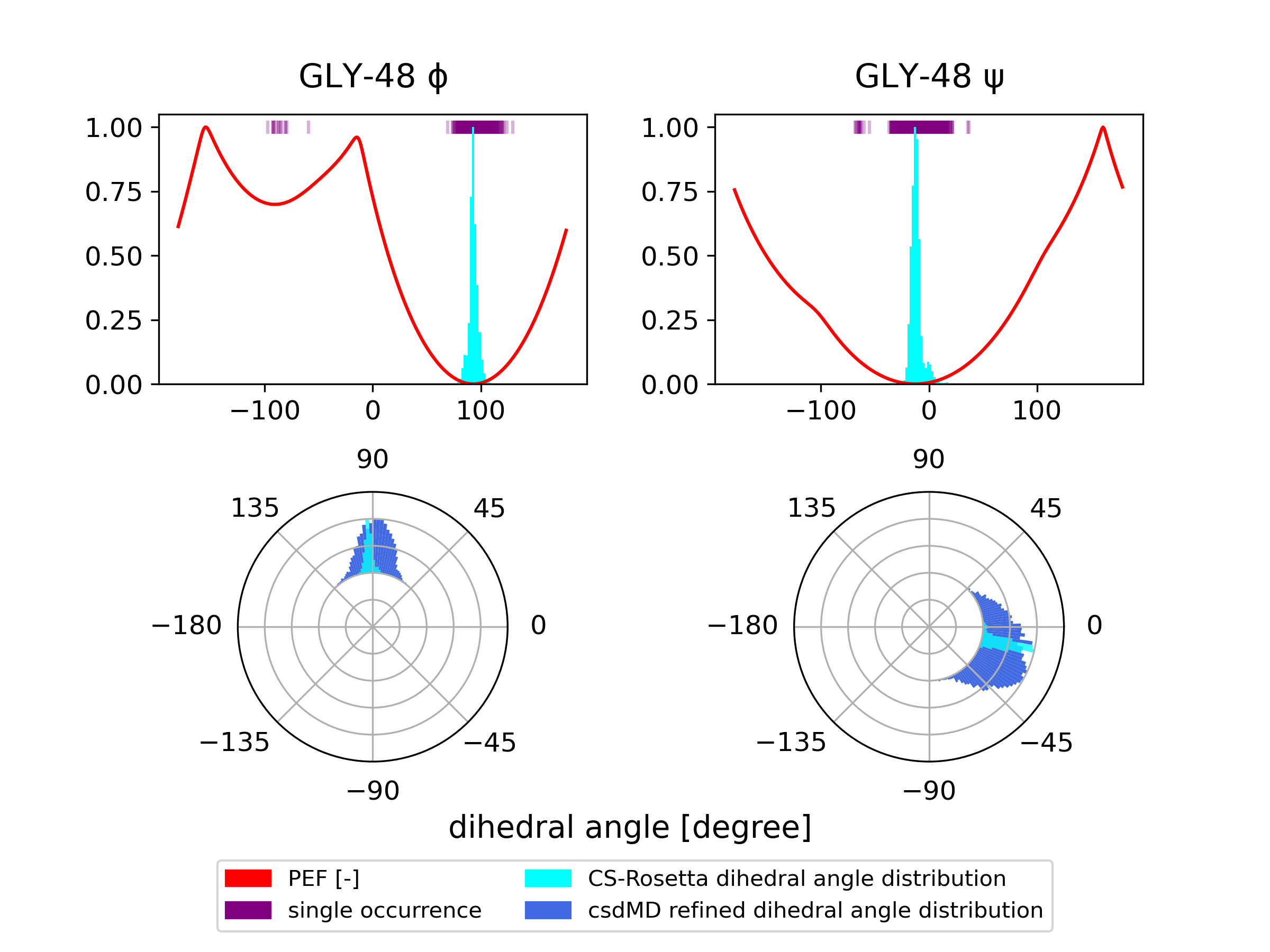

Supplement: Supplementary file 1 [file ijms-24-12101-s001.zip › KRAS-G12C-GDP-Mg-free_angle_figures/48-GLY.png]

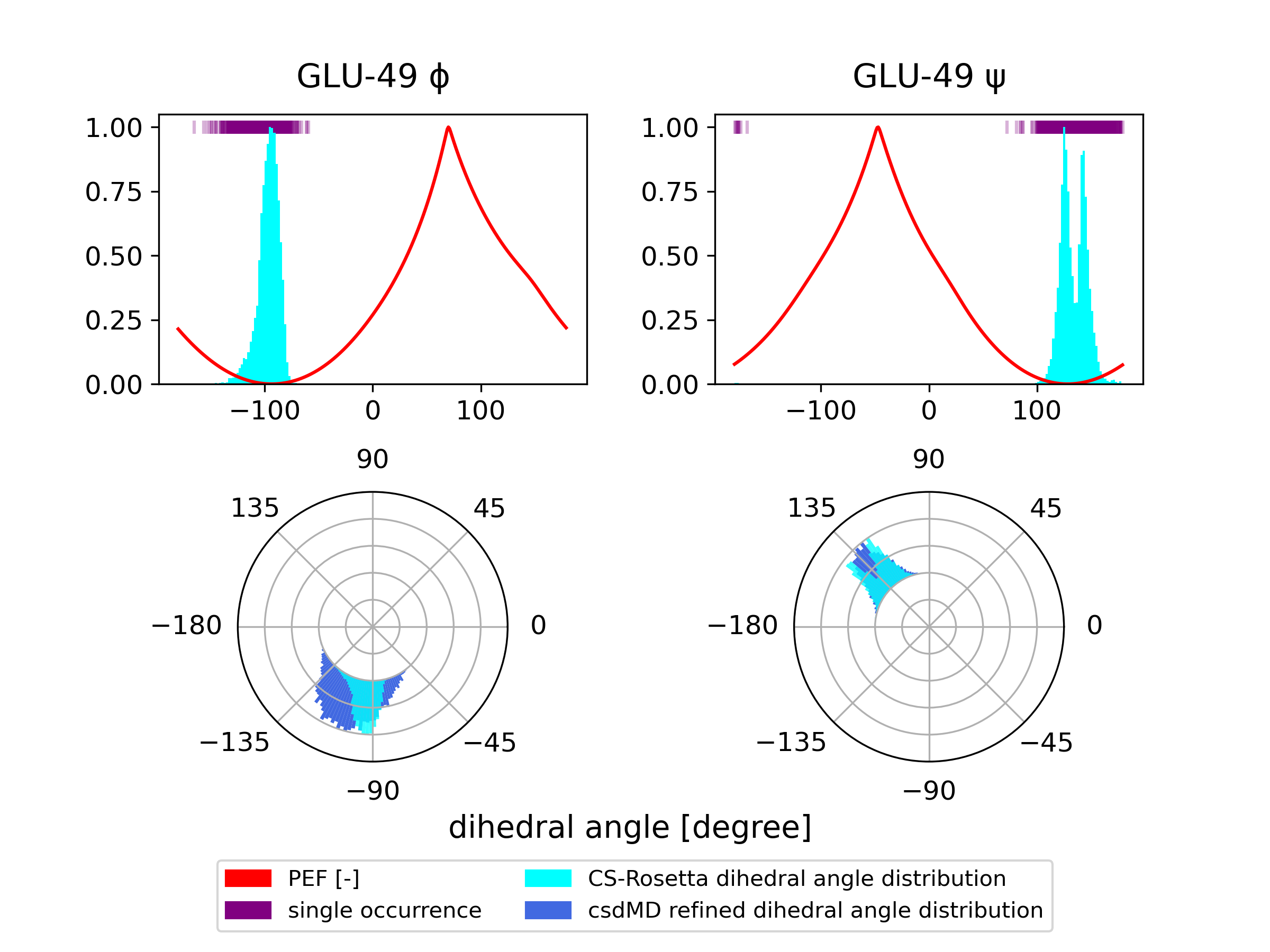

Supplement: Supplementary file 1 [file ijms-24-12101-s001.zip › KRAS-G12C-GDP-Mg-free_angle_figures/49-GLU.png]

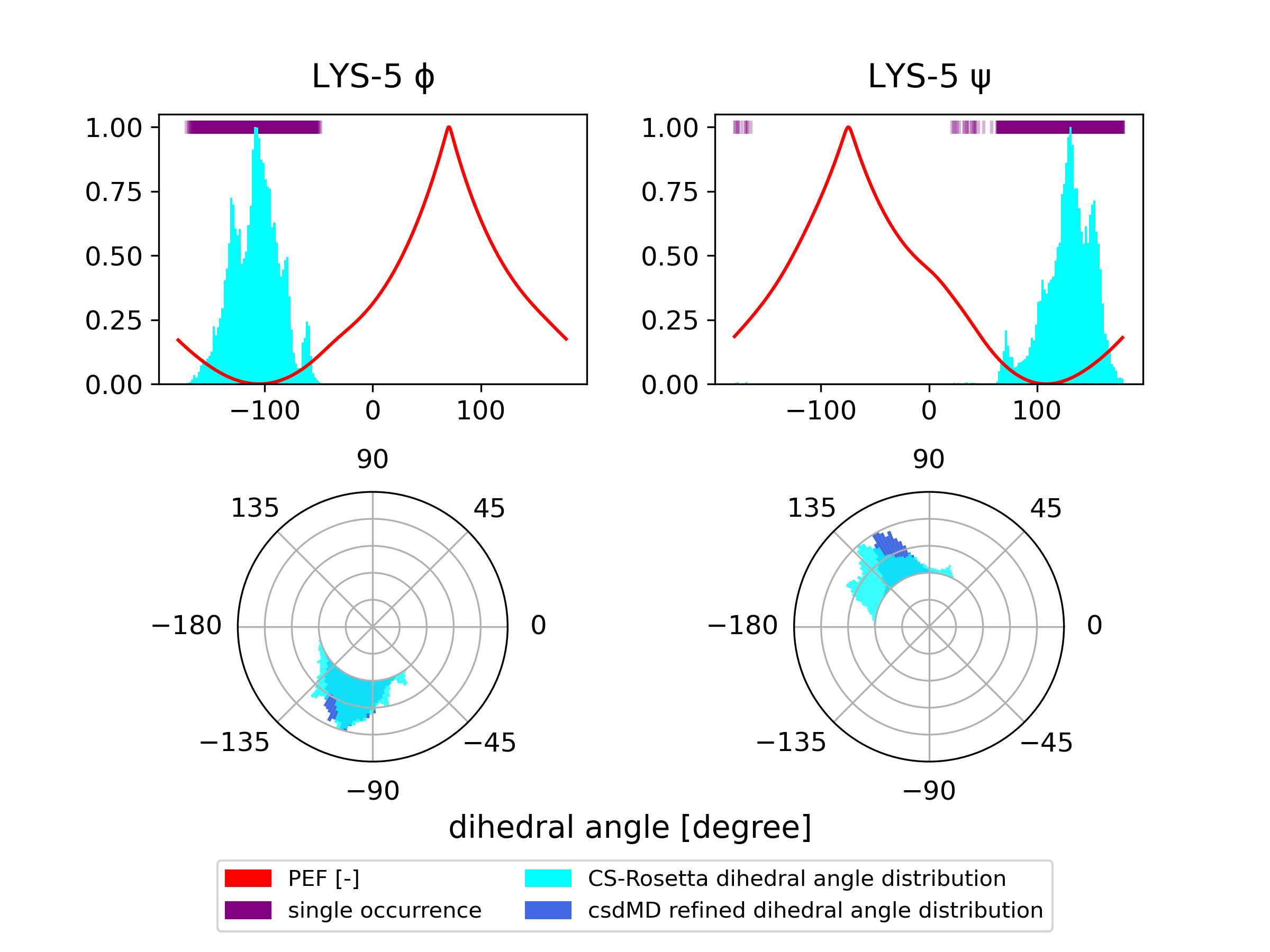

Supplement: Supplementary file 1 [file ijms-24-12101-s001.zip › KRAS-G12C-GDP-Mg-free_angle_figures/5-LYS.png]

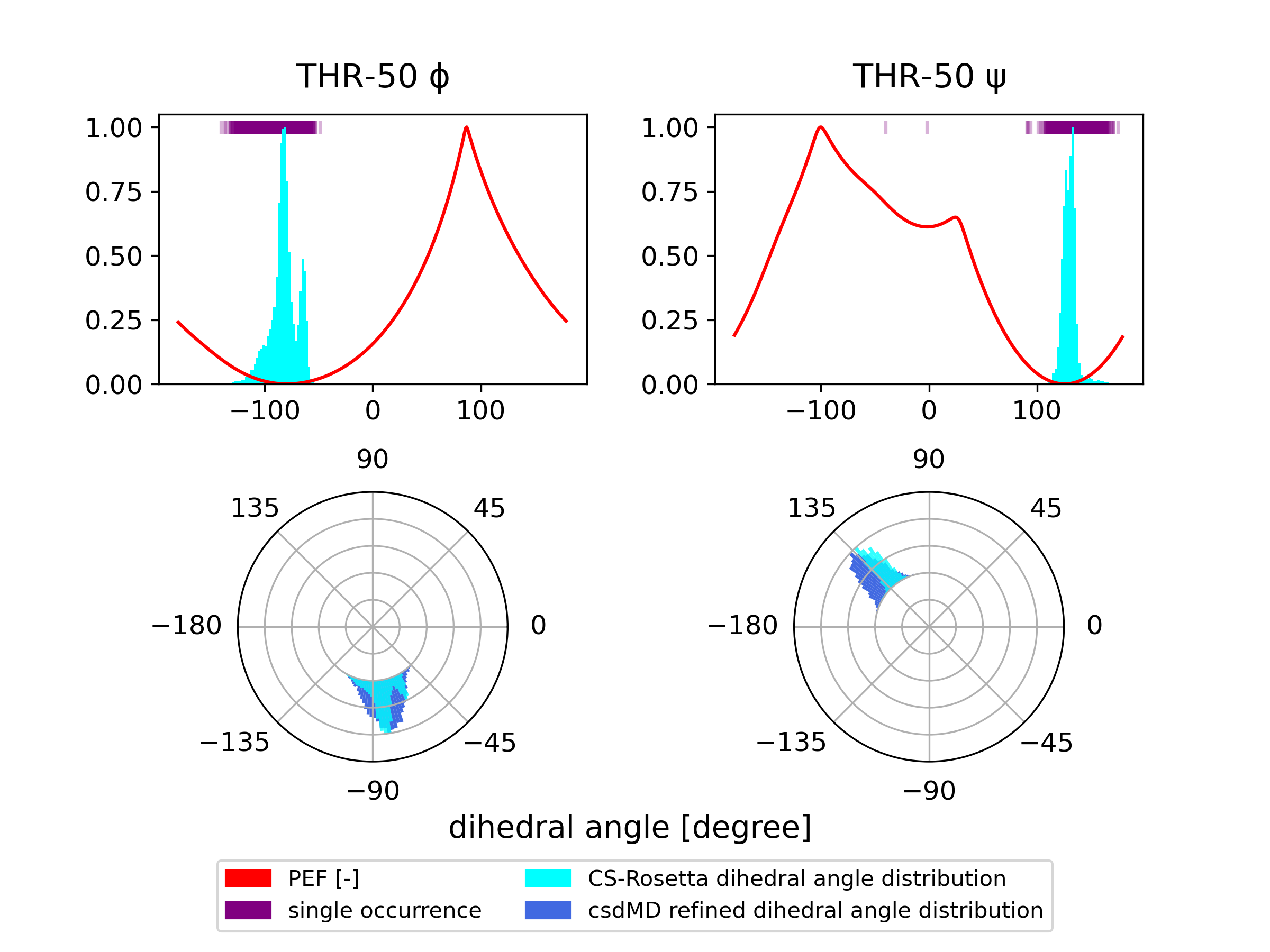

Supplement: Supplementary file 1 [file ijms-24-12101-s001.zip › KRAS-G12C-GDP-Mg-free_angle_figures/50-THR.png]

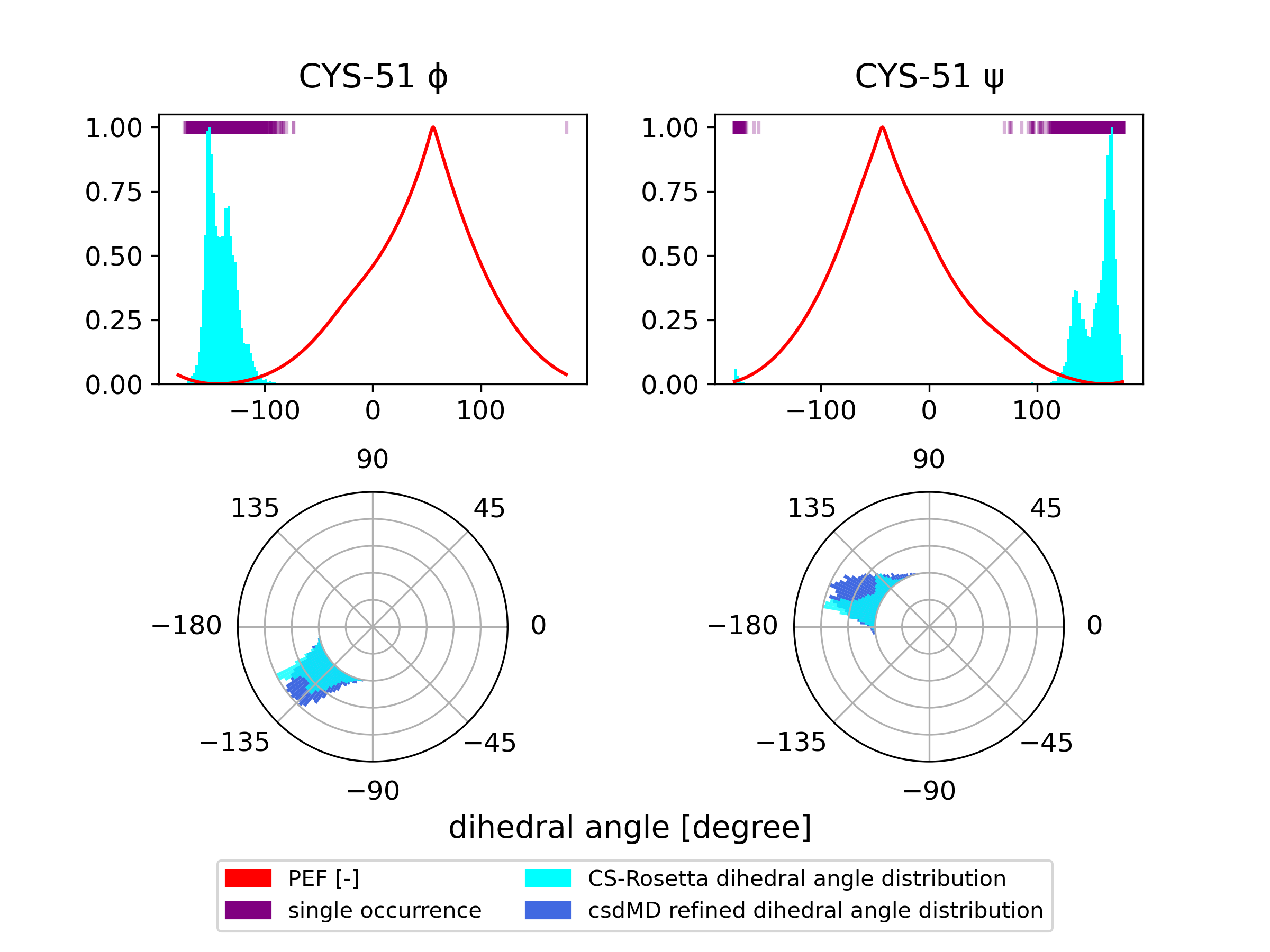

Supplement: Supplementary file 1 [file ijms-24-12101-s001.zip › KRAS-G12C-GDP-Mg-free_angle_figures/51-CYS.png]

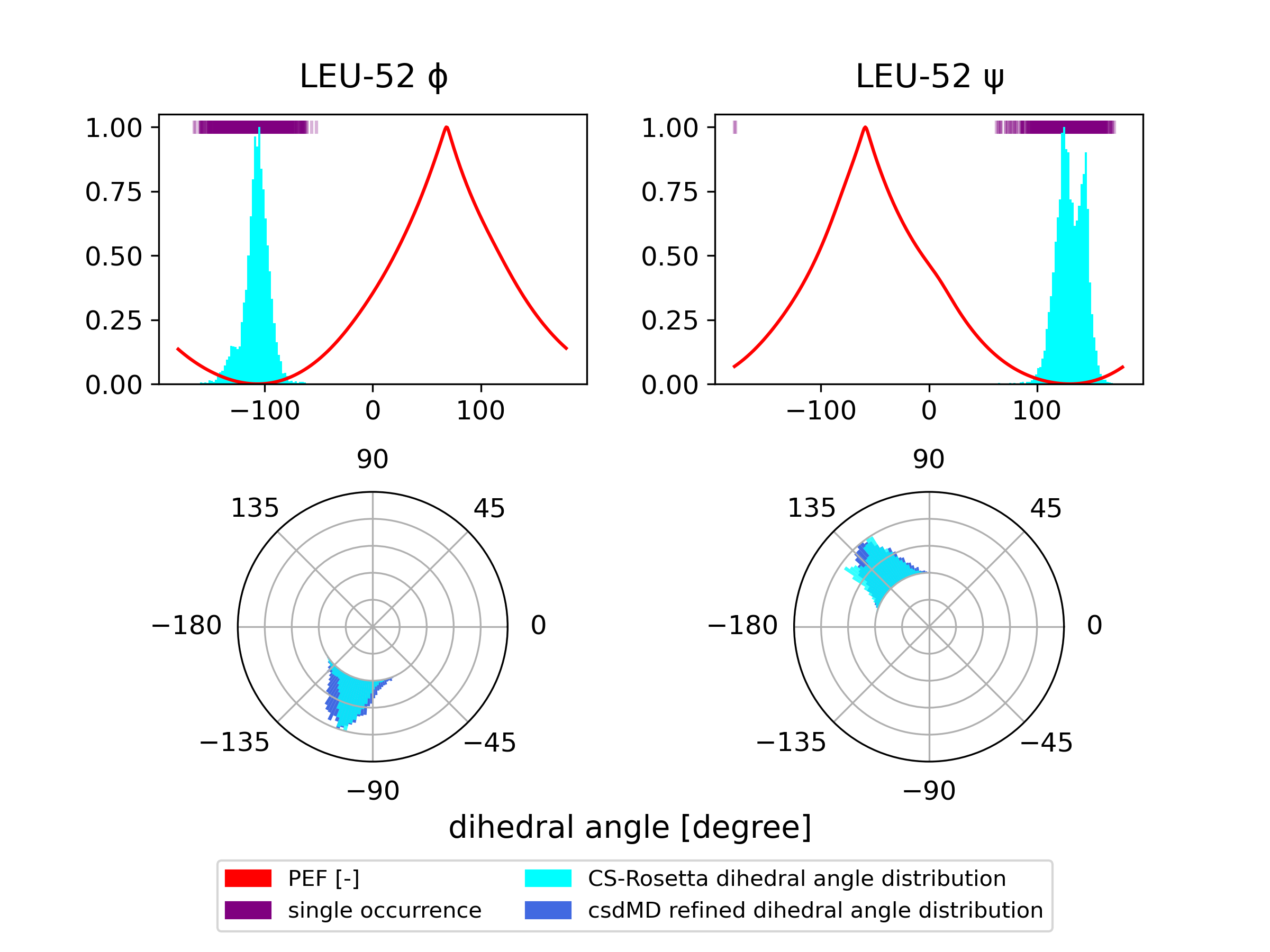

Supplement: Supplementary file 1 [file ijms-24-12101-s001.zip › KRAS-G12C-GDP-Mg-free_angle_figures/52-LEU.png]

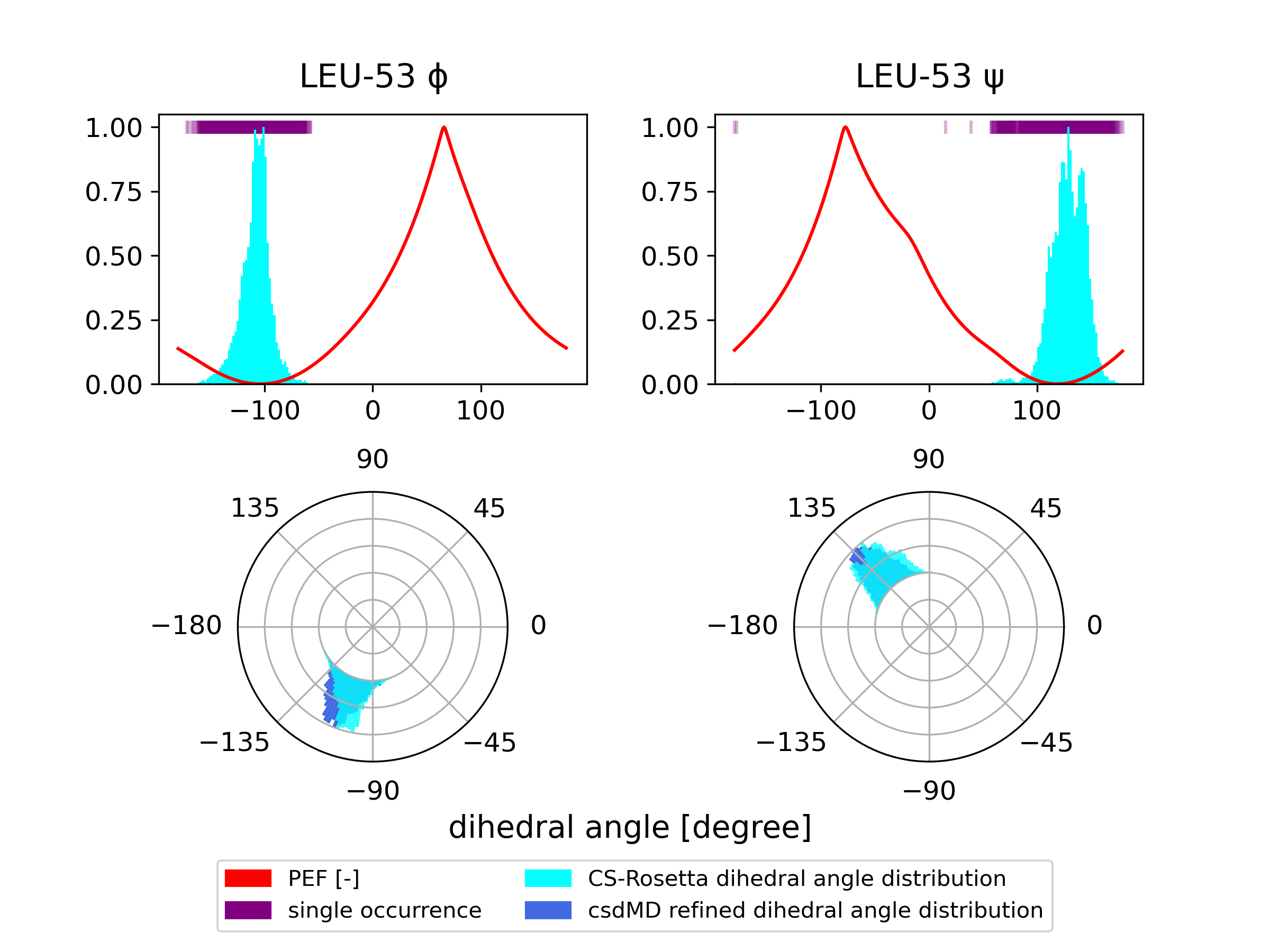

Supplement: Supplementary file 1 [file ijms-24-12101-s001.zip › KRAS-G12C-GDP-Mg-free_angle_figures/53-LEU.png]

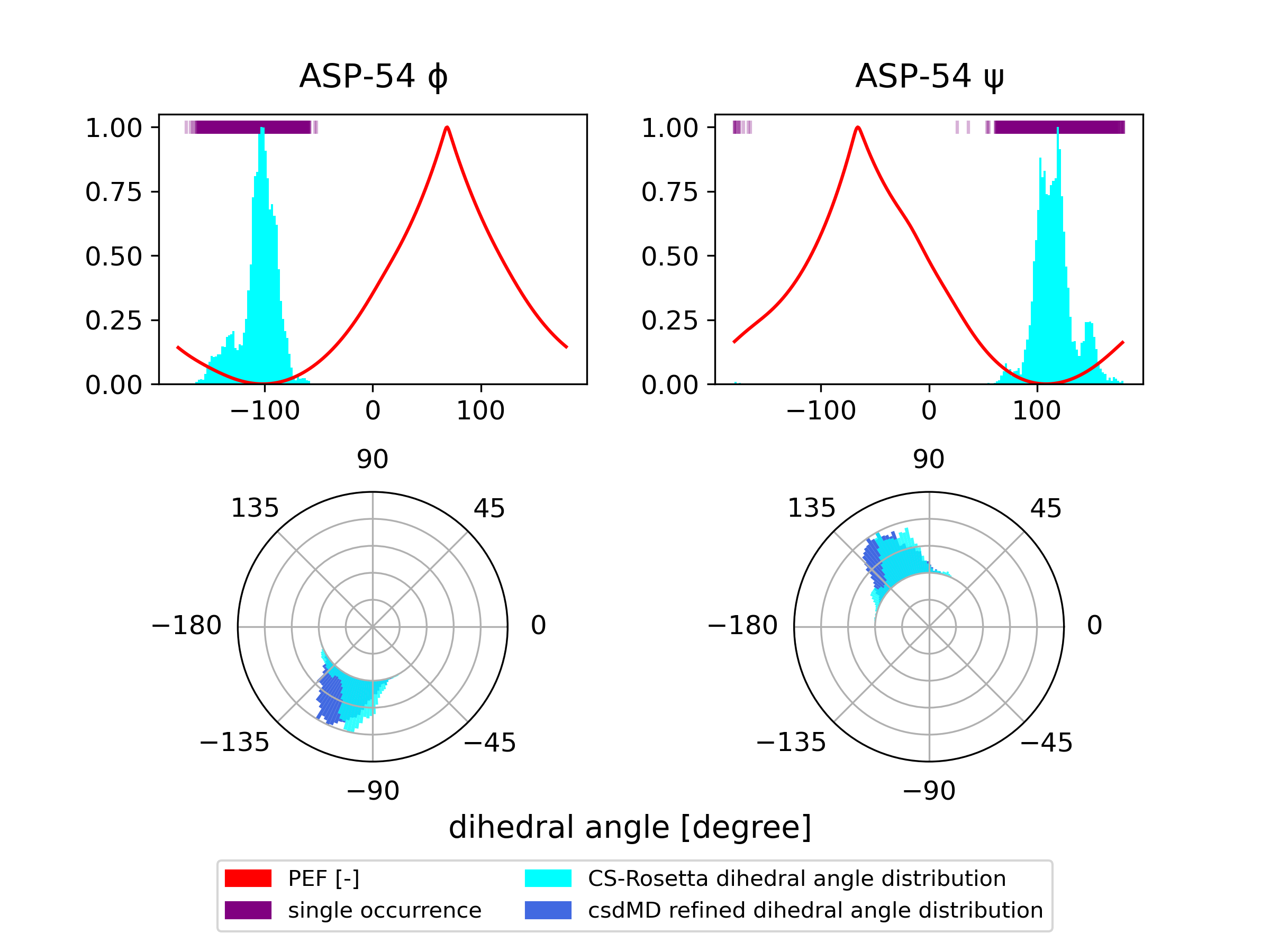

Supplement: Supplementary file 1 [file ijms-24-12101-s001.zip › KRAS-G12C-GDP-Mg-free_angle_figures/54-ASP.png]

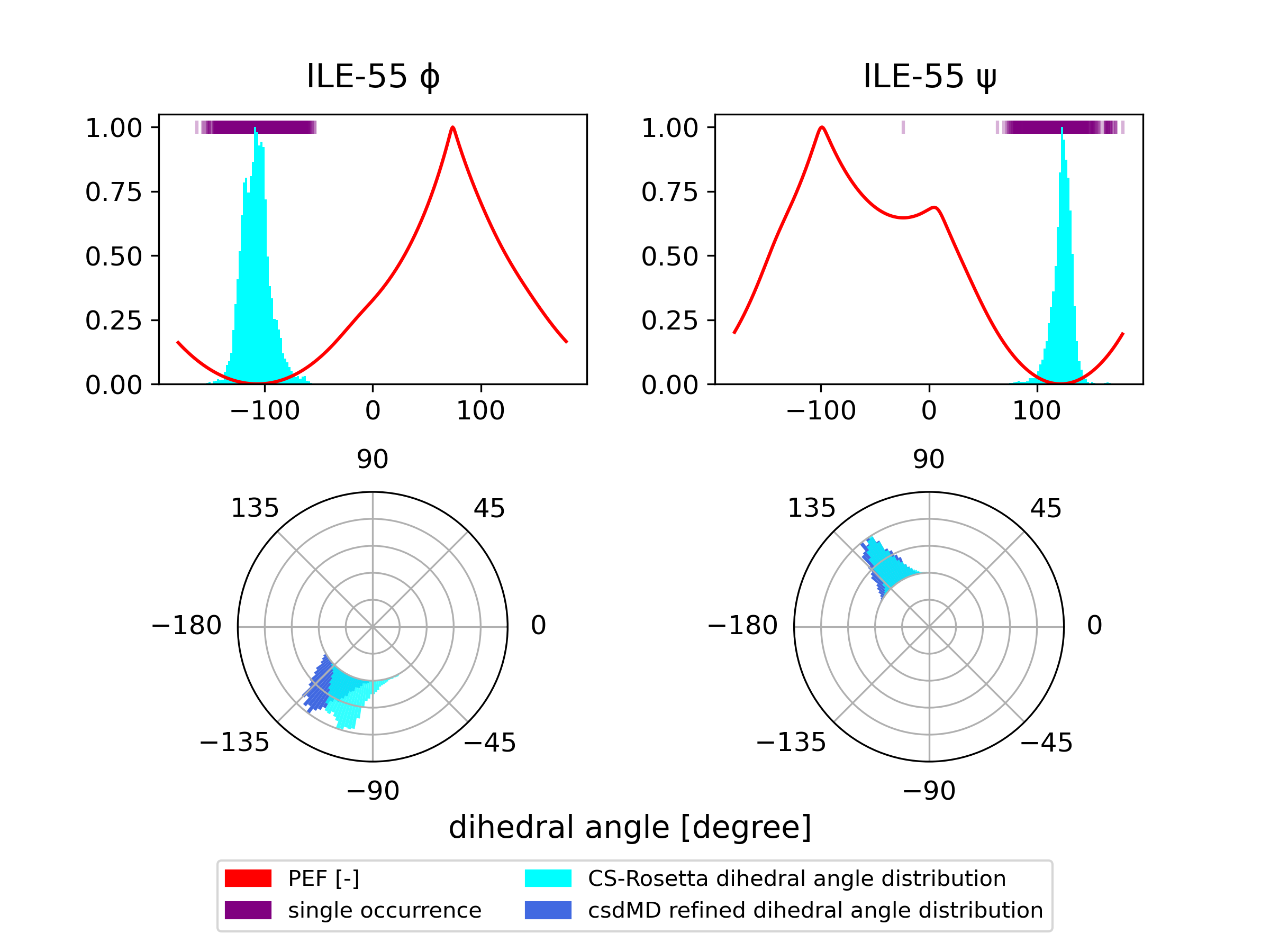

Supplement: Supplementary file 1 [file ijms-24-12101-s001.zip › KRAS-G12C-GDP-Mg-free_angle_figures/55-ILE.png]

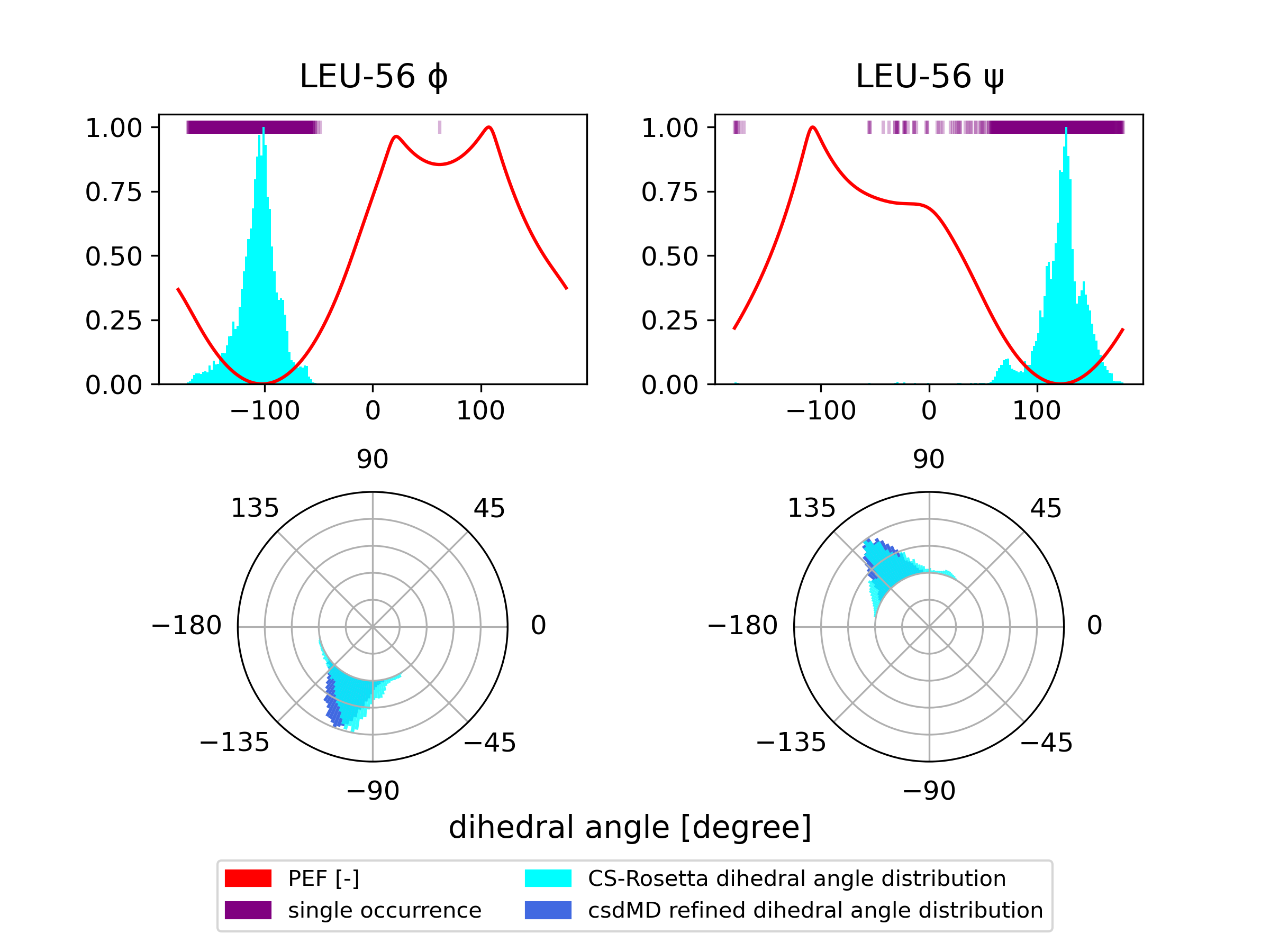

Supplement: Supplementary file 1 [file ijms-24-12101-s001.zip › KRAS-G12C-GDP-Mg-free_angle_figures/56-LEU.png]

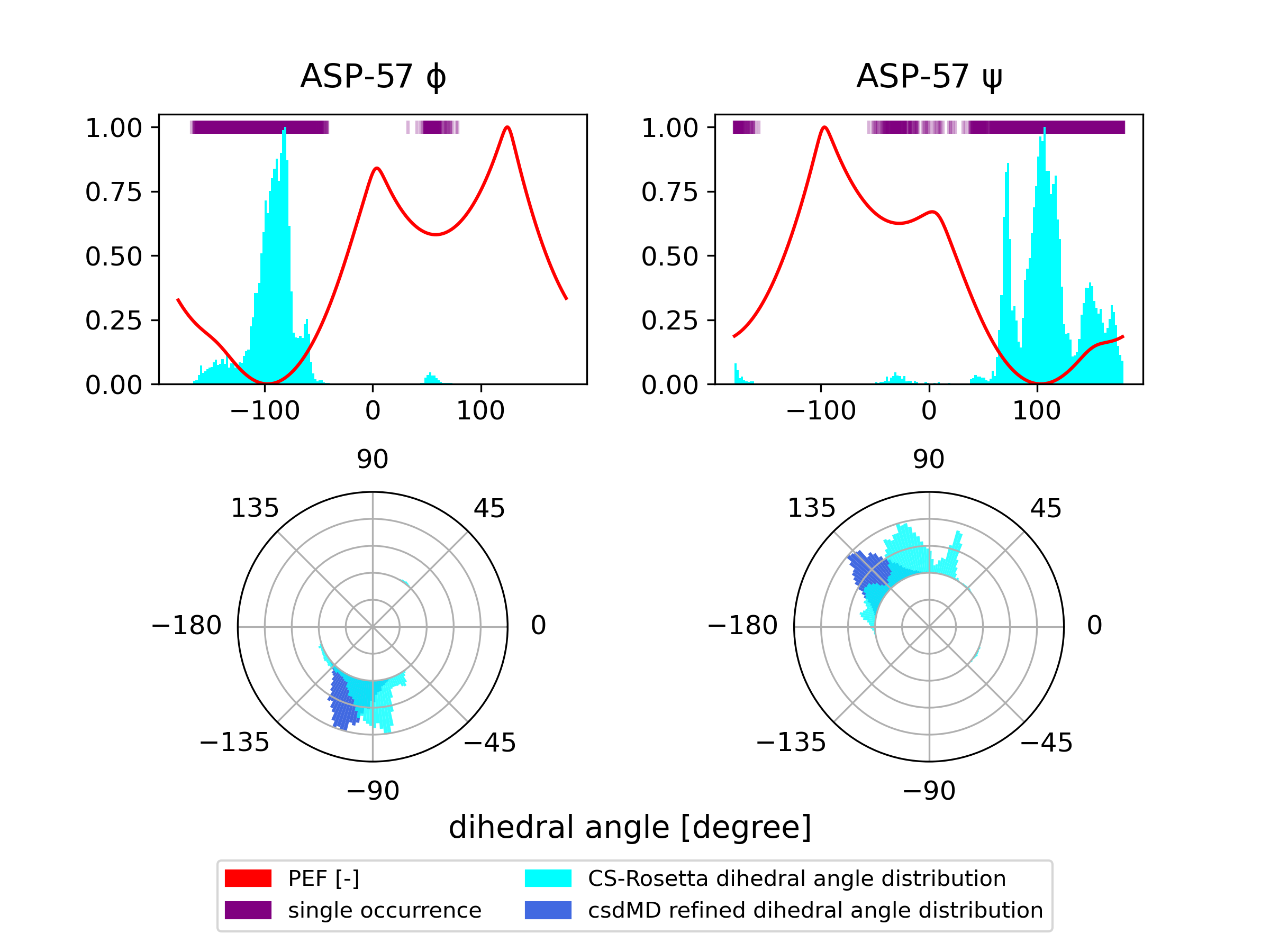

Supplement: Supplementary file 1 [file ijms-24-12101-s001.zip › KRAS-G12C-GDP-Mg-free_angle_figures/57-ASP.png]

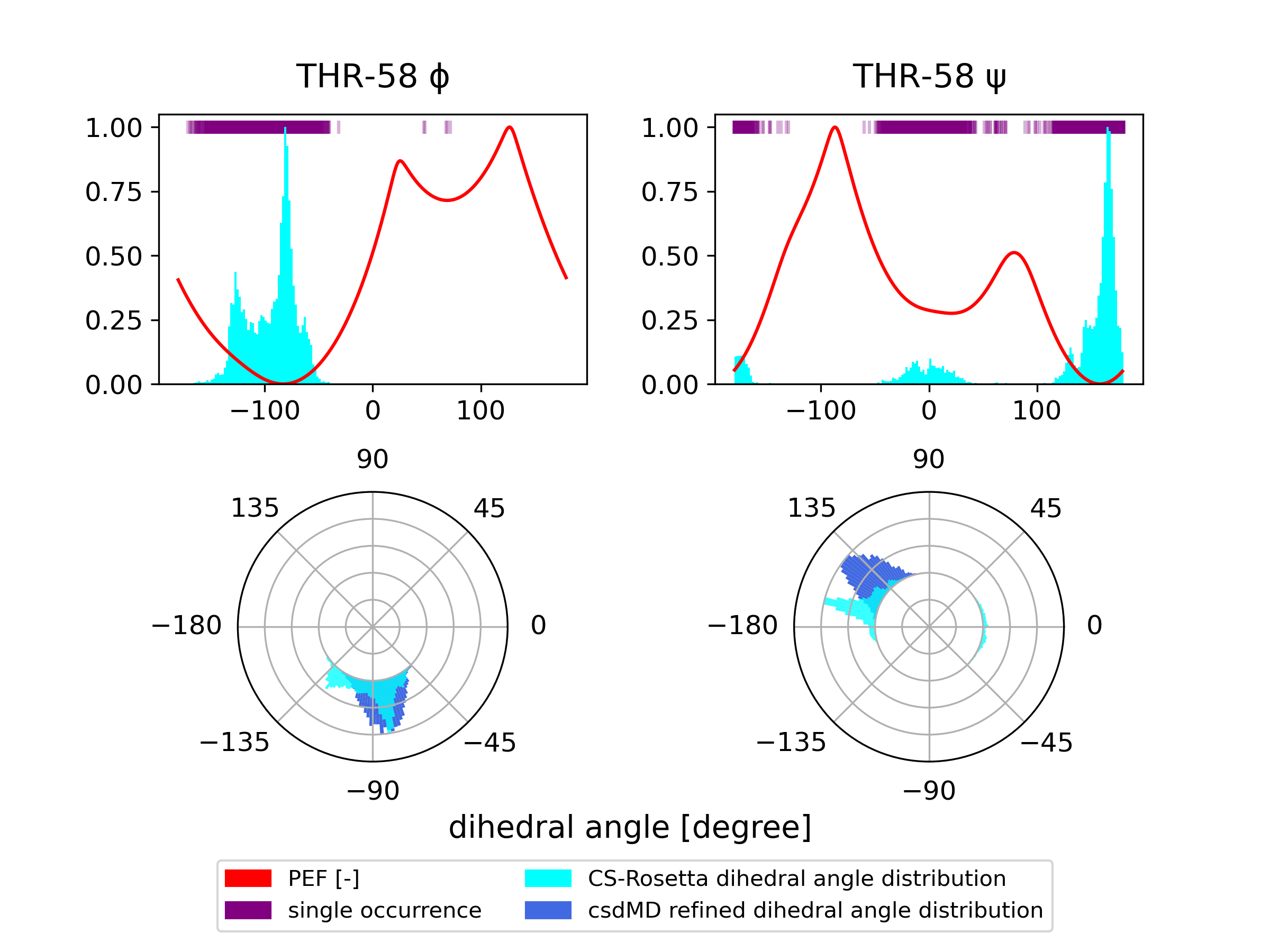

Supplement: Supplementary file 1 [file ijms-24-12101-s001.zip › KRAS-G12C-GDP-Mg-free_angle_figures/58-THR.png]

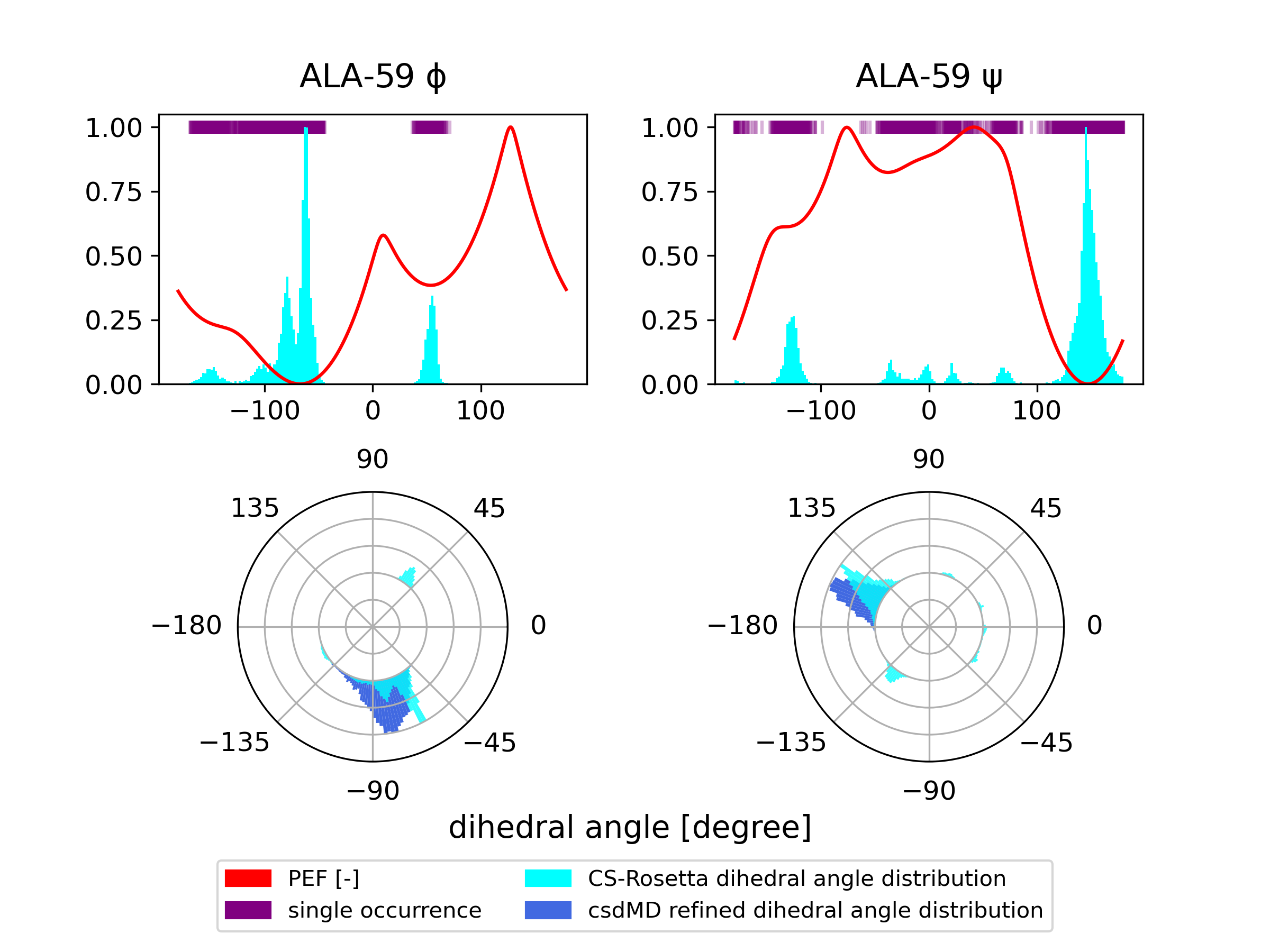

Supplement: Supplementary file 1 [file ijms-24-12101-s001.zip › KRAS-G12C-GDP-Mg-free_angle_figures/59-ALA.png]

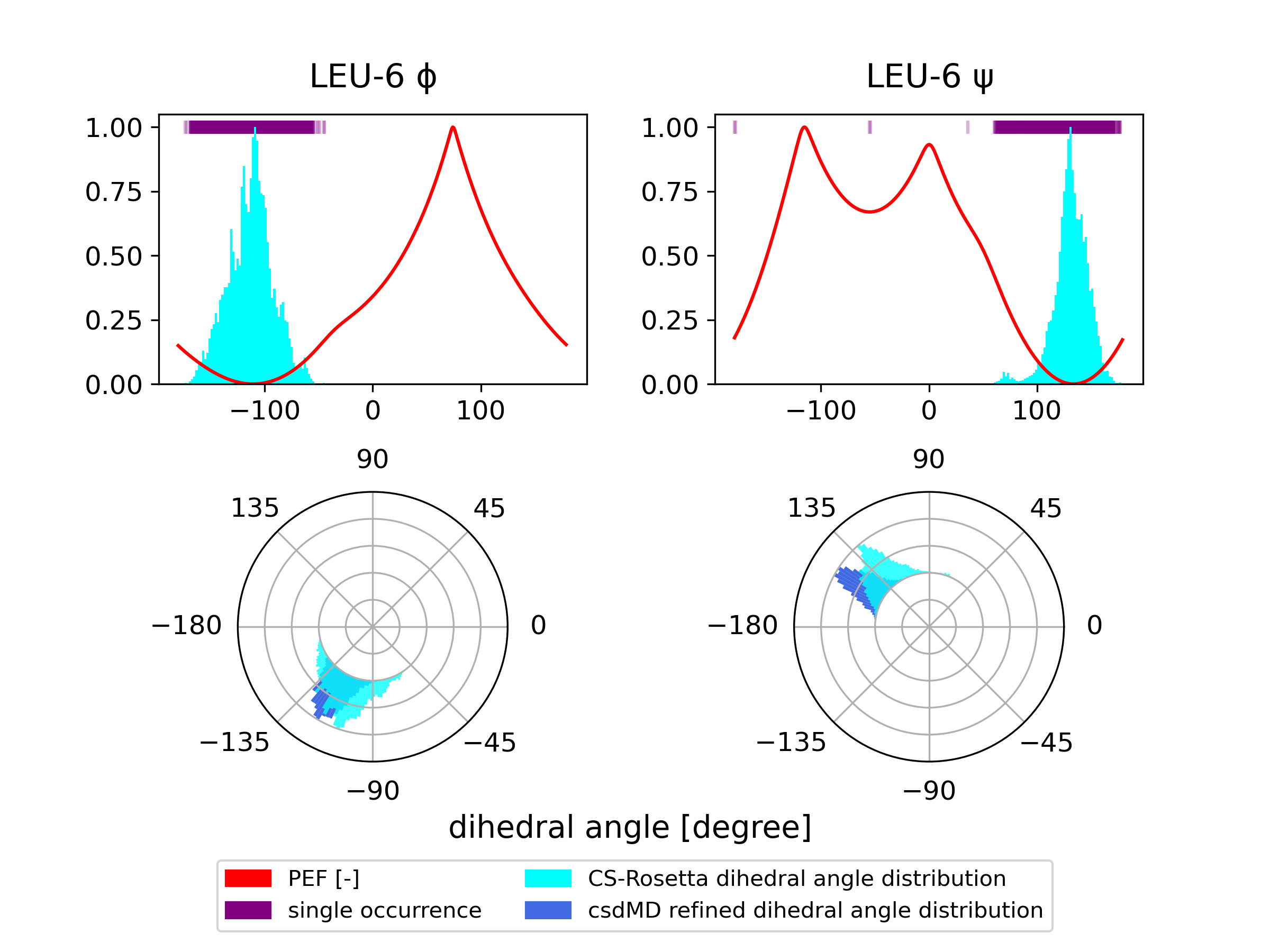

Supplement: Supplementary file 1 [file ijms-24-12101-s001.zip › KRAS-G12C-GDP-Mg-free_angle_figures/6-LEU.png]

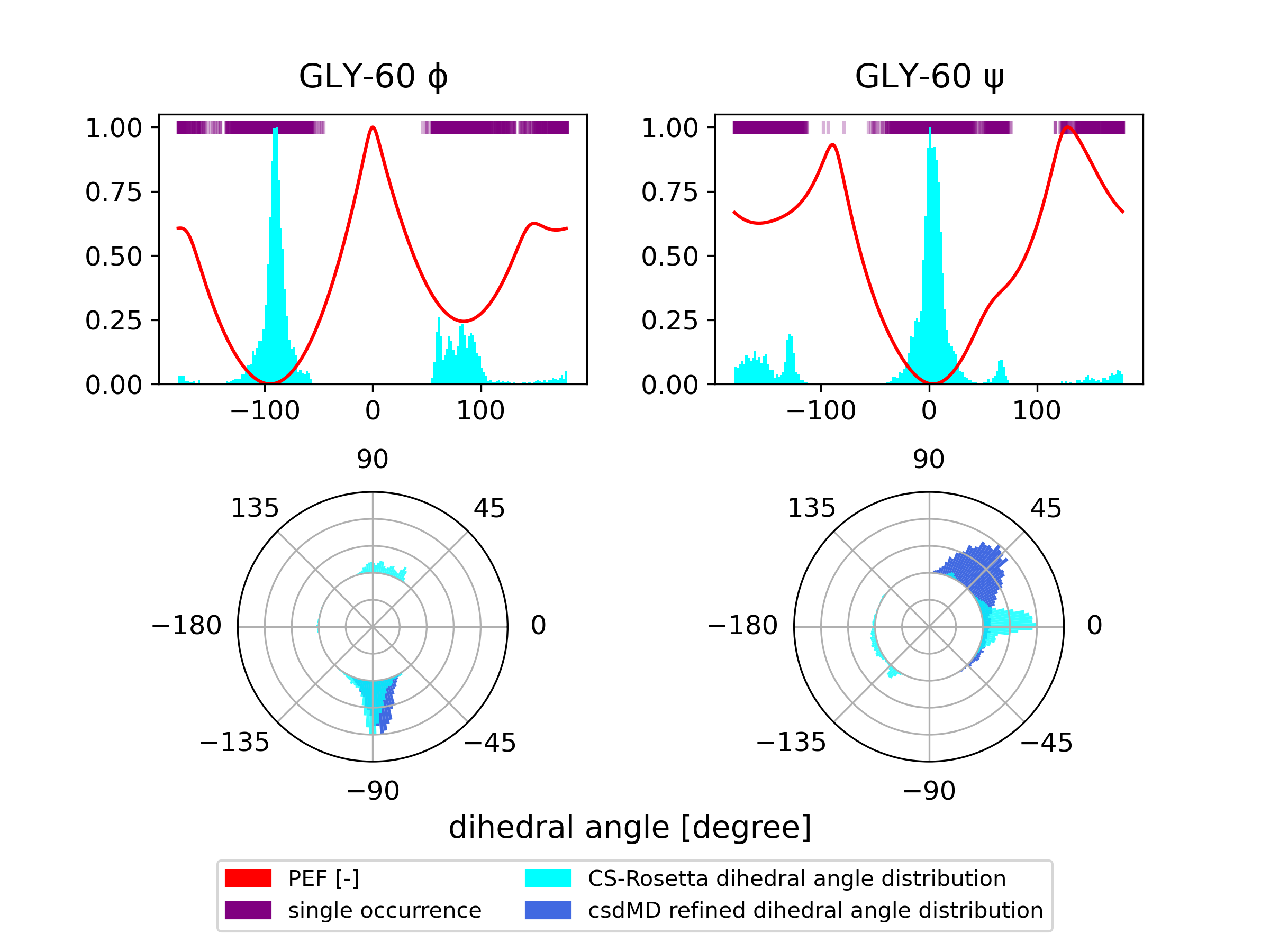

Supplement: Supplementary file 1 [file ijms-24-12101-s001.zip › KRAS-G12C-GDP-Mg-free_angle_figures/60-GLY.png]

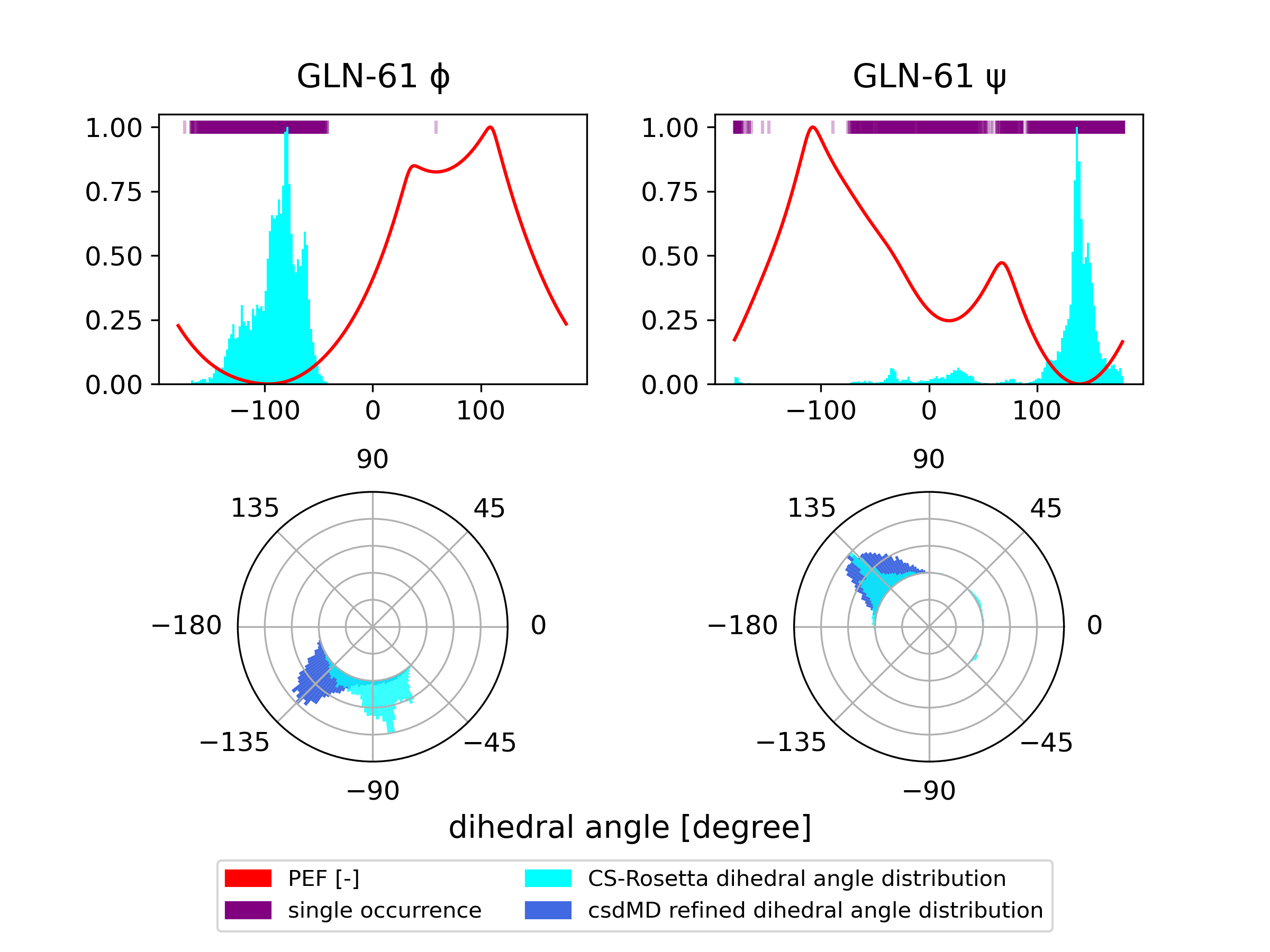

Supplement: Supplementary file 1 [file ijms-24-12101-s001.zip › KRAS-G12C-GDP-Mg-free_angle_figures/61-GLN.png]

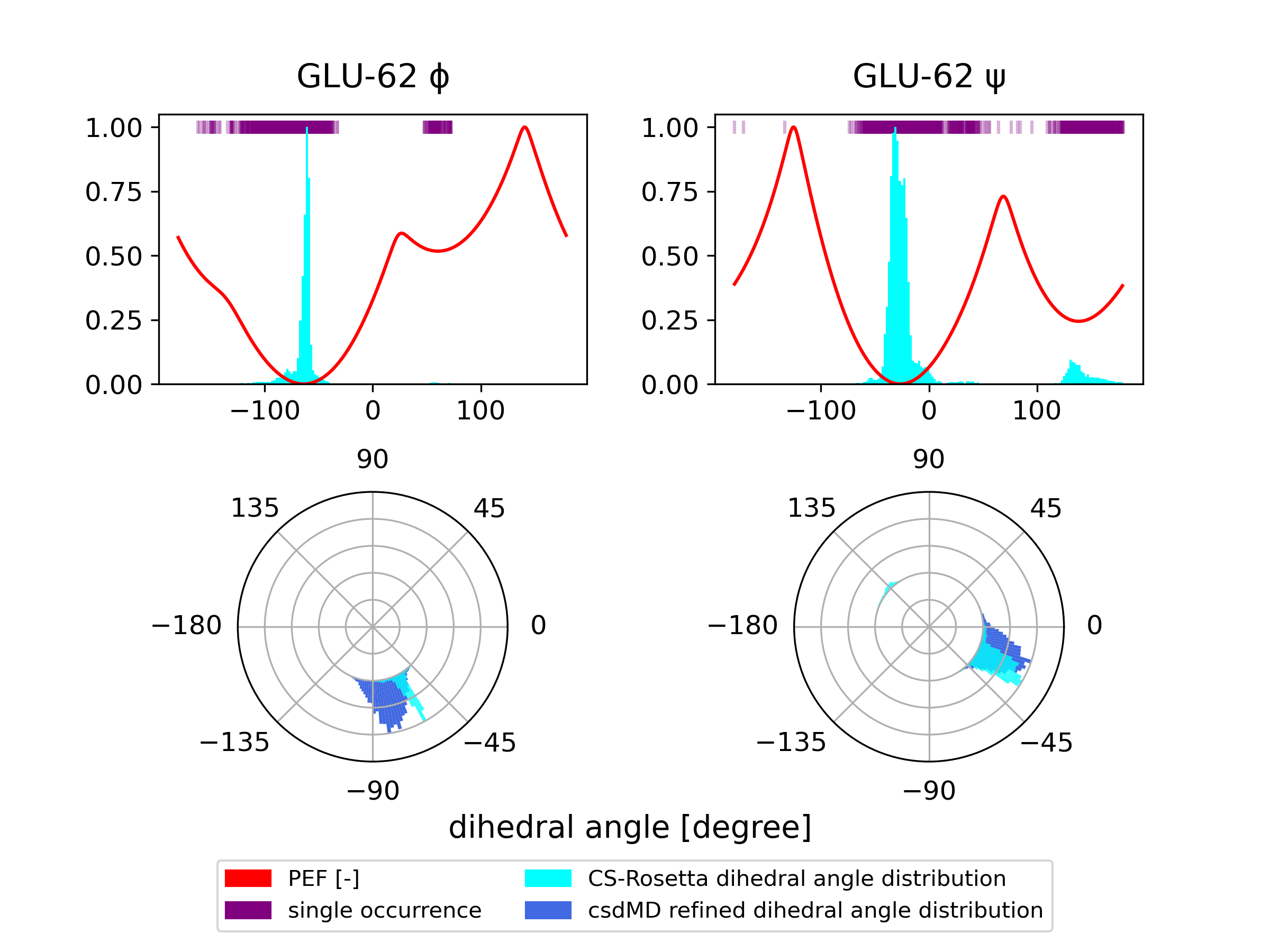

Supplement: Supplementary file 1 [file ijms-24-12101-s001.zip › KRAS-G12C-GDP-Mg-free_angle_figures/62-GLU.png]

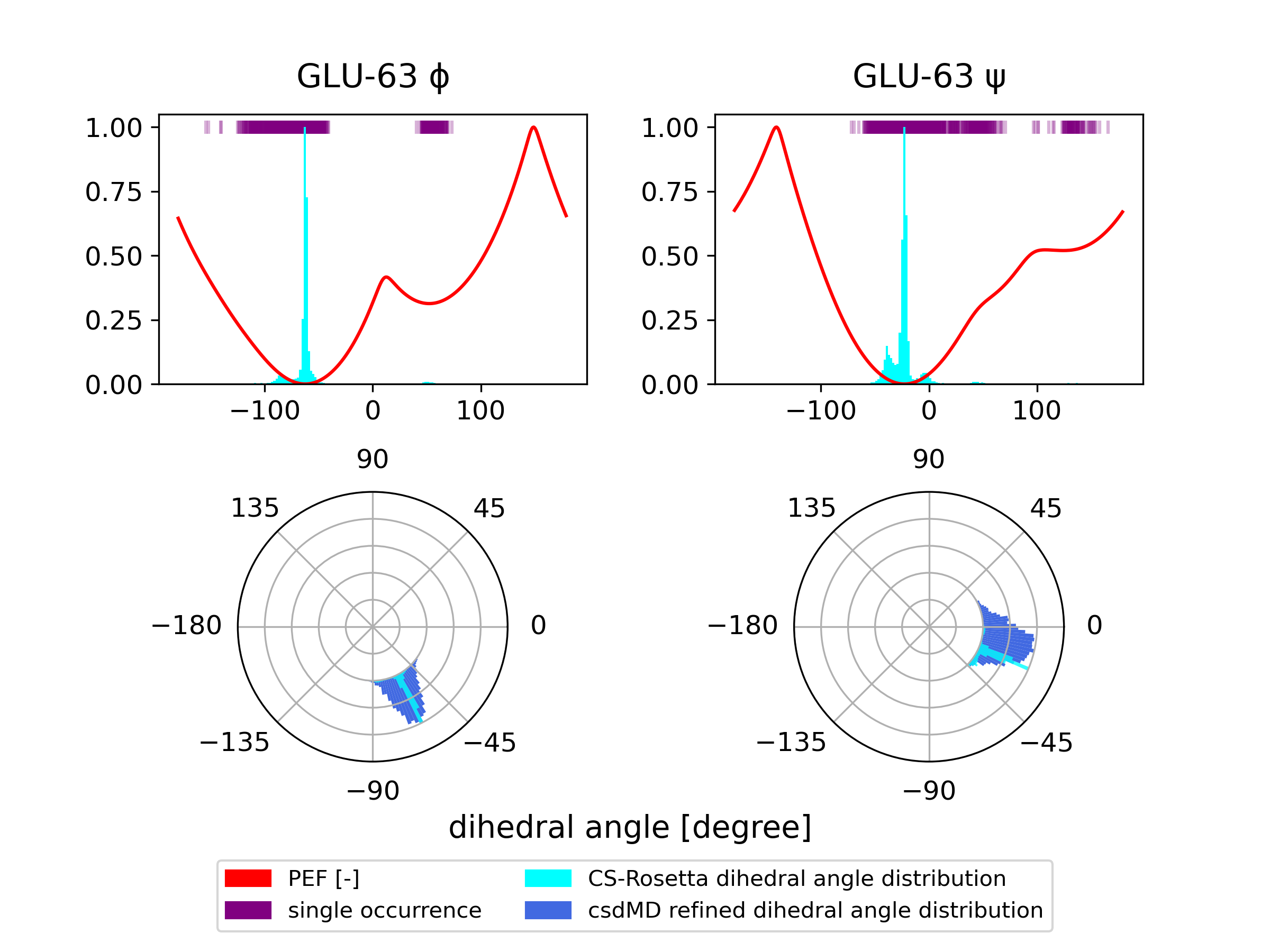

Supplement: Supplementary file 1 [file ijms-24-12101-s001.zip › KRAS-G12C-GDP-Mg-free_angle_figures/63-GLU.png]

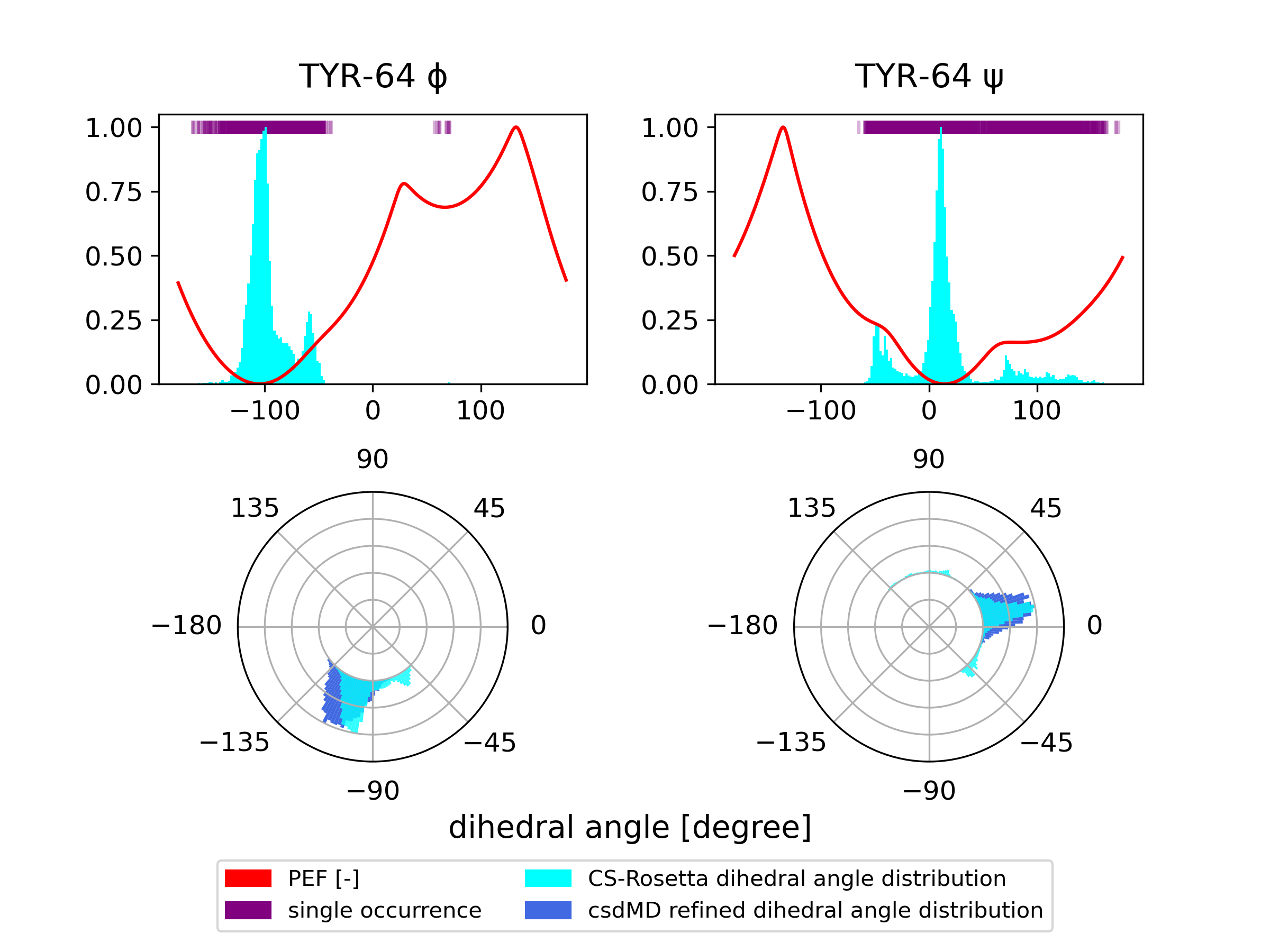

Supplement: Supplementary file 1 [file ijms-24-12101-s001.zip › KRAS-G12C-GDP-Mg-free_angle_figures/64-TYR.png]

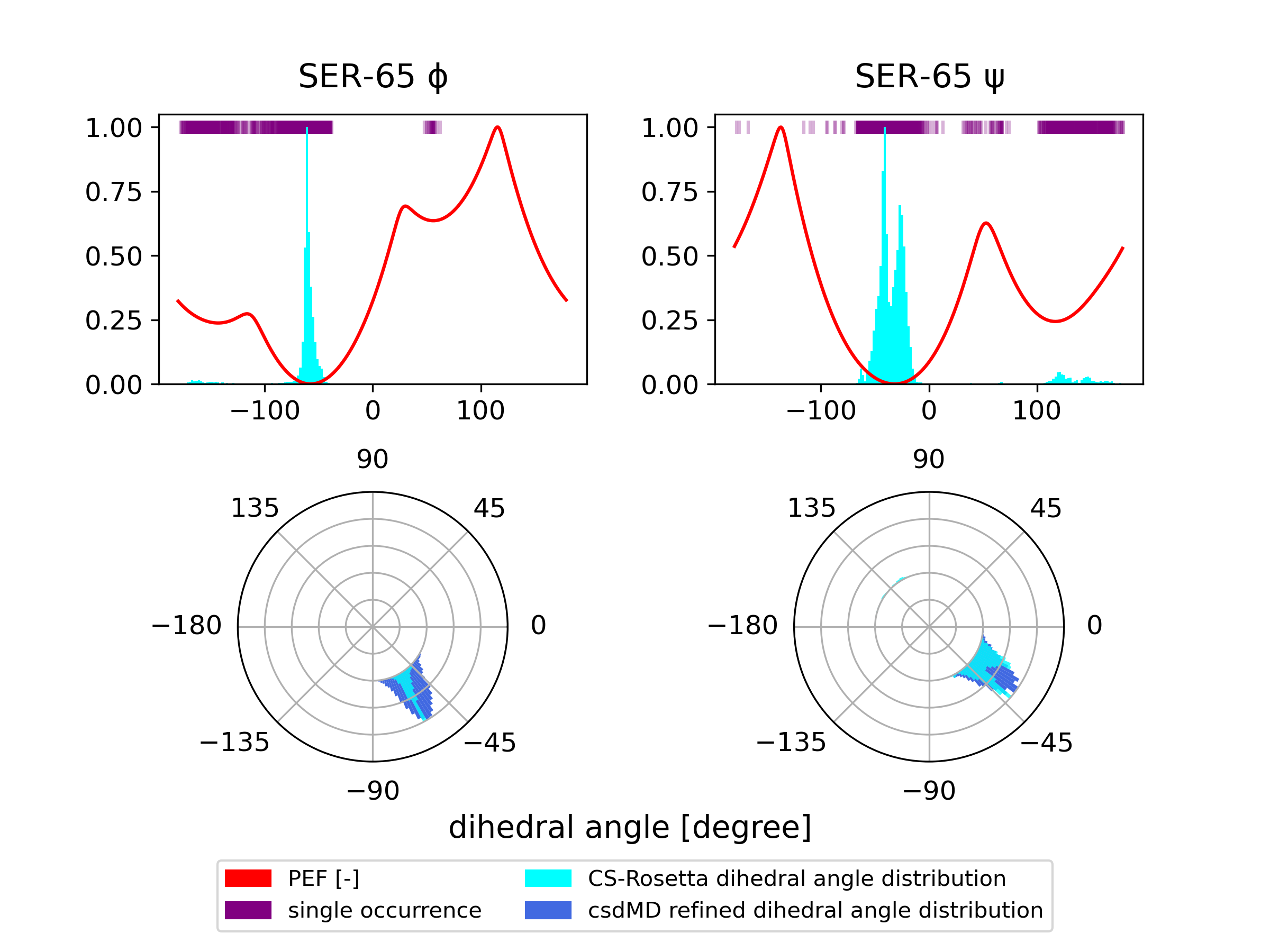

Supplement: Supplementary file 1 [file ijms-24-12101-s001.zip › KRAS-G12C-GDP-Mg-free_angle_figures/65-SER.png]

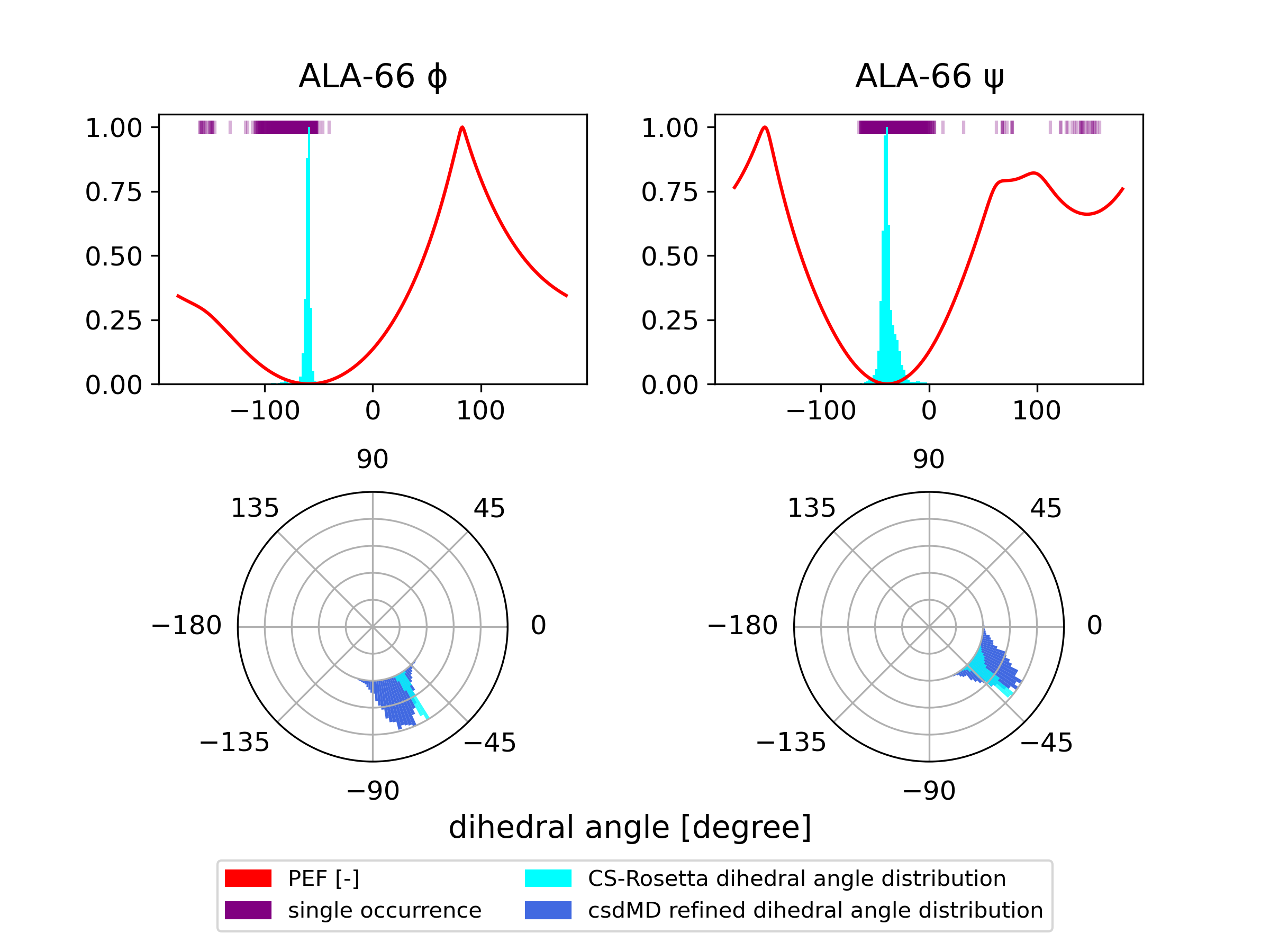

Supplement: Supplementary file 1 [file ijms-24-12101-s001.zip › KRAS-G12C-GDP-Mg-free_angle_figures/66-ALA.png]

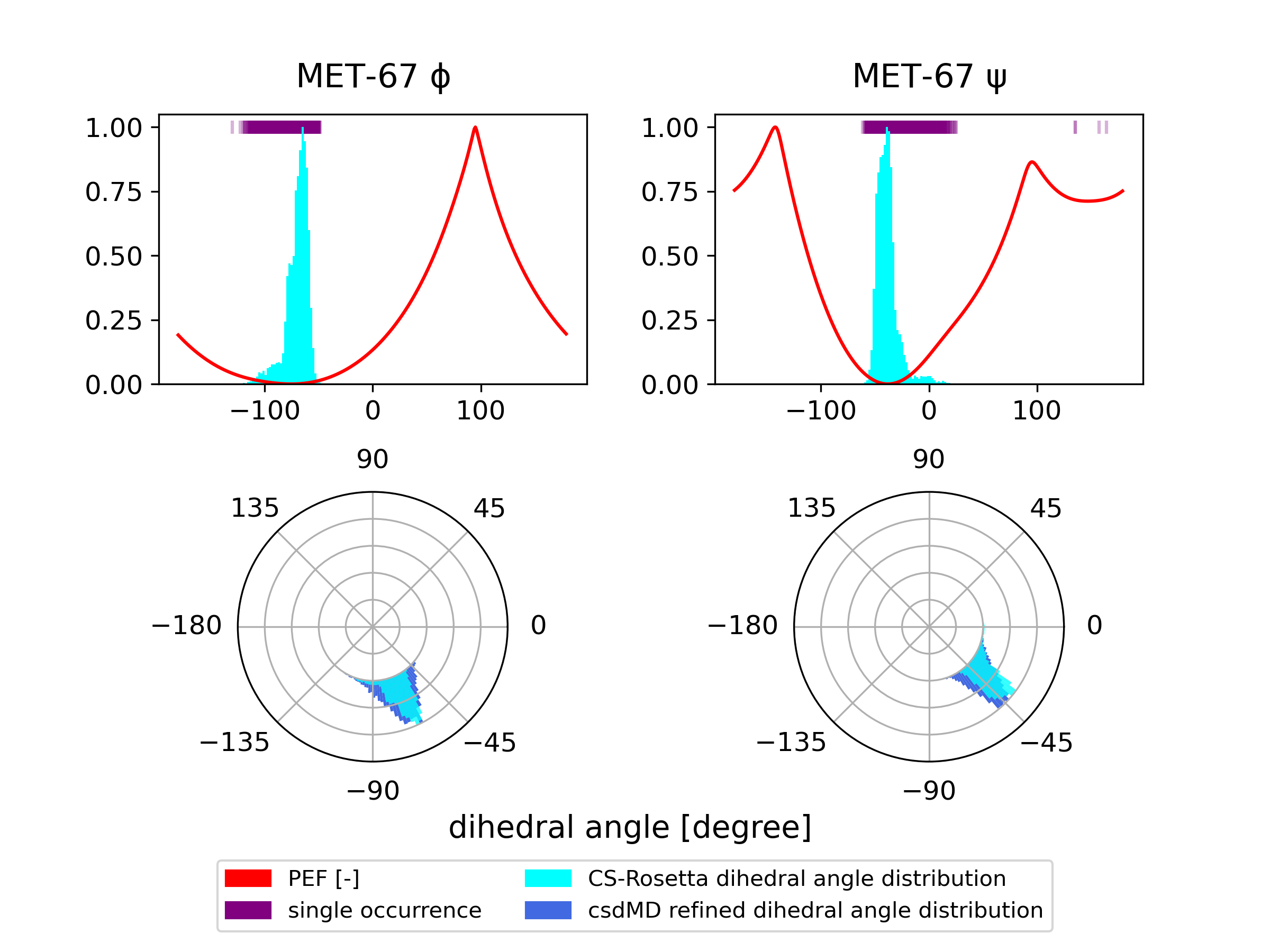

Supplement: Supplementary file 1 [file ijms-24-12101-s001.zip › KRAS-G12C-GDP-Mg-free_angle_figures/67-MET.png]

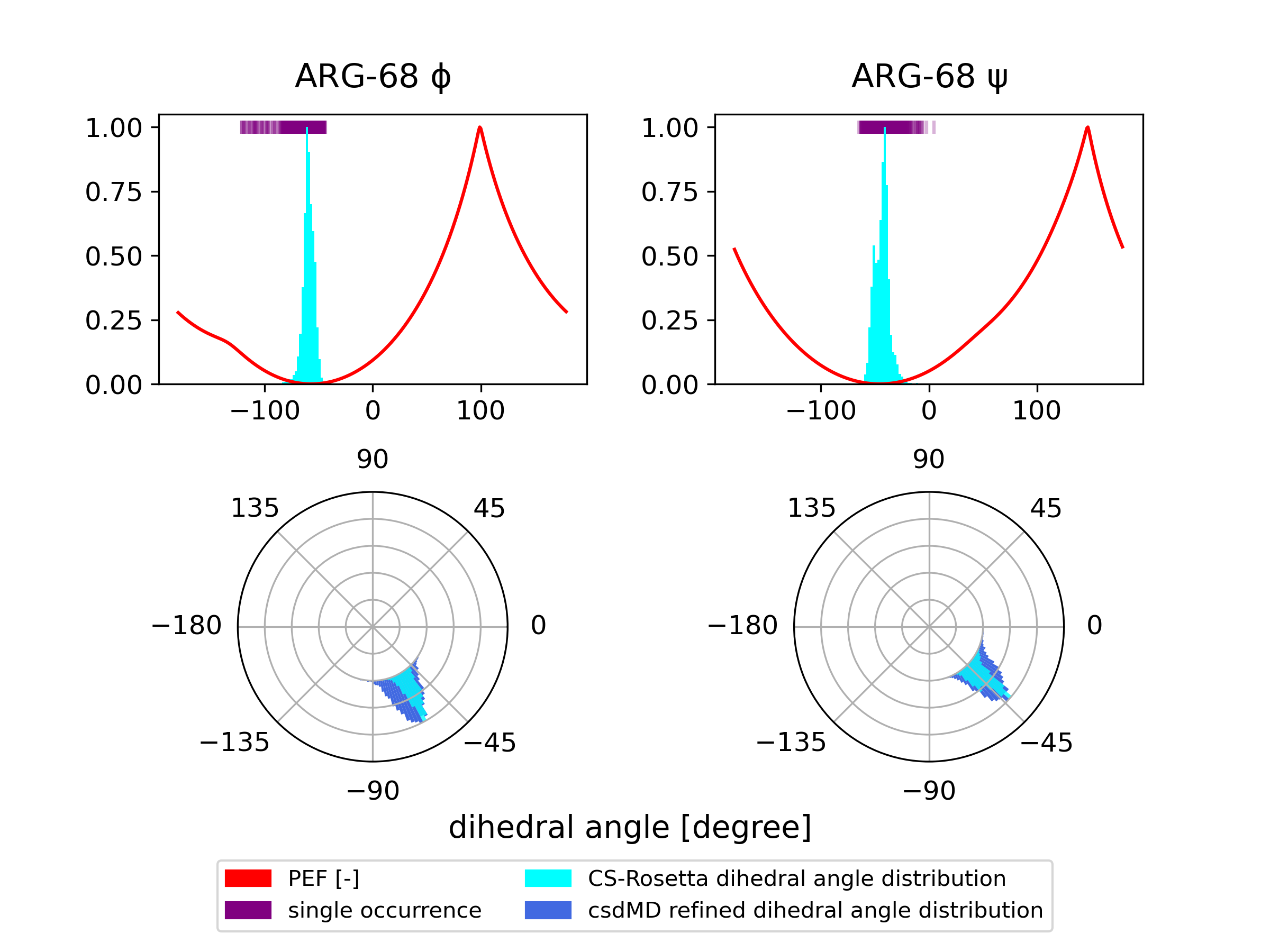

Supplement: Supplementary file 1 [file ijms-24-12101-s001.zip › KRAS-G12C-GDP-Mg-free_angle_figures/68-ARG.png]

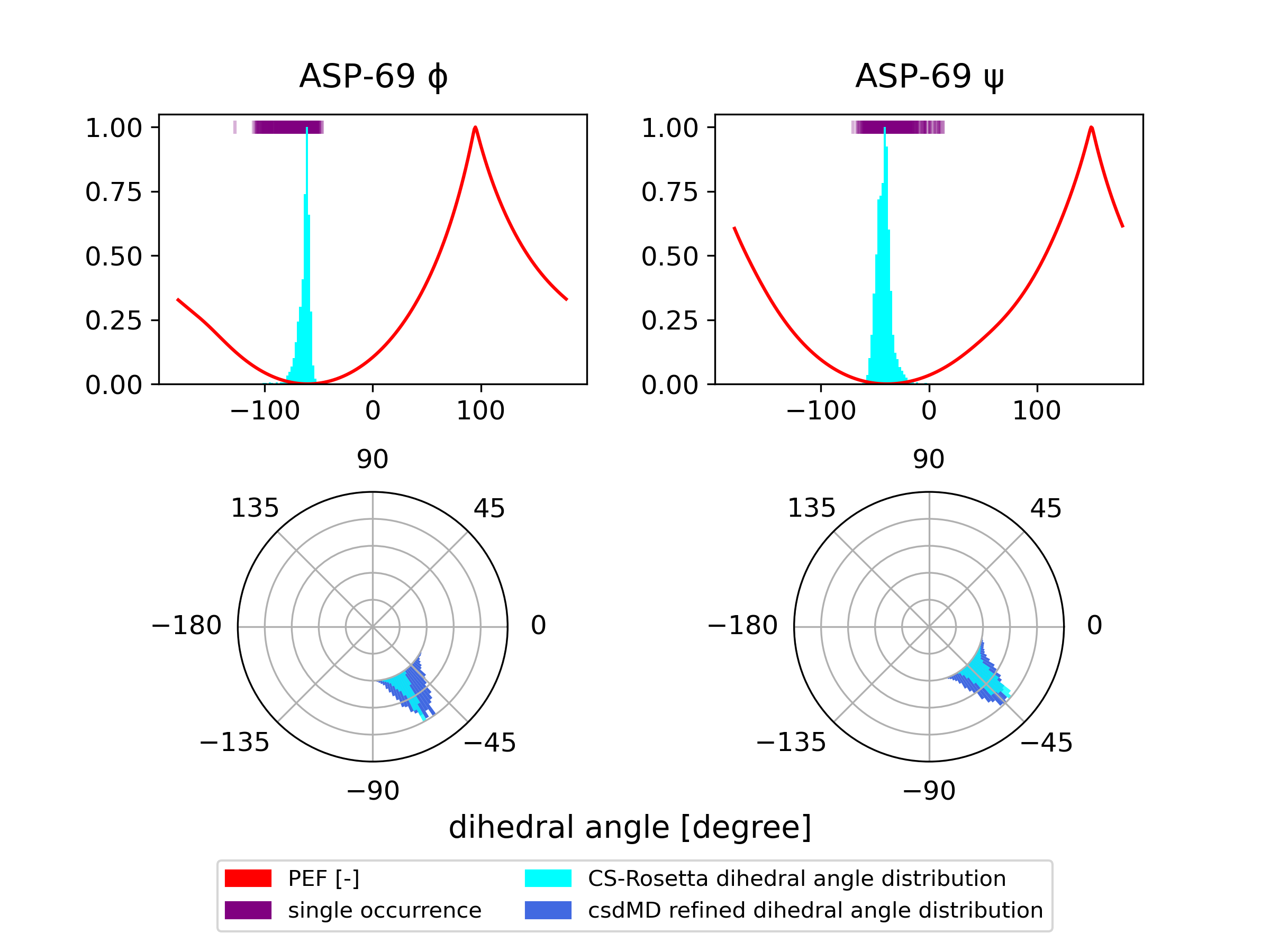

Supplement: Supplementary file 1 [file ijms-24-12101-s001.zip › KRAS-G12C-GDP-Mg-free_angle_figures/69-ASP.png]

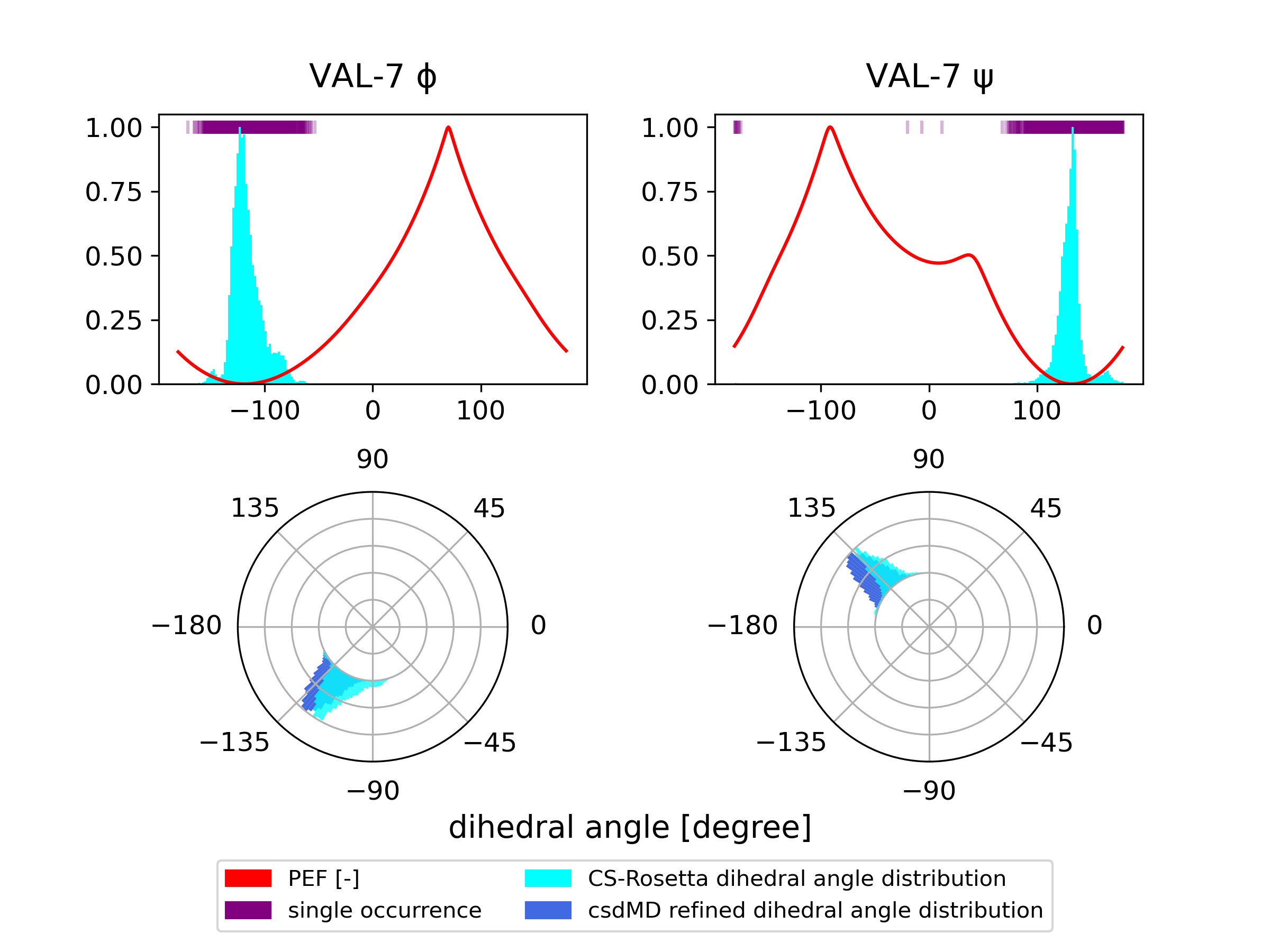

Supplement: Supplementary file 1 [file ijms-24-12101-s001.zip › KRAS-G12C-GDP-Mg-free_angle_figures/7-VAL.png]

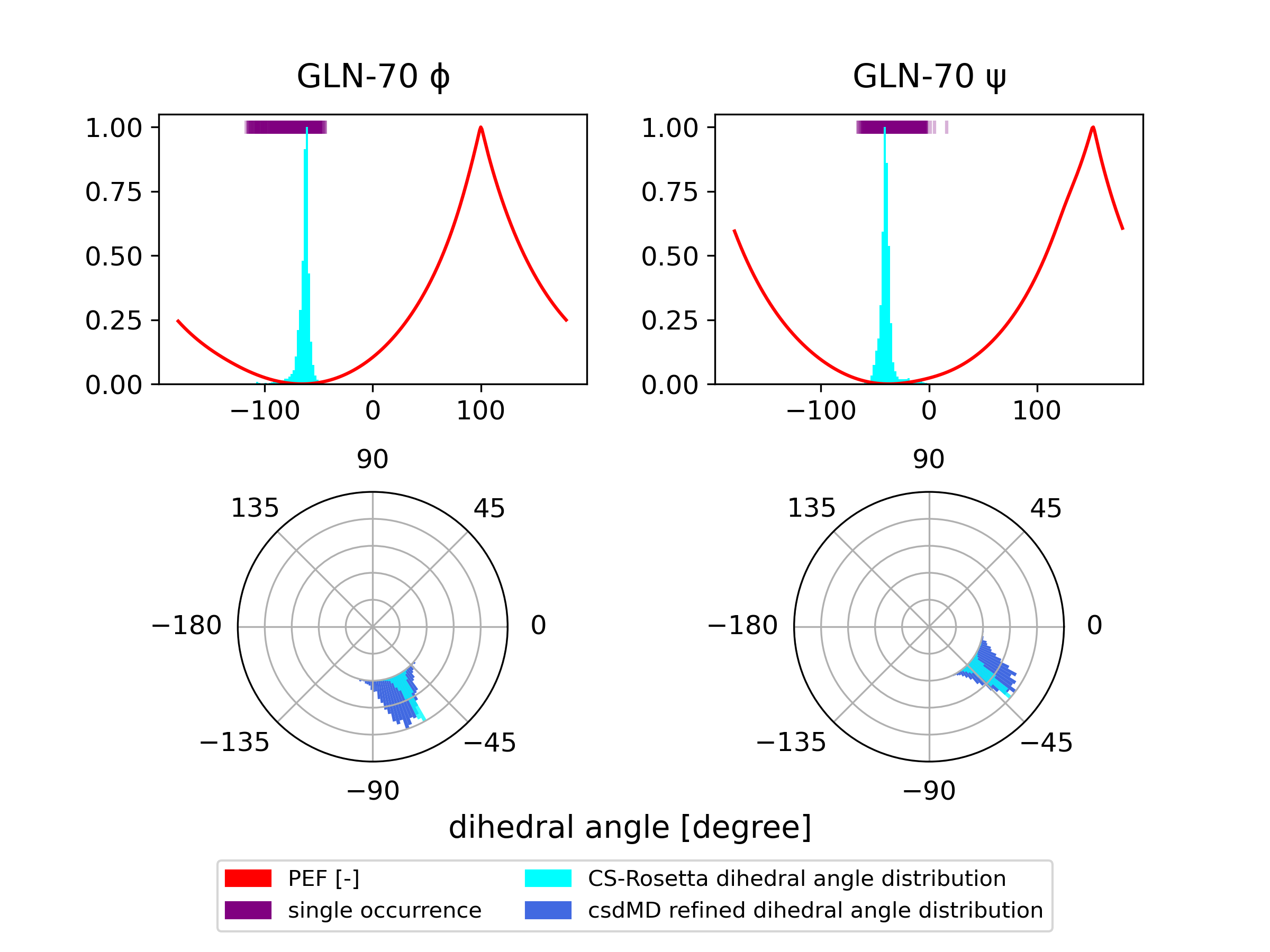

Supplement: Supplementary file 1 [file ijms-24-12101-s001.zip › KRAS-G12C-GDP-Mg-free_angle_figures/70-GLN.png]

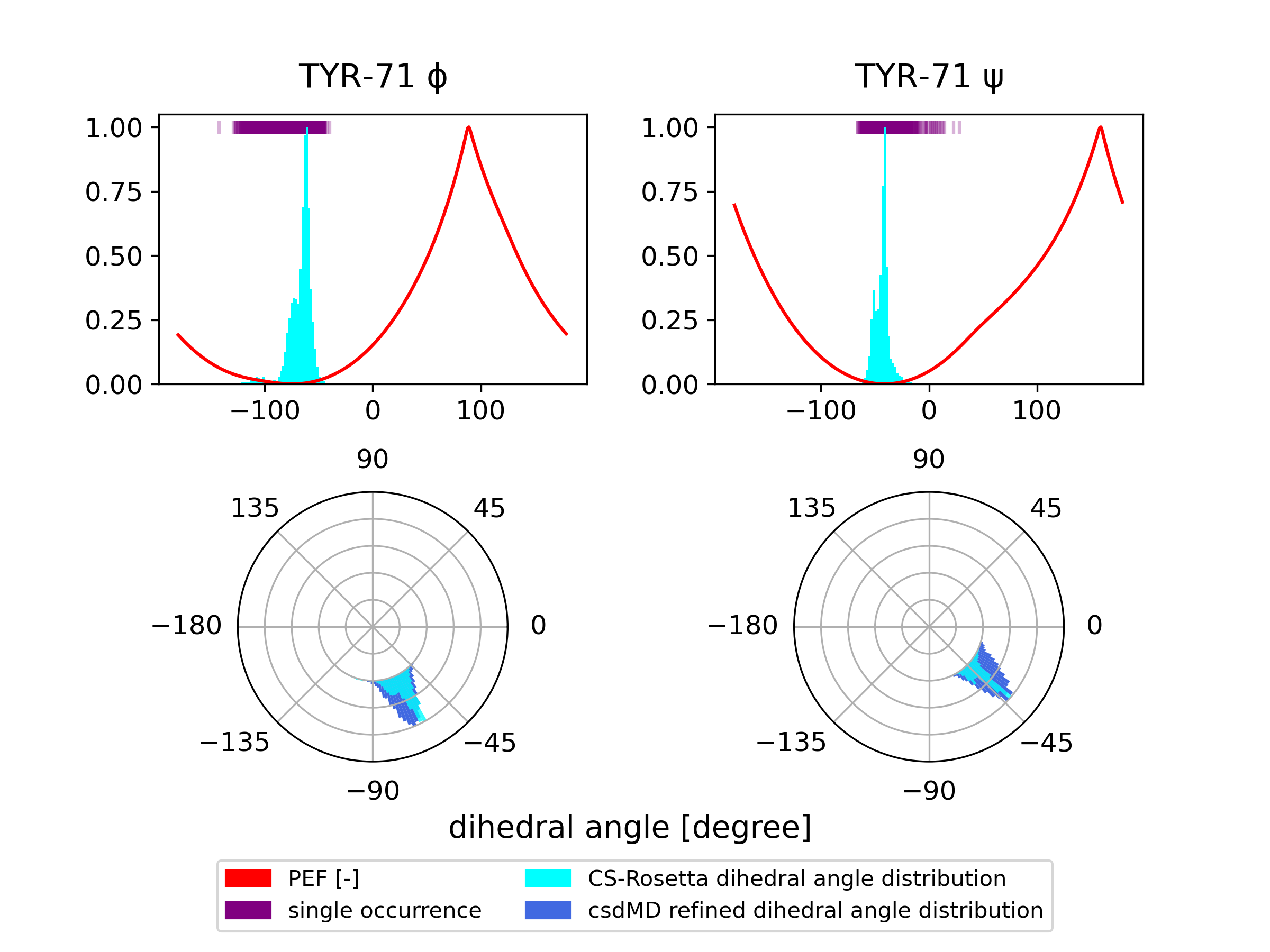

Supplement: Supplementary file 1 [file ijms-24-12101-s001.zip › KRAS-G12C-GDP-Mg-free_angle_figures/71-TYR.png]

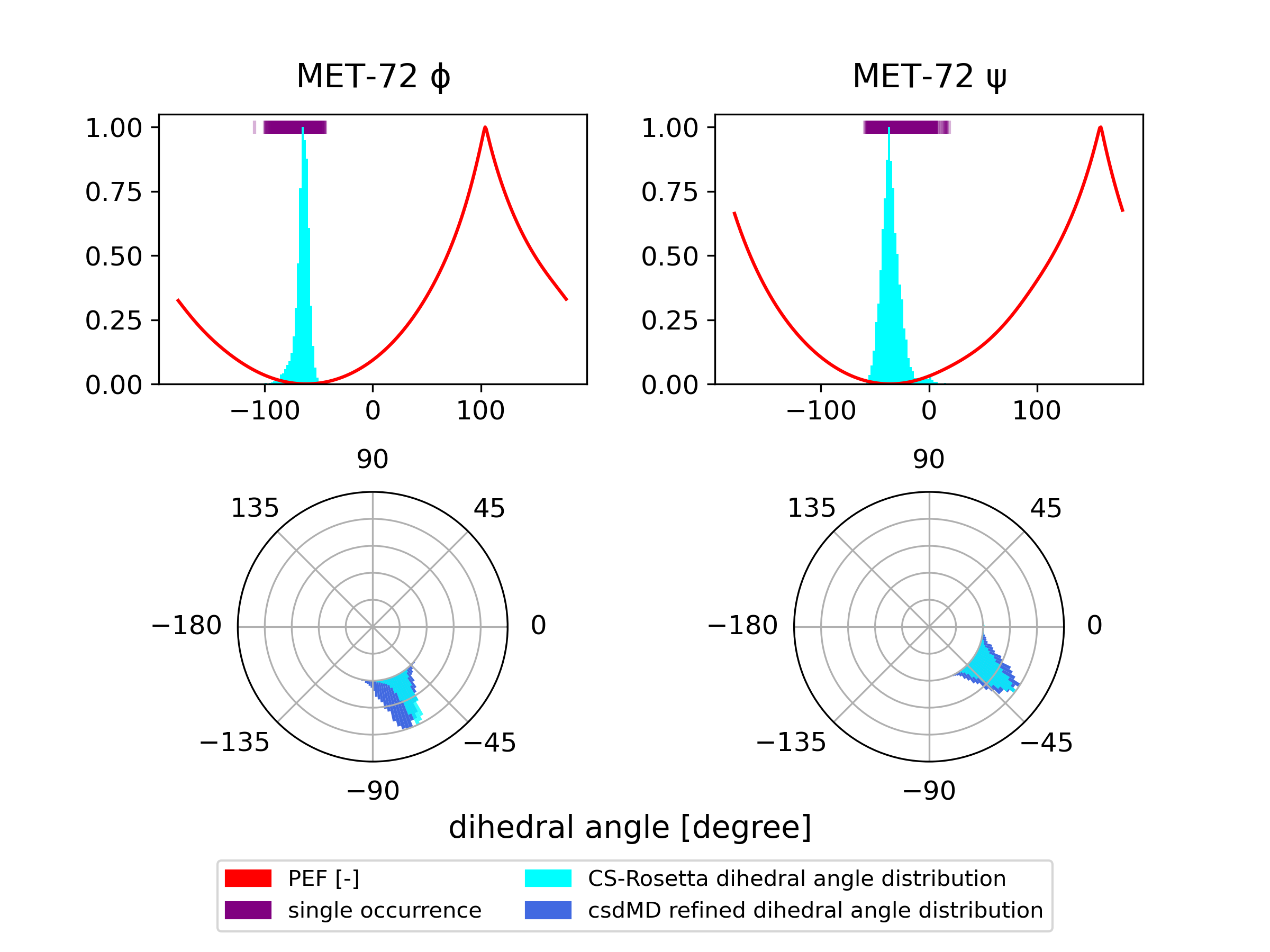

Supplement: Supplementary file 1 [file ijms-24-12101-s001.zip › KRAS-G12C-GDP-Mg-free_angle_figures/72-MET.png]

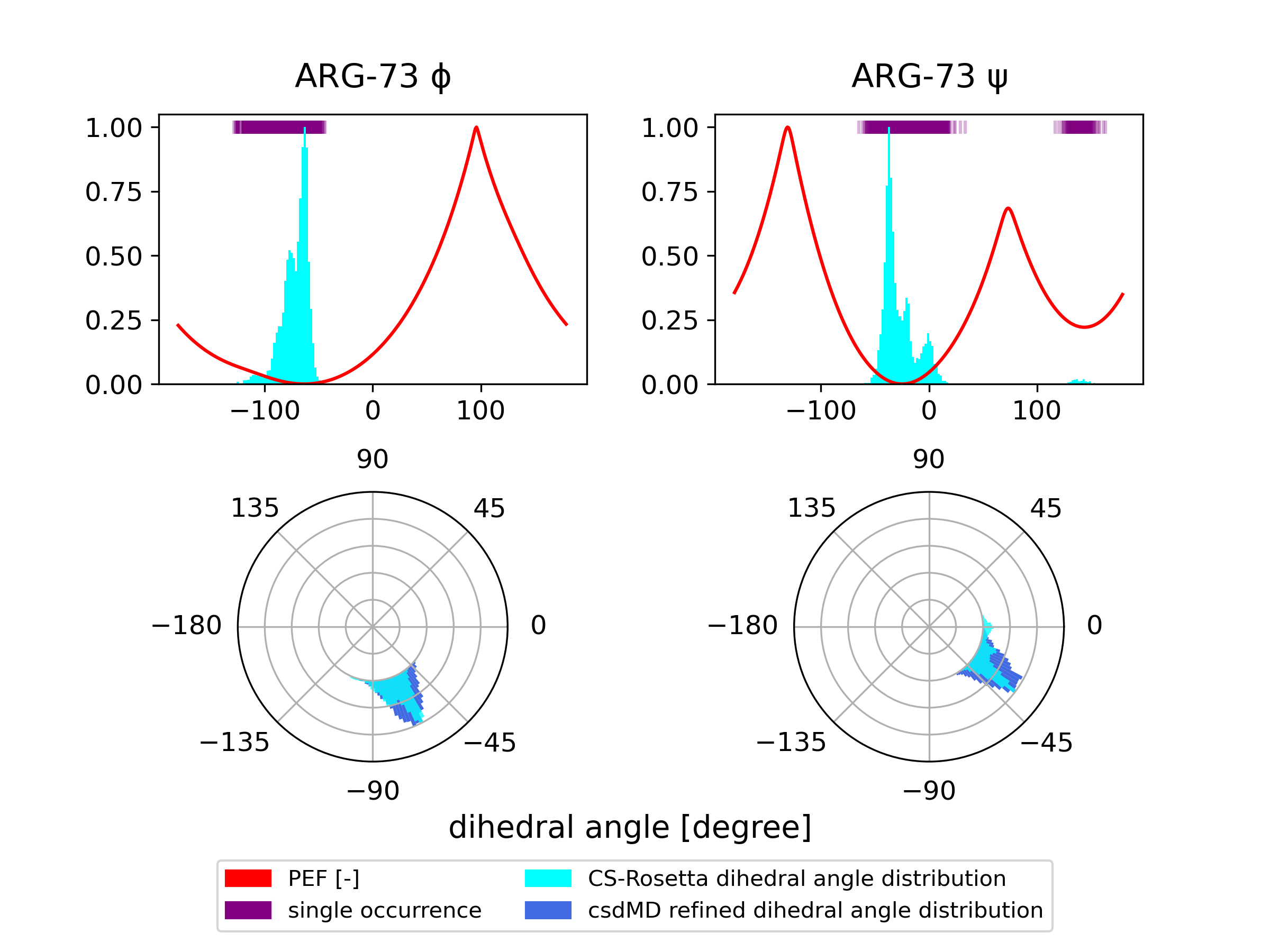

Supplement: Supplementary file 1 [file ijms-24-12101-s001.zip › KRAS-G12C-GDP-Mg-free_angle_figures/73-ARG.png]

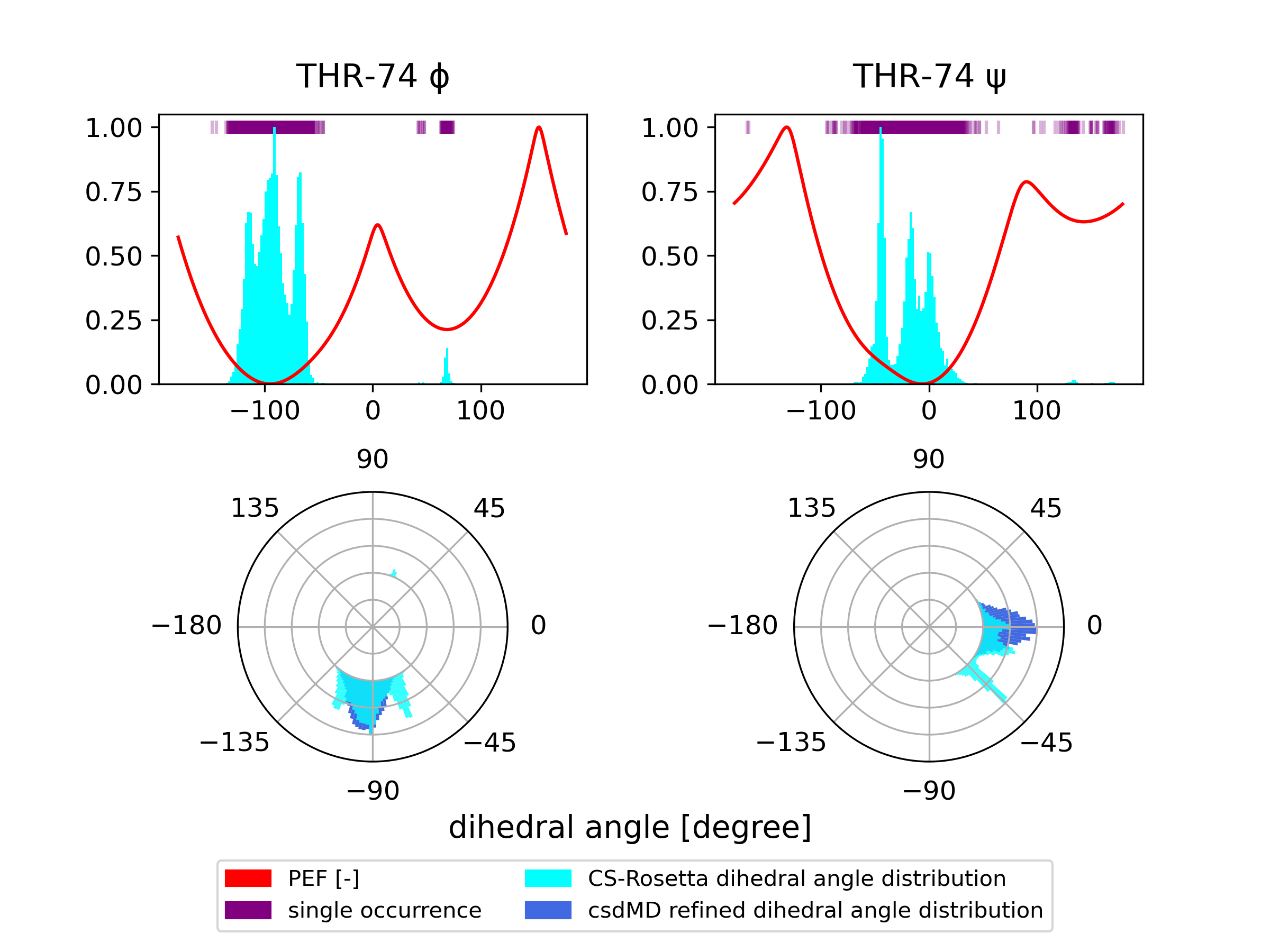

Supplement: Supplementary file 1 [file ijms-24-12101-s001.zip › KRAS-G12C-GDP-Mg-free_angle_figures/74-THR.png]

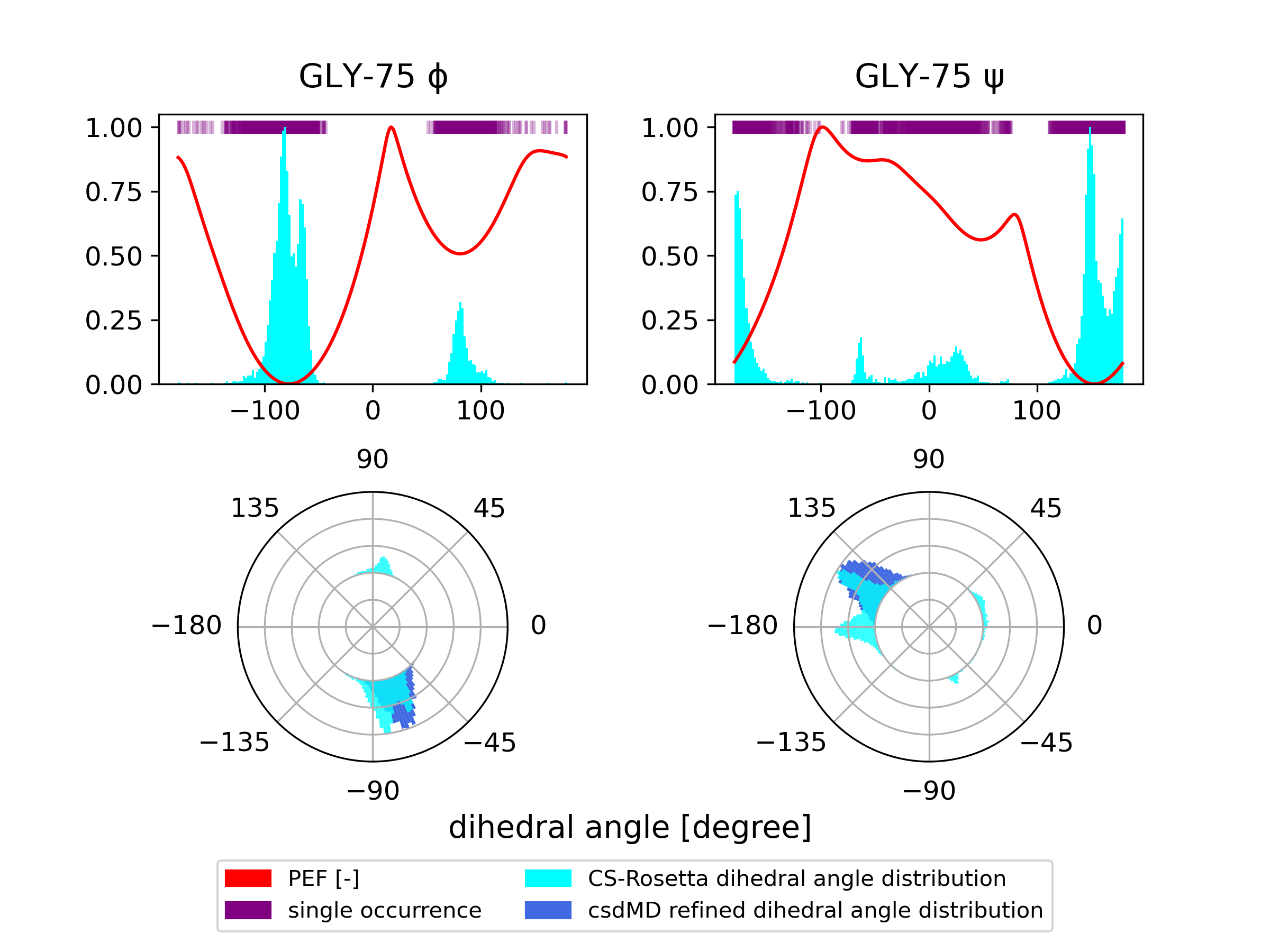

Supplement: Supplementary file 1 [file ijms-24-12101-s001.zip › KRAS-G12C-GDP-Mg-free_angle_figures/75-GLY.png]

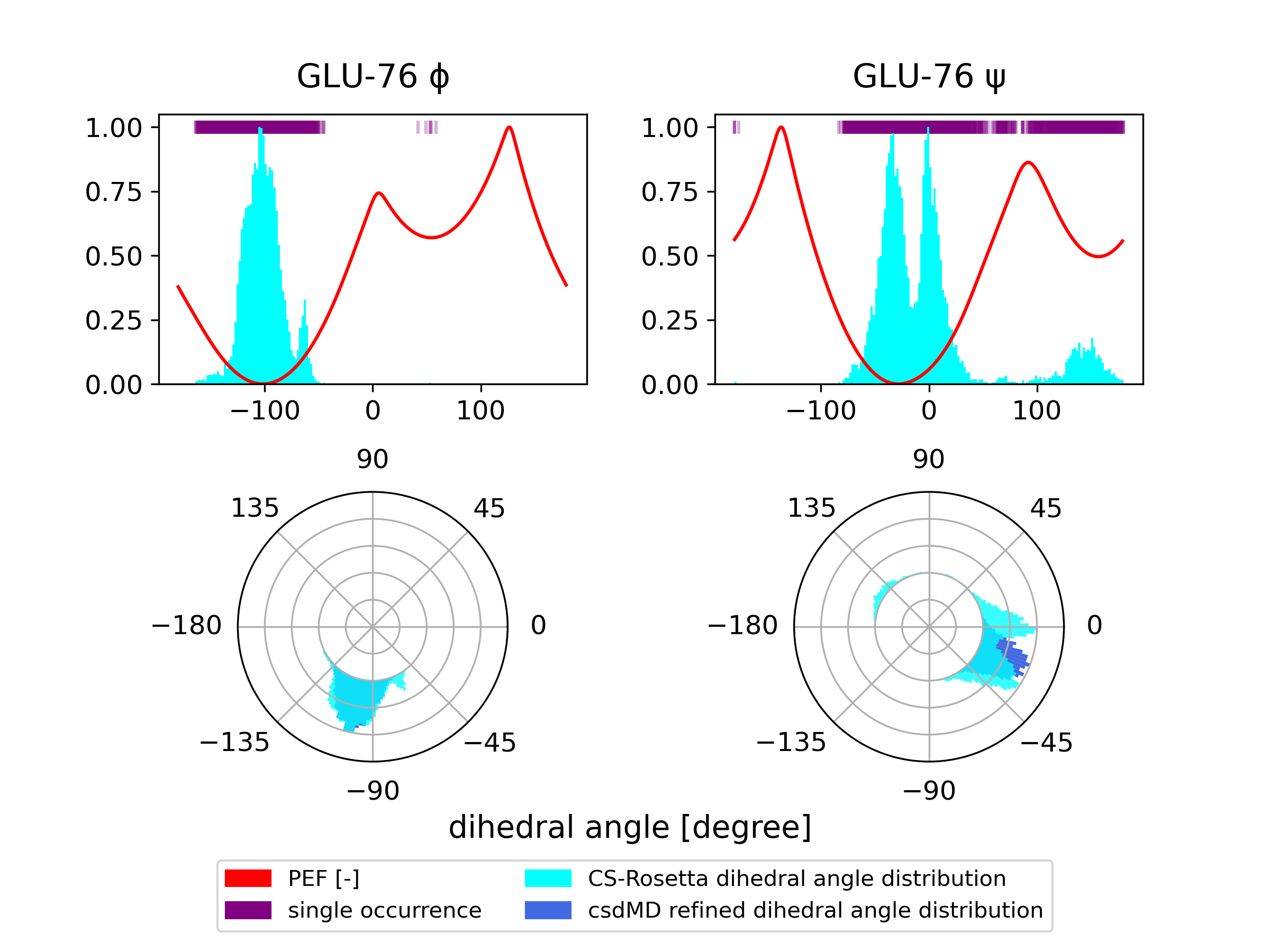

Supplement: Supplementary file 1 [file ijms-24-12101-s001.zip › KRAS-G12C-GDP-Mg-free_angle_figures/76-GLU.png]

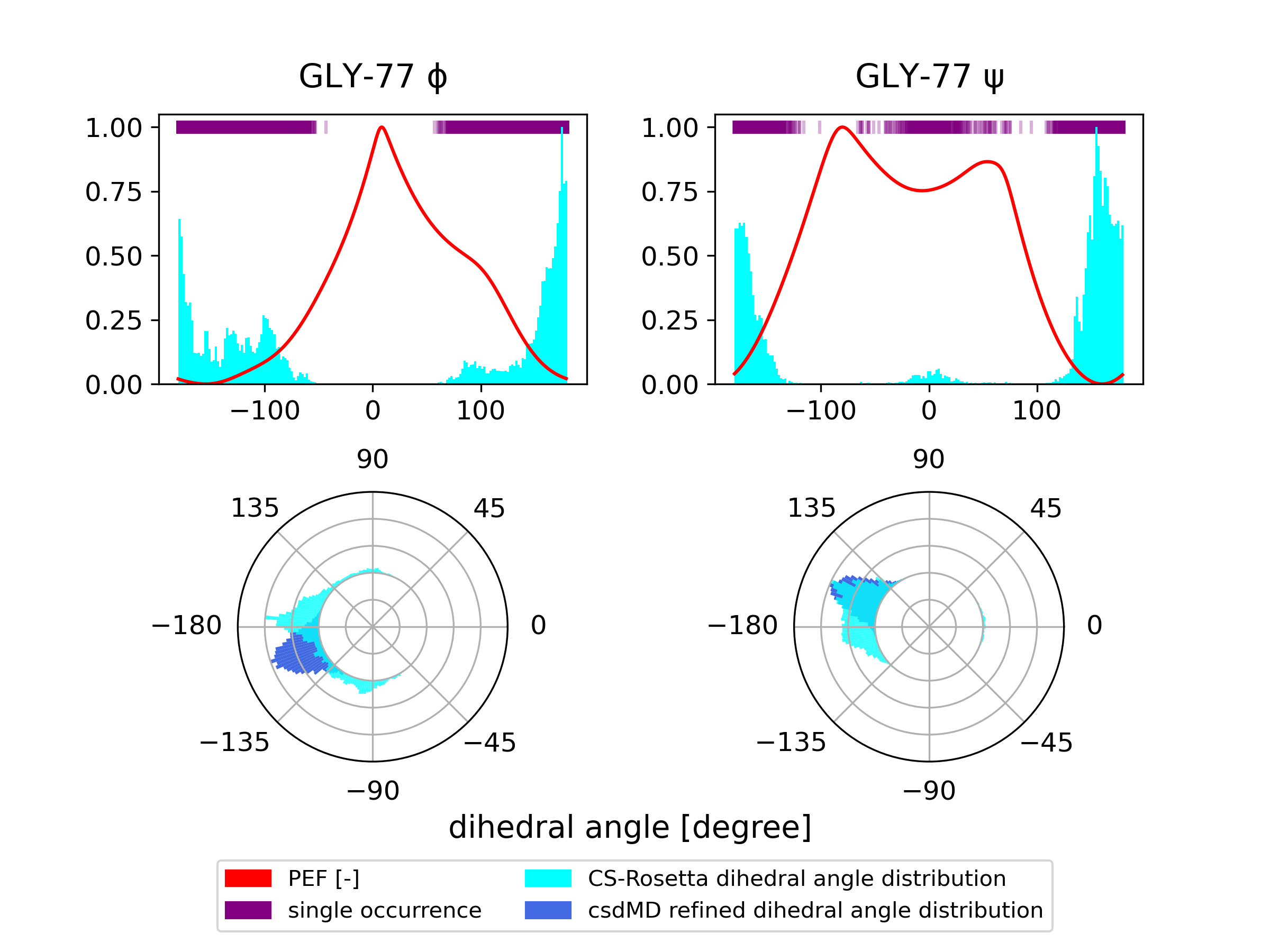

Supplement: Supplementary file 1 [file ijms-24-12101-s001.zip › KRAS-G12C-GDP-Mg-free_angle_figures/77-GLY.png]

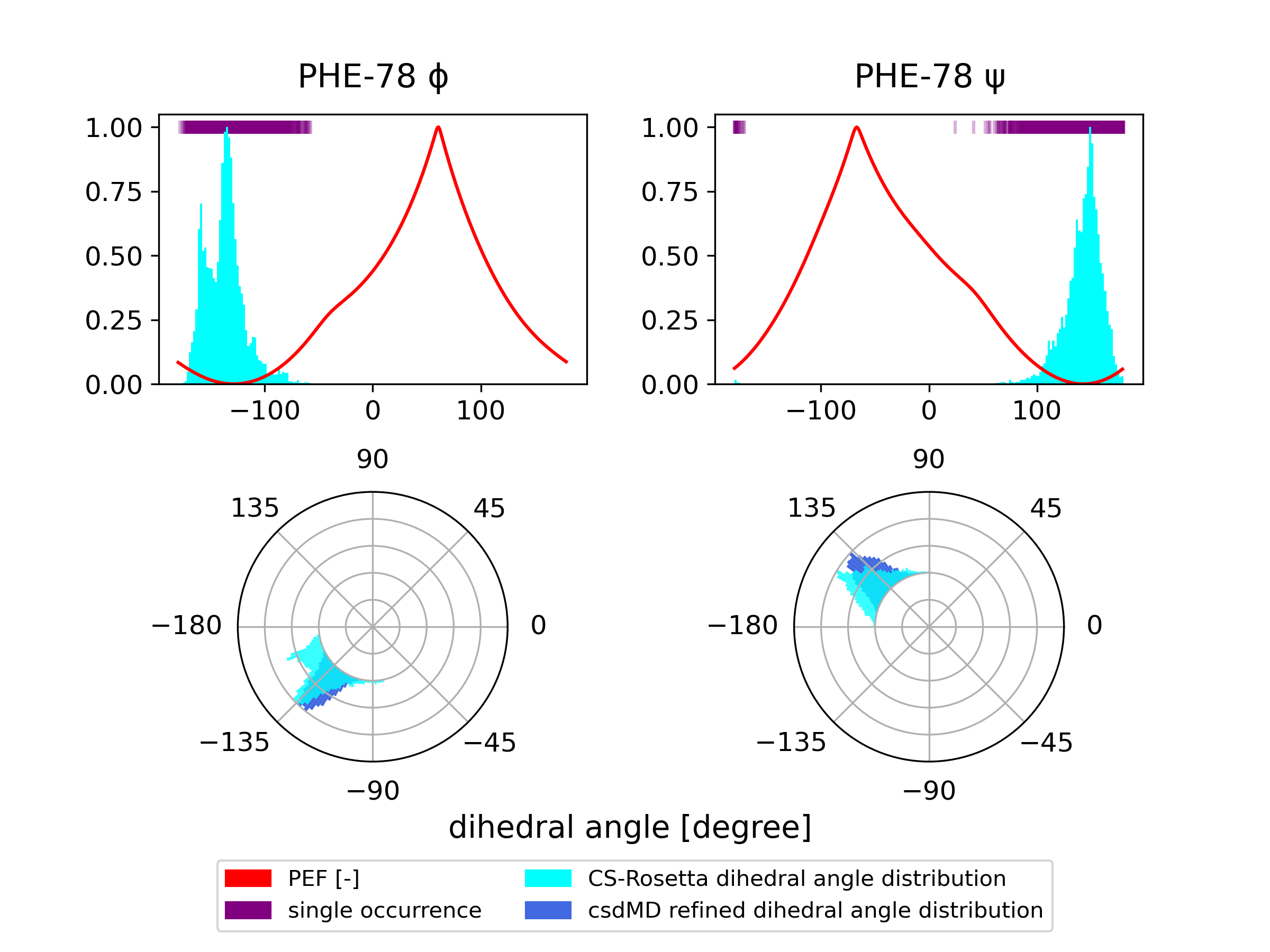

Supplement: Supplementary file 1 [file ijms-24-12101-s001.zip › KRAS-G12C-GDP-Mg-free_angle_figures/78-PHE.png]

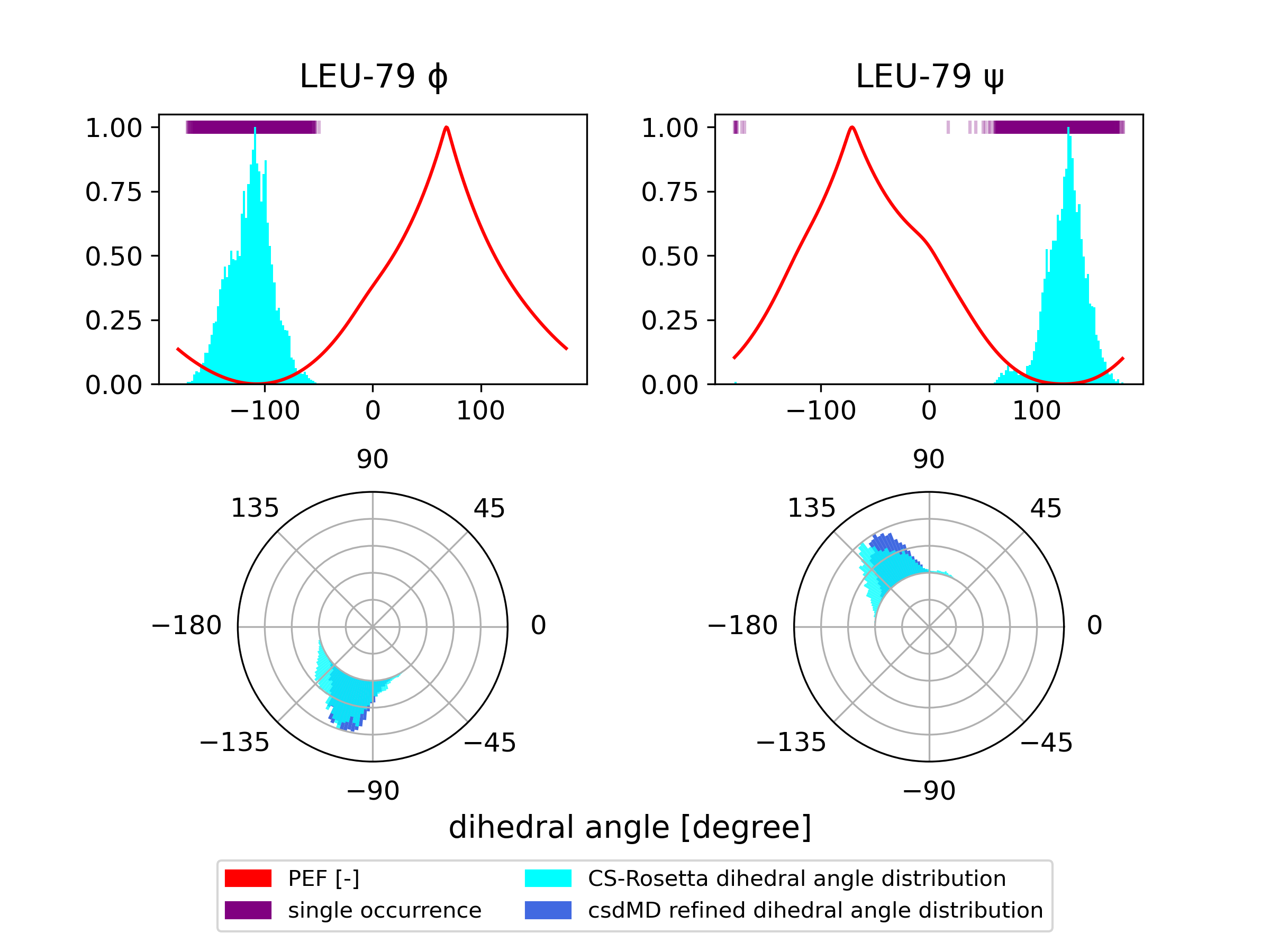

Supplement: Supplementary file 1 [file ijms-24-12101-s001.zip › KRAS-G12C-GDP-Mg-free_angle_figures/79-LEU.png]

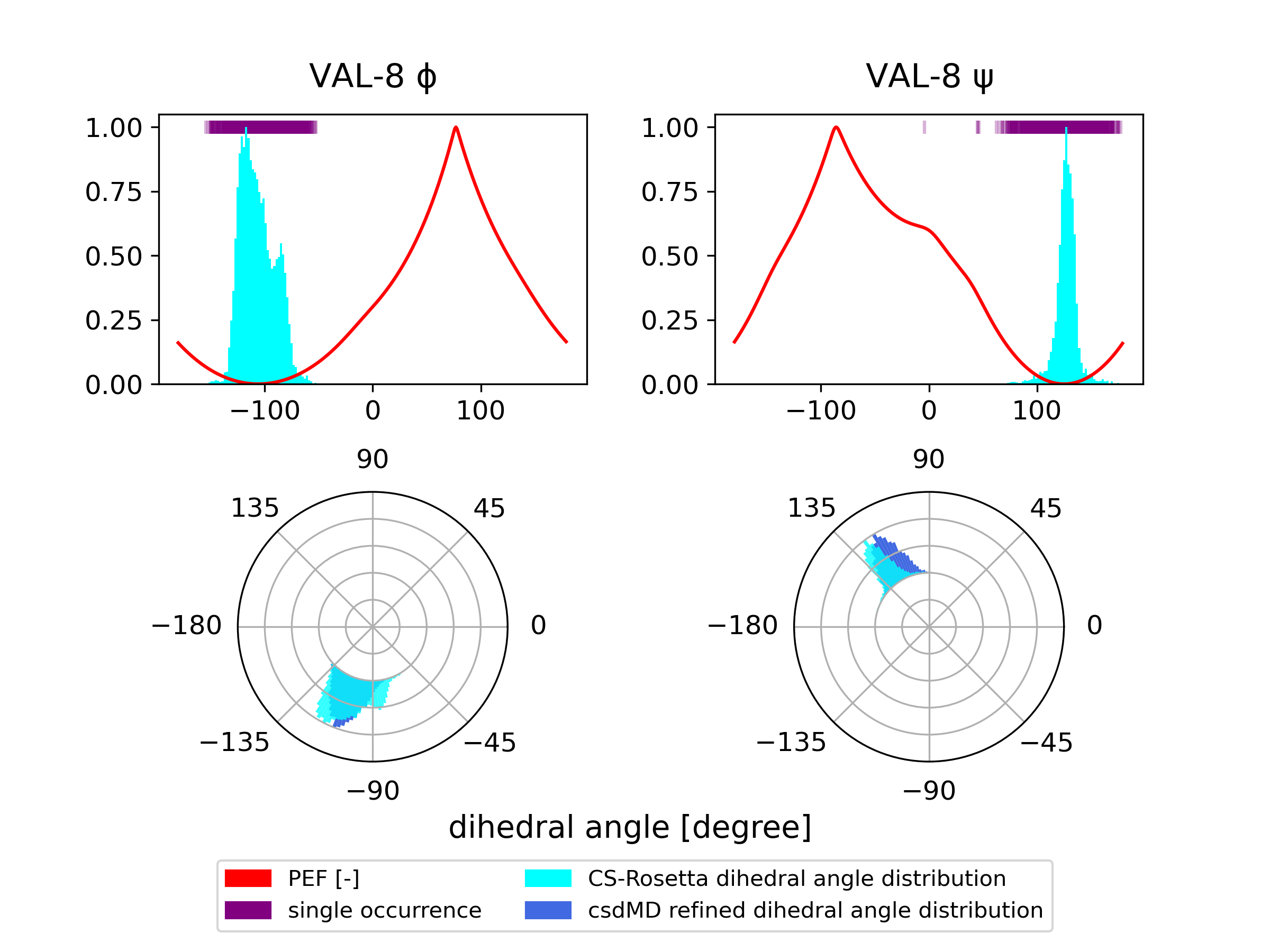

Supplement: Supplementary file 1 [file ijms-24-12101-s001.zip › KRAS-G12C-GDP-Mg-free_angle_figures/8-VAL.png]

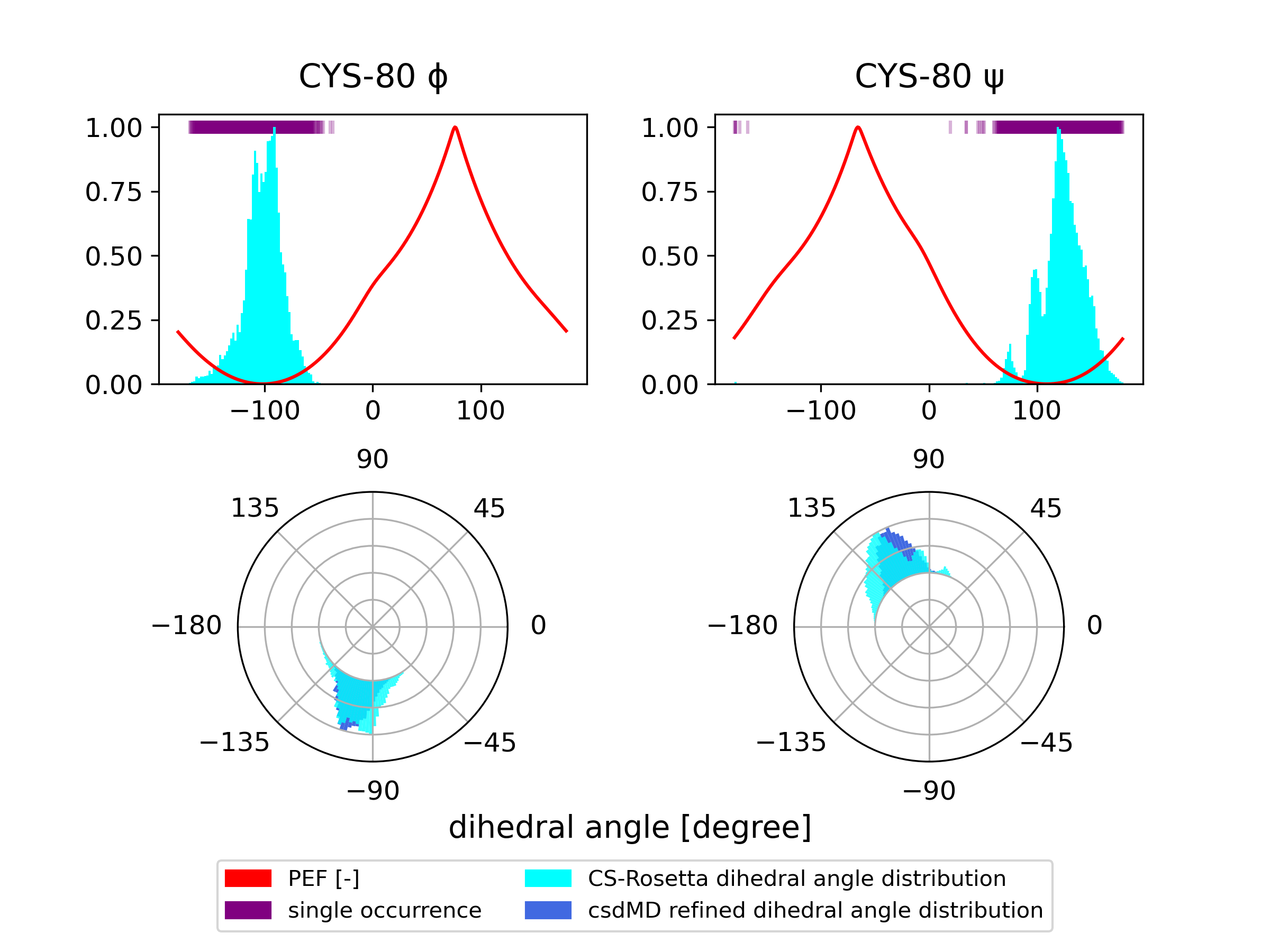

Supplement: Supplementary file 1 [file ijms-24-12101-s001.zip › KRAS-G12C-GDP-Mg-free_angle_figures/80-CYS.png]

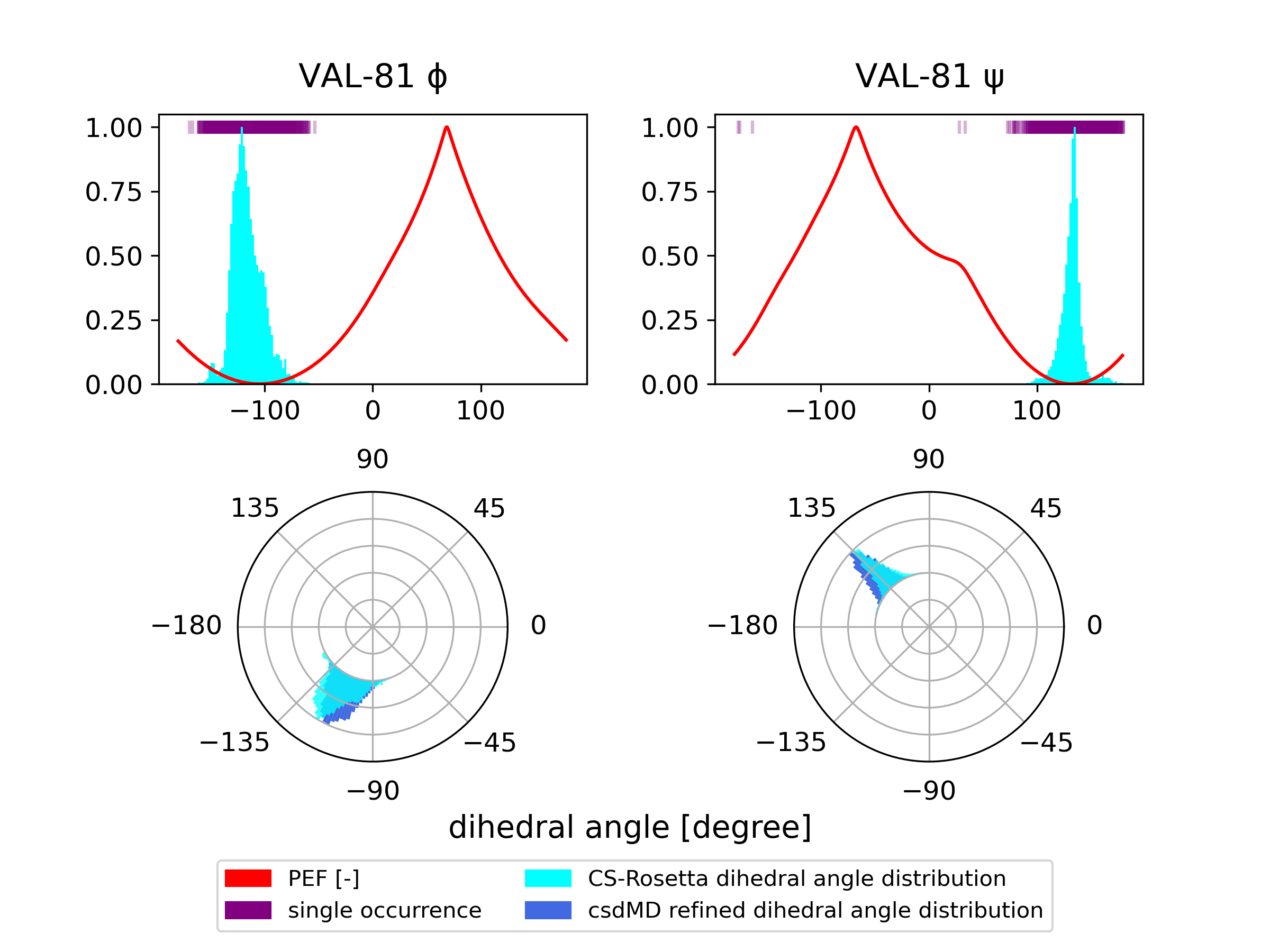

Supplement: Supplementary file 1 [file ijms-24-12101-s001.zip › KRAS-G12C-GDP-Mg-free_angle_figures/81-VAL.png]

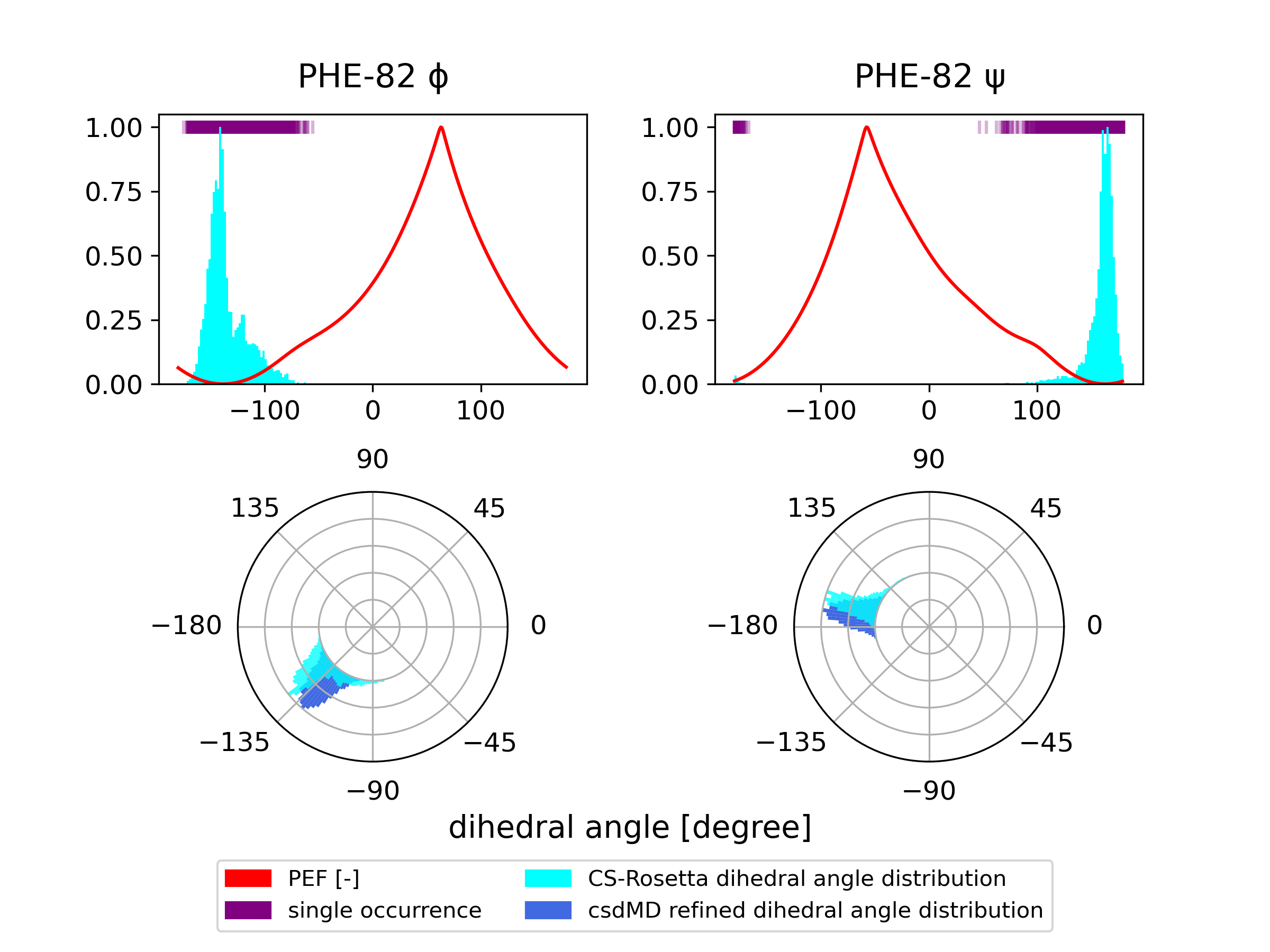

Supplement: Supplementary file 1 [file ijms-24-12101-s001.zip › KRAS-G12C-GDP-Mg-free_angle_figures/82-PHE.png]

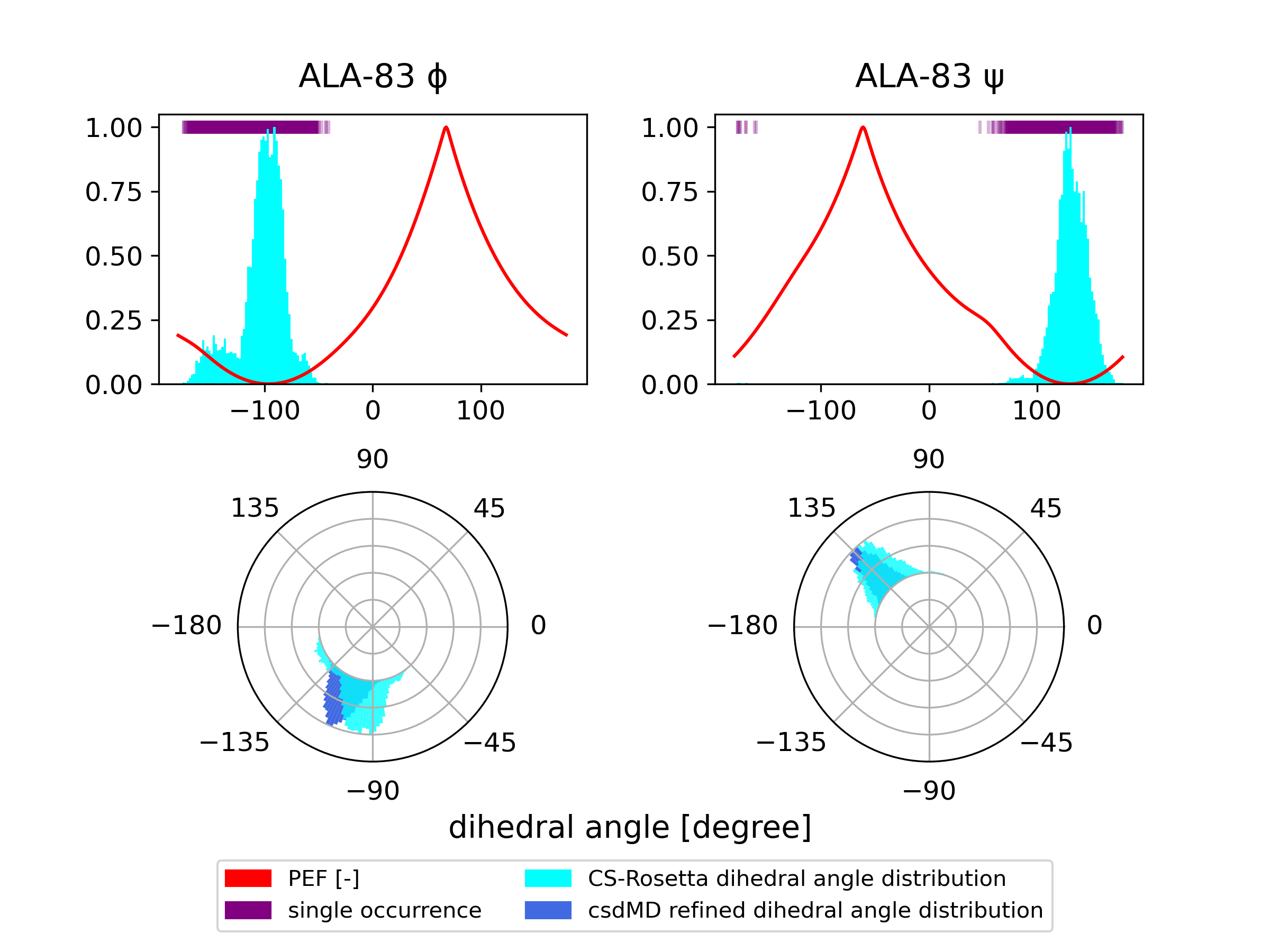

Supplement: Supplementary file 1 [file ijms-24-12101-s001.zip › KRAS-G12C-GDP-Mg-free_angle_figures/83-ALA.png]

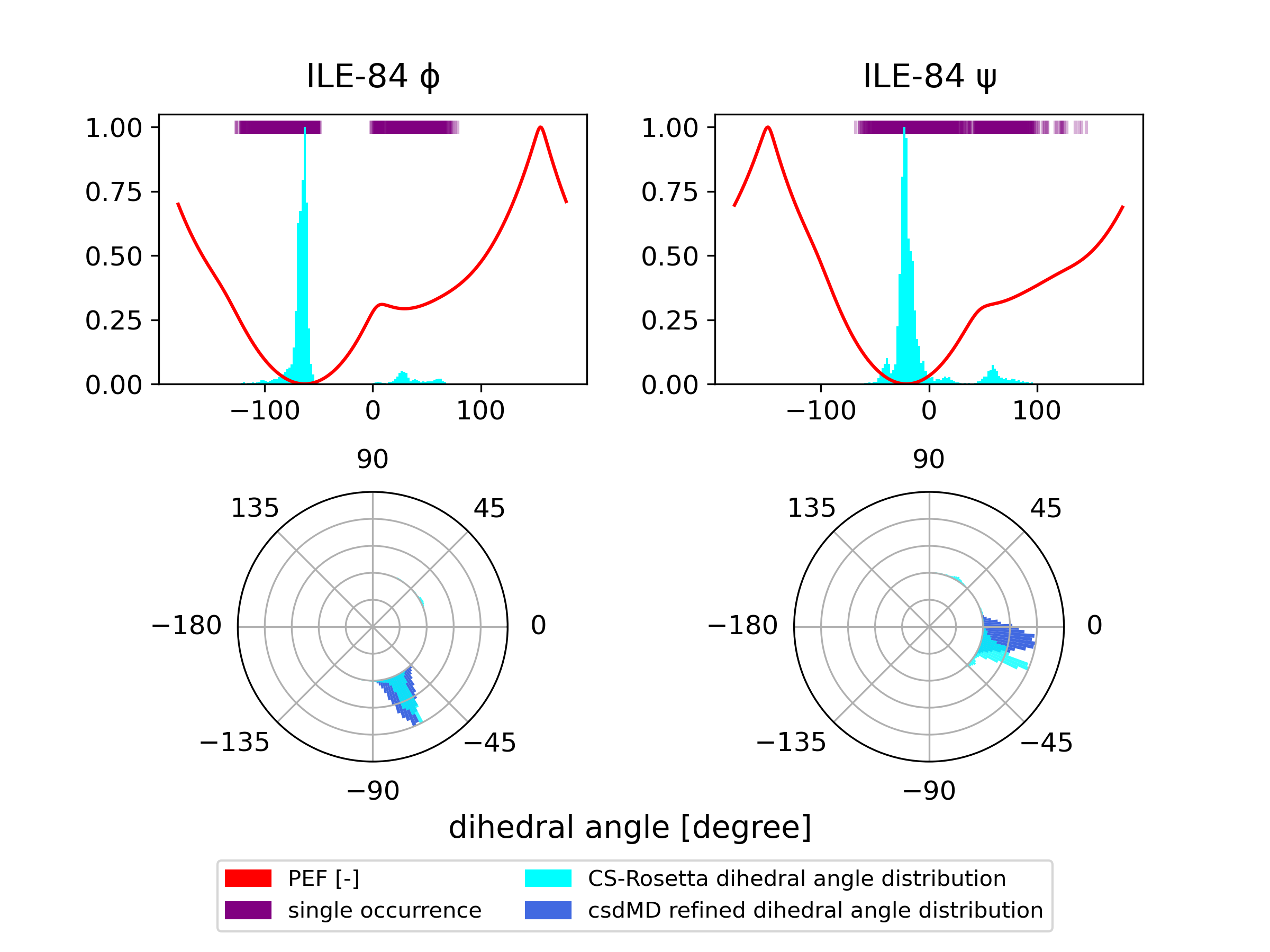

Supplement: Supplementary file 1 [file ijms-24-12101-s001.zip › KRAS-G12C-GDP-Mg-free_angle_figures/84-ILE.png]

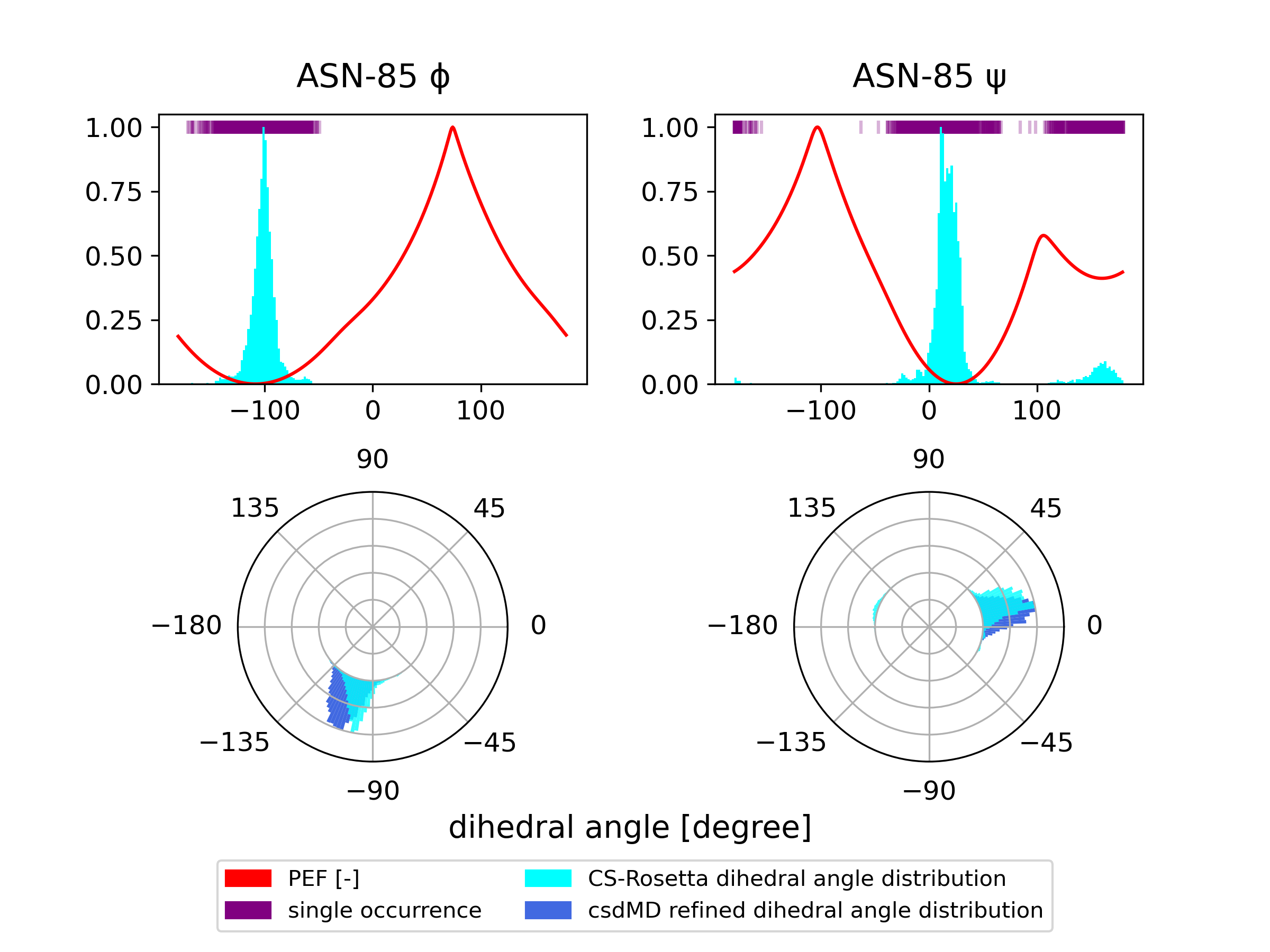

Supplement: Supplementary file 1 [file ijms-24-12101-s001.zip › KRAS-G12C-GDP-Mg-free_angle_figures/85-ASN.png]

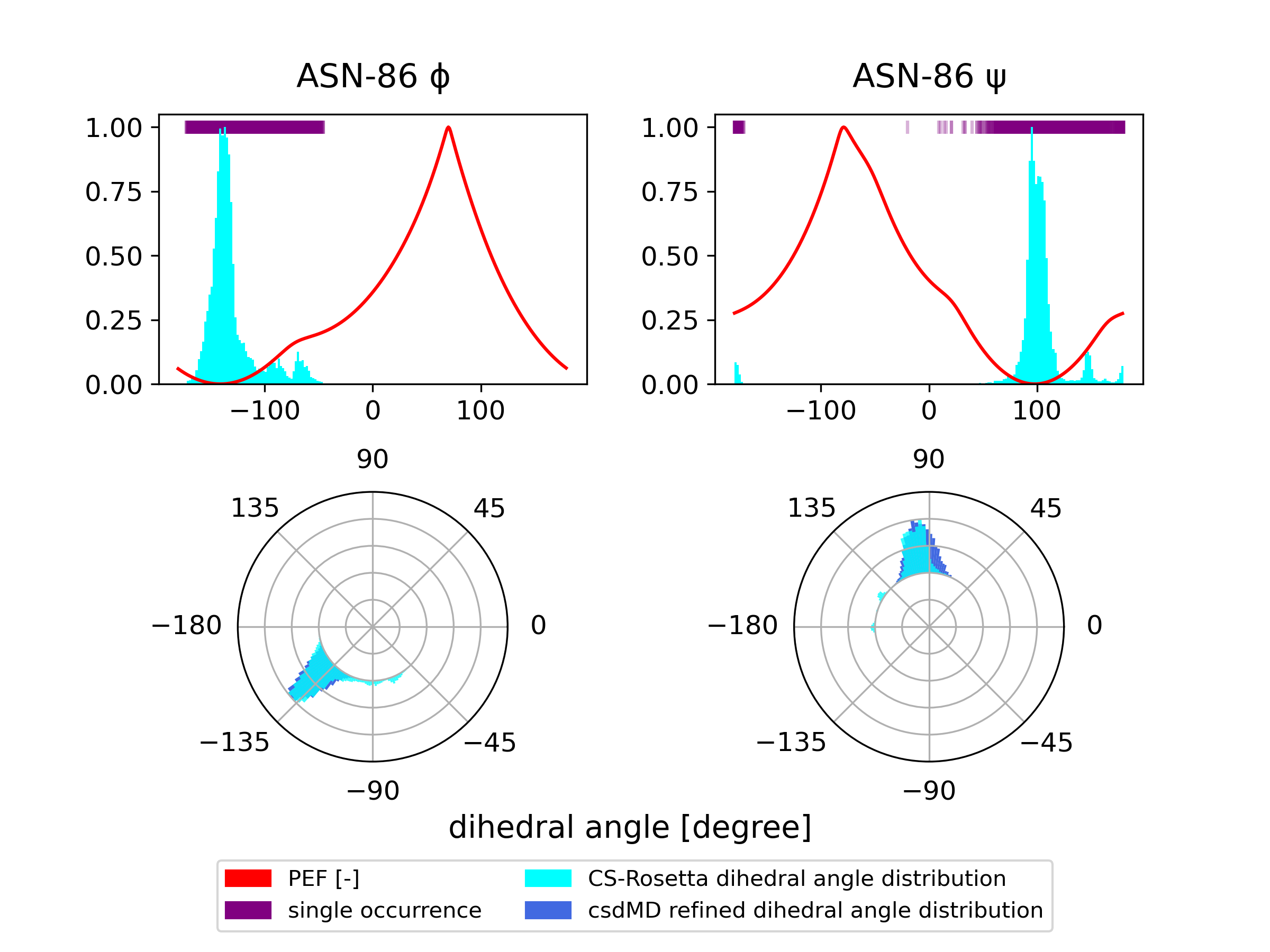

Supplement: Supplementary file 1 [file ijms-24-12101-s001.zip › KRAS-G12C-GDP-Mg-free_angle_figures/86-ASN.png]

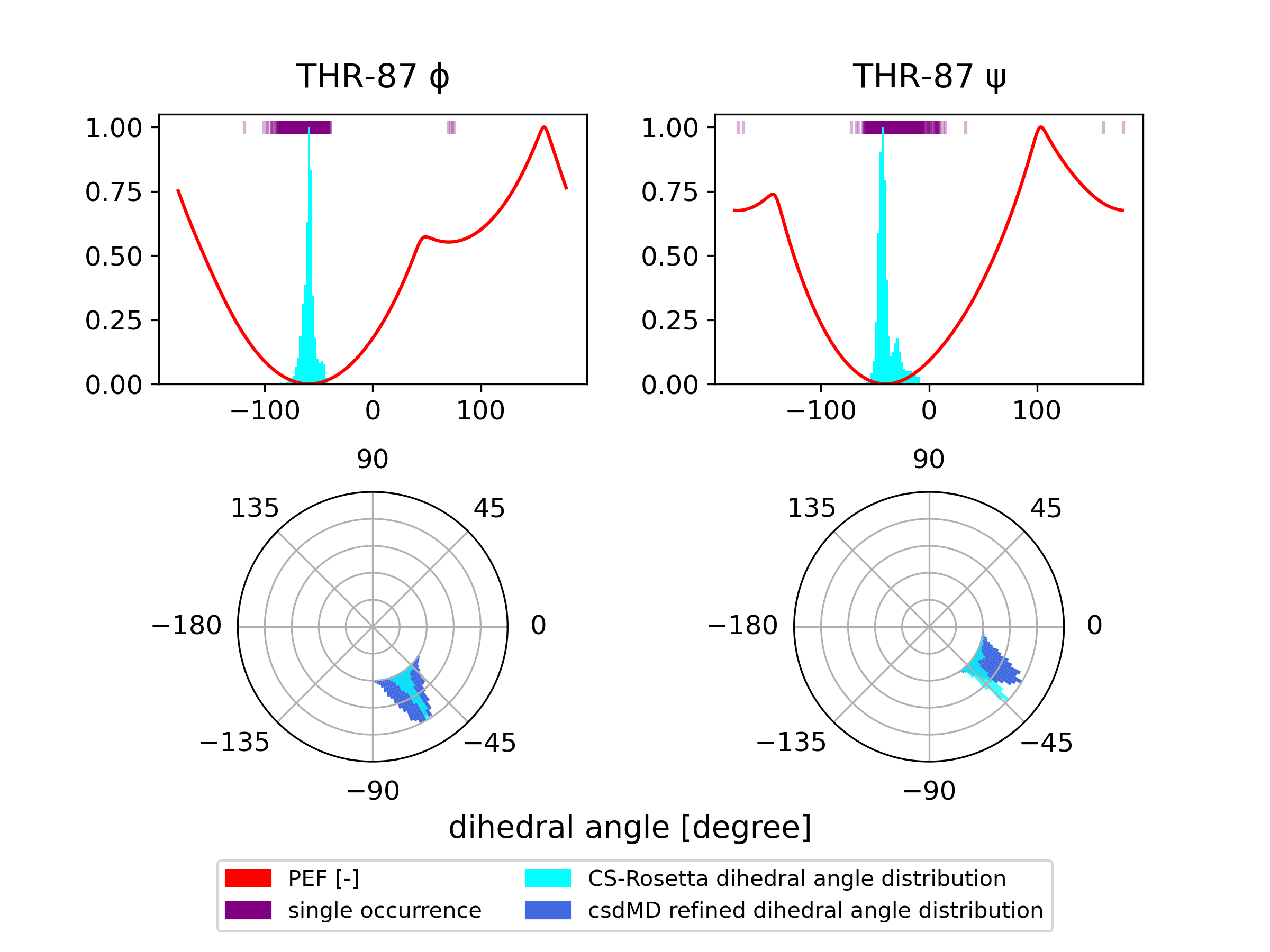

Supplement: Supplementary file 1 [file ijms-24-12101-s001.zip › KRAS-G12C-GDP-Mg-free_angle_figures/87-THR.png]

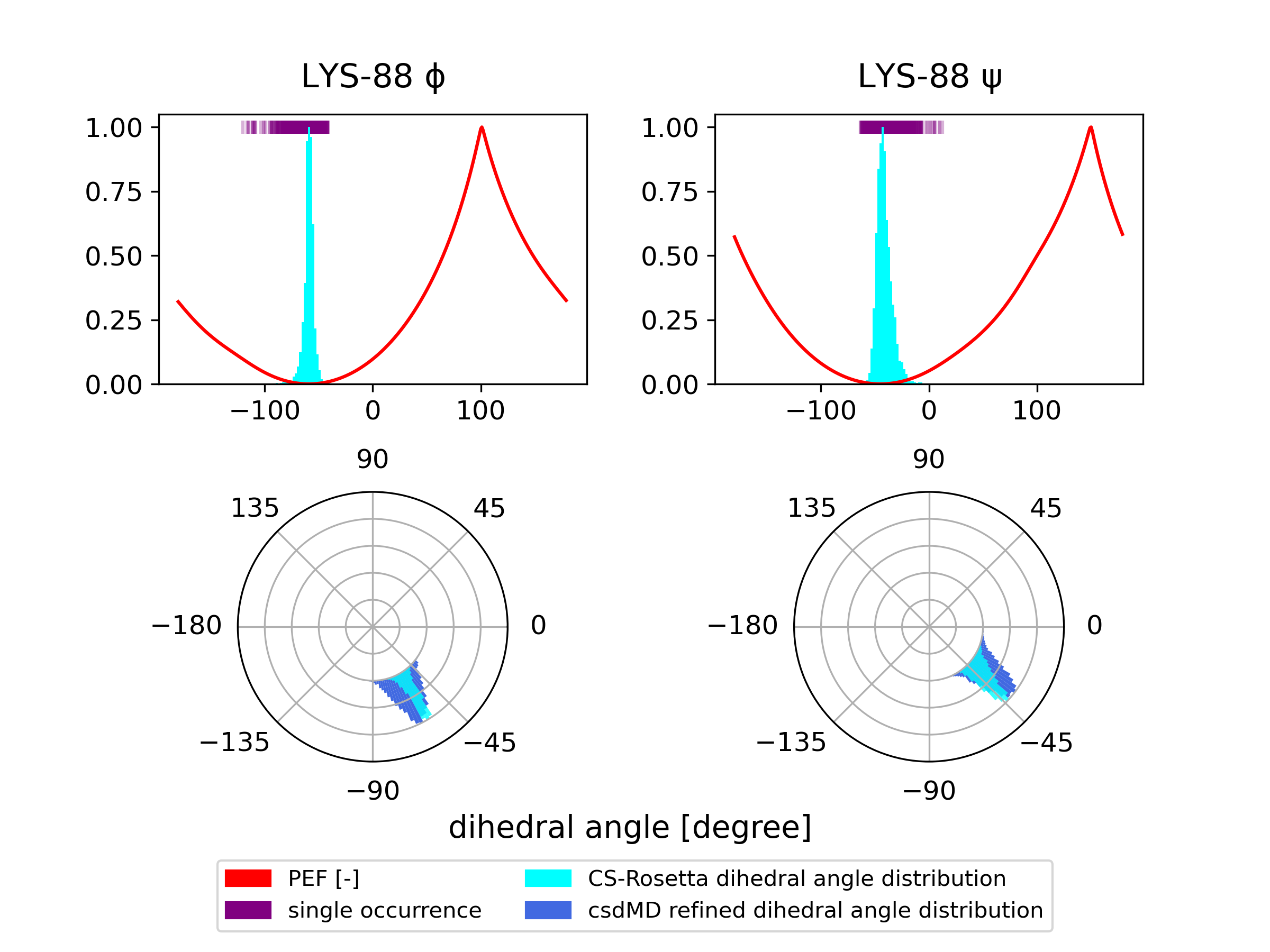

Supplement: Supplementary file 1 [file ijms-24-12101-s001.zip › KRAS-G12C-GDP-Mg-free_angle_figures/88-LYS.png]

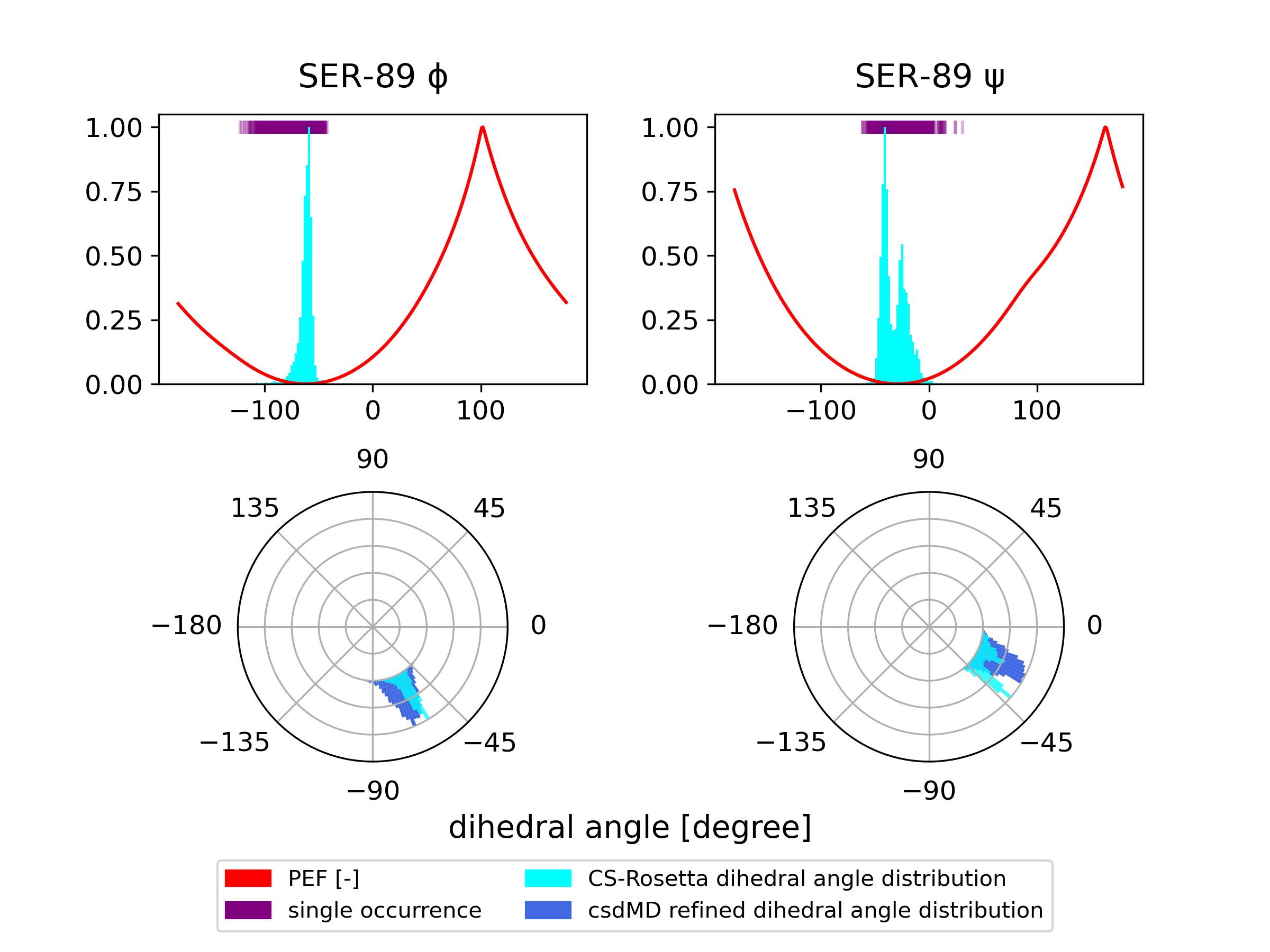

Supplement: Supplementary file 1 [file ijms-24-12101-s001.zip › KRAS-G12C-GDP-Mg-free_angle_figures/89-SER.png]

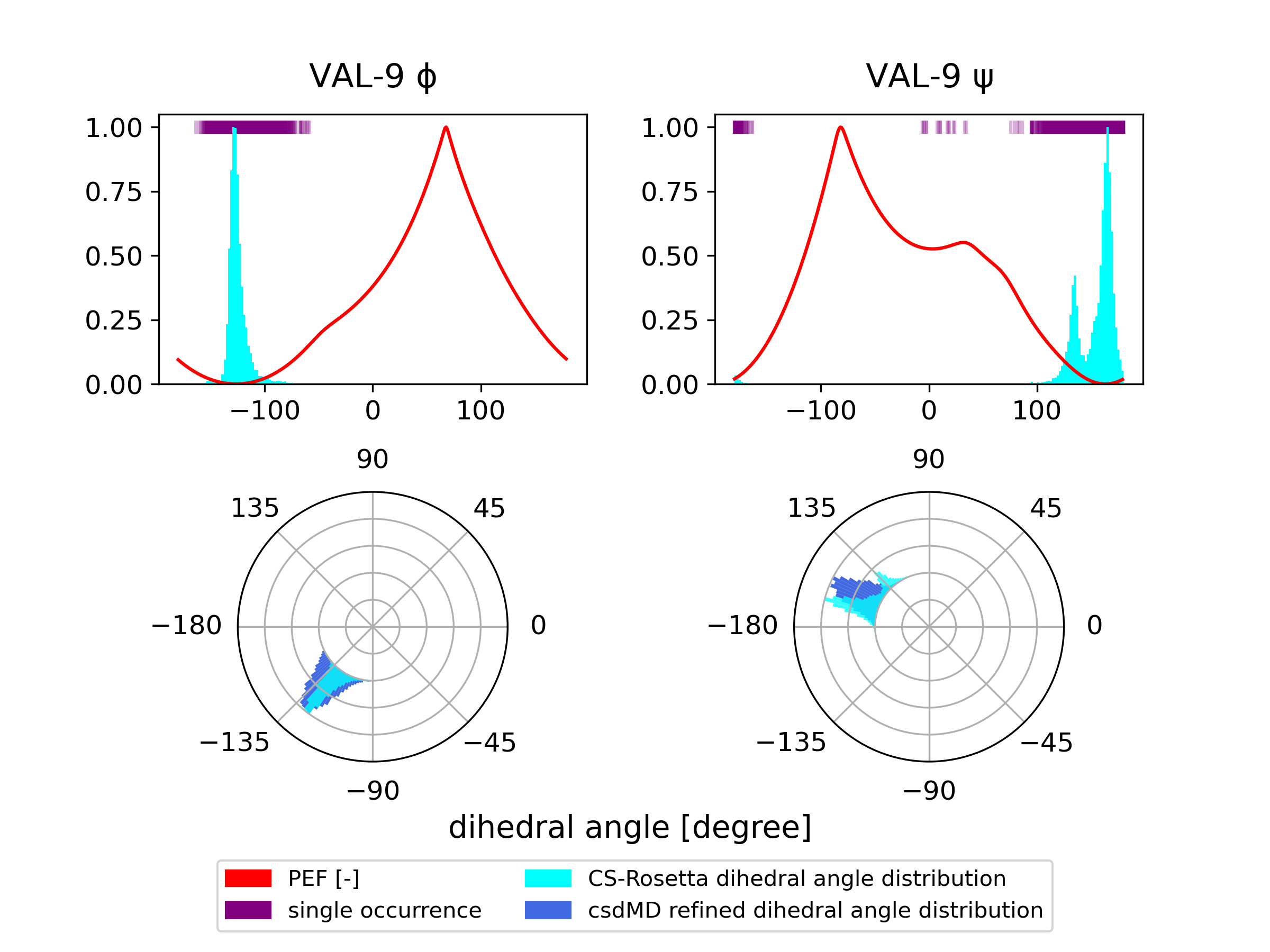

Supplement: Supplementary file 1 [file ijms-24-12101-s001.zip › KRAS-G12C-GDP-Mg-free_angle_figures/9-VAL.png]

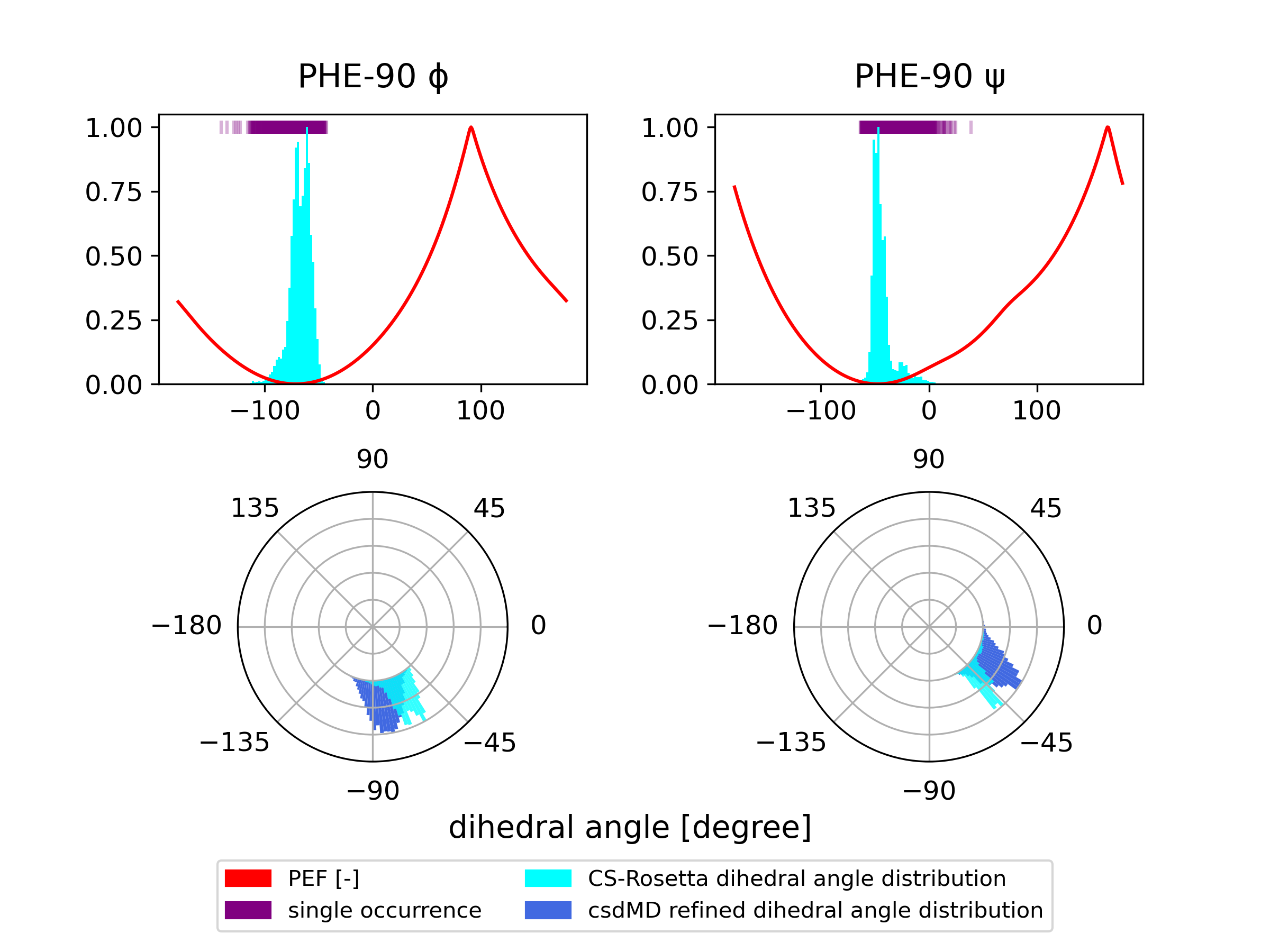

Supplement: Supplementary file 1 [file ijms-24-12101-s001.zip › KRAS-G12C-GDP-Mg-free_angle_figures/90-PHE.png]

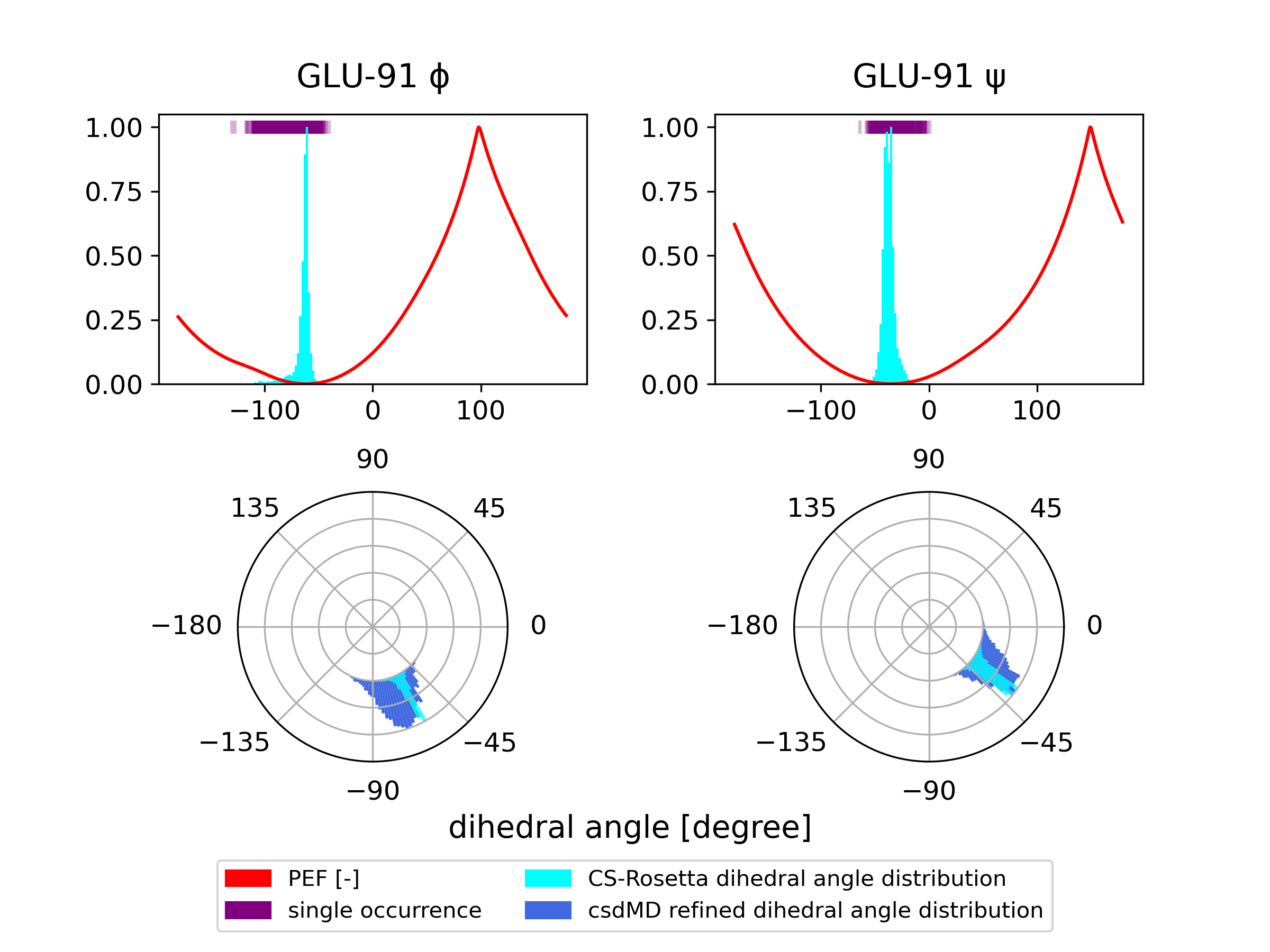

Supplement: Supplementary file 1 [file ijms-24-12101-s001.zip › KRAS-G12C-GDP-Mg-free_angle_figures/91-GLU.png]

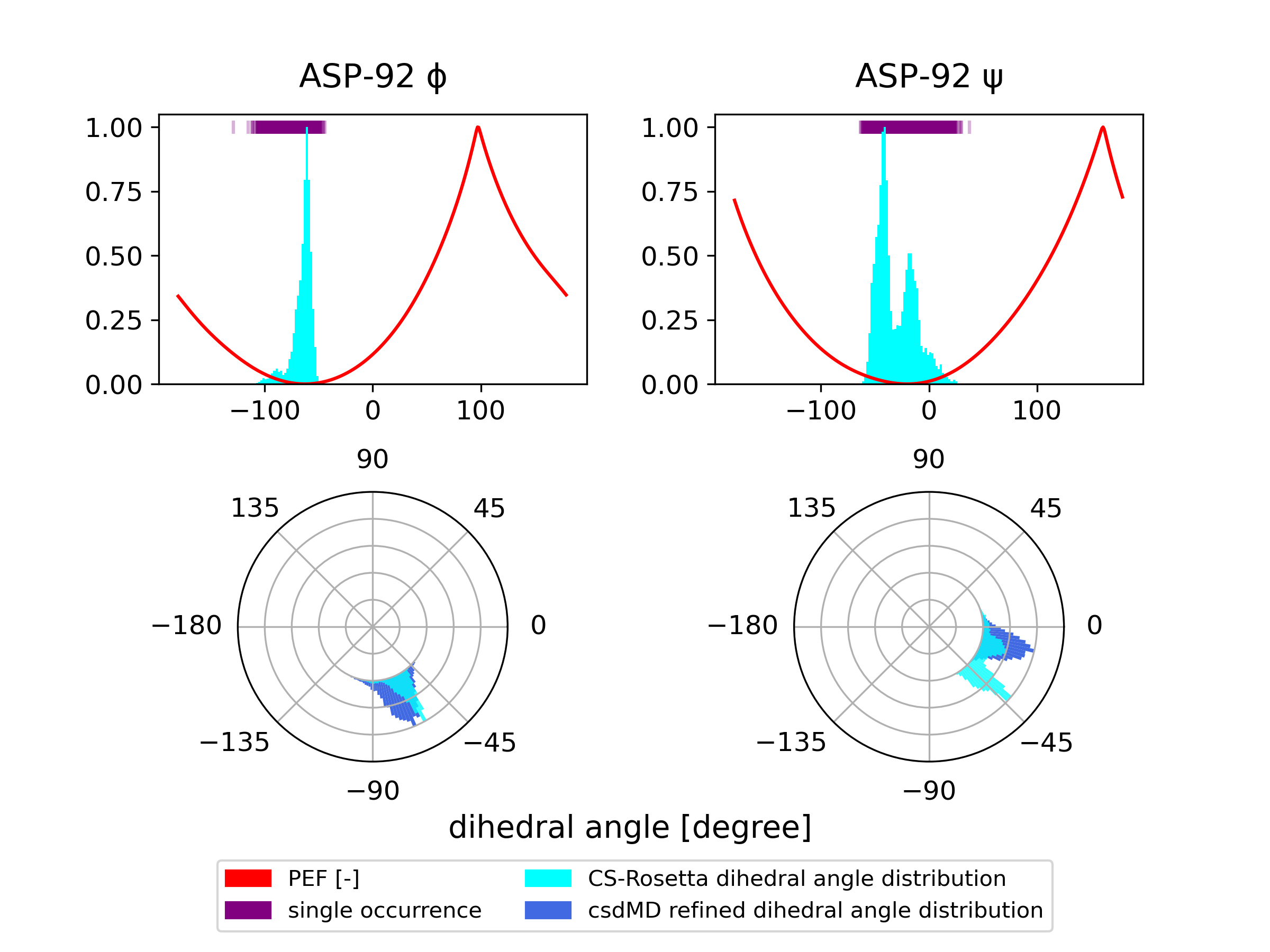

Supplement: Supplementary file 1 [file ijms-24-12101-s001.zip › KRAS-G12C-GDP-Mg-free_angle_figures/92-ASP.png]

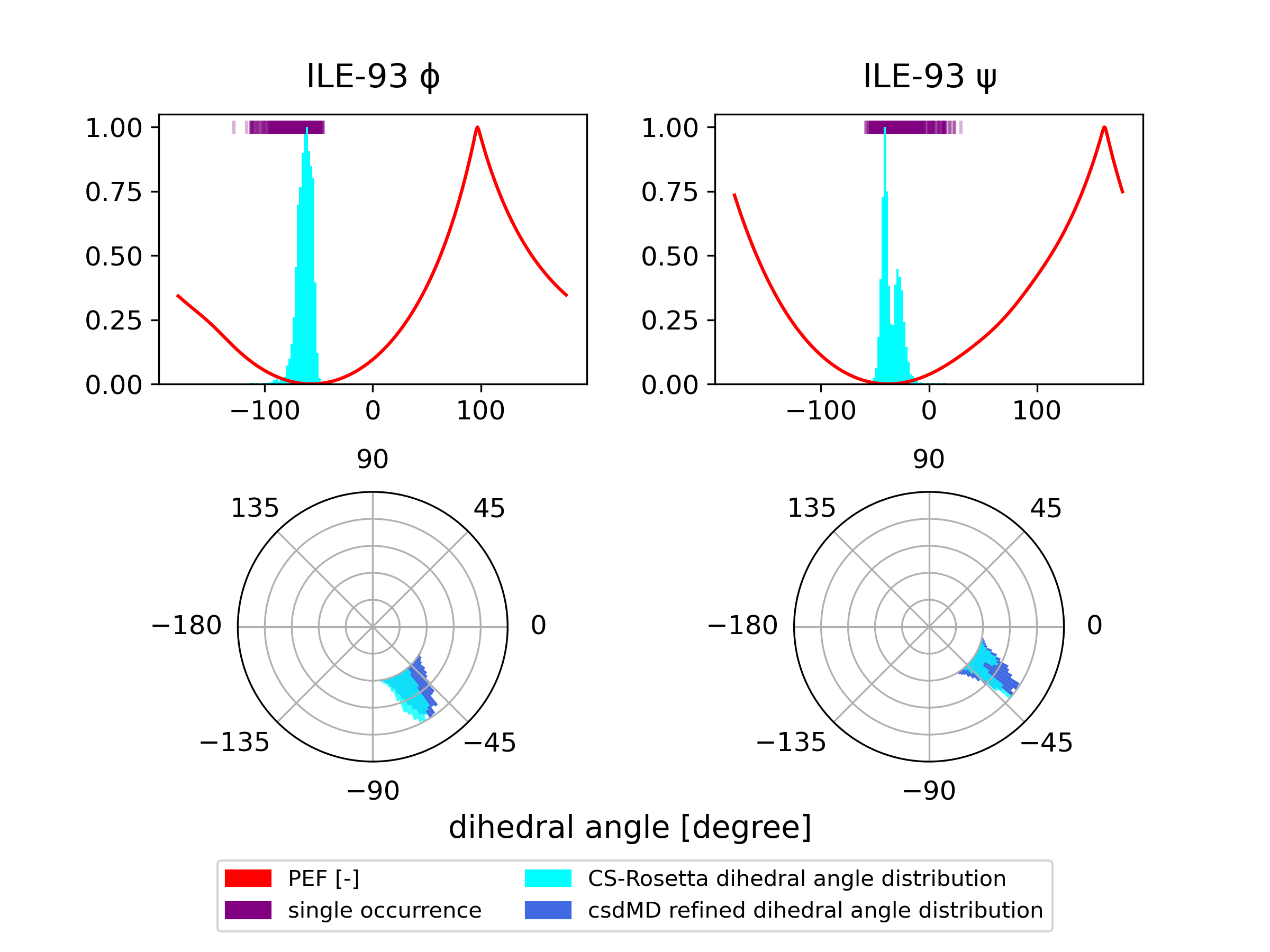

Supplement: Supplementary file 1 [file ijms-24-12101-s001.zip › KRAS-G12C-GDP-Mg-free_angle_figures/93-ILE.png]

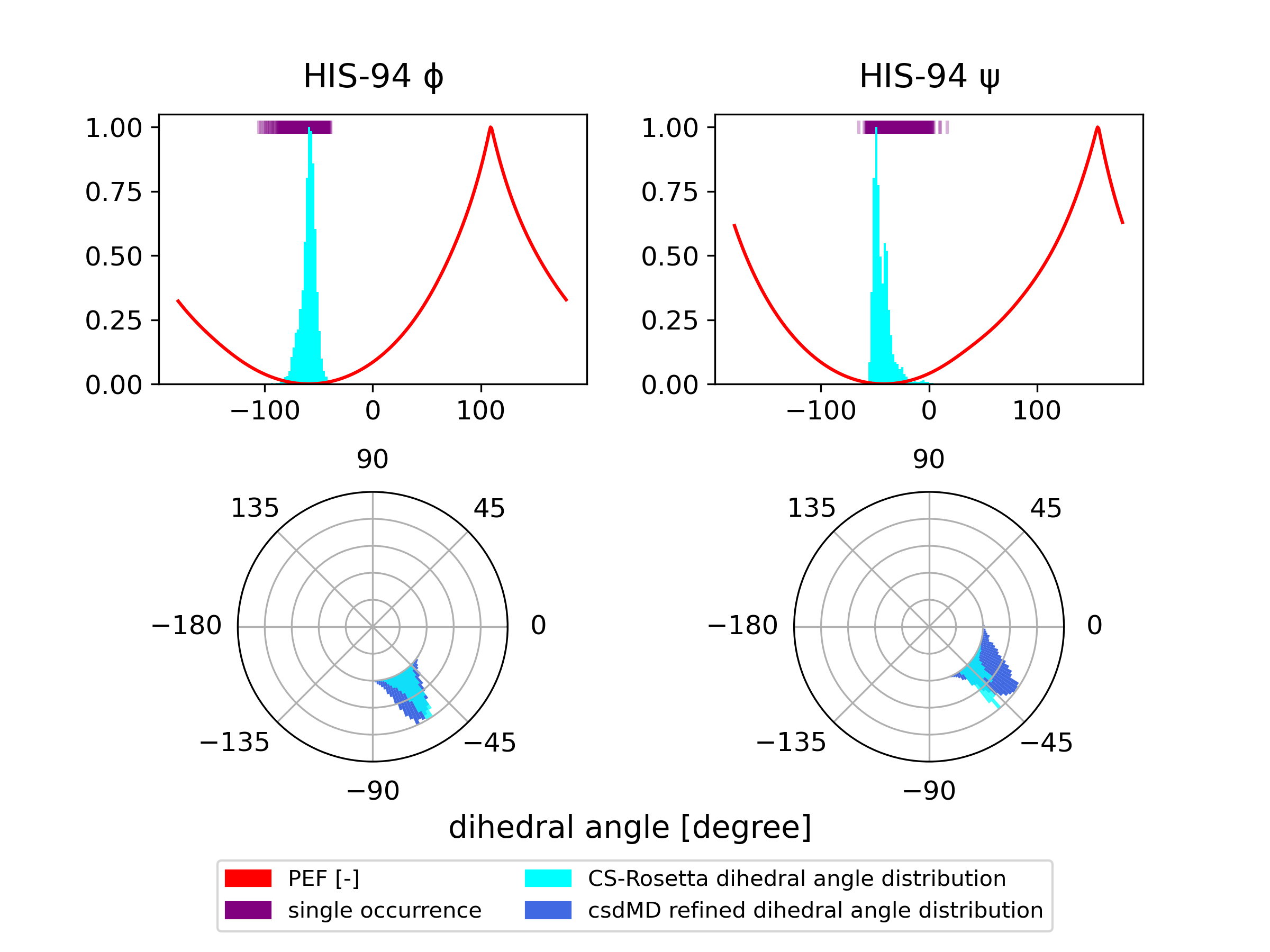

Supplement: Supplementary file 1 [file ijms-24-12101-s001.zip › KRAS-G12C-GDP-Mg-free_angle_figures/94-HIS.png]

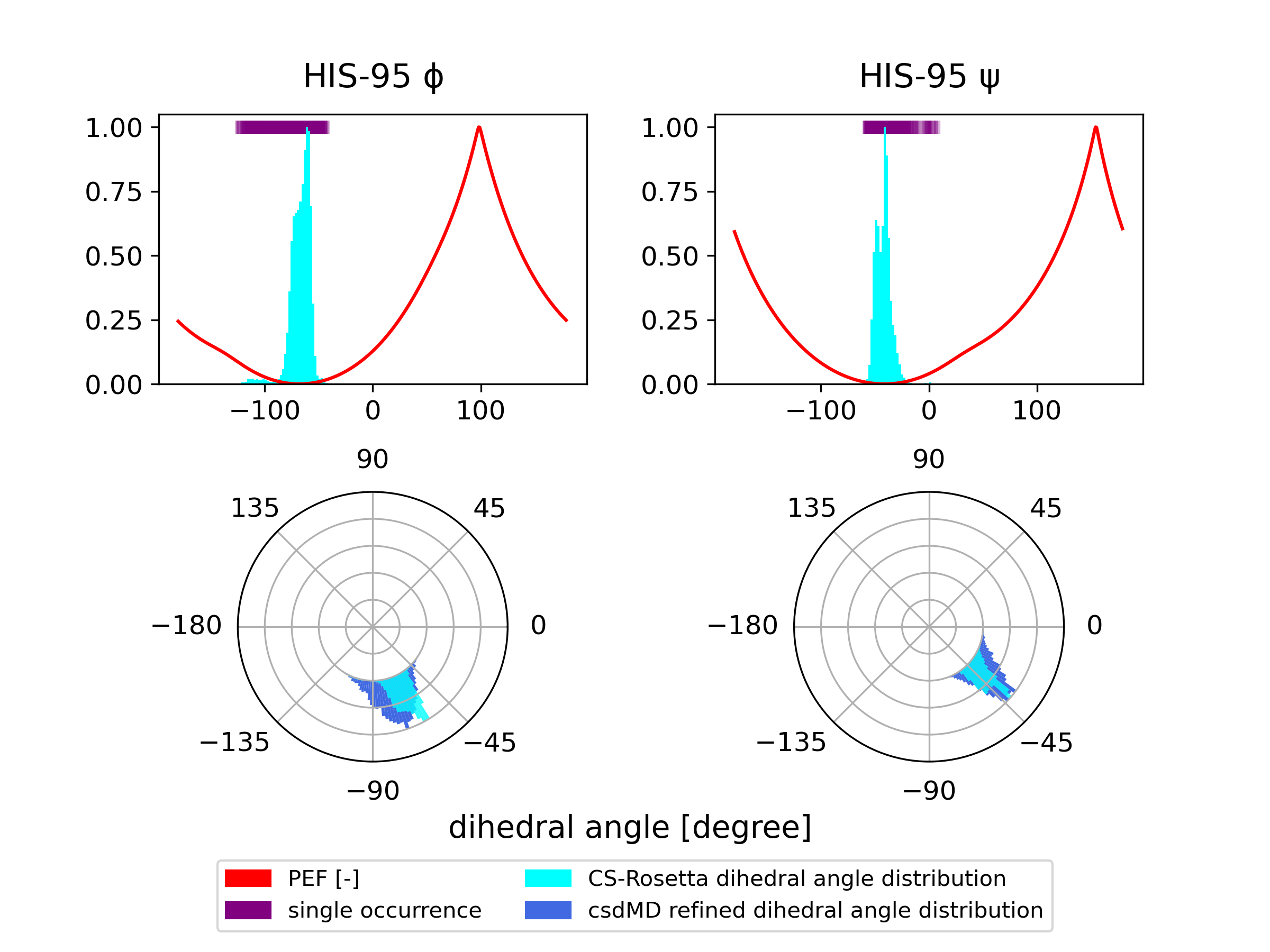

Supplement: Supplementary file 1 [file ijms-24-12101-s001.zip › KRAS-G12C-GDP-Mg-free_angle_figures/95-HIS.png]

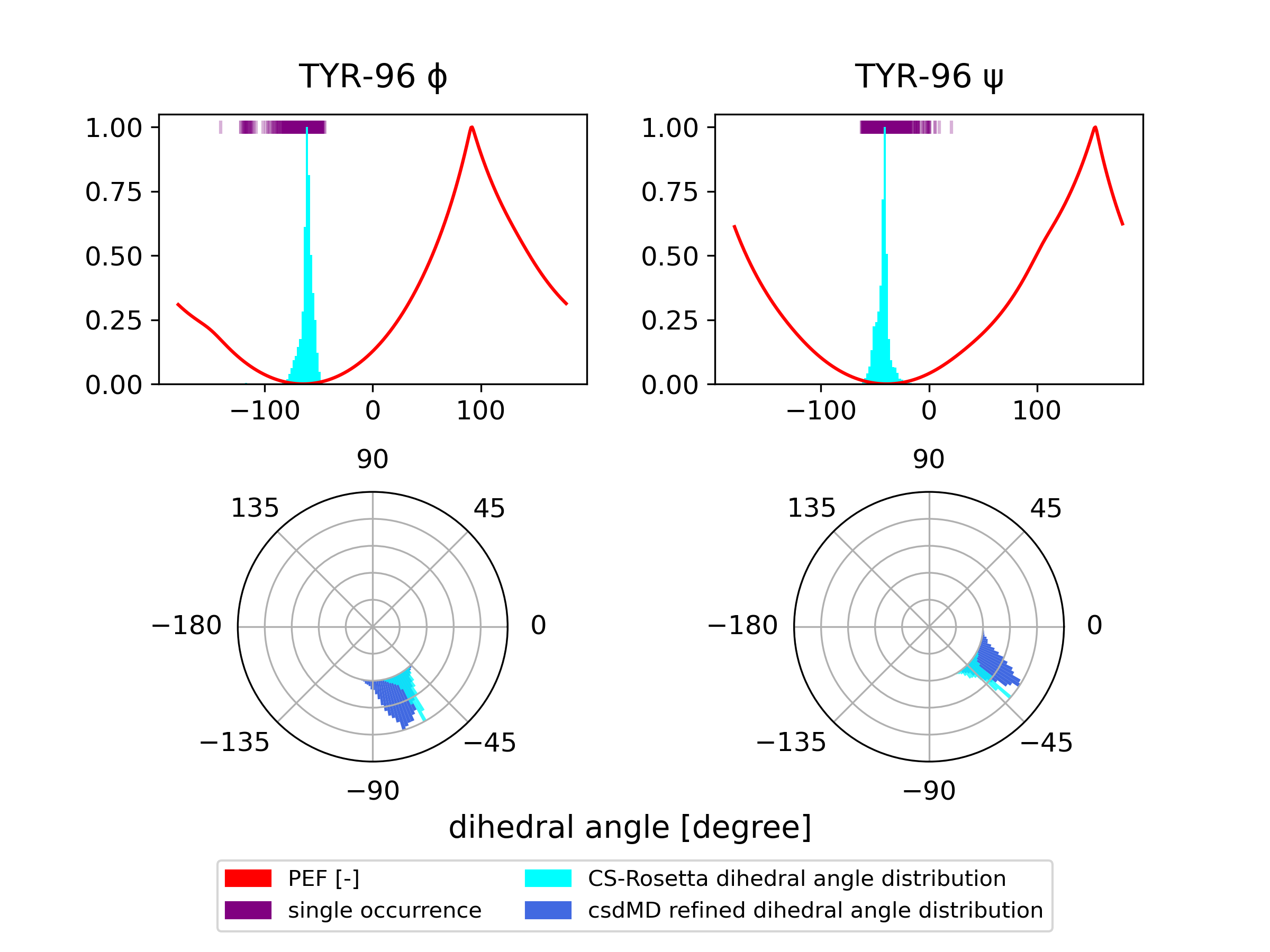

Supplement: Supplementary file 1 [file ijms-24-12101-s001.zip › KRAS-G12C-GDP-Mg-free_angle_figures/96-TYR.png]

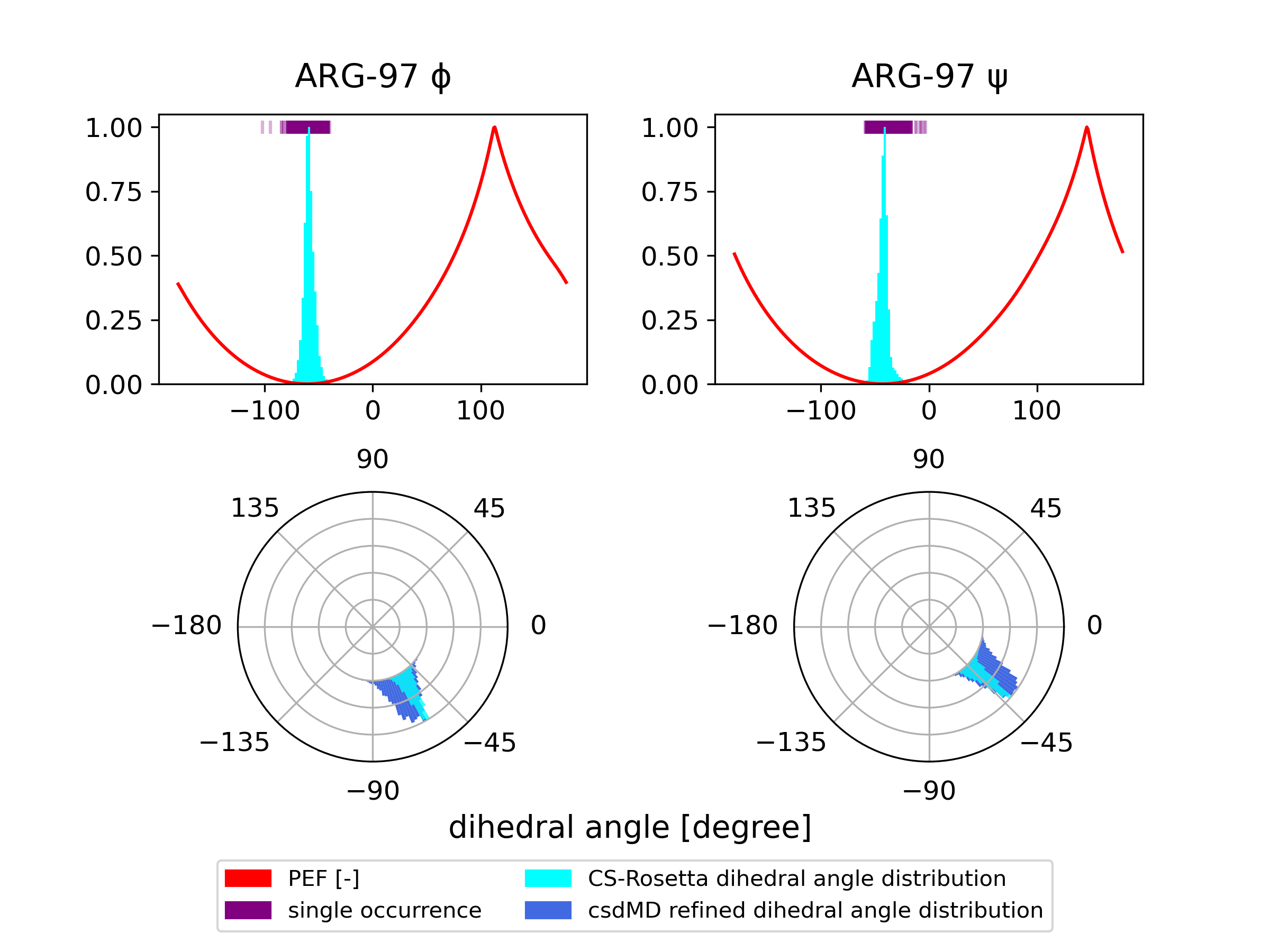

Supplement: Supplementary file 1 [file ijms-24-12101-s001.zip › KRAS-G12C-GDP-Mg-free_angle_figures/97-ARG.png]

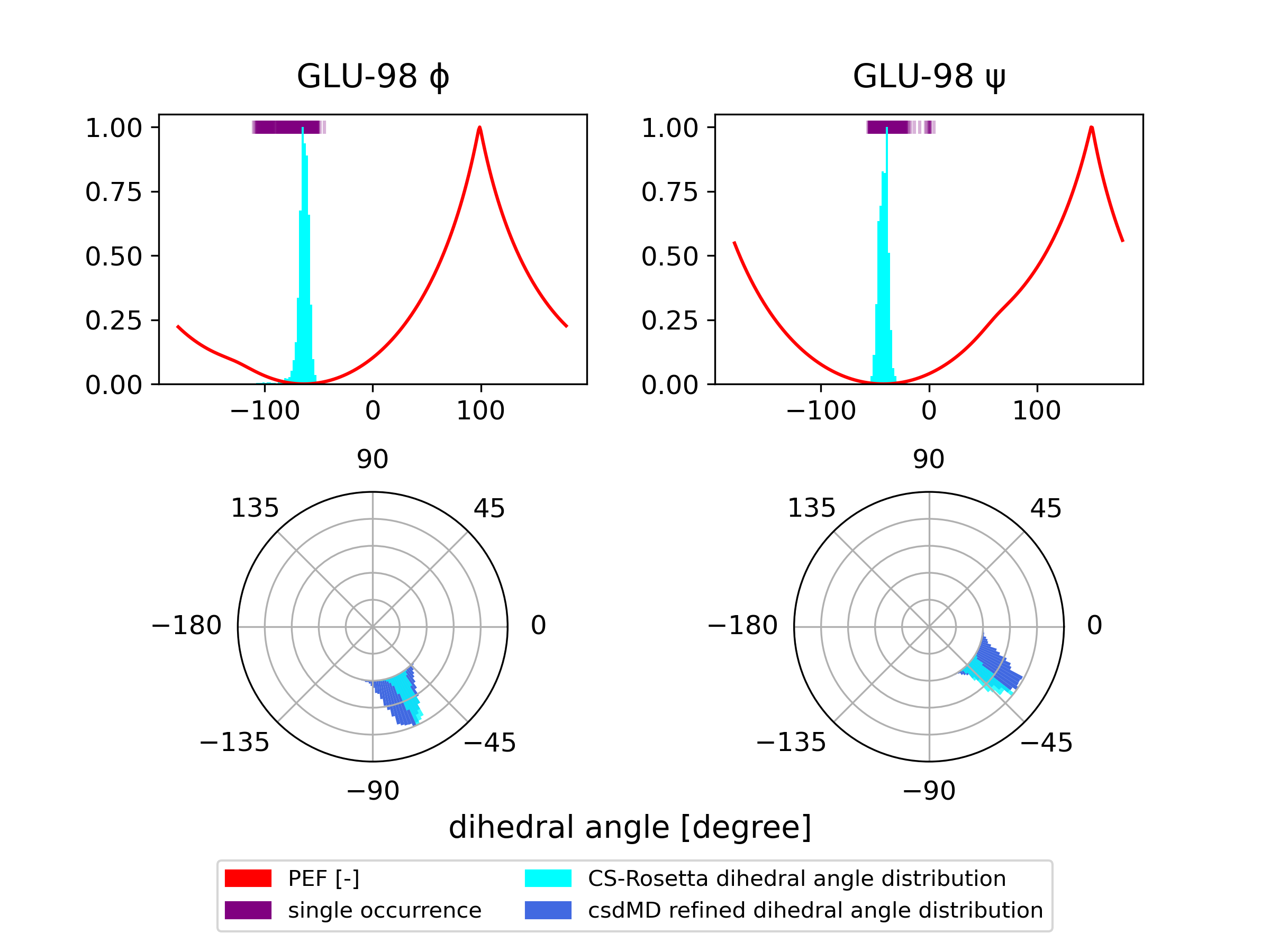

Supplement: Supplementary file 1 [file ijms-24-12101-s001.zip › KRAS-G12C-GDP-Mg-free_angle_figures/98-GLU.png]

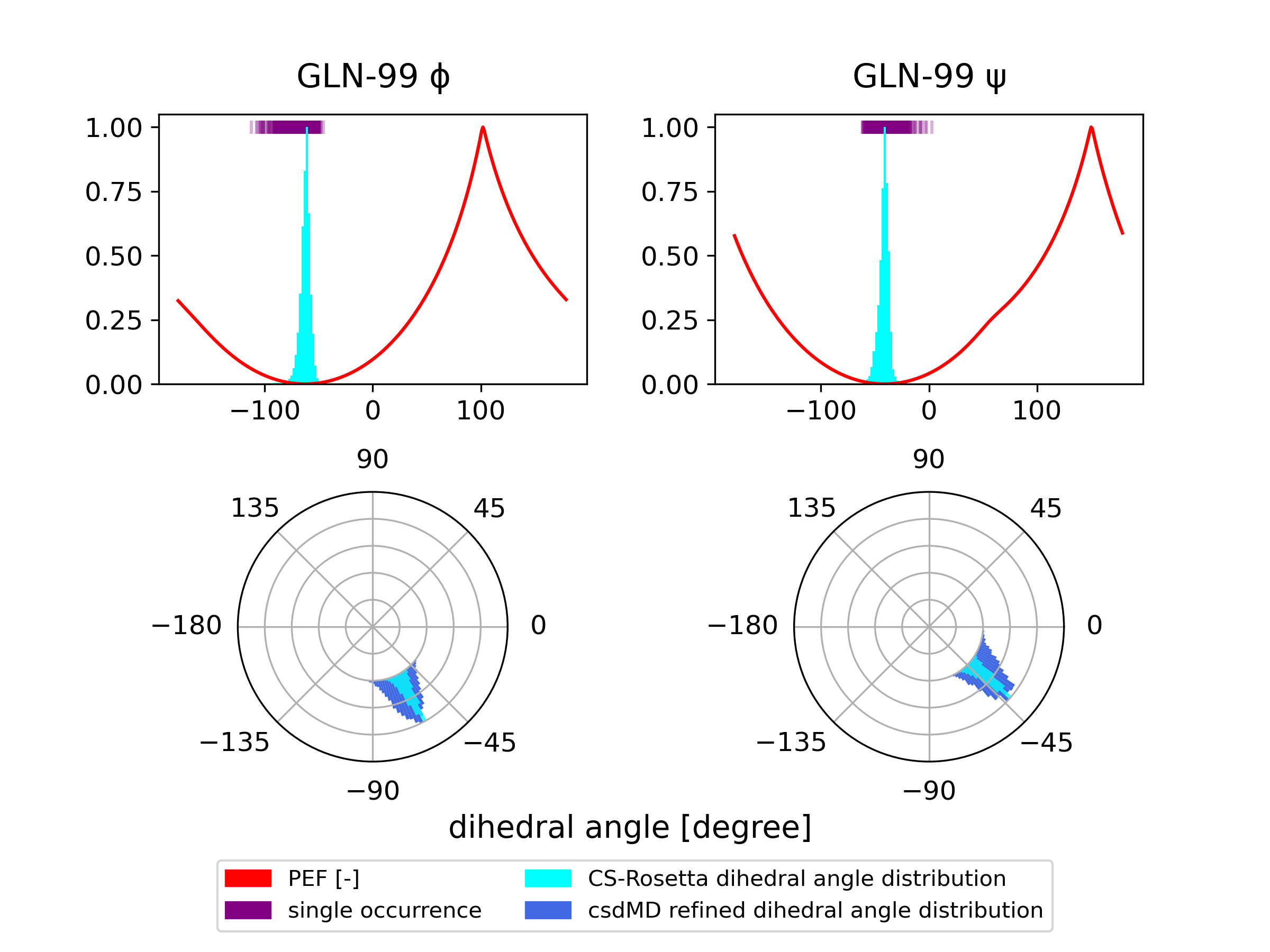

Supplement: Supplementary file 1 [file ijms-24-12101-s001.zip › KRAS-G12C-GDP-Mg-free_angle_figures/99-GLN.png]

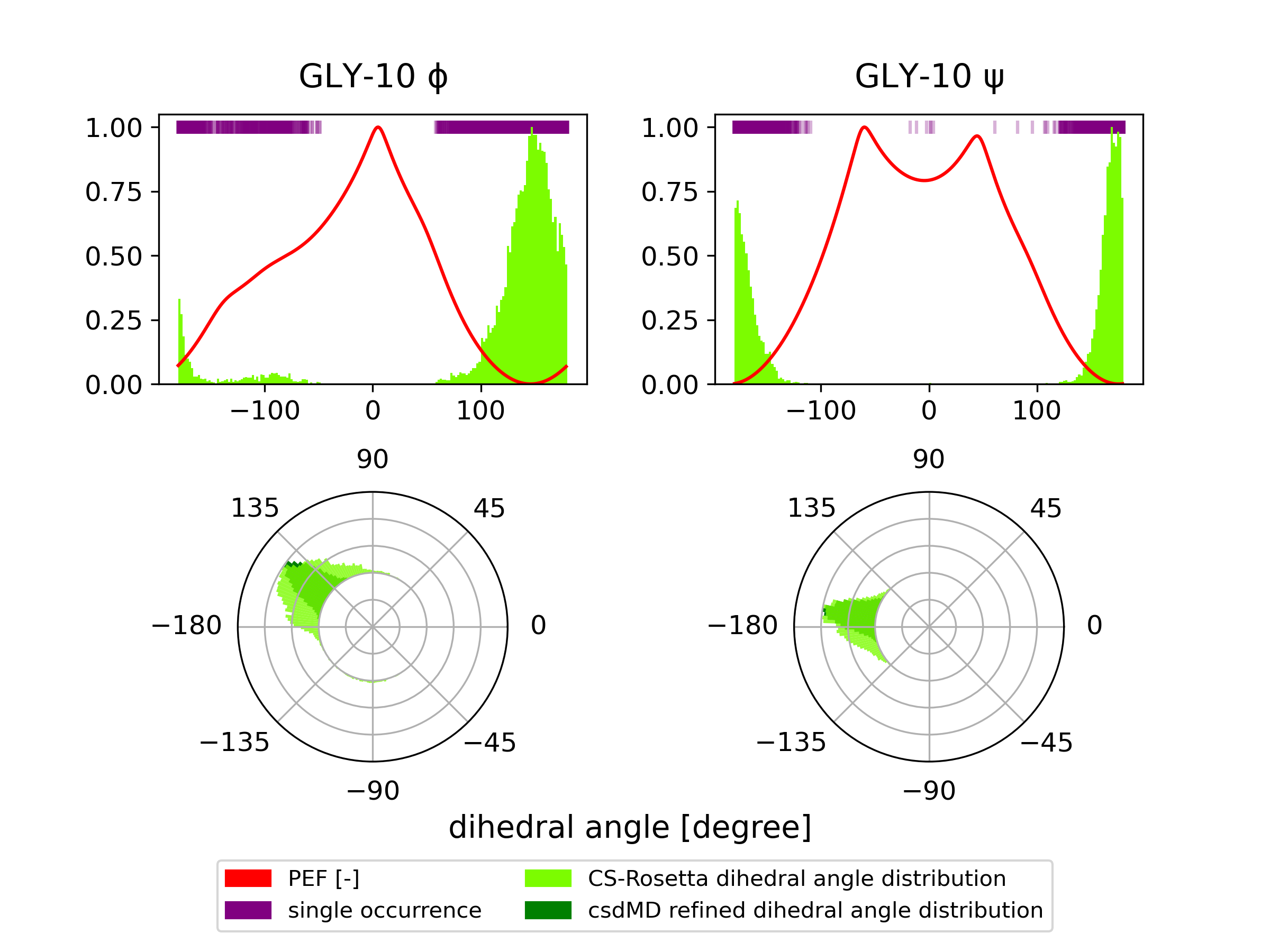

Supplement: Supplementary file 1 [file ijms-24-12101-s001.zip › KRAS-G12C-GDP-Mg_angle_figures/10-GLY.png]

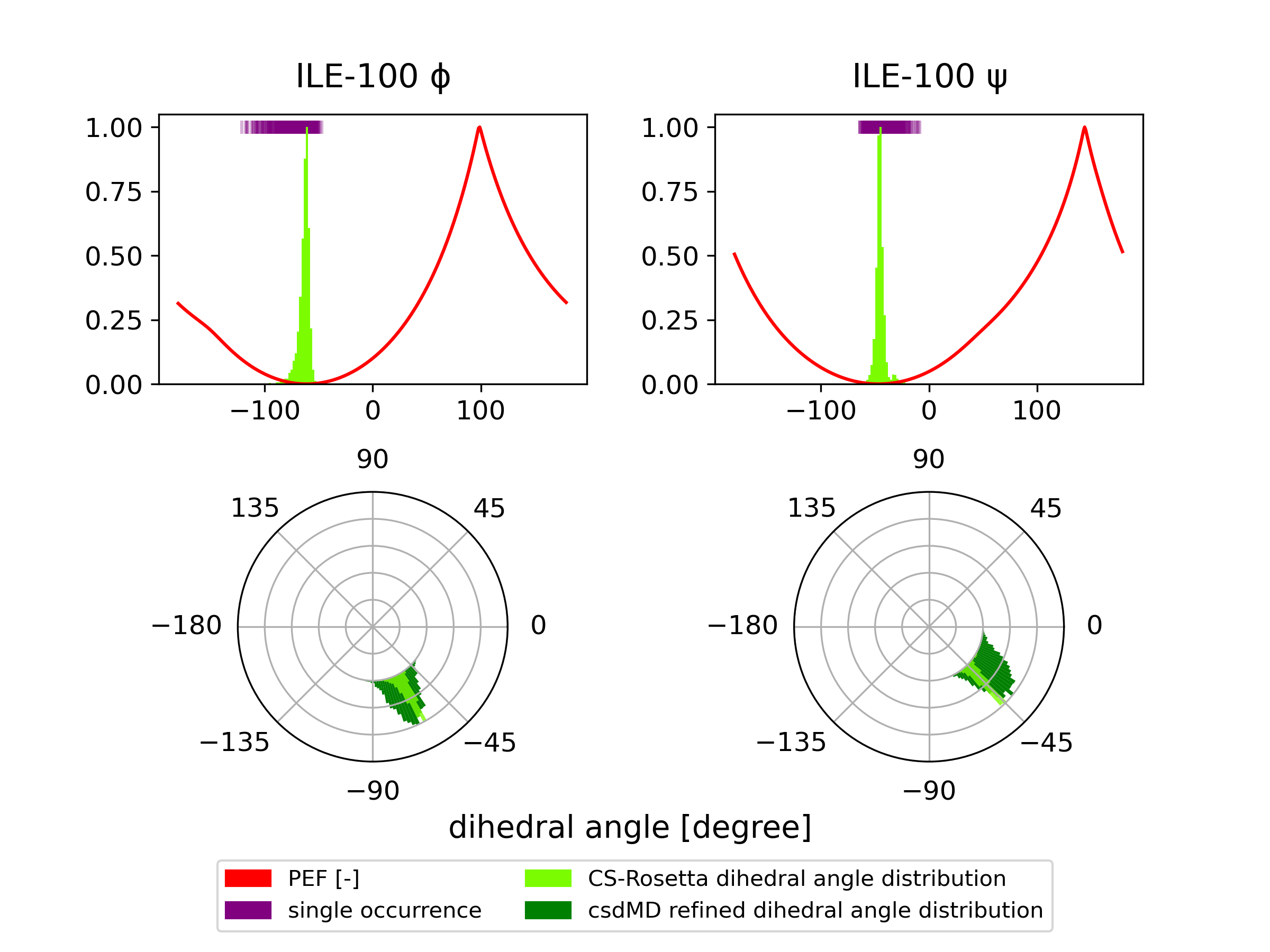

Supplement: Supplementary file 1 [file ijms-24-12101-s001.zip › KRAS-G12C-GDP-Mg_angle_figures/100-ILE.png]

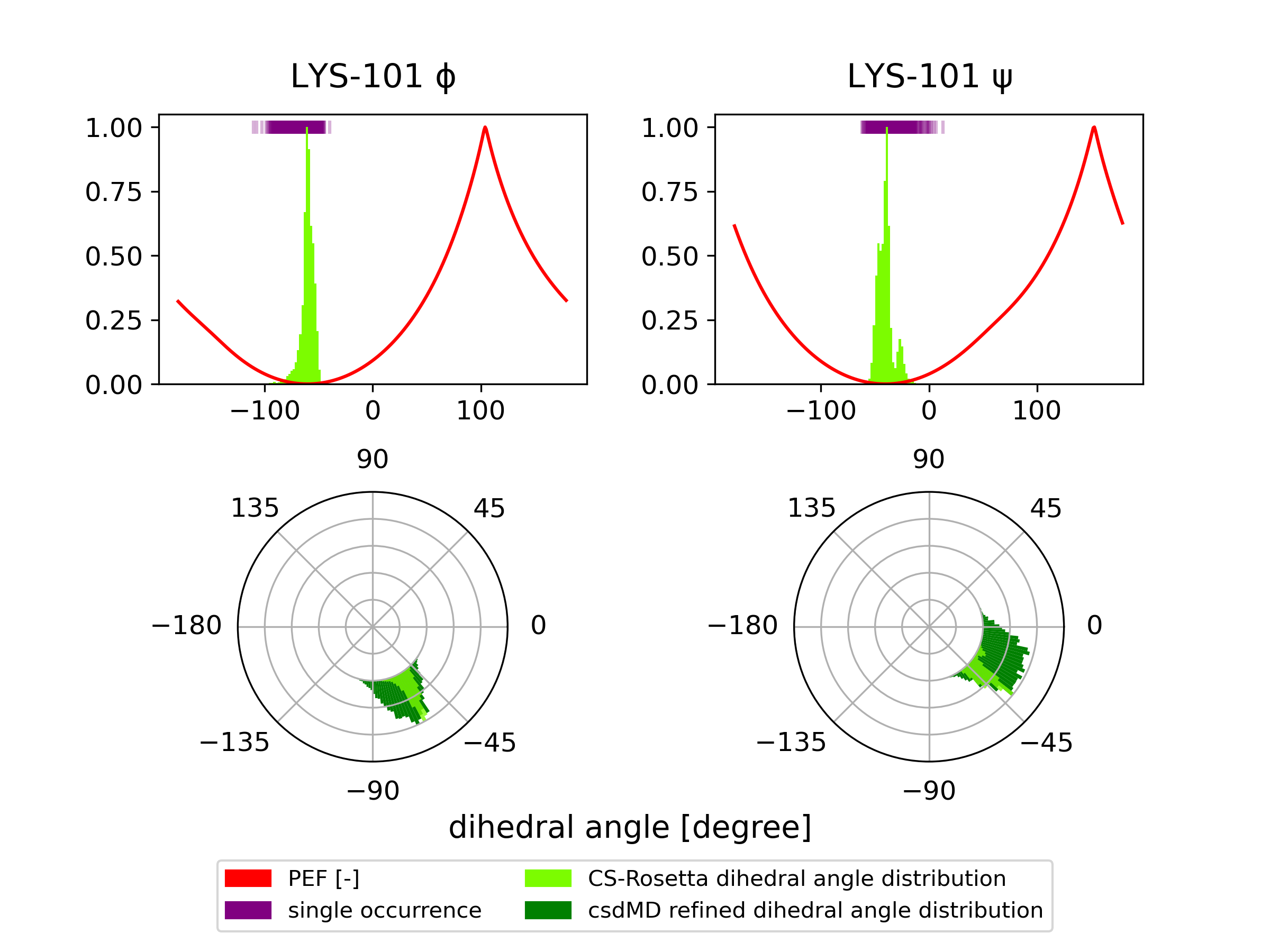

Supplement: Supplementary file 1 [file ijms-24-12101-s001.zip › KRAS-G12C-GDP-Mg_angle_figures/101-LYS.png]

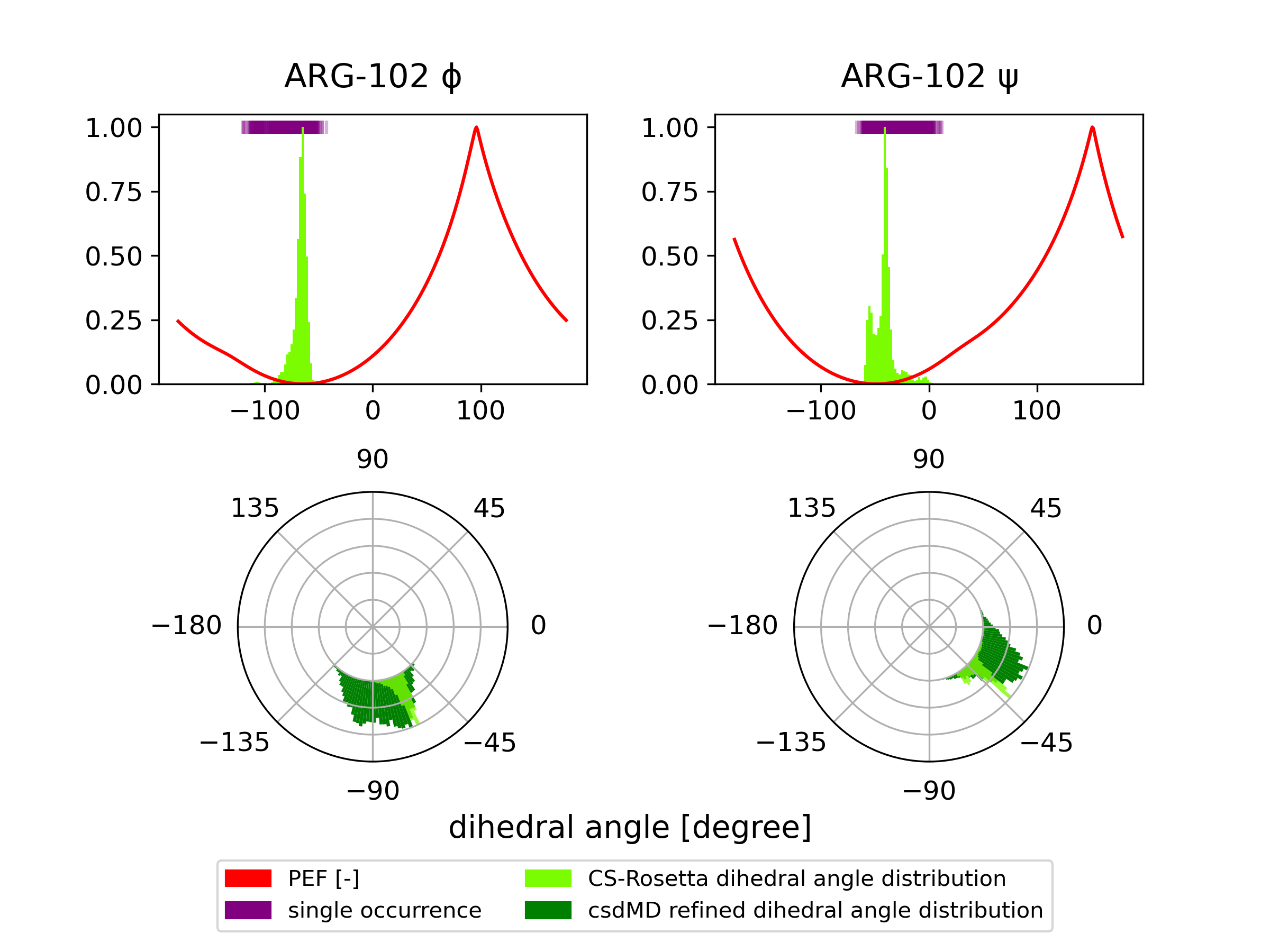

Supplement: Supplementary file 1 [file ijms-24-12101-s001.zip › KRAS-G12C-GDP-Mg_angle_figures/102-ARG.png]

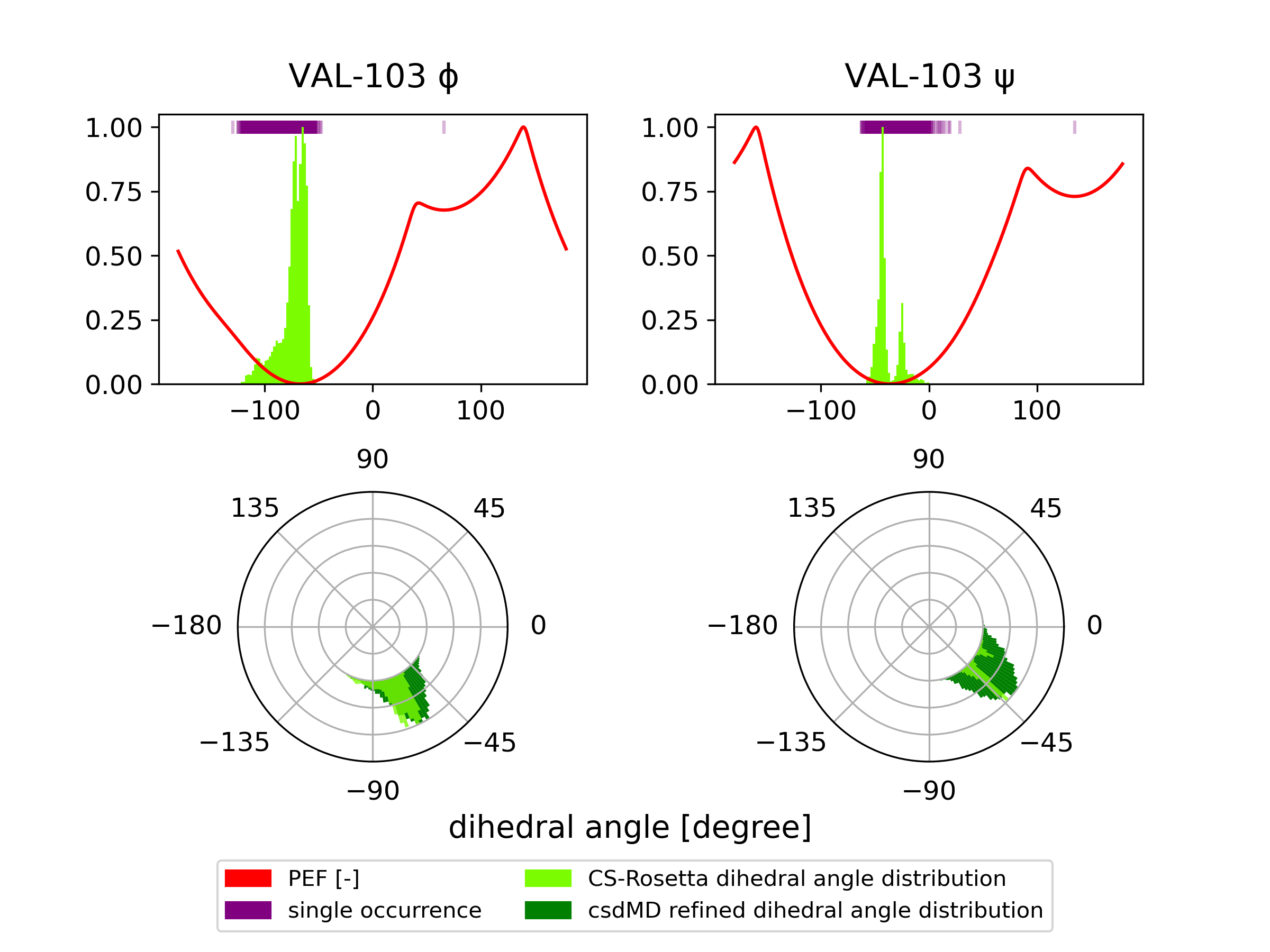

Supplement: Supplementary file 1 [file ijms-24-12101-s001.zip › KRAS-G12C-GDP-Mg_angle_figures/103-VAL.png]

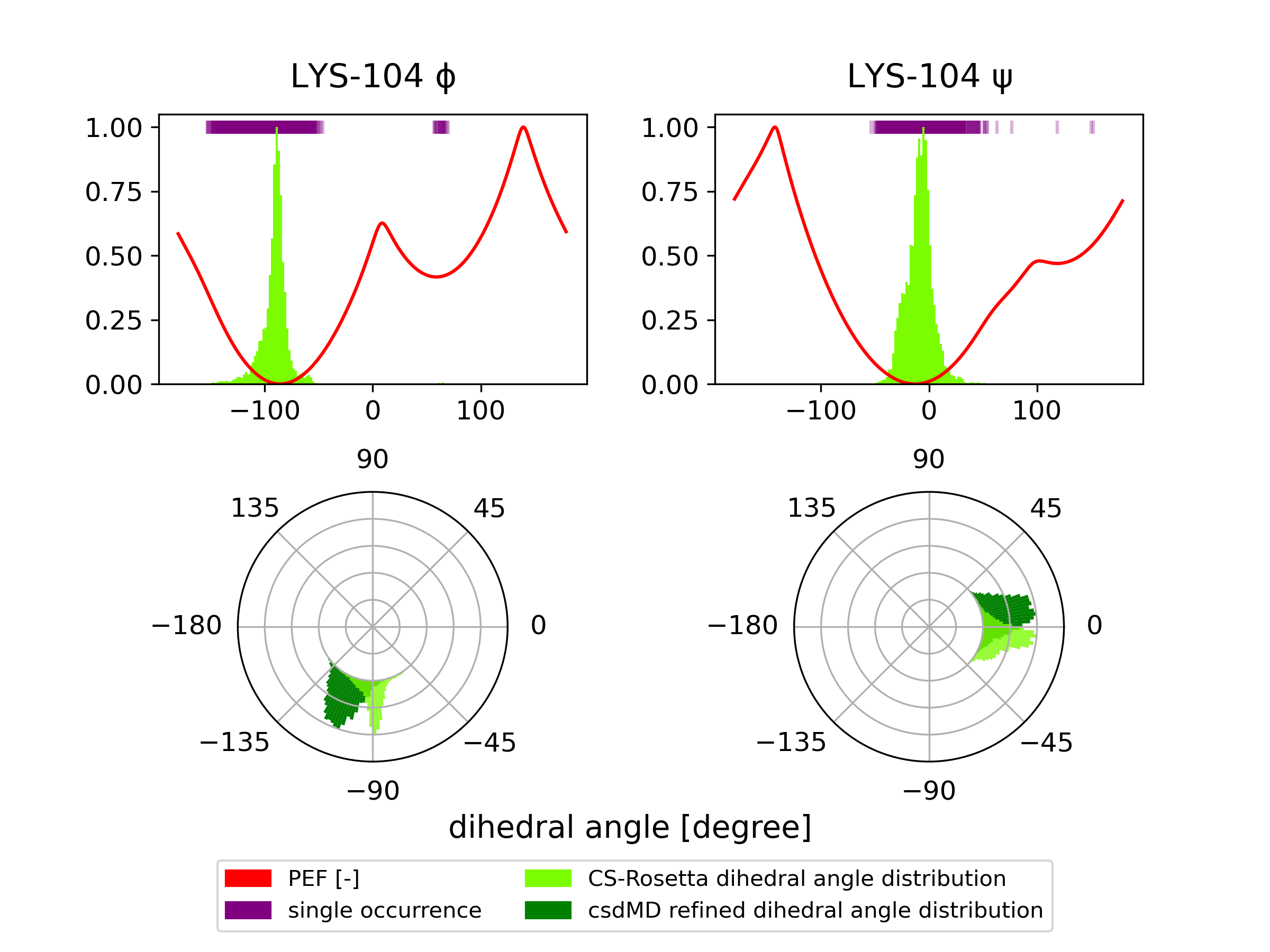

Supplement: Supplementary file 1 [file ijms-24-12101-s001.zip › KRAS-G12C-GDP-Mg_angle_figures/104-LYS.png]

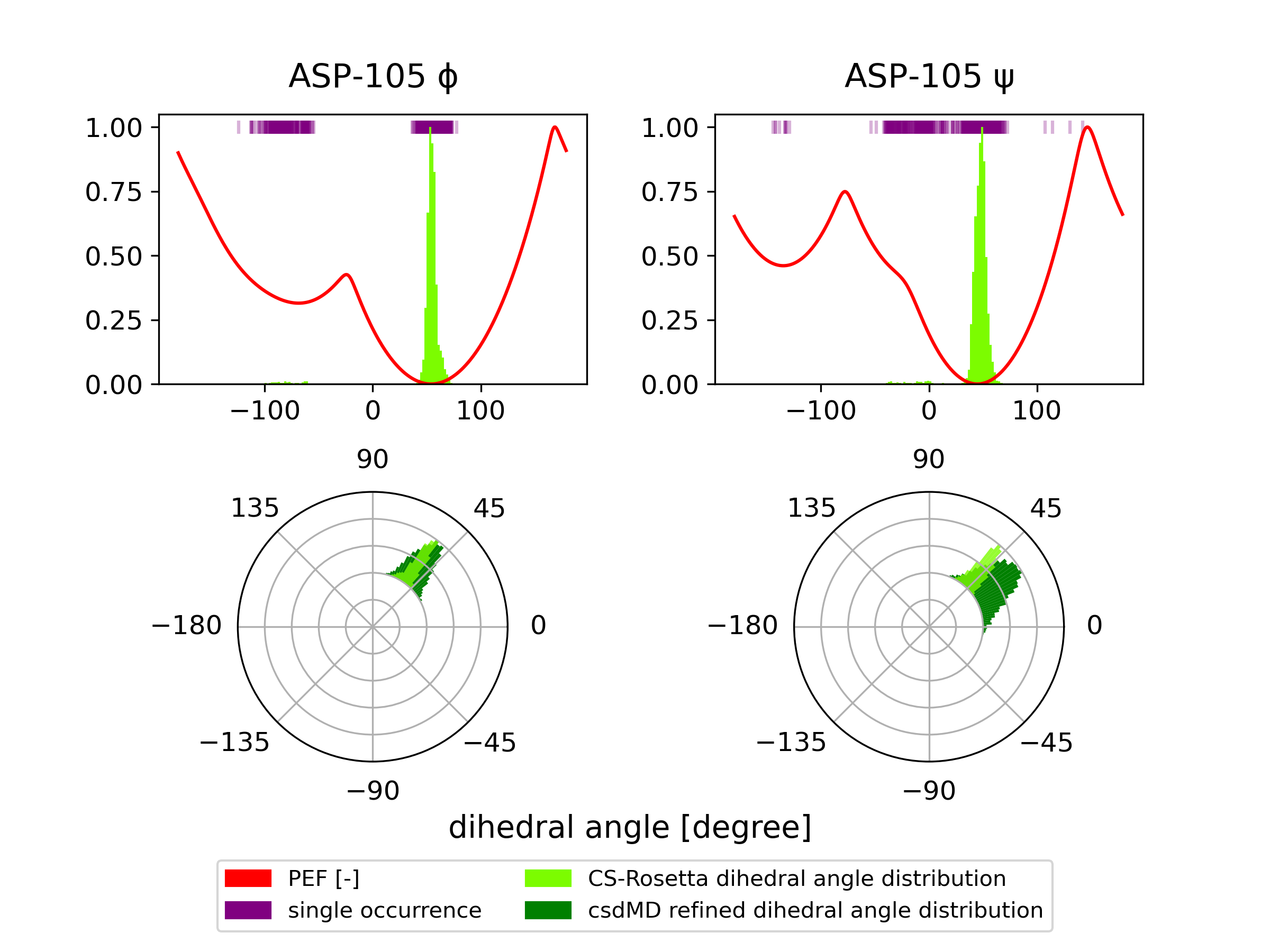

Supplement: Supplementary file 1 [file ijms-24-12101-s001.zip › KRAS-G12C-GDP-Mg_angle_figures/105-ASP.png]

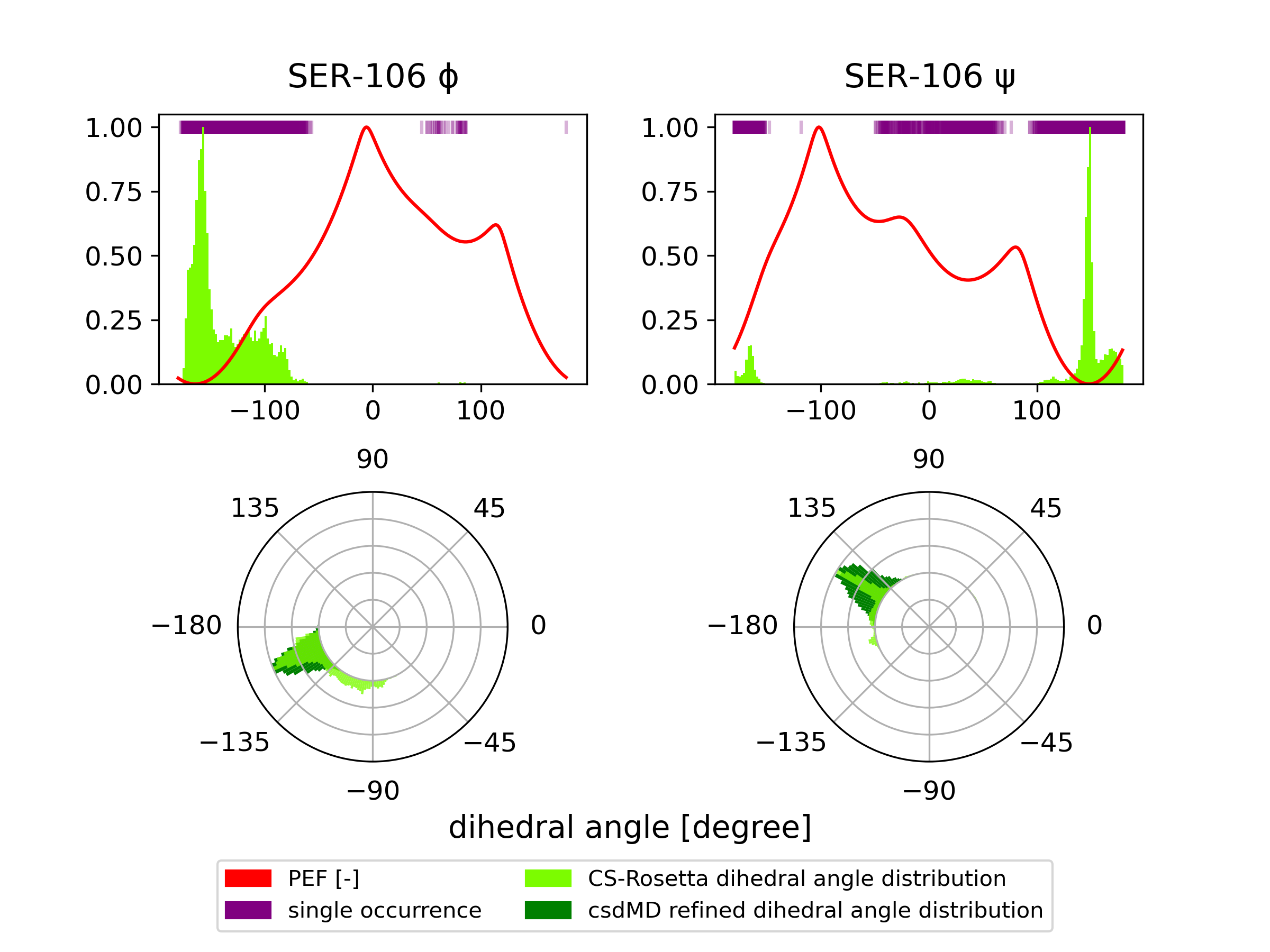

Supplement: Supplementary file 1 [file ijms-24-12101-s001.zip › KRAS-G12C-GDP-Mg_angle_figures/106-SER.png]

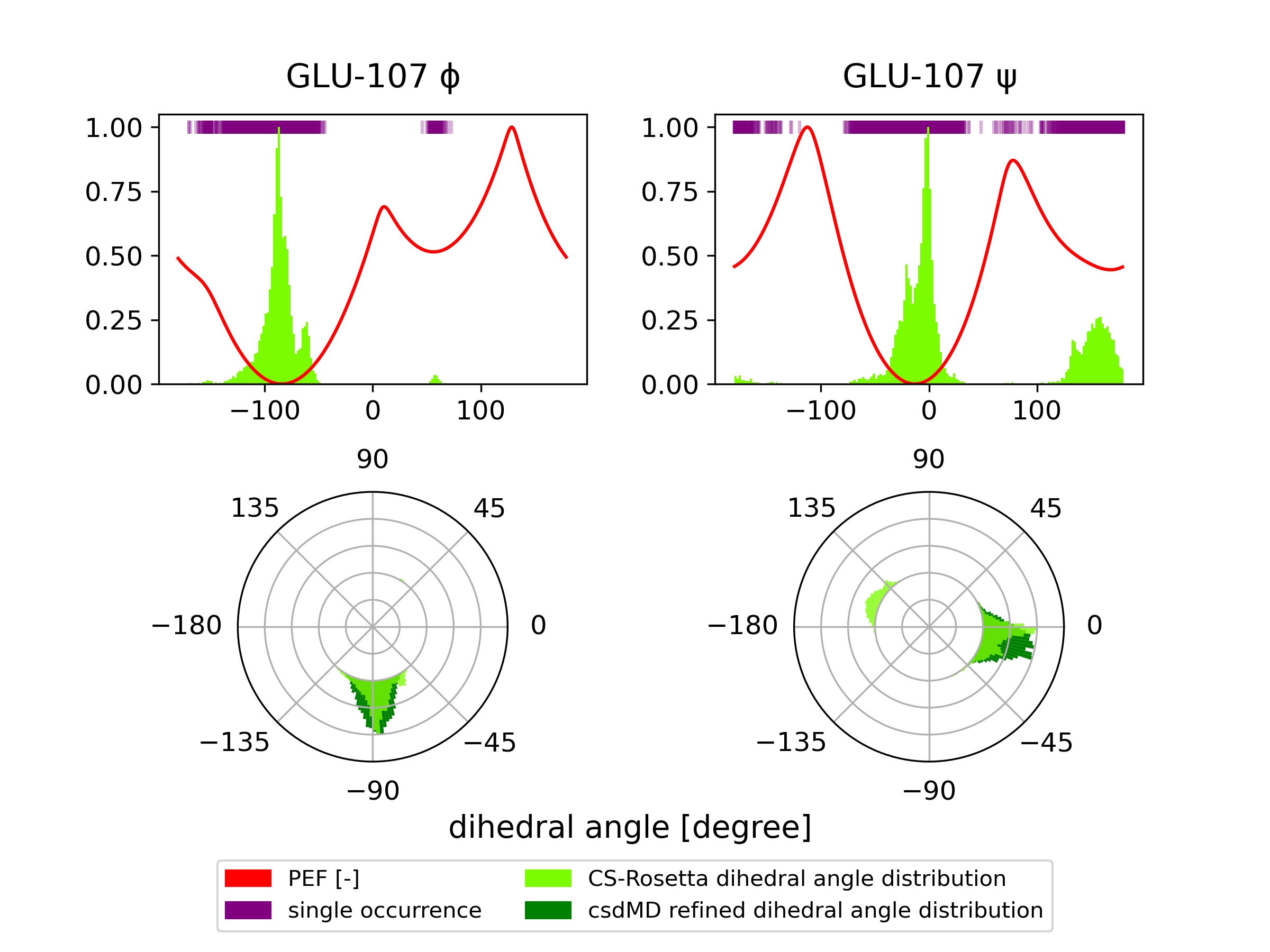

Supplement: Supplementary file 1 [file ijms-24-12101-s001.zip › KRAS-G12C-GDP-Mg_angle_figures/107-GLU.png]

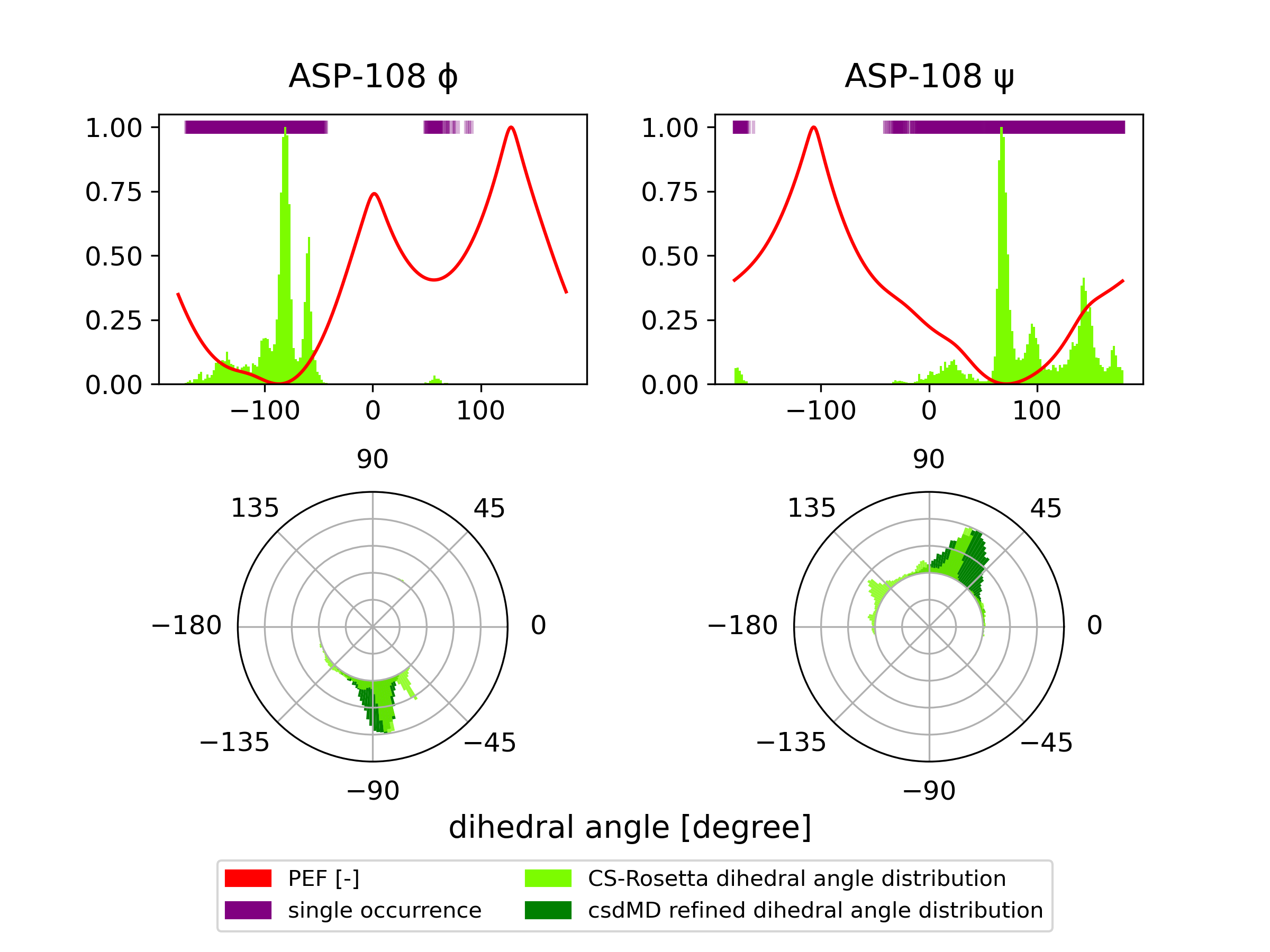

Supplement: Supplementary file 1 [file ijms-24-12101-s001.zip › KRAS-G12C-GDP-Mg_angle_figures/108-ASP.png]

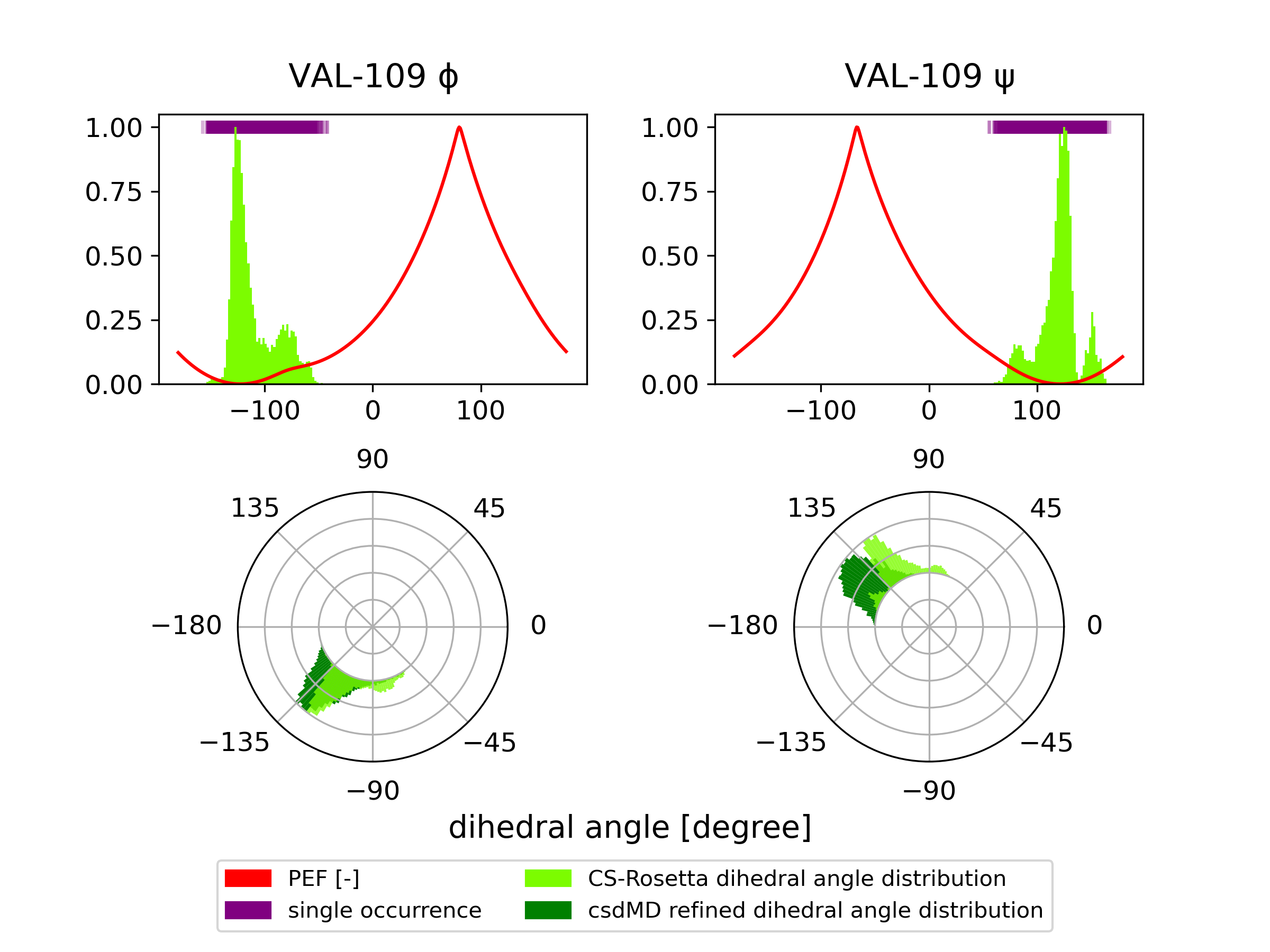

Supplement: Supplementary file 1 [file ijms-24-12101-s001.zip › KRAS-G12C-GDP-Mg_angle_figures/109-VAL.png]

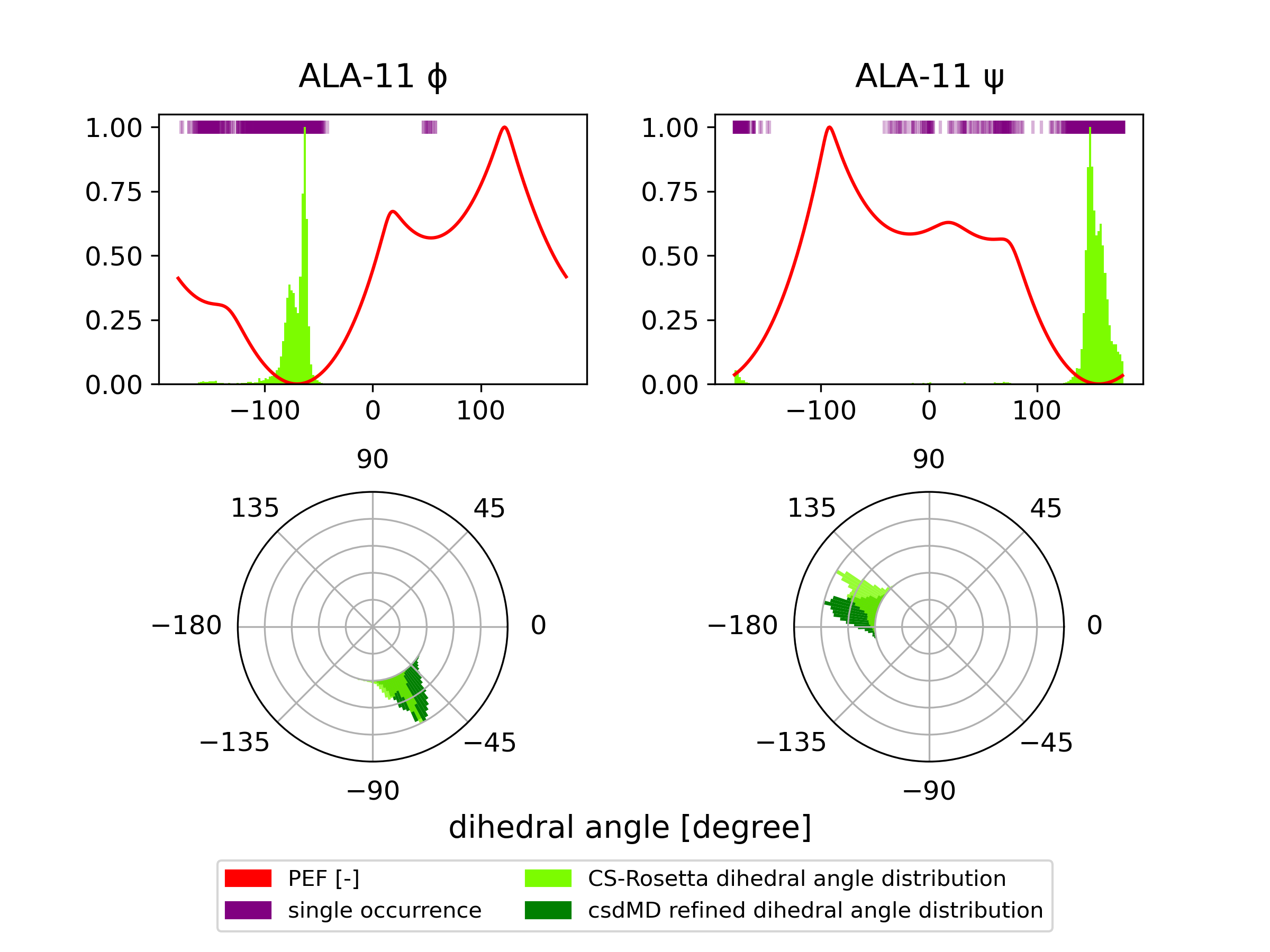

Supplement: Supplementary file 1 [file ijms-24-12101-s001.zip › KRAS-G12C-GDP-Mg_angle_figures/11-ALA.png]

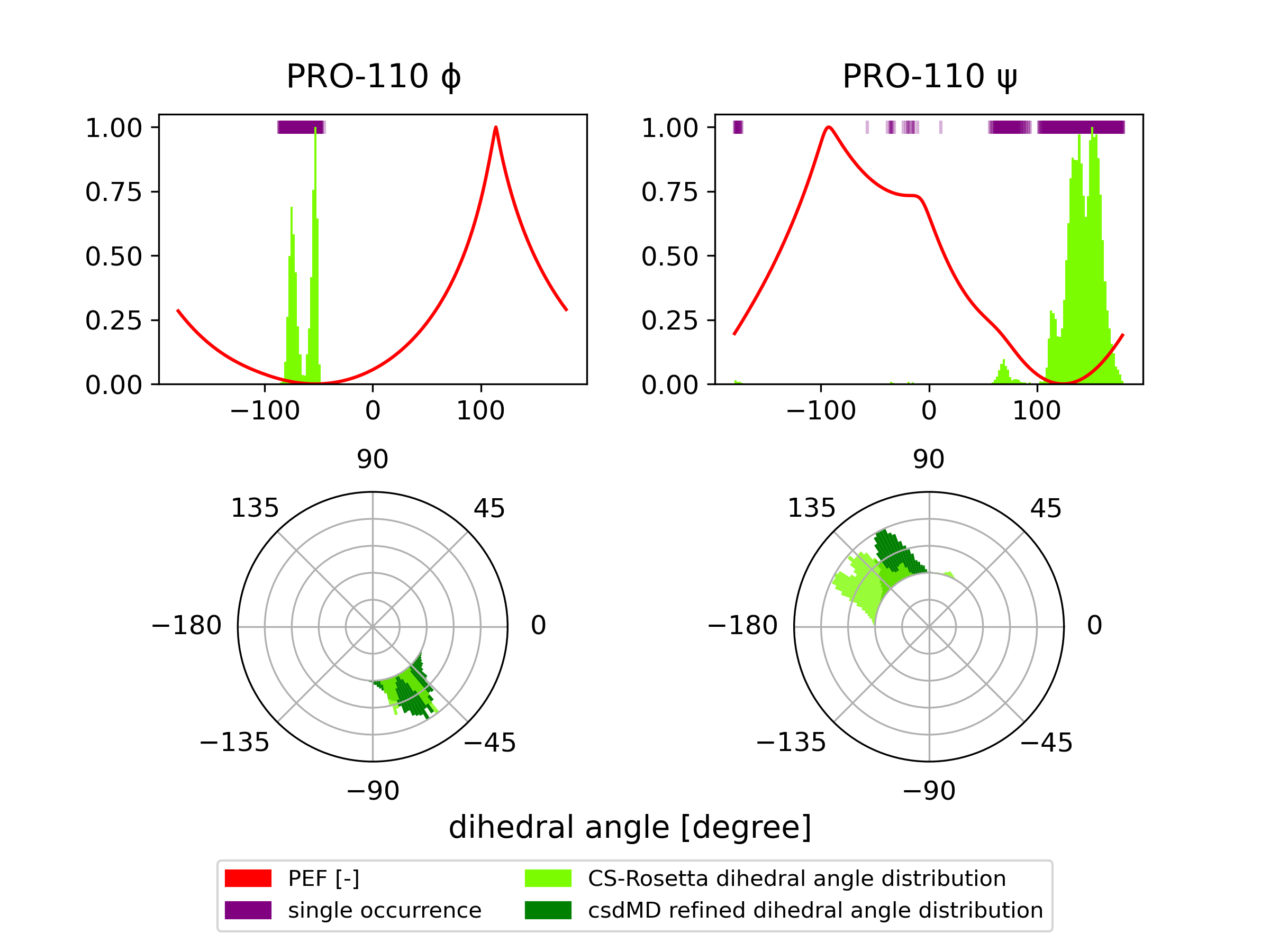

Supplement: Supplementary file 1 [file ijms-24-12101-s001.zip › KRAS-G12C-GDP-Mg_angle_figures/110-PRO.png]

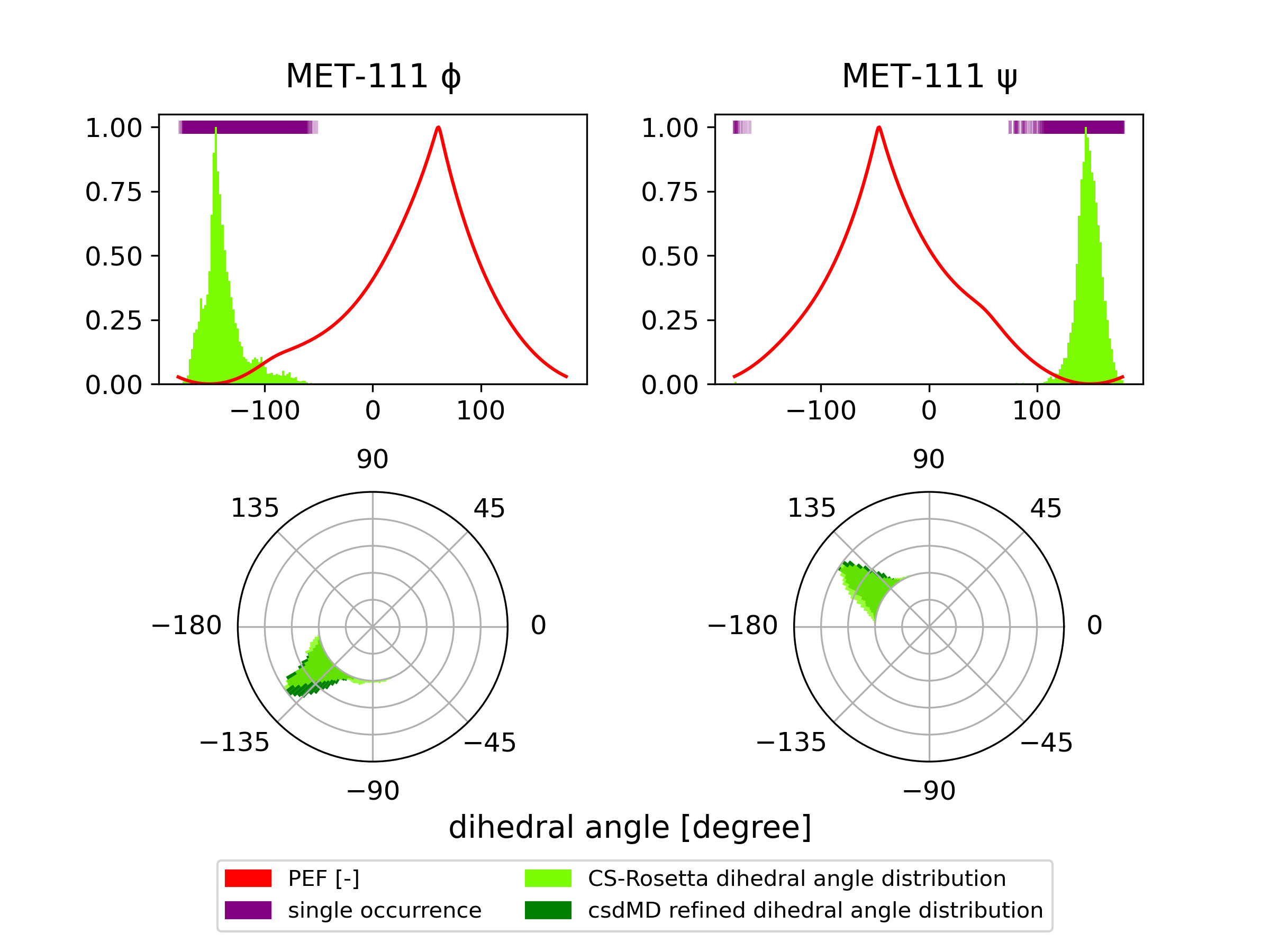

Supplement: Supplementary file 1 [file ijms-24-12101-s001.zip › KRAS-G12C-GDP-Mg_angle_figures/111-MET.png]

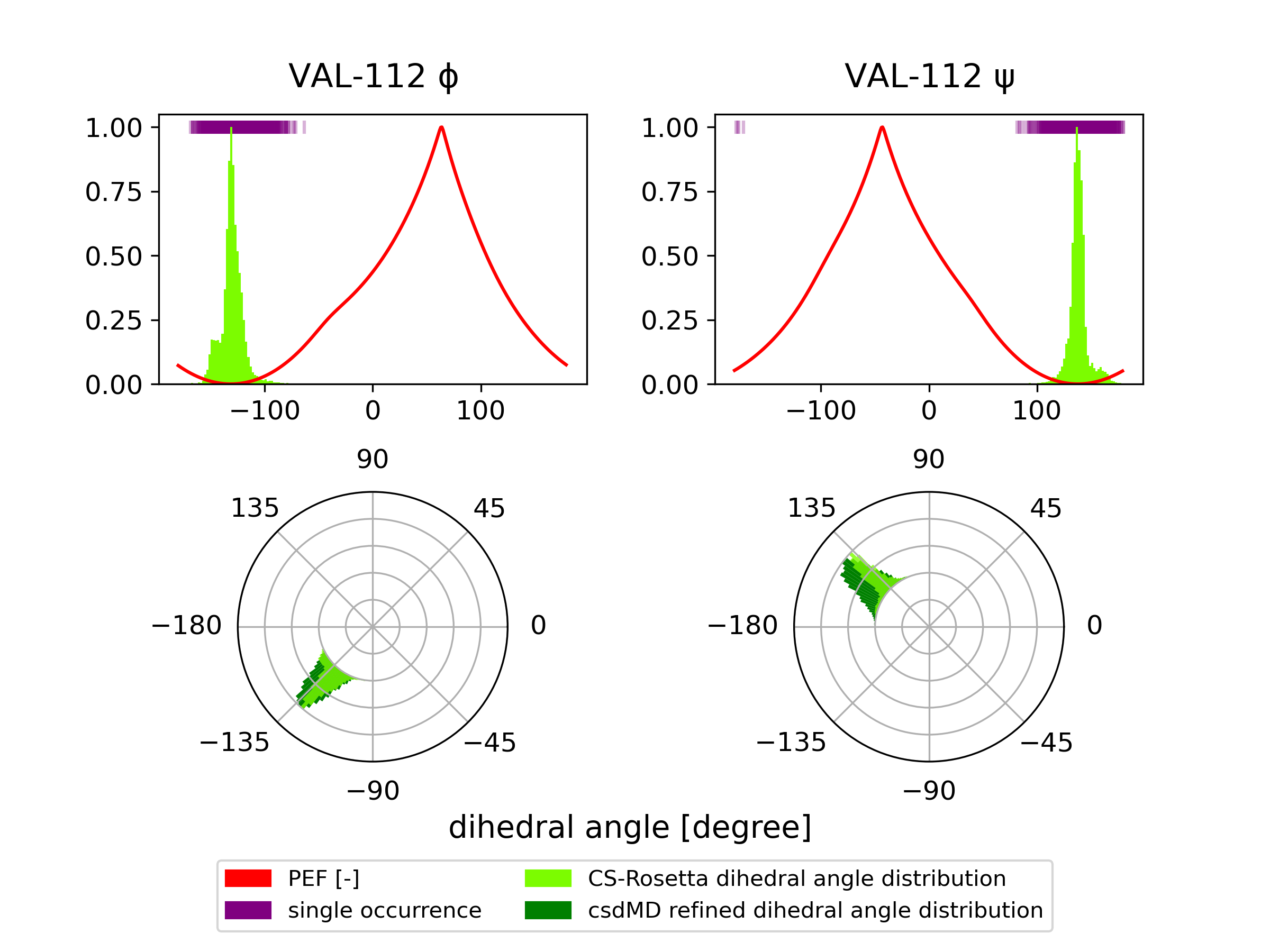

Supplement: Supplementary file 1 [file ijms-24-12101-s001.zip › KRAS-G12C-GDP-Mg_angle_figures/112-VAL.png]

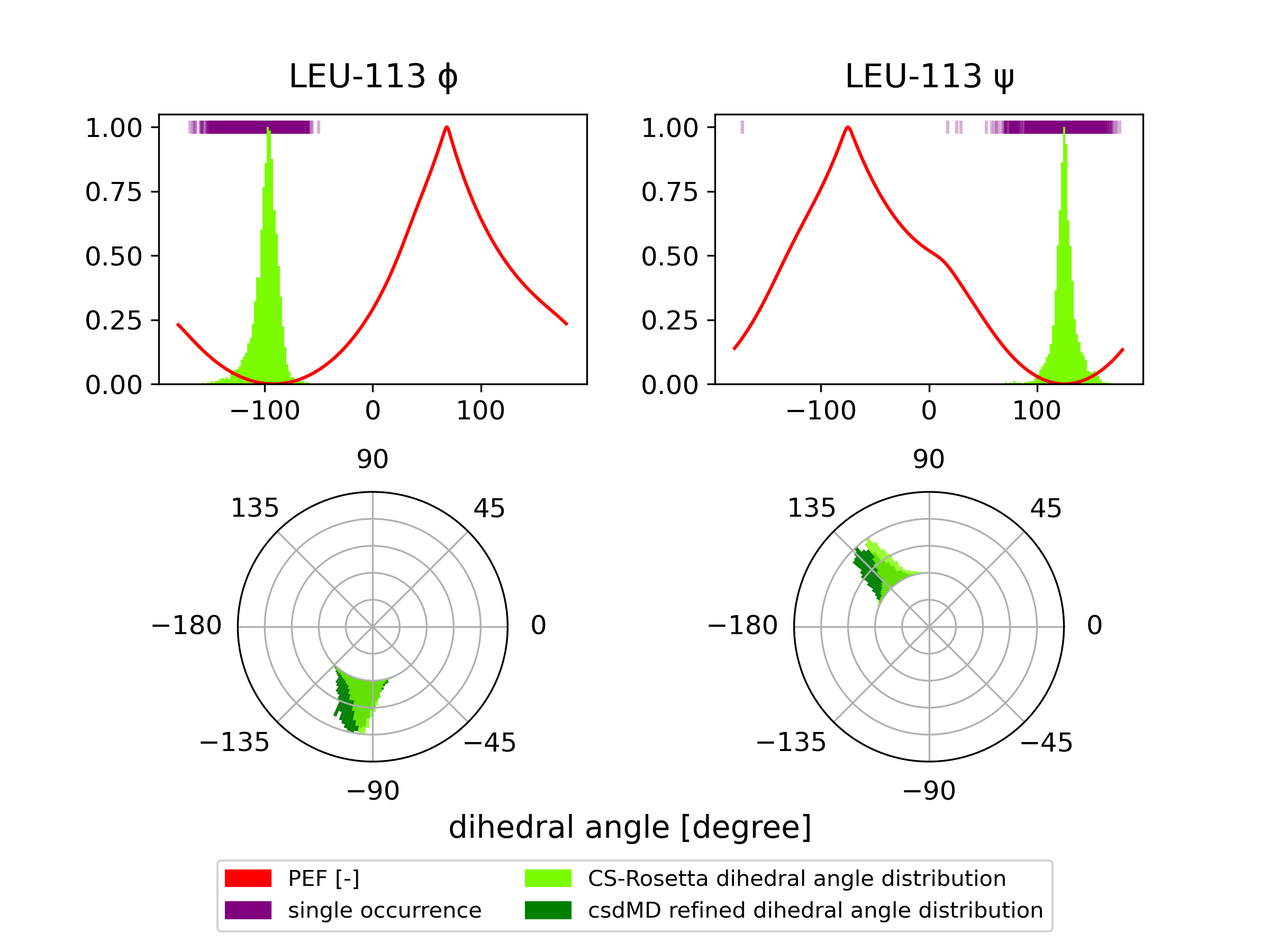

Supplement: Supplementary file 1 [file ijms-24-12101-s001.zip › KRAS-G12C-GDP-Mg_angle_figures/113-LEU.png]

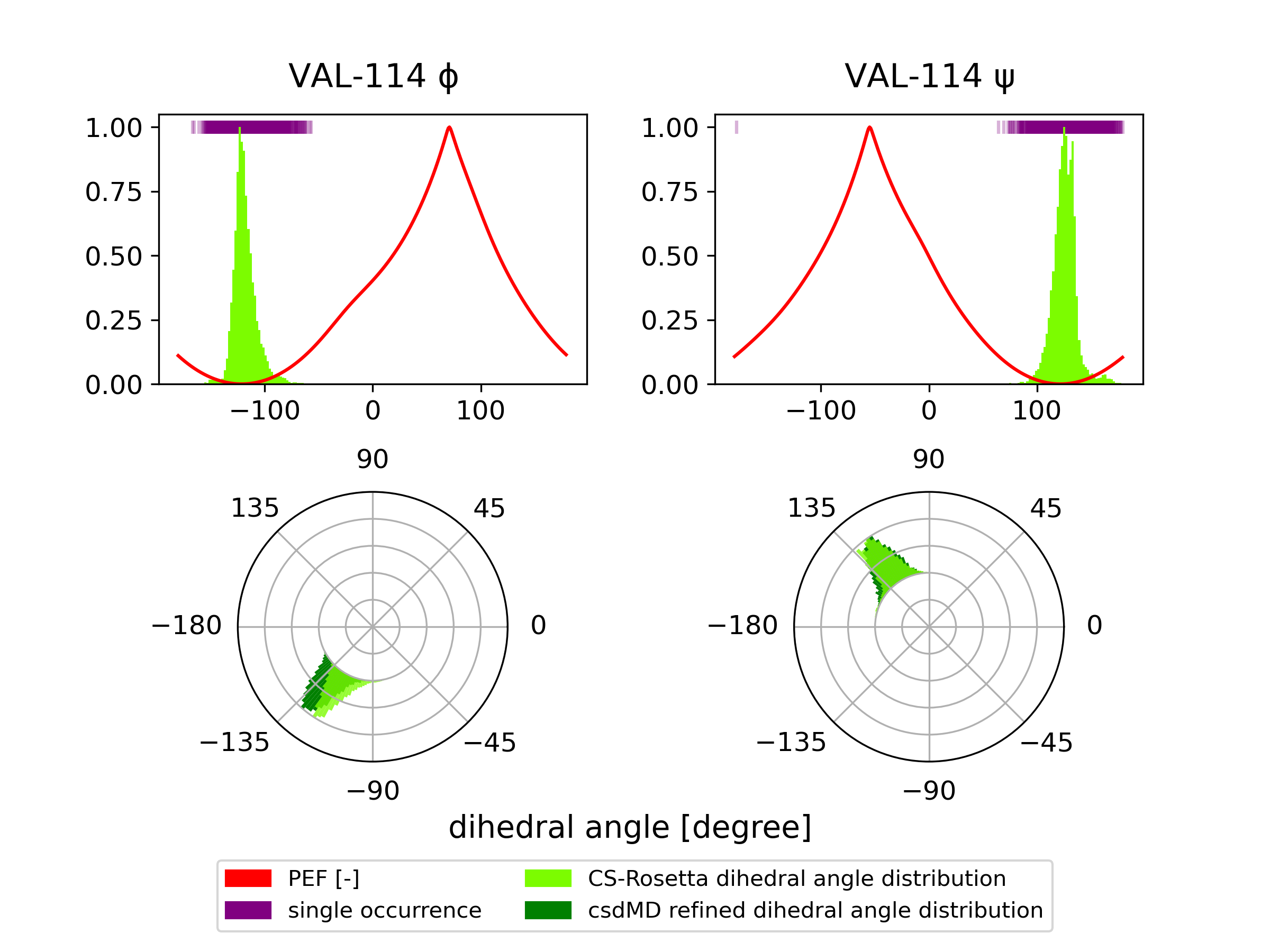

Supplement: Supplementary file 1 [file ijms-24-12101-s001.zip › KRAS-G12C-GDP-Mg_angle_figures/114-VAL.png]

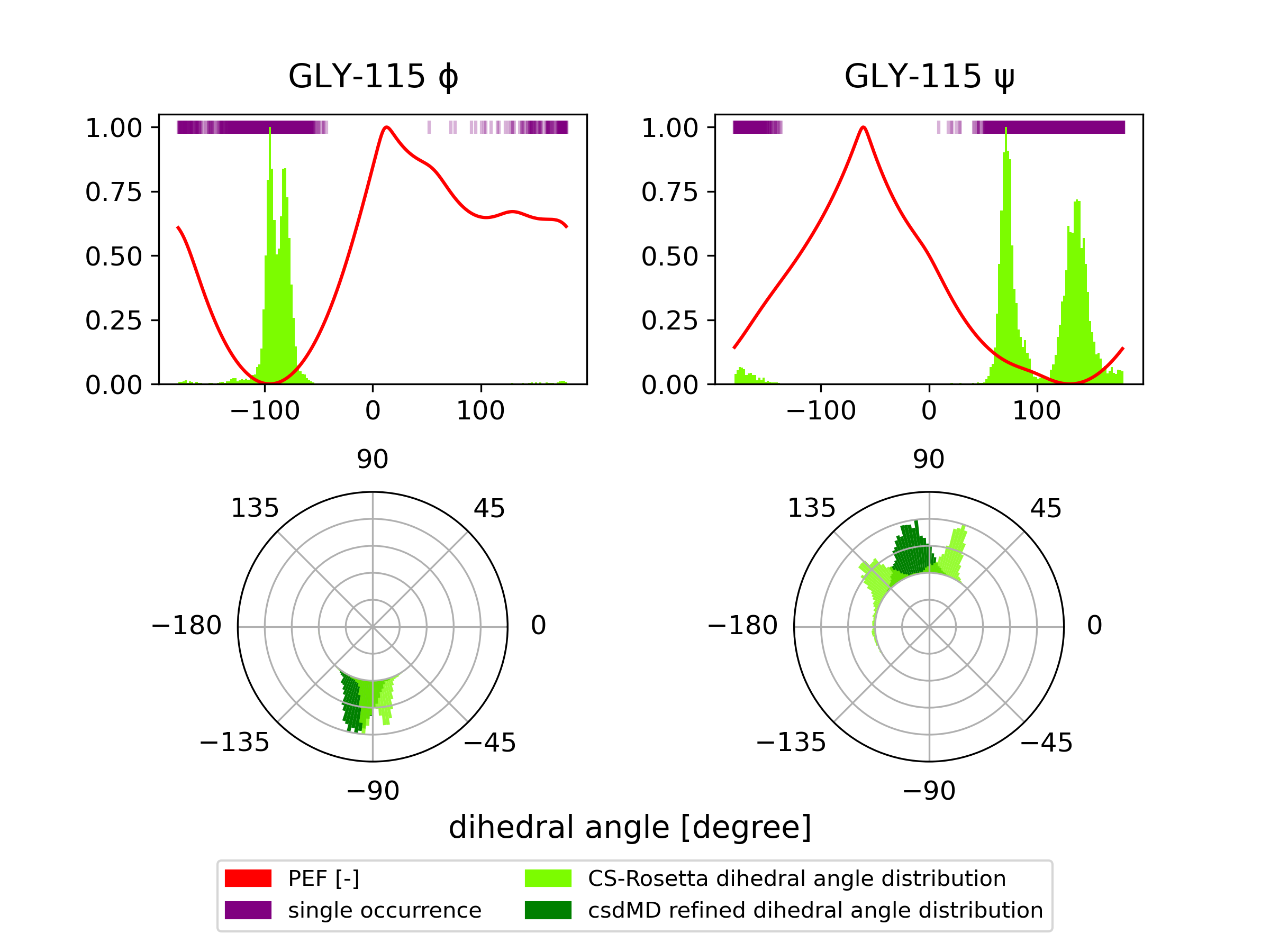

Supplement: Supplementary file 1 [file ijms-24-12101-s001.zip › KRAS-G12C-GDP-Mg_angle_figures/115-GLY.png]

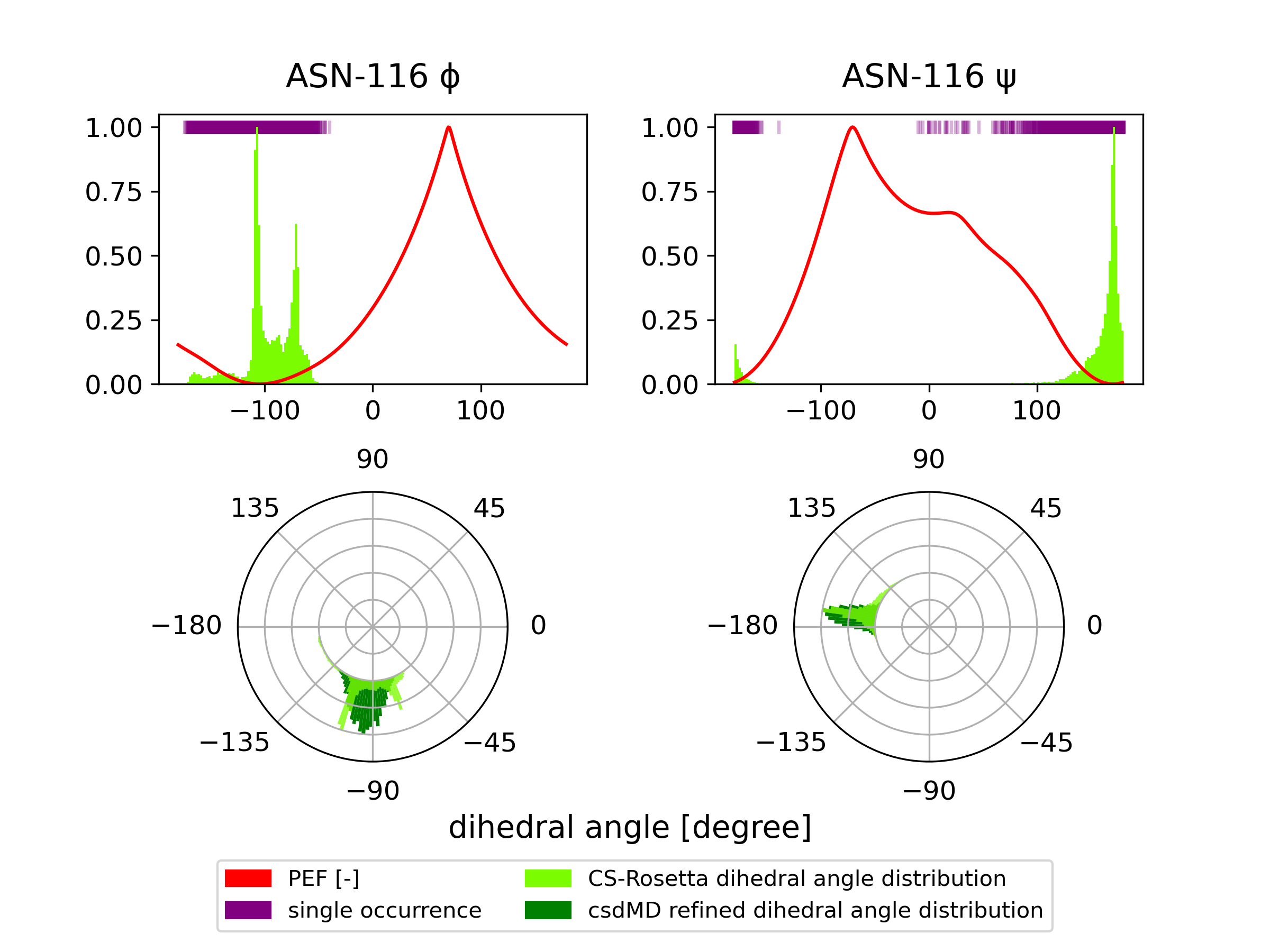

Supplement: Supplementary file 1 [file ijms-24-12101-s001.zip › KRAS-G12C-GDP-Mg_angle_figures/116-ASN.png]

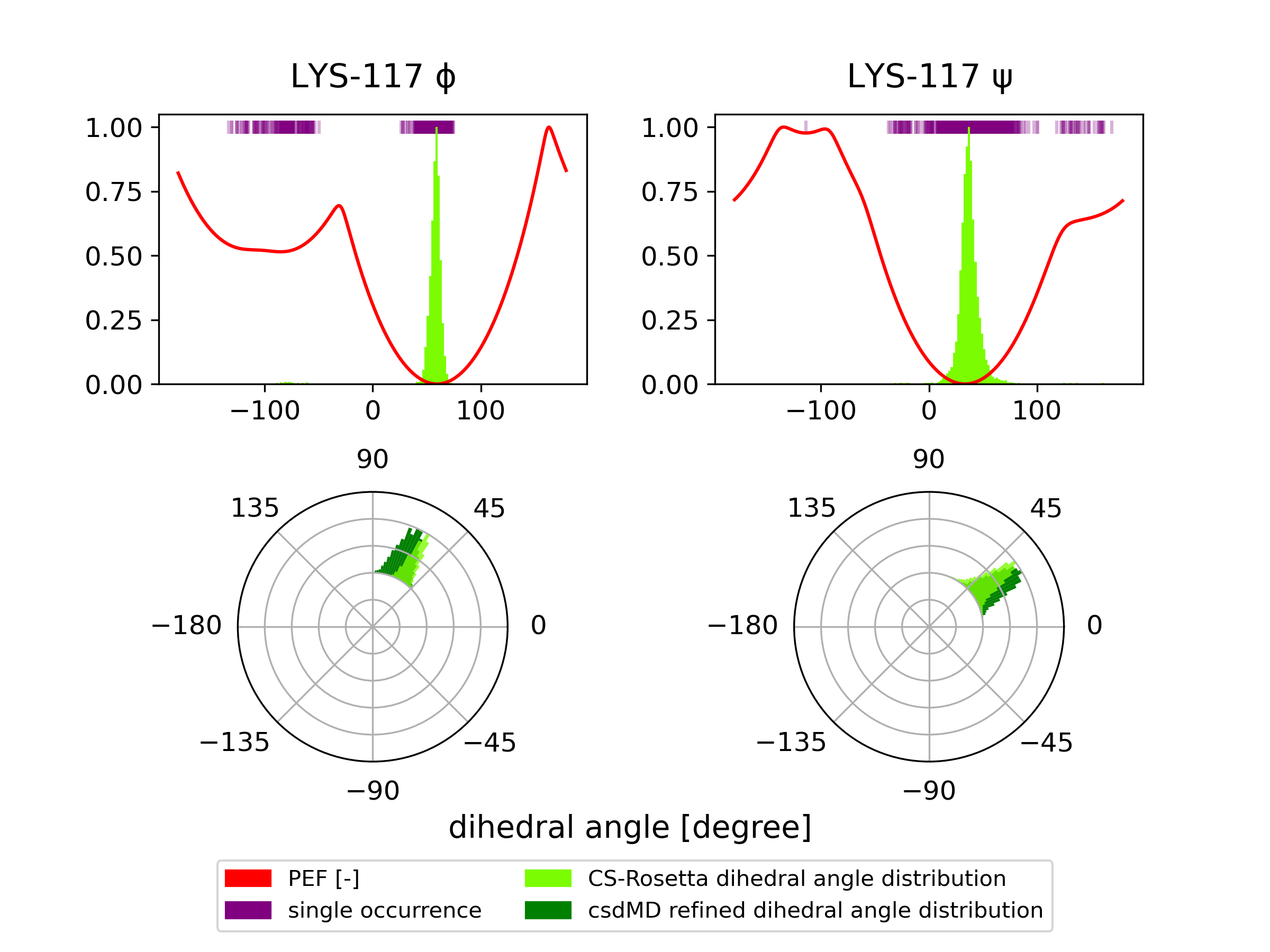

Supplement: Supplementary file 1 [file ijms-24-12101-s001.zip › KRAS-G12C-GDP-Mg_angle_figures/117-LYS.png]

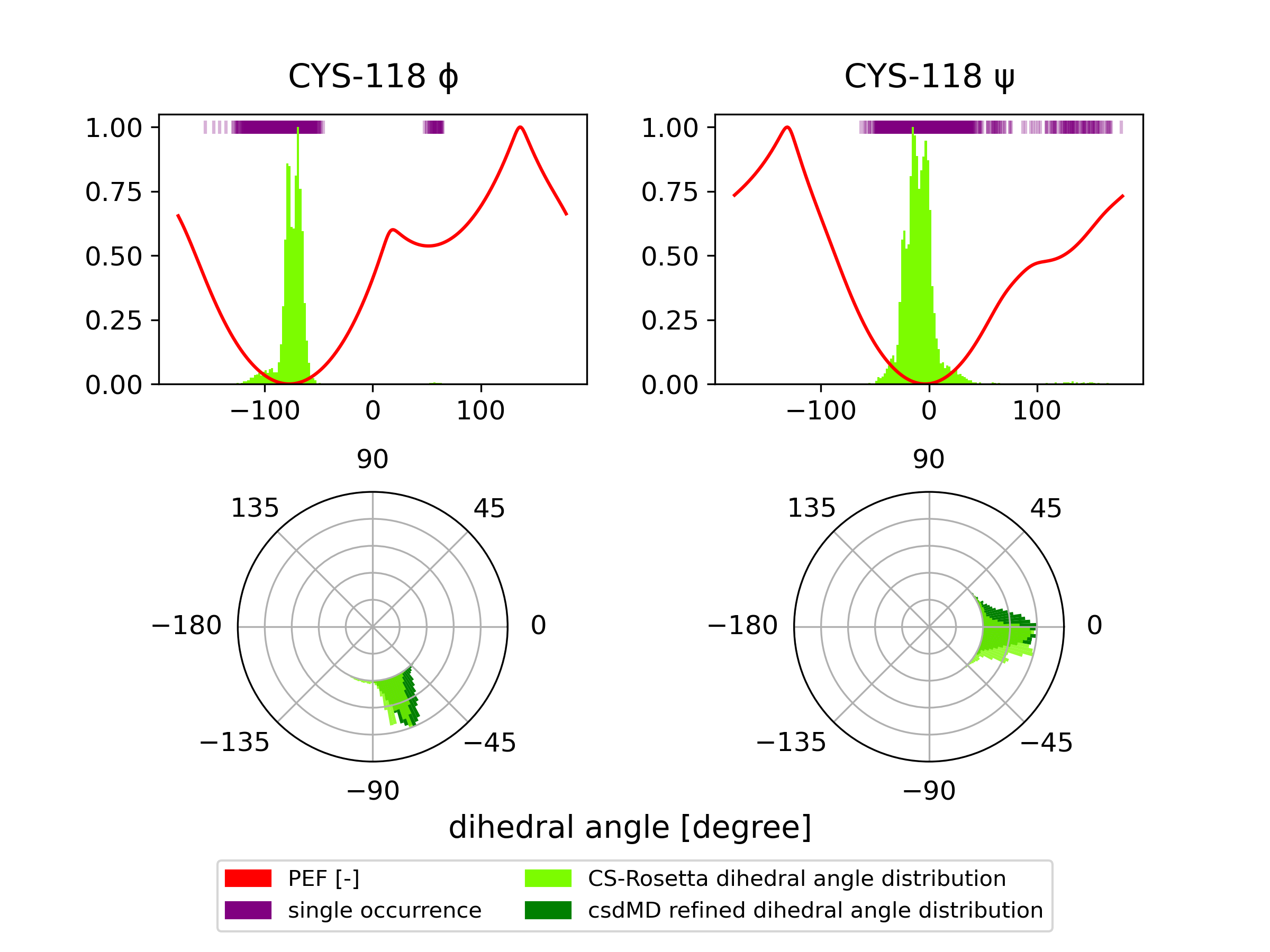

Supplement: Supplementary file 1 [file ijms-24-12101-s001.zip › KRAS-G12C-GDP-Mg_angle_figures/118-CYS.png]

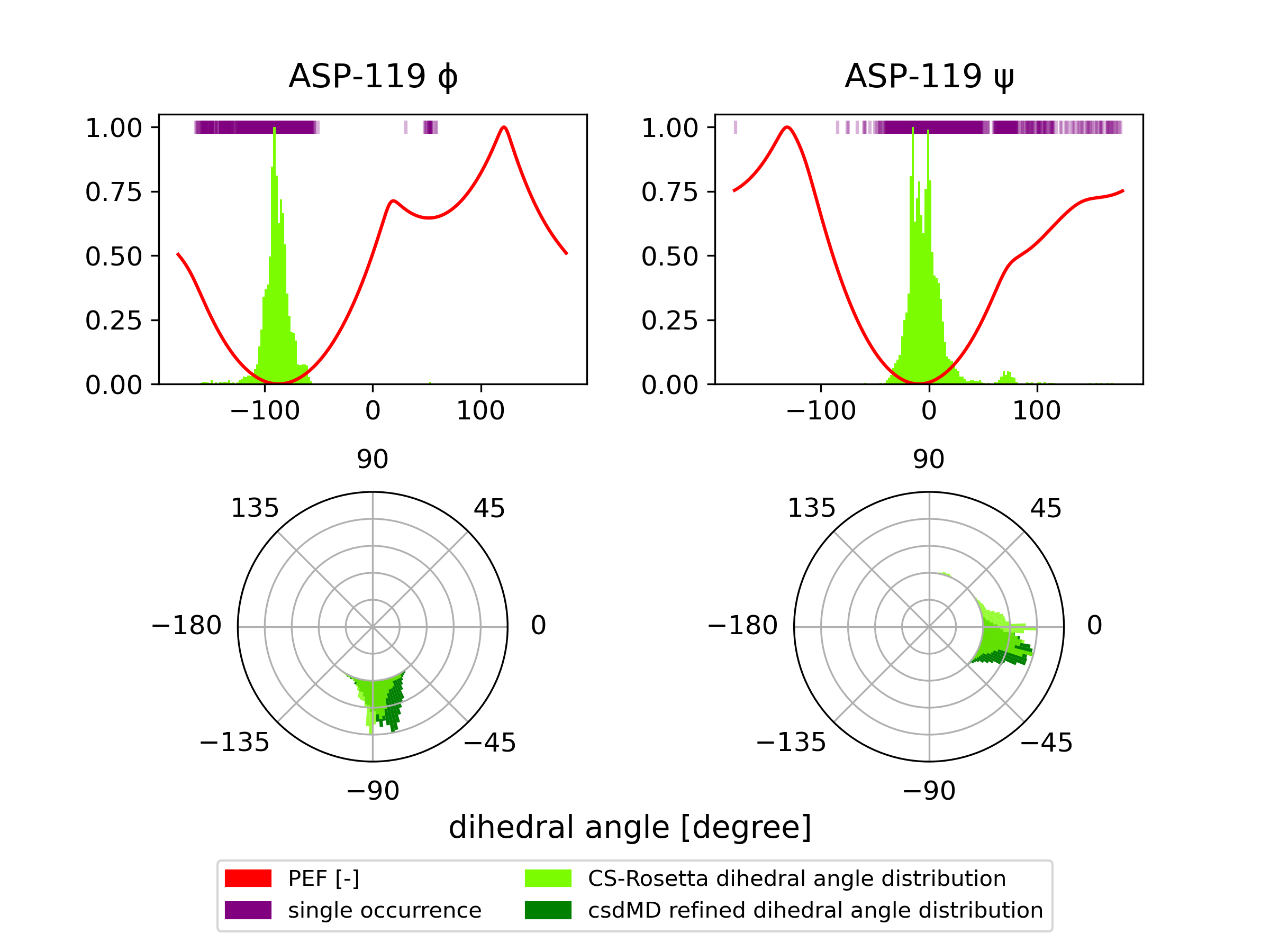

Supplement: Supplementary file 1 [file ijms-24-12101-s001.zip › KRAS-G12C-GDP-Mg_angle_figures/119-ASP.png]

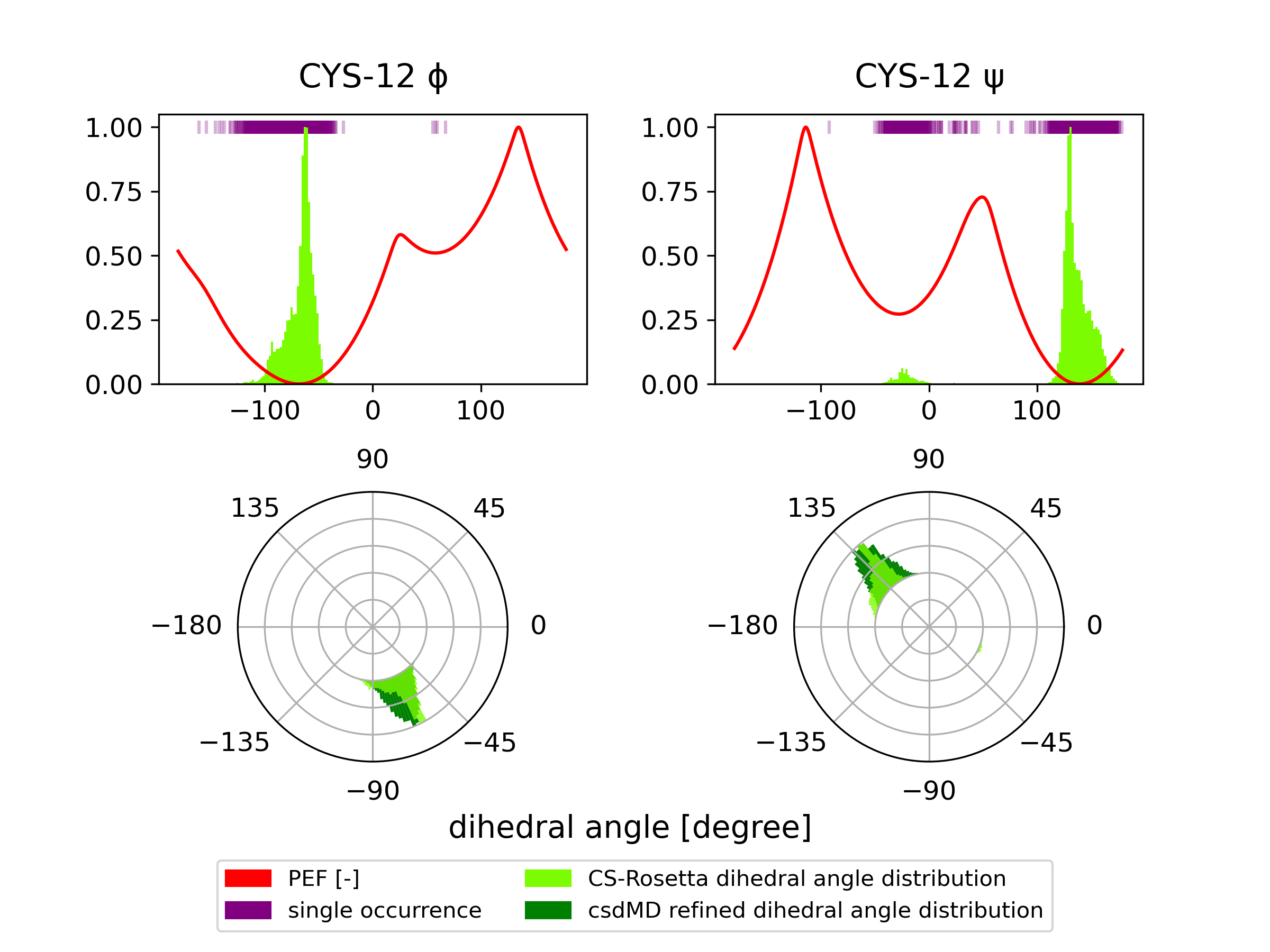

Supplement: Supplementary file 1 [file ijms-24-12101-s001.zip › KRAS-G12C-GDP-Mg_angle_figures/12-CYS.png]

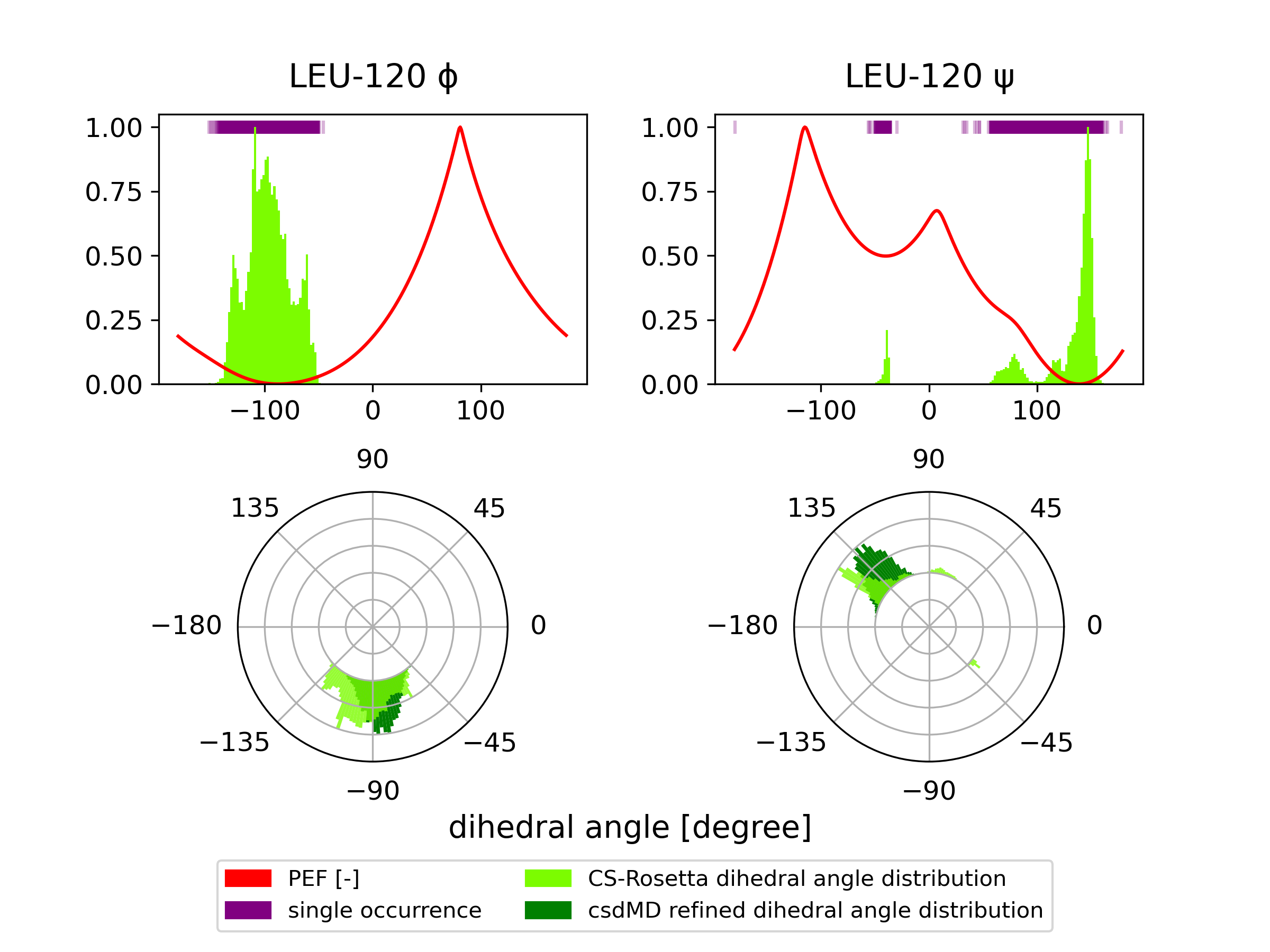

Supplement: Supplementary file 1 [file ijms-24-12101-s001.zip › KRAS-G12C-GDP-Mg_angle_figures/120-LEU.png]

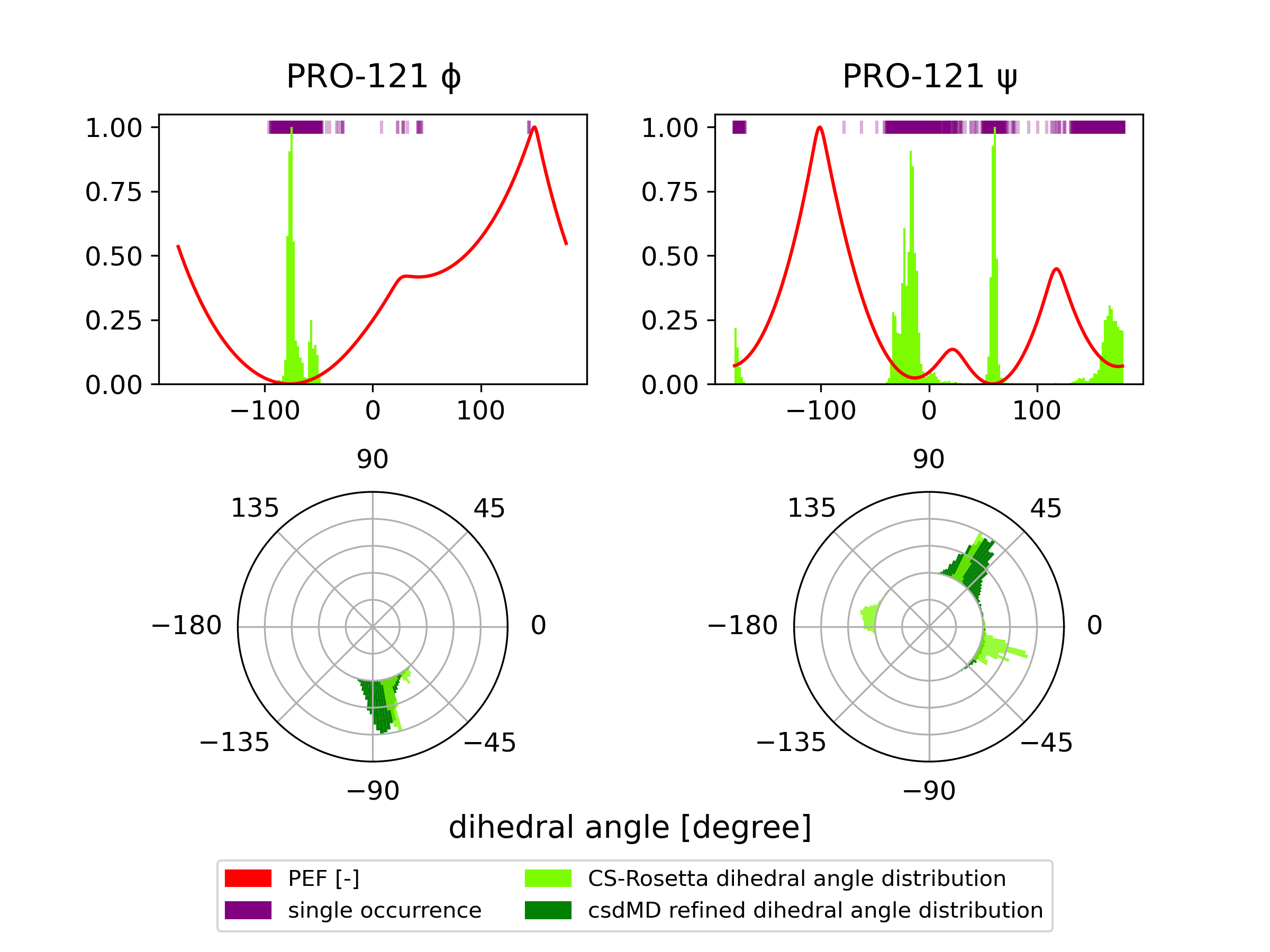

Supplement: Supplementary file 1 [file ijms-24-12101-s001.zip › KRAS-G12C-GDP-Mg_angle_figures/121-PRO.png]

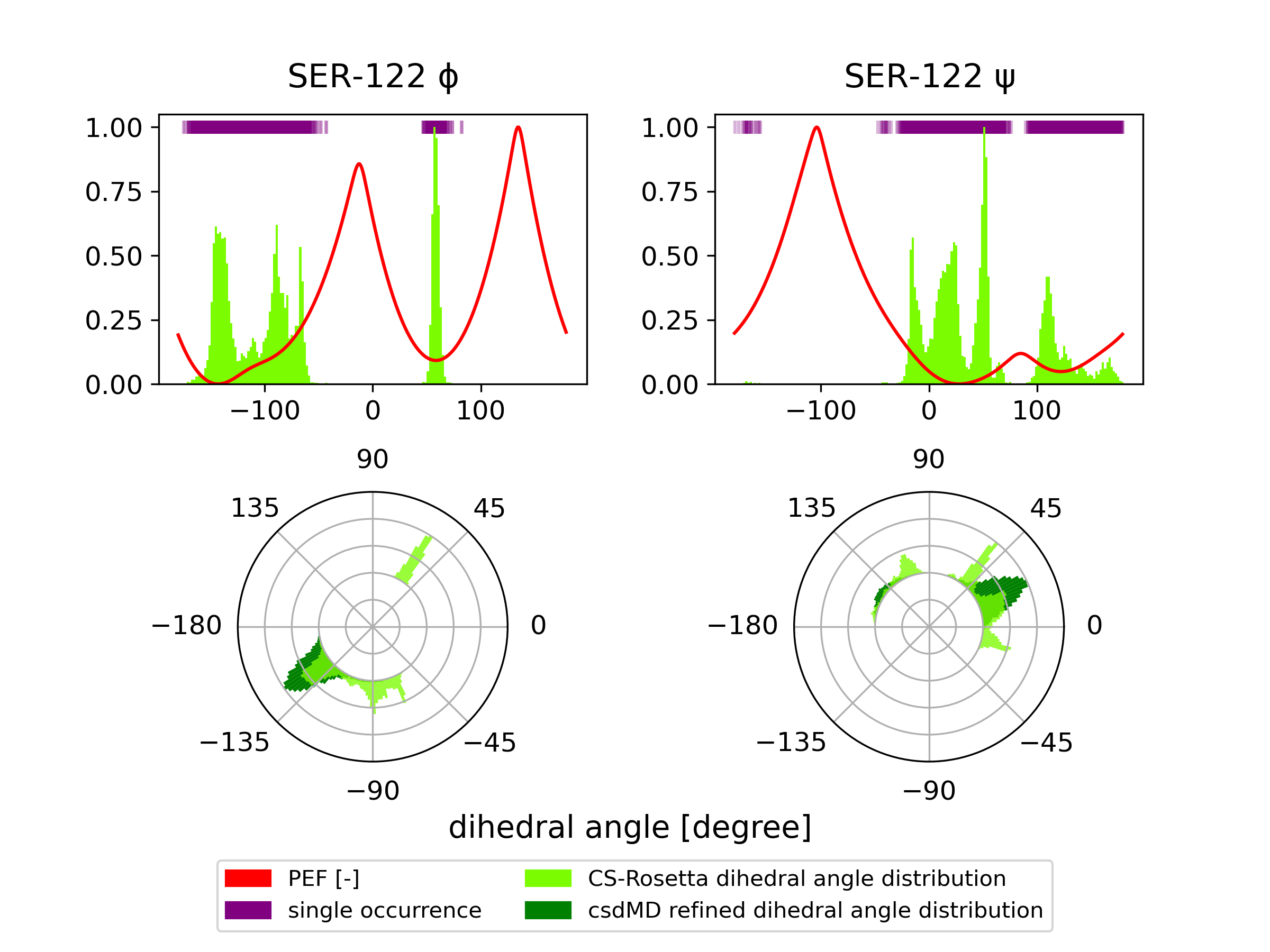

Supplement: Supplementary file 1 [file ijms-24-12101-s001.zip › KRAS-G12C-GDP-Mg_angle_figures/122-SER.png]

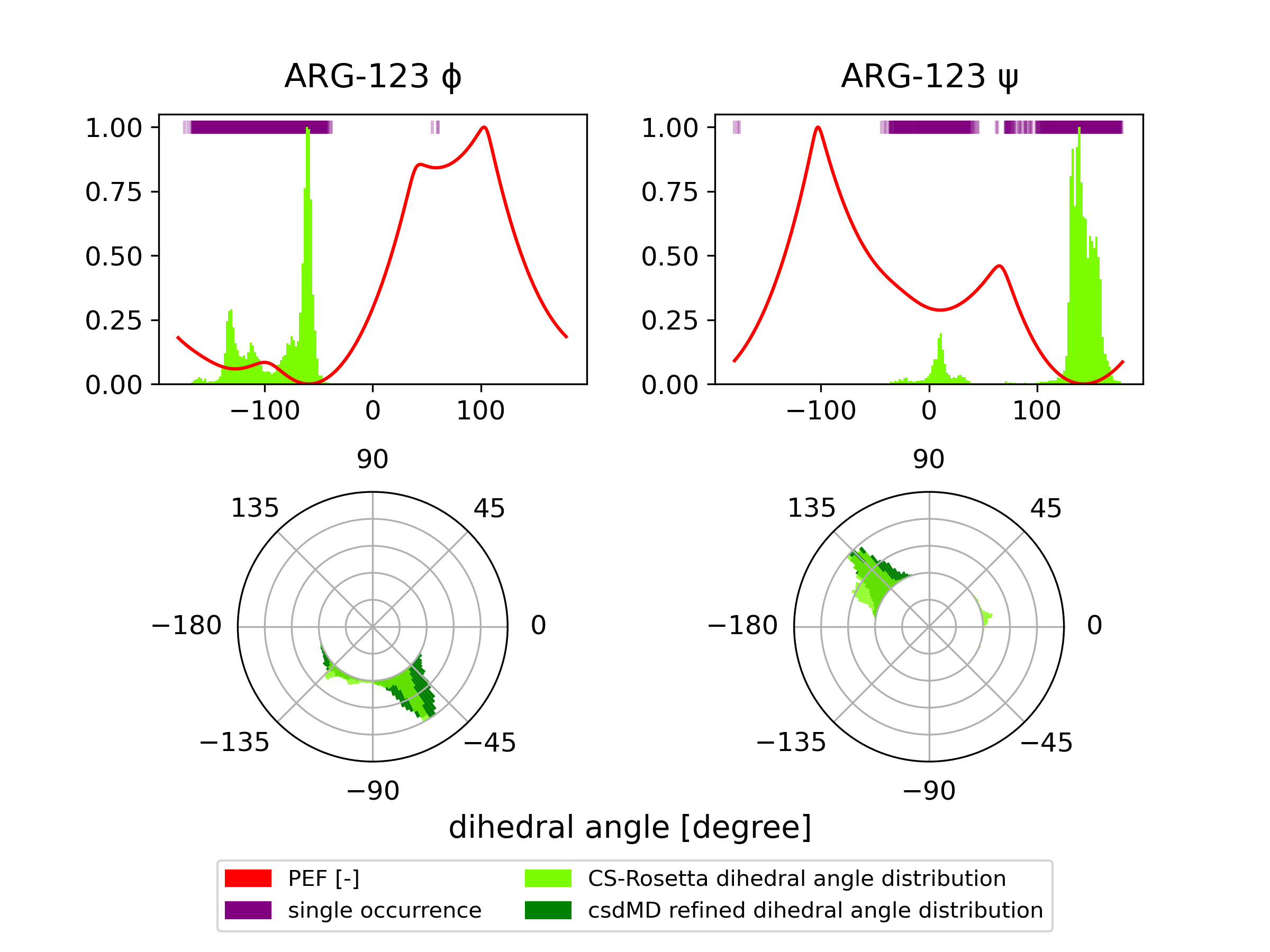

Supplement: Supplementary file 1 [file ijms-24-12101-s001.zip › KRAS-G12C-GDP-Mg_angle_figures/123-ARG.png]

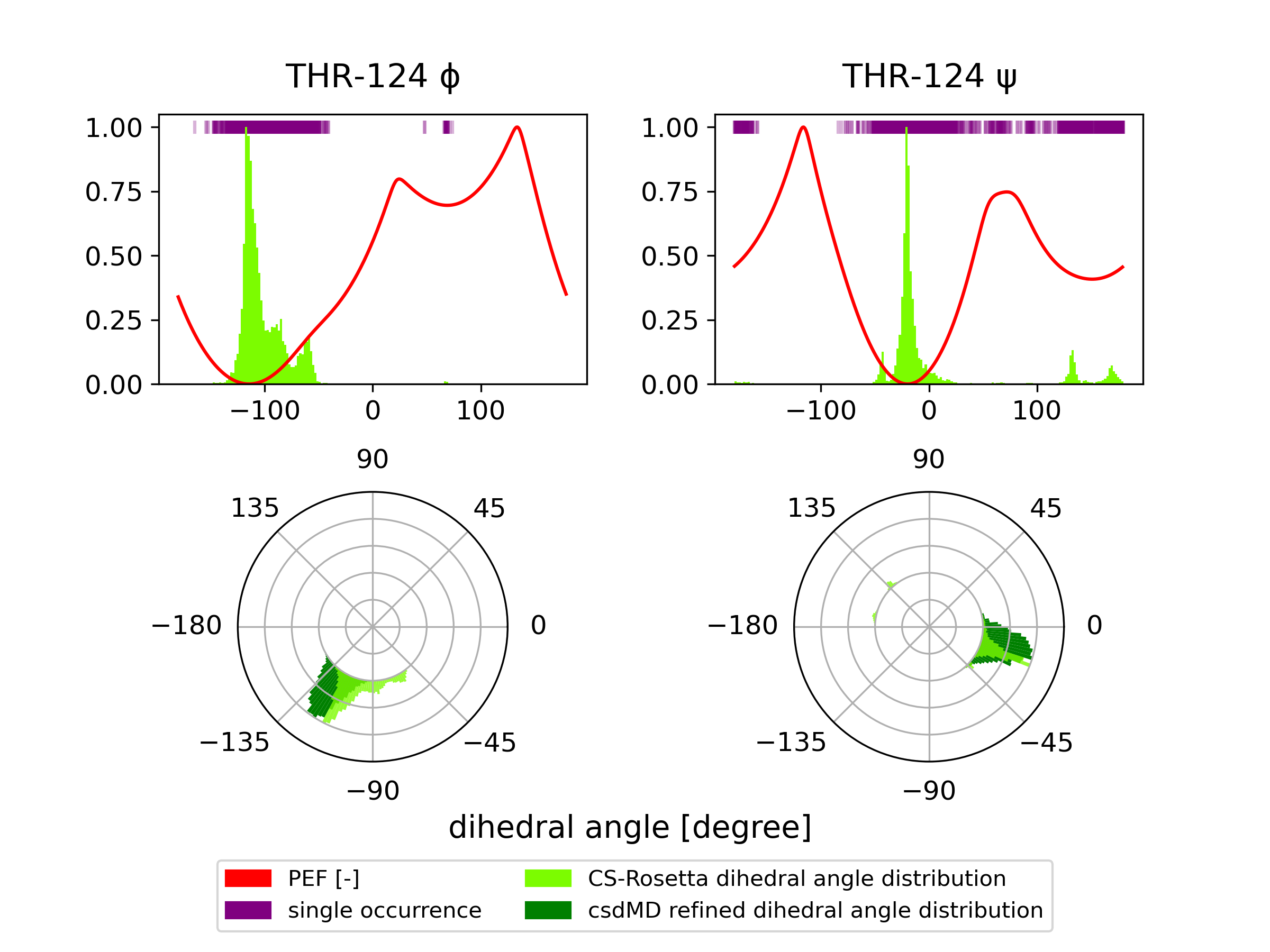

Supplement: Supplementary file 1 [file ijms-24-12101-s001.zip › KRAS-G12C-GDP-Mg_angle_figures/124-THR.png]

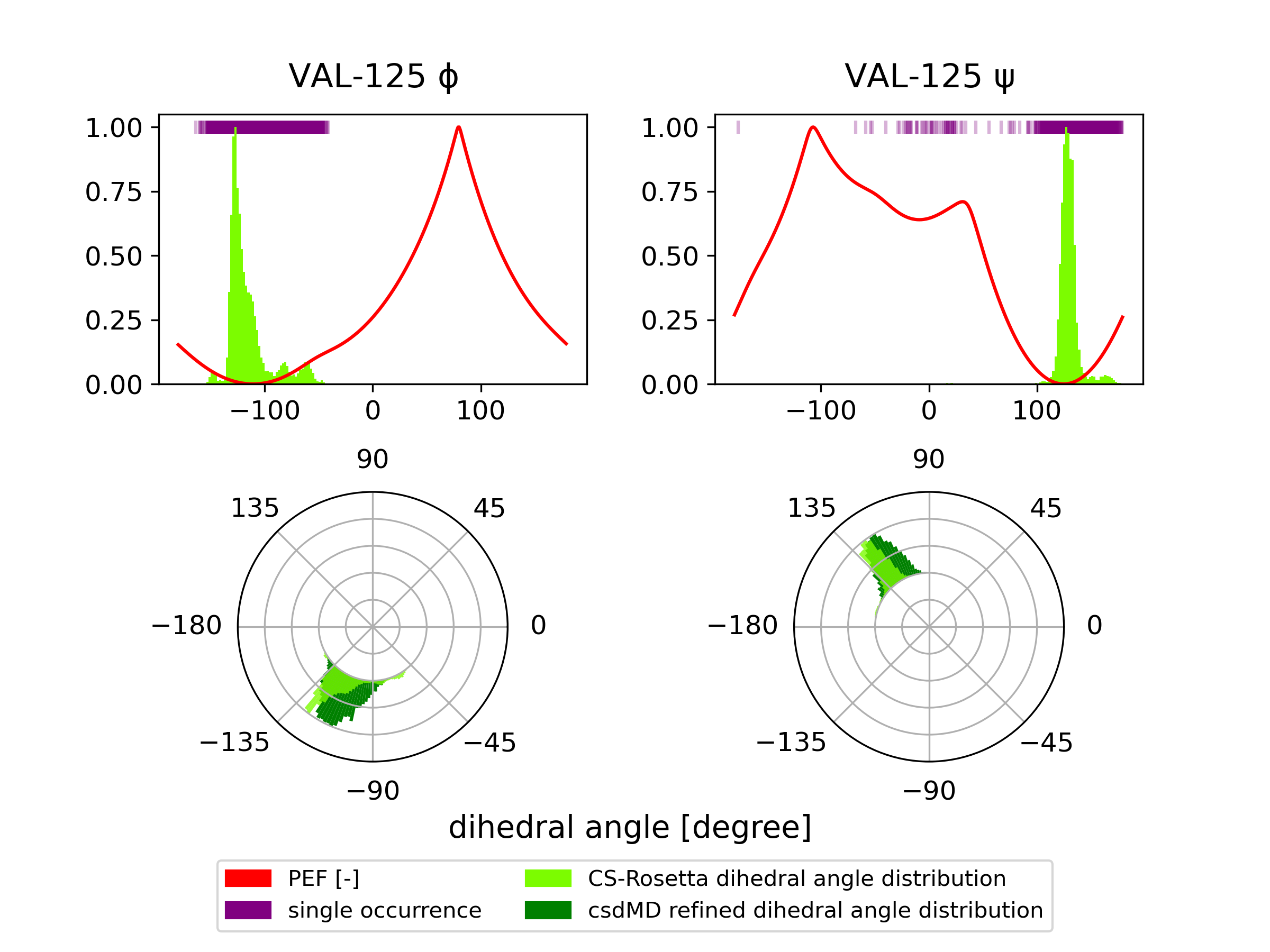

Supplement: Supplementary file 1 [file ijms-24-12101-s001.zip › KRAS-G12C-GDP-Mg_angle_figures/125-VAL.png]
