# Supplementary figures and images for: NMR-Chemical-Shift-Driven Protocol Reveals the Cofactor-Bound, Complete Structure of Dynamic Intermediates of the Catalytic Cycle of Oncogenic KRAS G12C Protein and the Significance of the Mg2+ Ion (part 1 of 2)
Source: Int J Mol Sci. 2023 Jul 28;24(15):12101. doi: 10.3390/ijms241512101 (PMC10418480; doi:10.3390/ijms241512101)

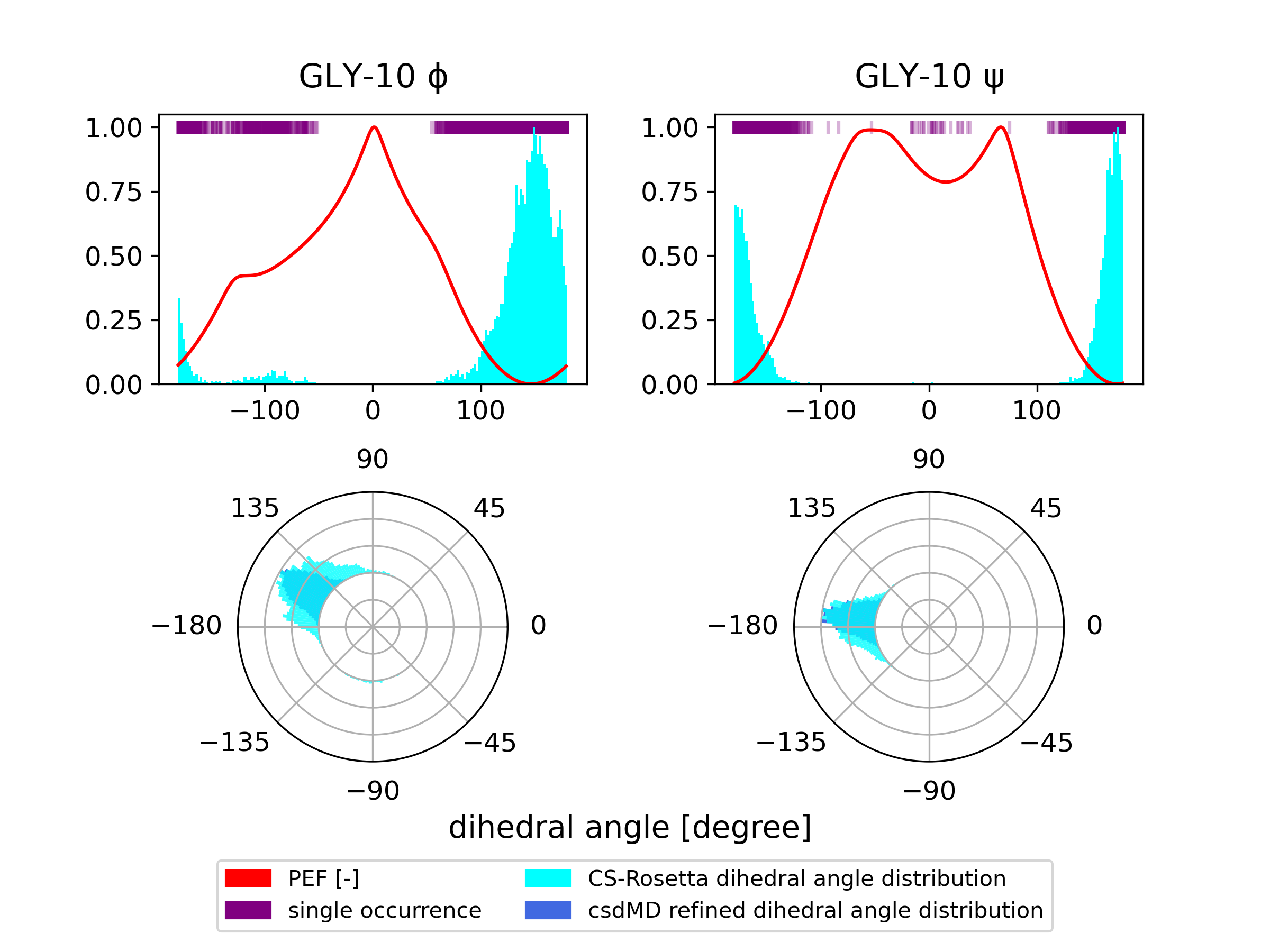

Supplement: Supplementary file 1 [file ijms-24-12101-s001.zip › KRAS-G12C-GDP-Mg-free_angle_figures/10-GLY.png]

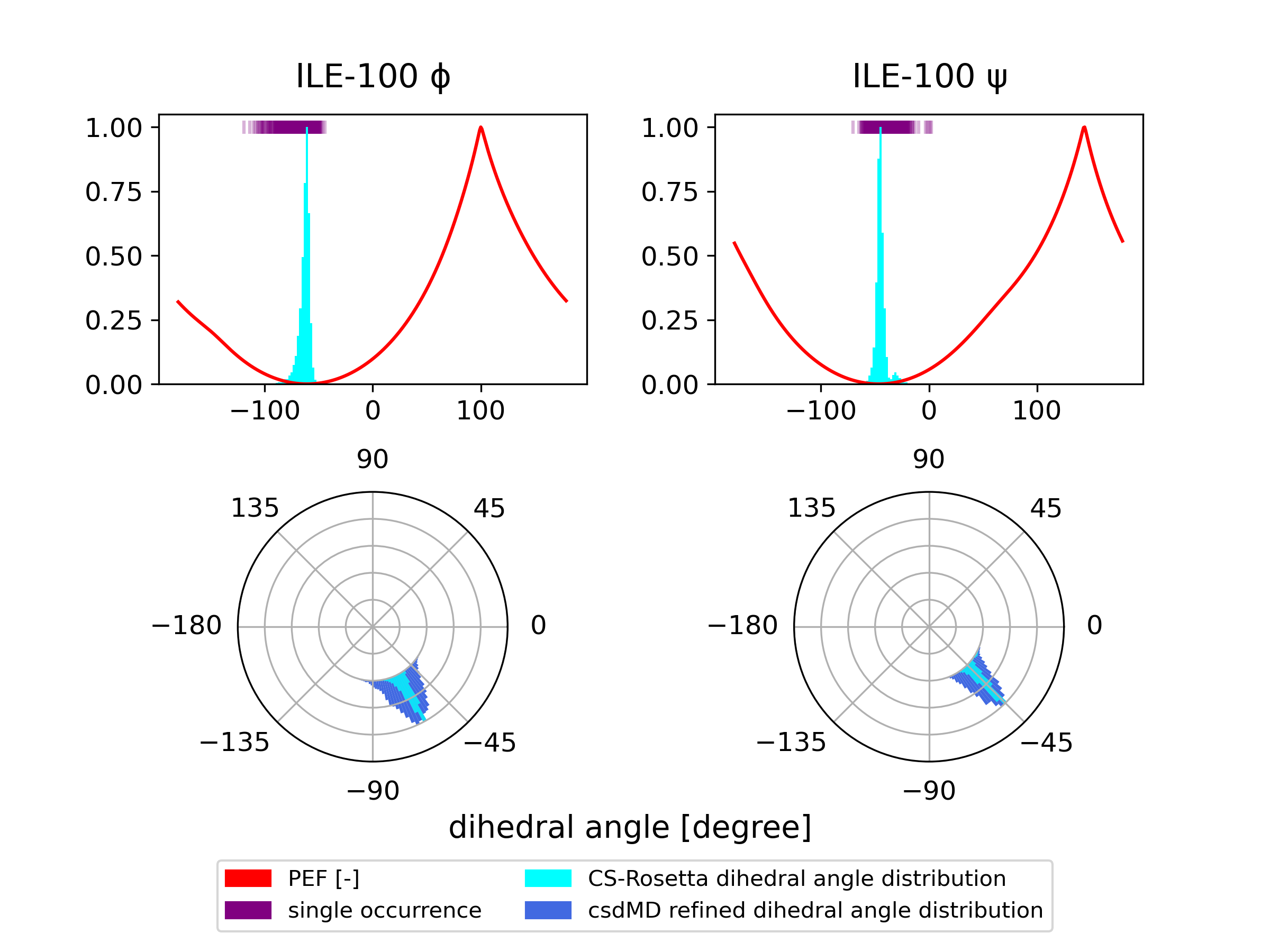

Supplement: Supplementary file 1 [file ijms-24-12101-s001.zip › KRAS-G12C-GDP-Mg-free_angle_figures/100-ILE.png]

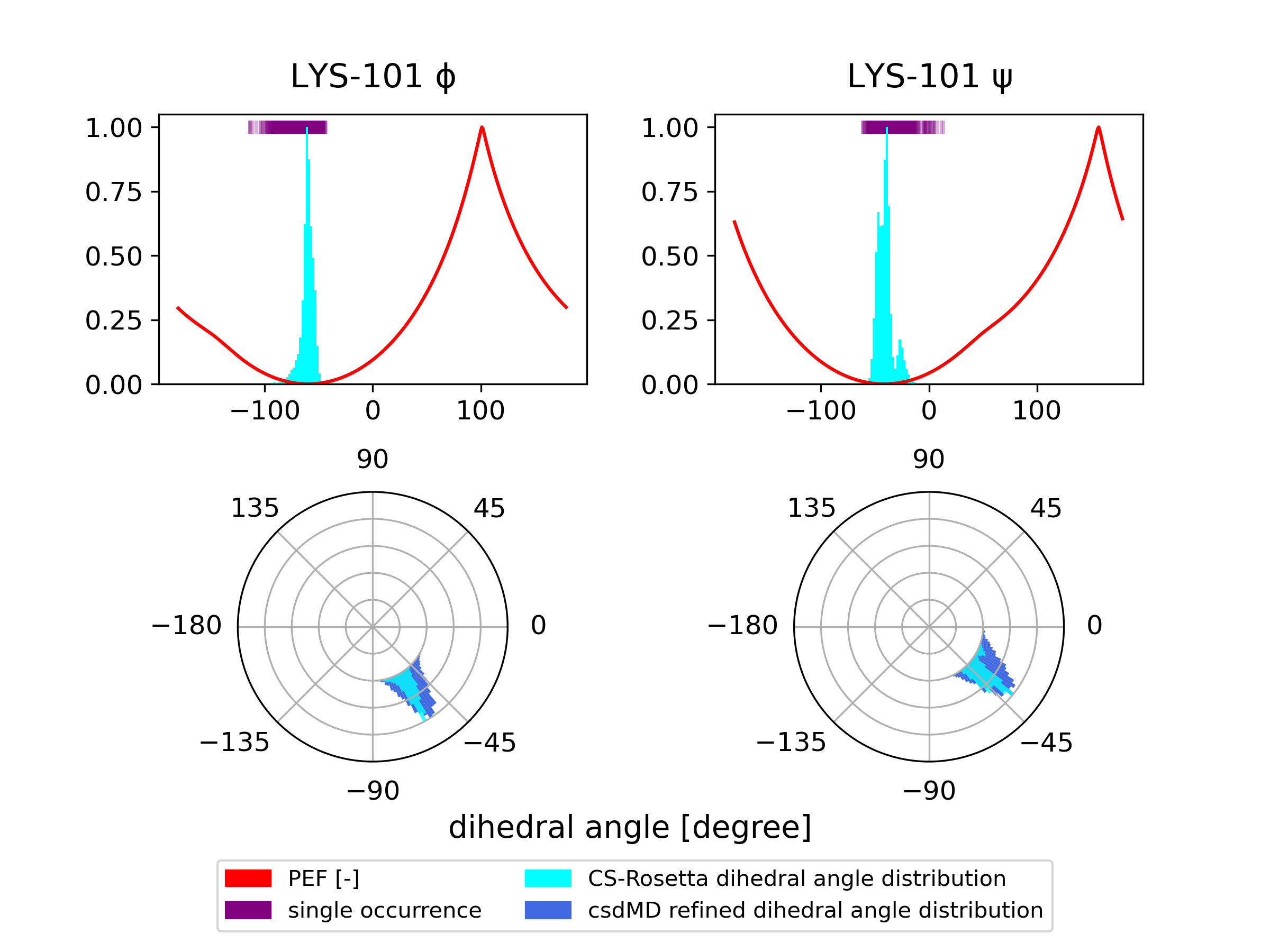

Supplement: Supplementary file 1 [file ijms-24-12101-s001.zip › KRAS-G12C-GDP-Mg-free_angle_figures/101-LYS.png]

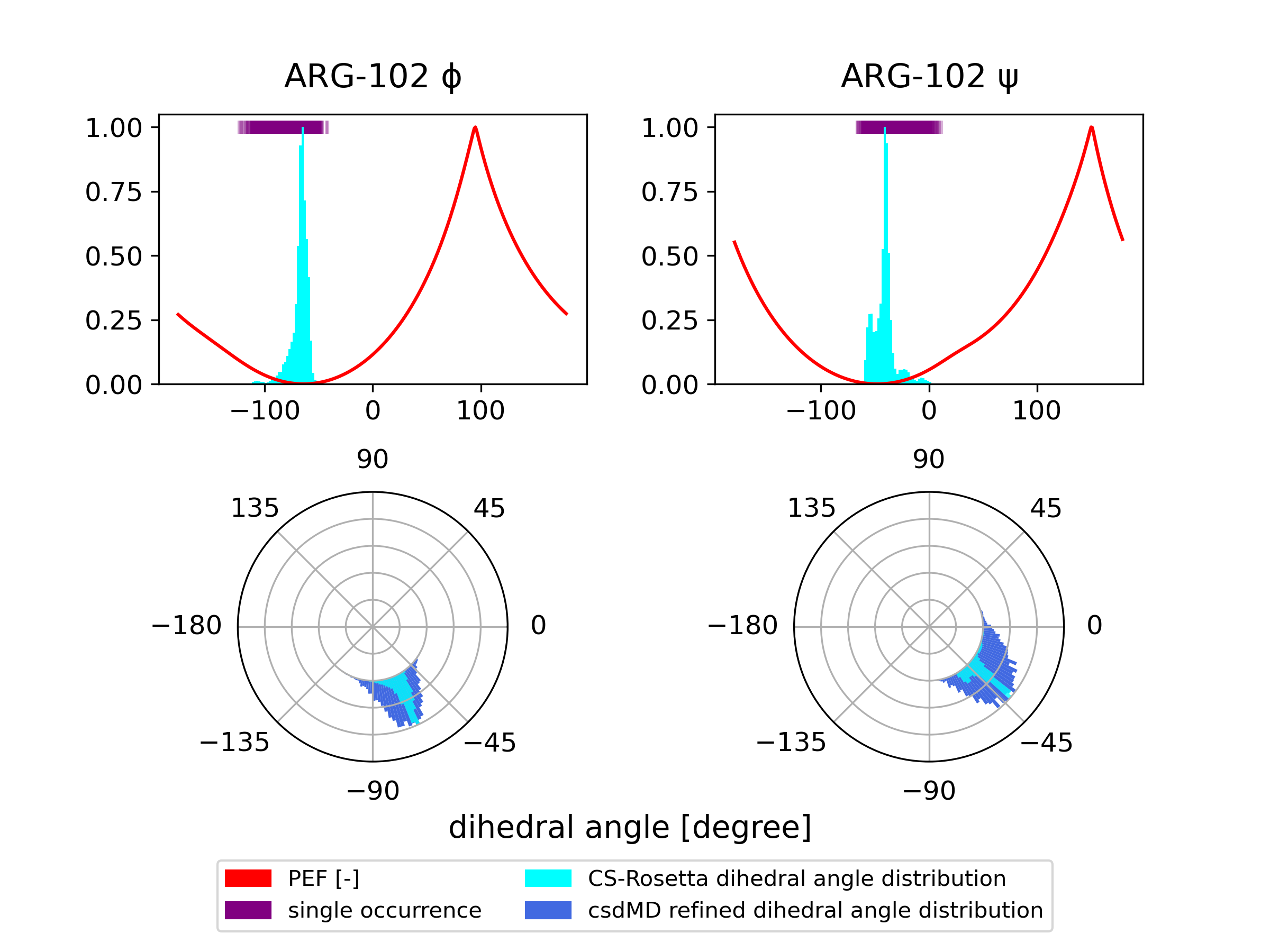

Supplement: Supplementary file 1 [file ijms-24-12101-s001.zip › KRAS-G12C-GDP-Mg-free_angle_figures/102-ARG.png]

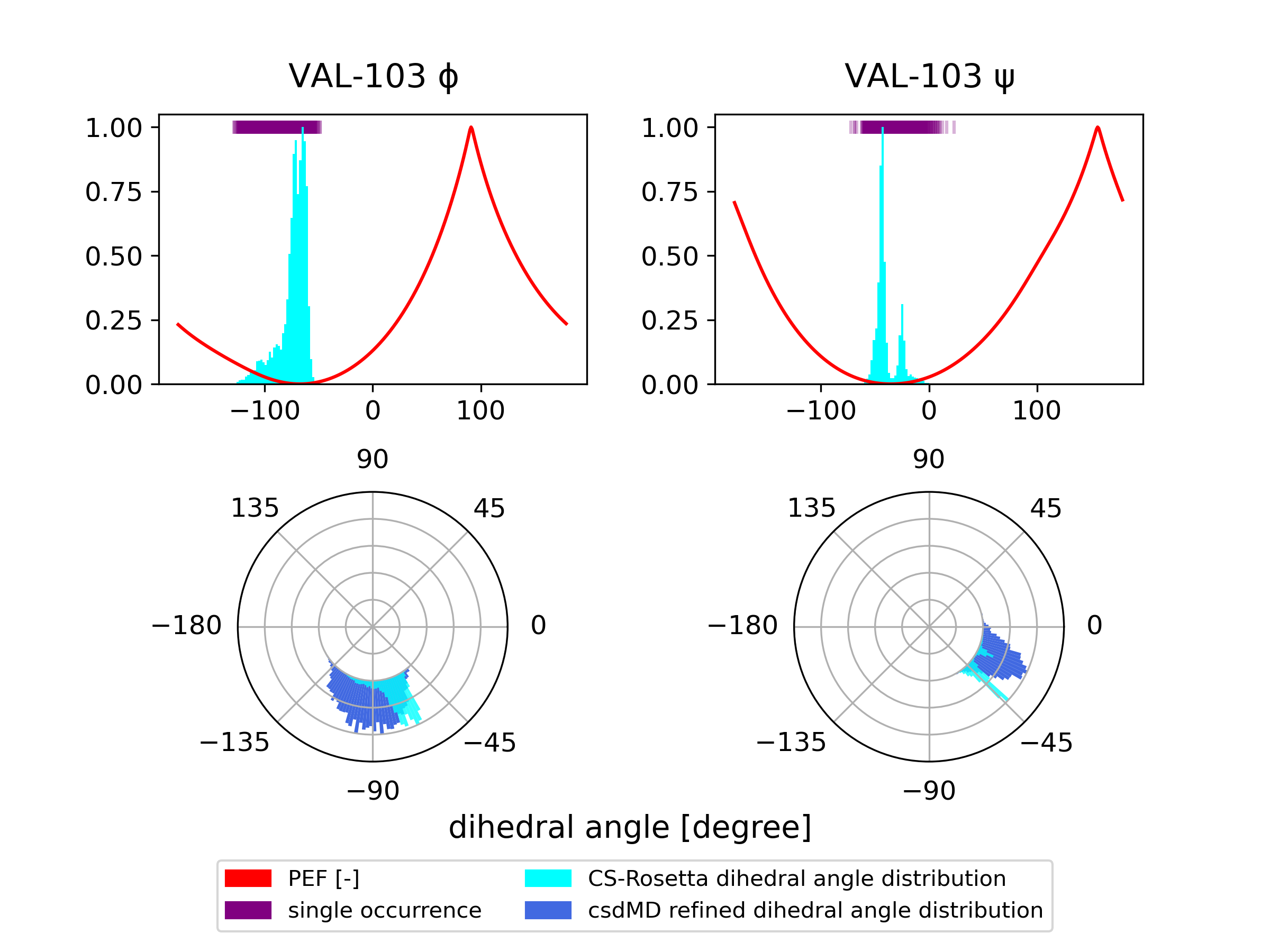

Supplement: Supplementary file 1 [file ijms-24-12101-s001.zip › KRAS-G12C-GDP-Mg-free_angle_figures/103-VAL.png]

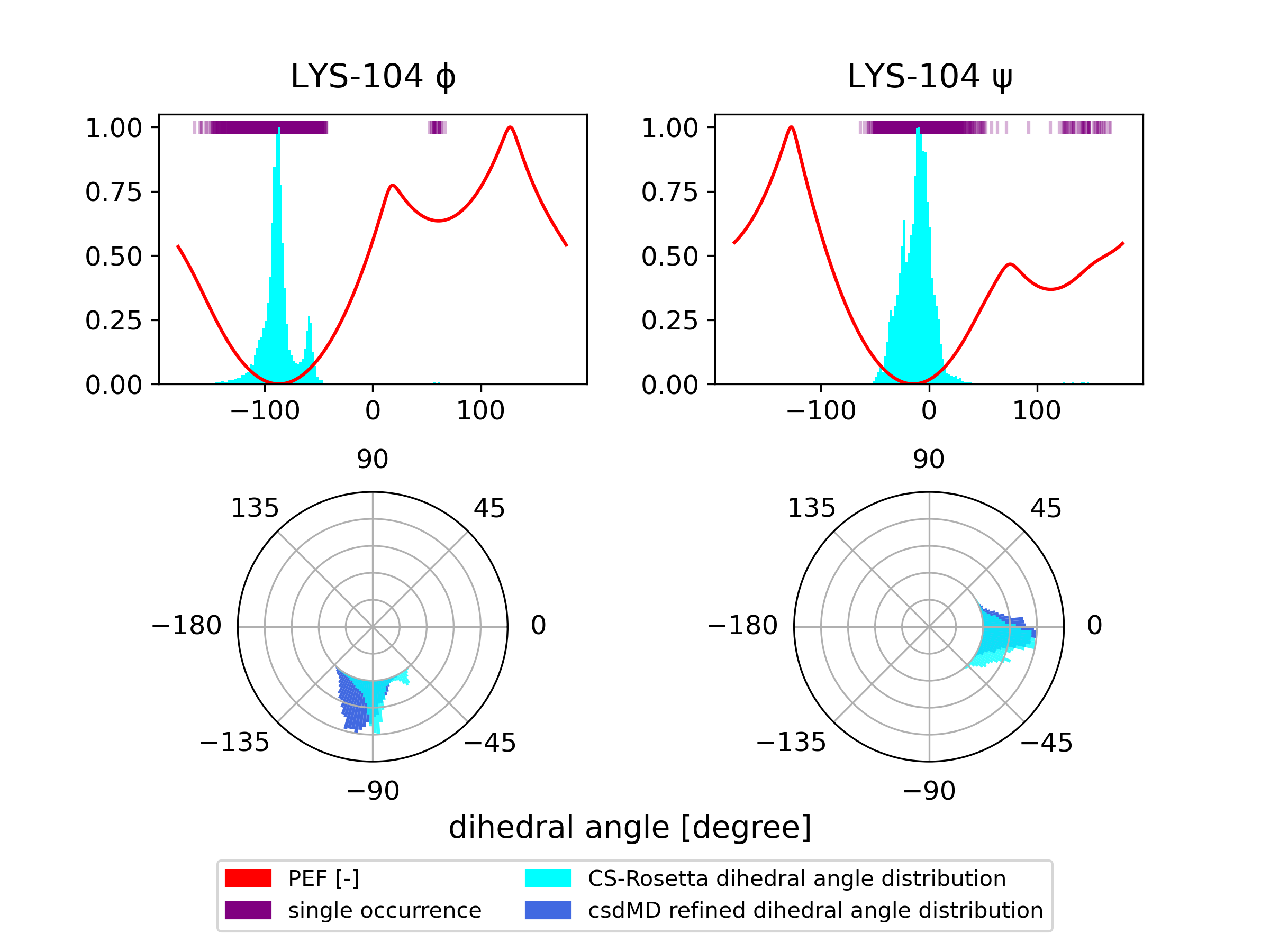

Supplement: Supplementary file 1 [file ijms-24-12101-s001.zip › KRAS-G12C-GDP-Mg-free_angle_figures/104-LYS.png]

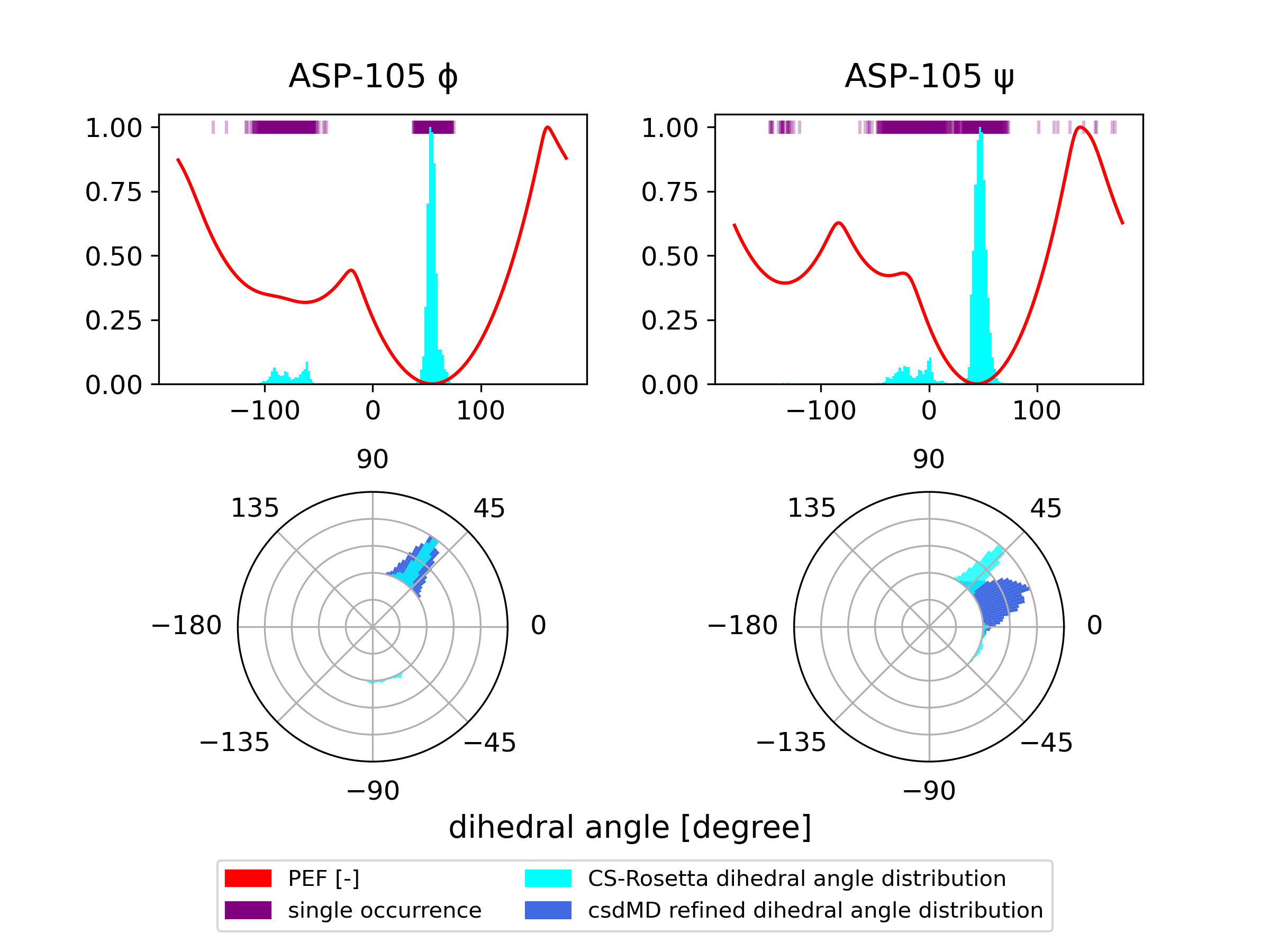

Supplement: Supplementary file 1 [file ijms-24-12101-s001.zip › KRAS-G12C-GDP-Mg-free_angle_figures/105-ASP.png]

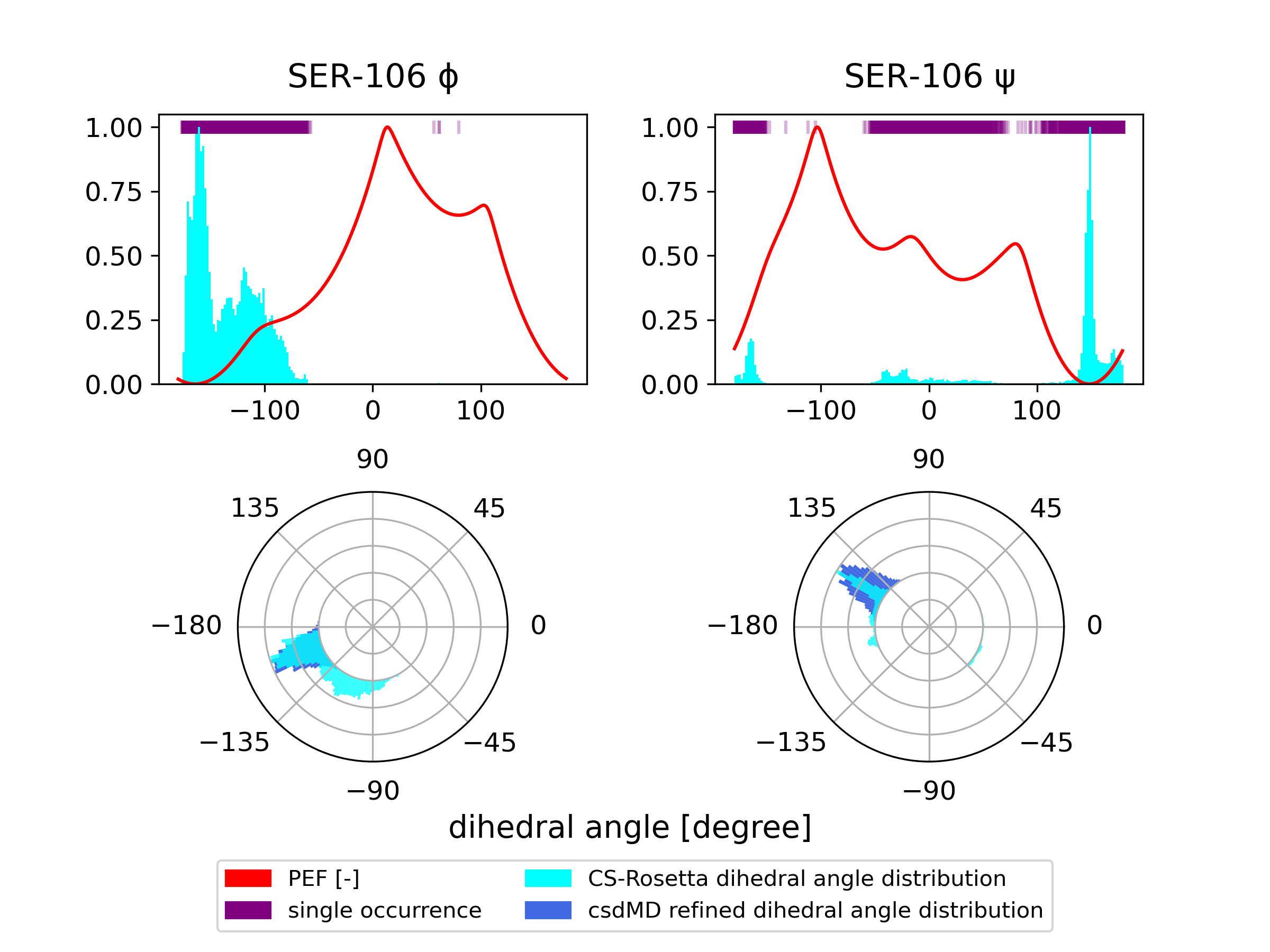

Supplement: Supplementary file 1 [file ijms-24-12101-s001.zip › KRAS-G12C-GDP-Mg-free_angle_figures/106-SER.png]

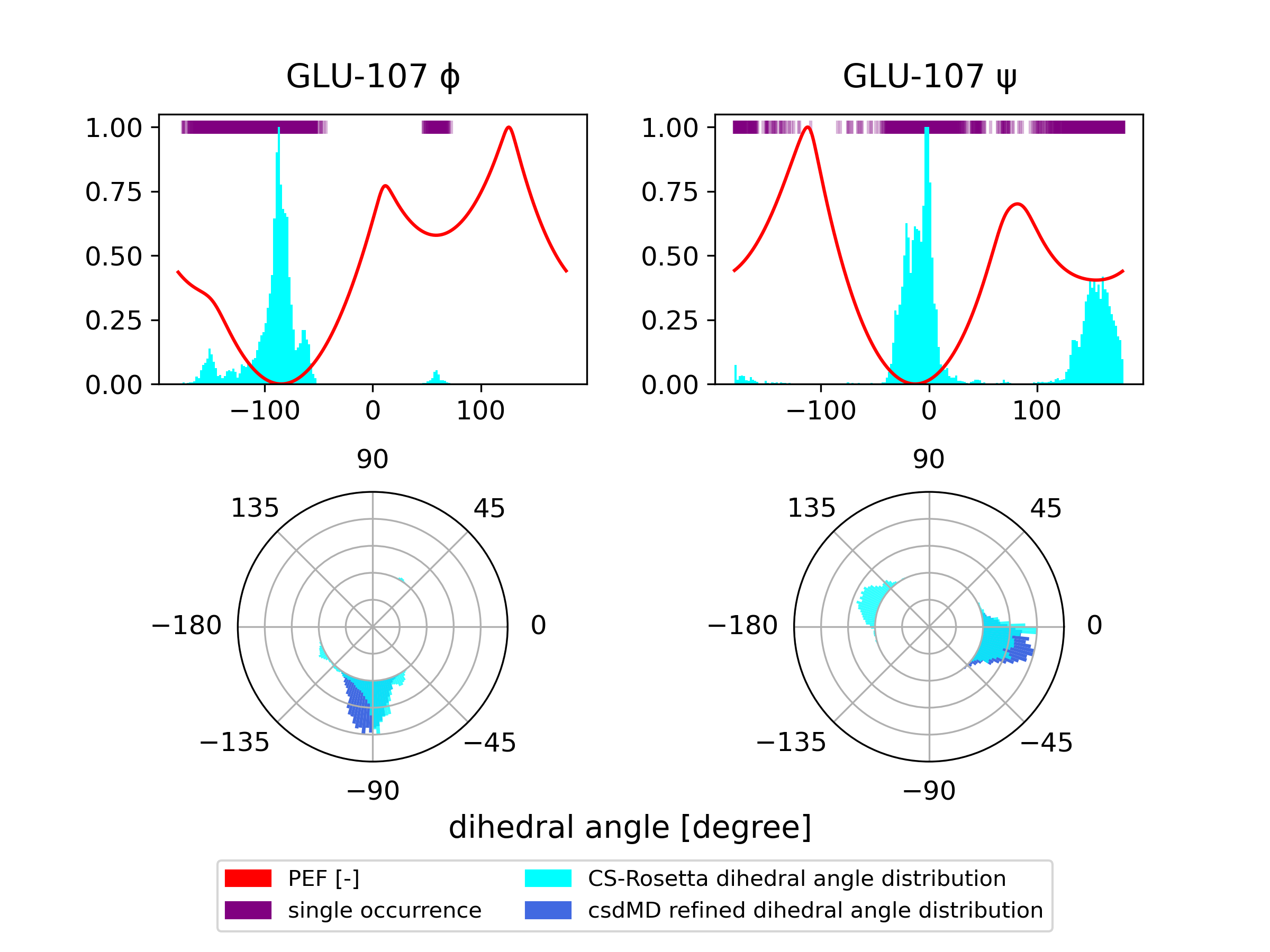

Supplement: Supplementary file 1 [file ijms-24-12101-s001.zip › KRAS-G12C-GDP-Mg-free_angle_figures/107-GLU.png]

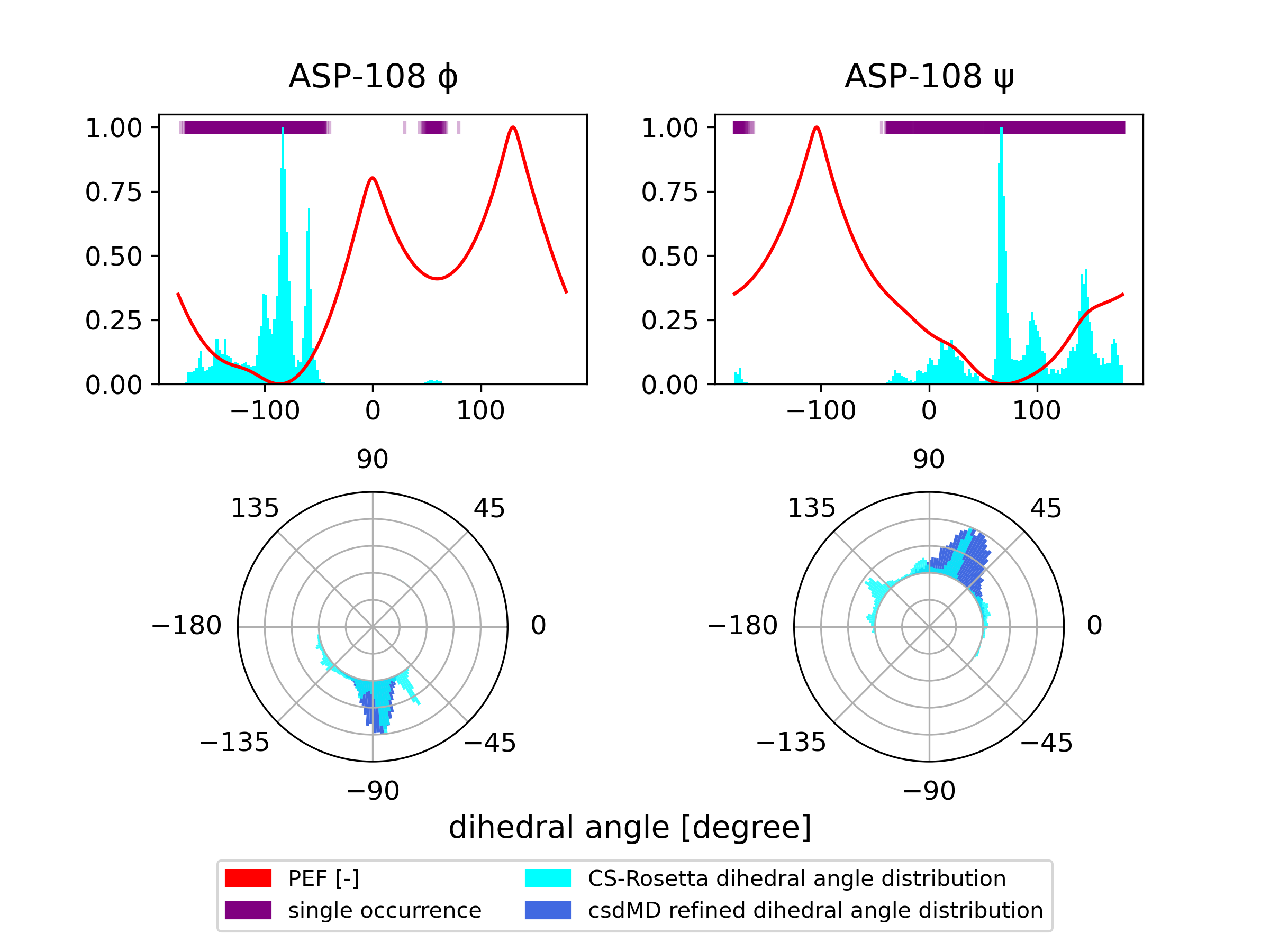

Supplement: Supplementary file 1 [file ijms-24-12101-s001.zip › KRAS-G12C-GDP-Mg-free_angle_figures/108-ASP.png]

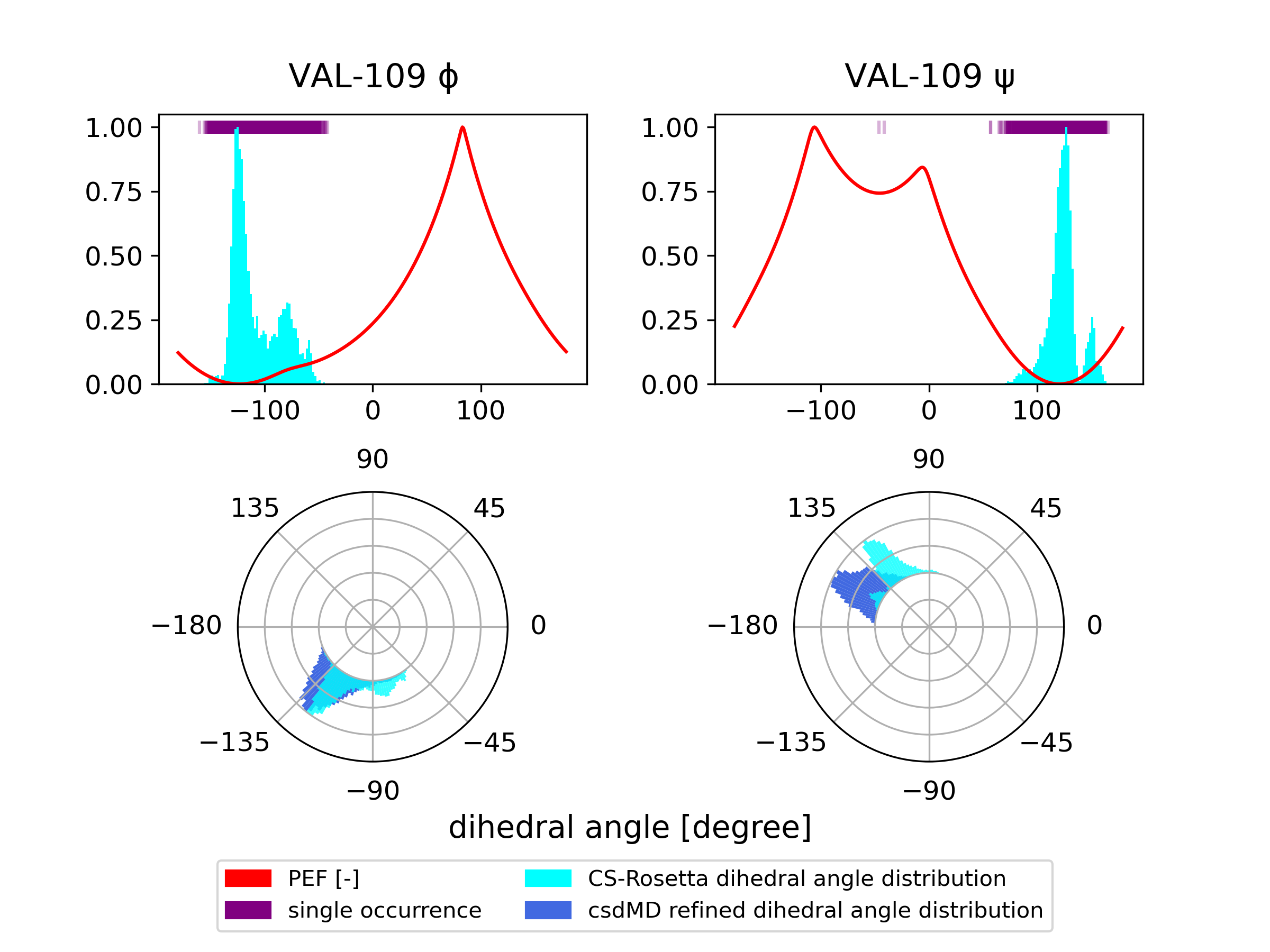

Supplement: Supplementary file 1 [file ijms-24-12101-s001.zip › KRAS-G12C-GDP-Mg-free_angle_figures/109-VAL.png]

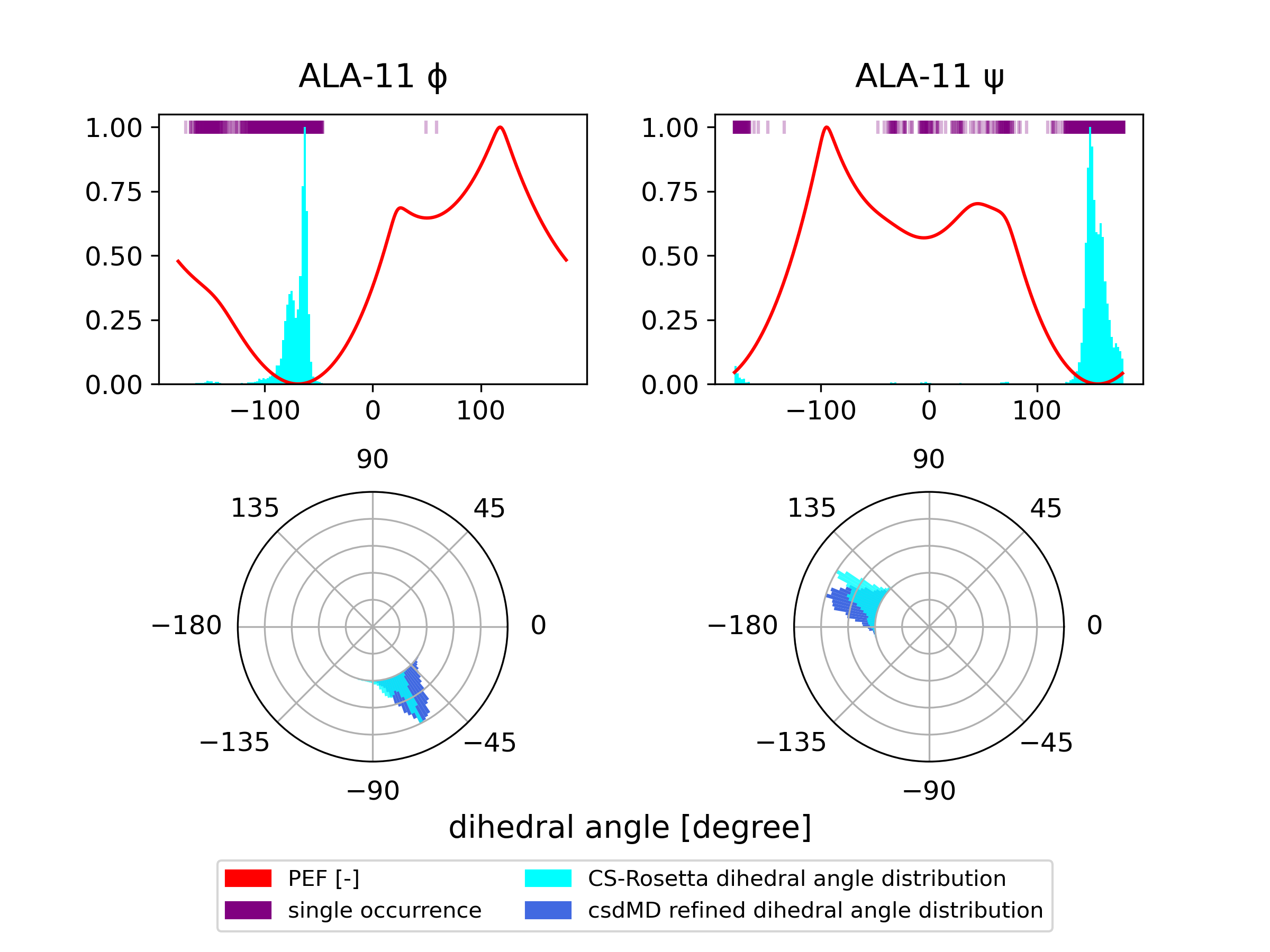

Supplement: Supplementary file 1 [file ijms-24-12101-s001.zip › KRAS-G12C-GDP-Mg-free_angle_figures/11-ALA.png]

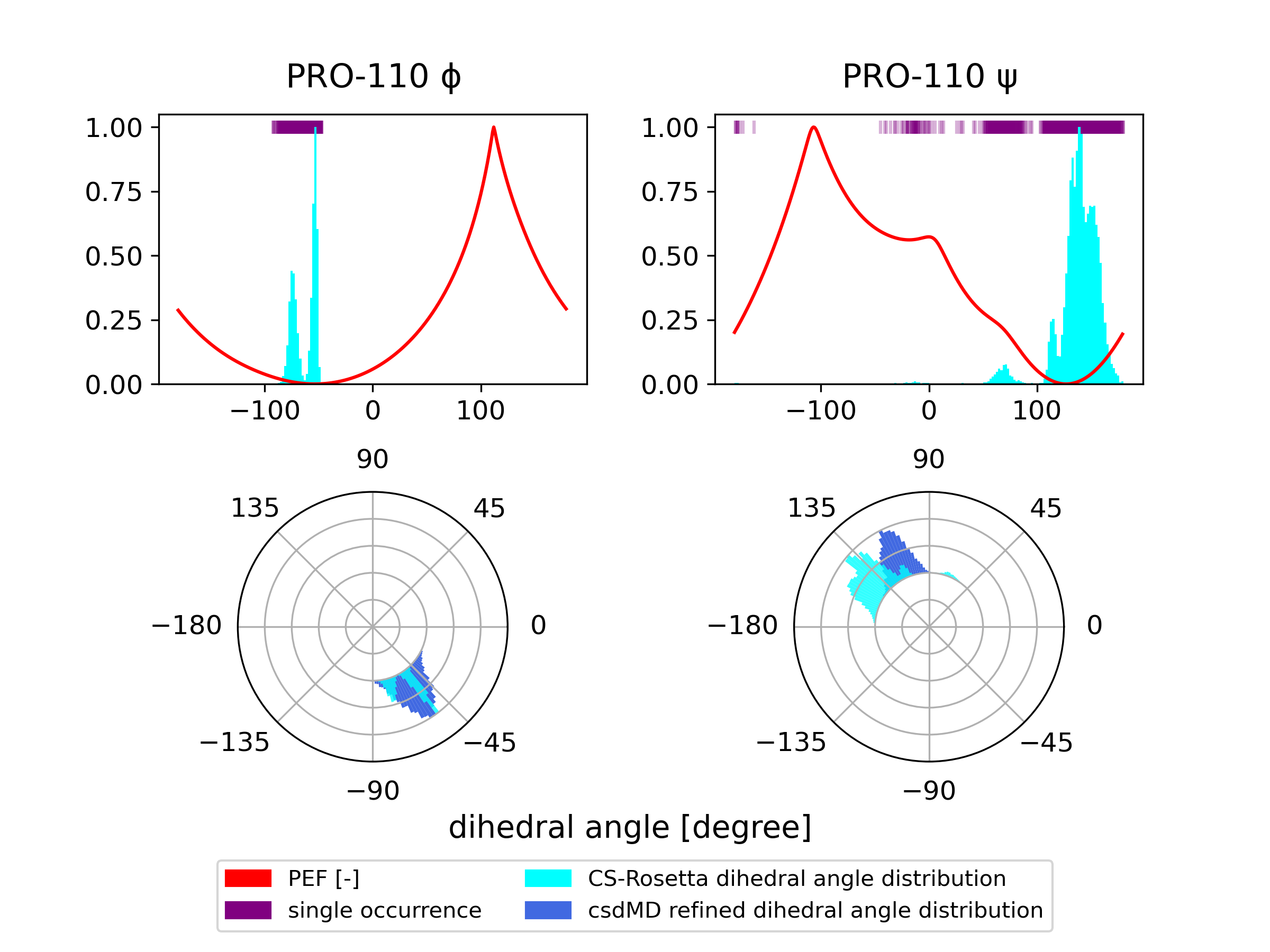

Supplement: Supplementary file 1 [file ijms-24-12101-s001.zip › KRAS-G12C-GDP-Mg-free_angle_figures/110-PRO.png]

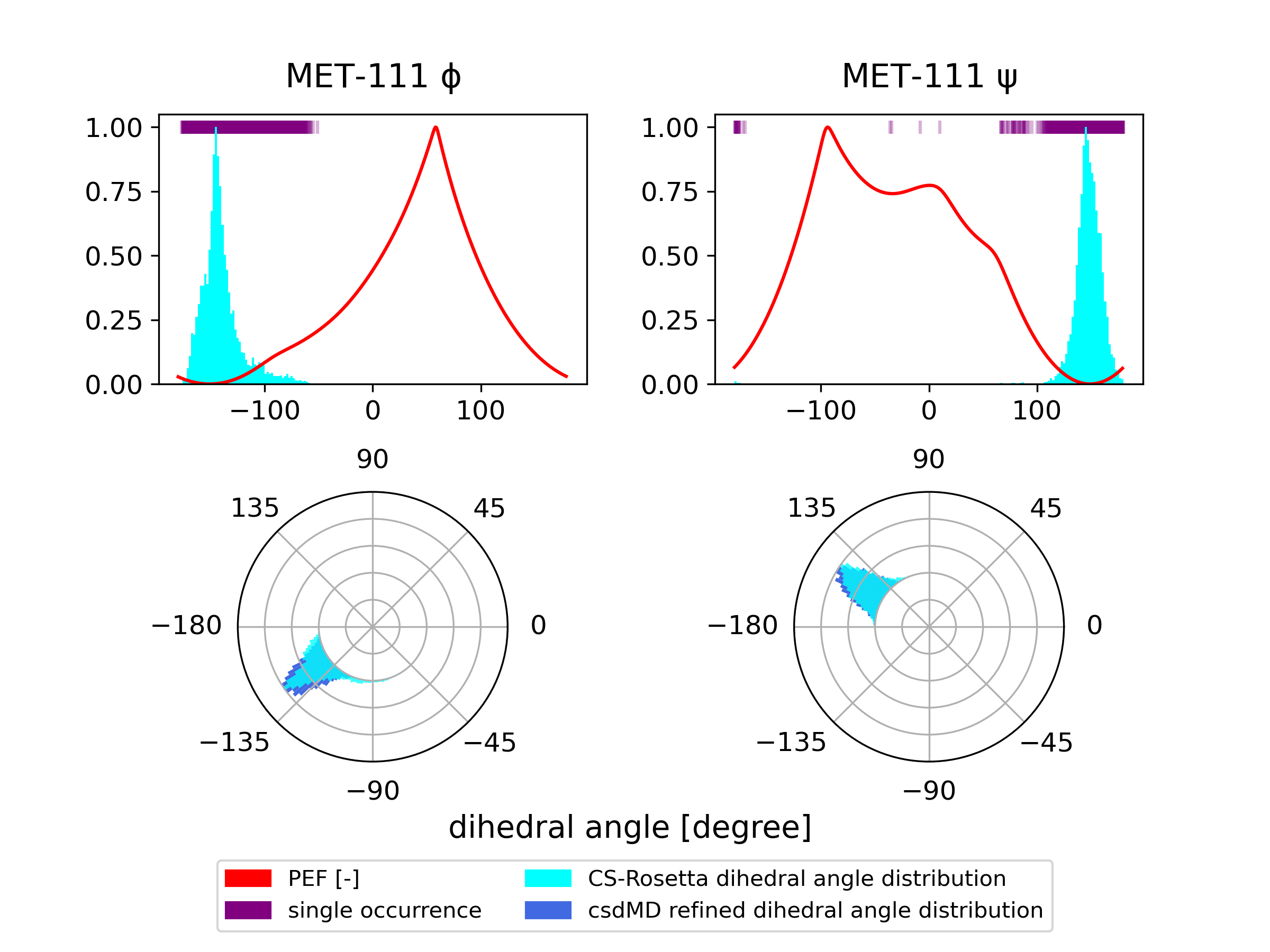

Supplement: Supplementary file 1 [file ijms-24-12101-s001.zip › KRAS-G12C-GDP-Mg-free_angle_figures/111-MET.png]

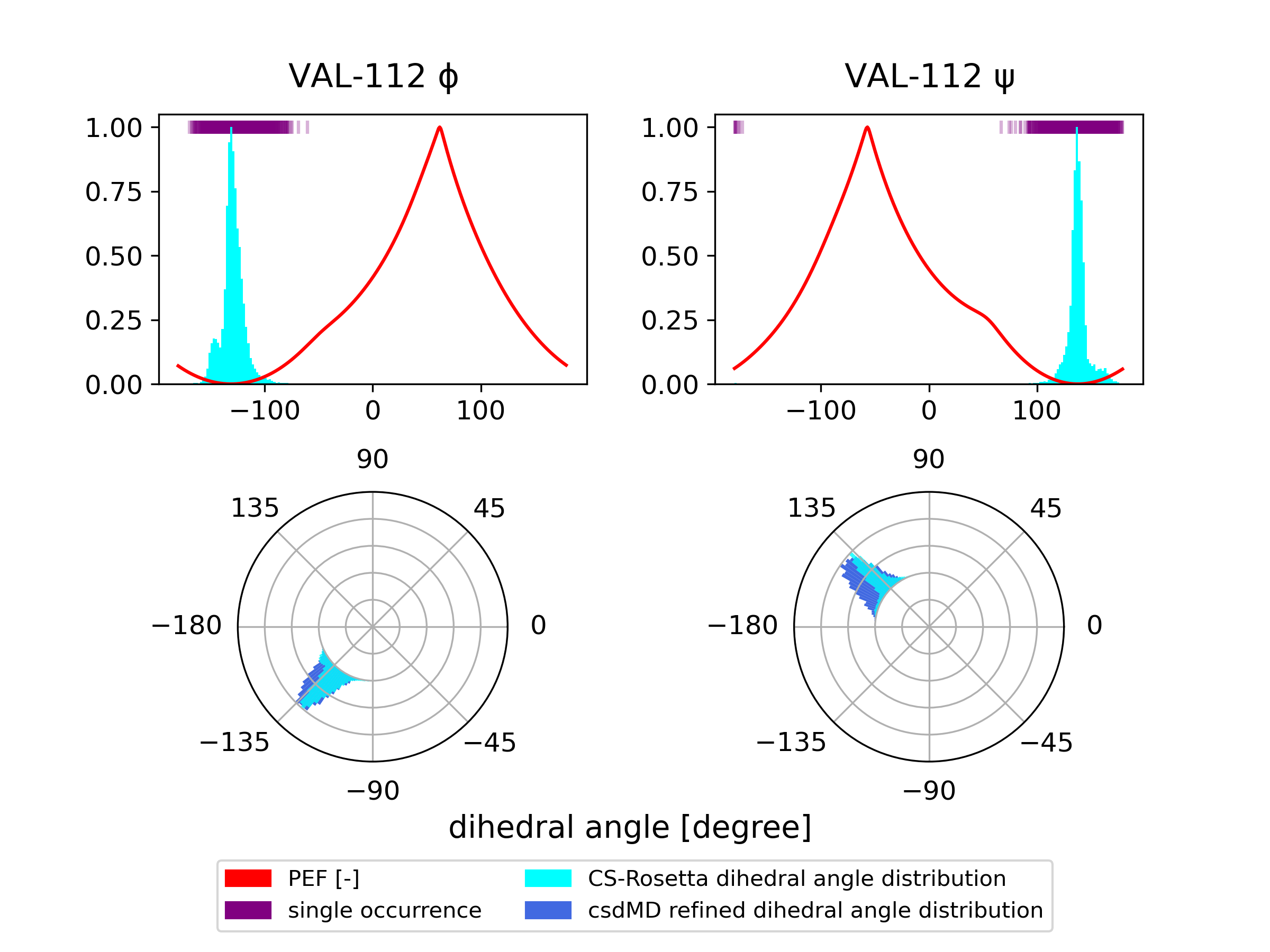

Supplement: Supplementary file 1 [file ijms-24-12101-s001.zip › KRAS-G12C-GDP-Mg-free_angle_figures/112-VAL.png]

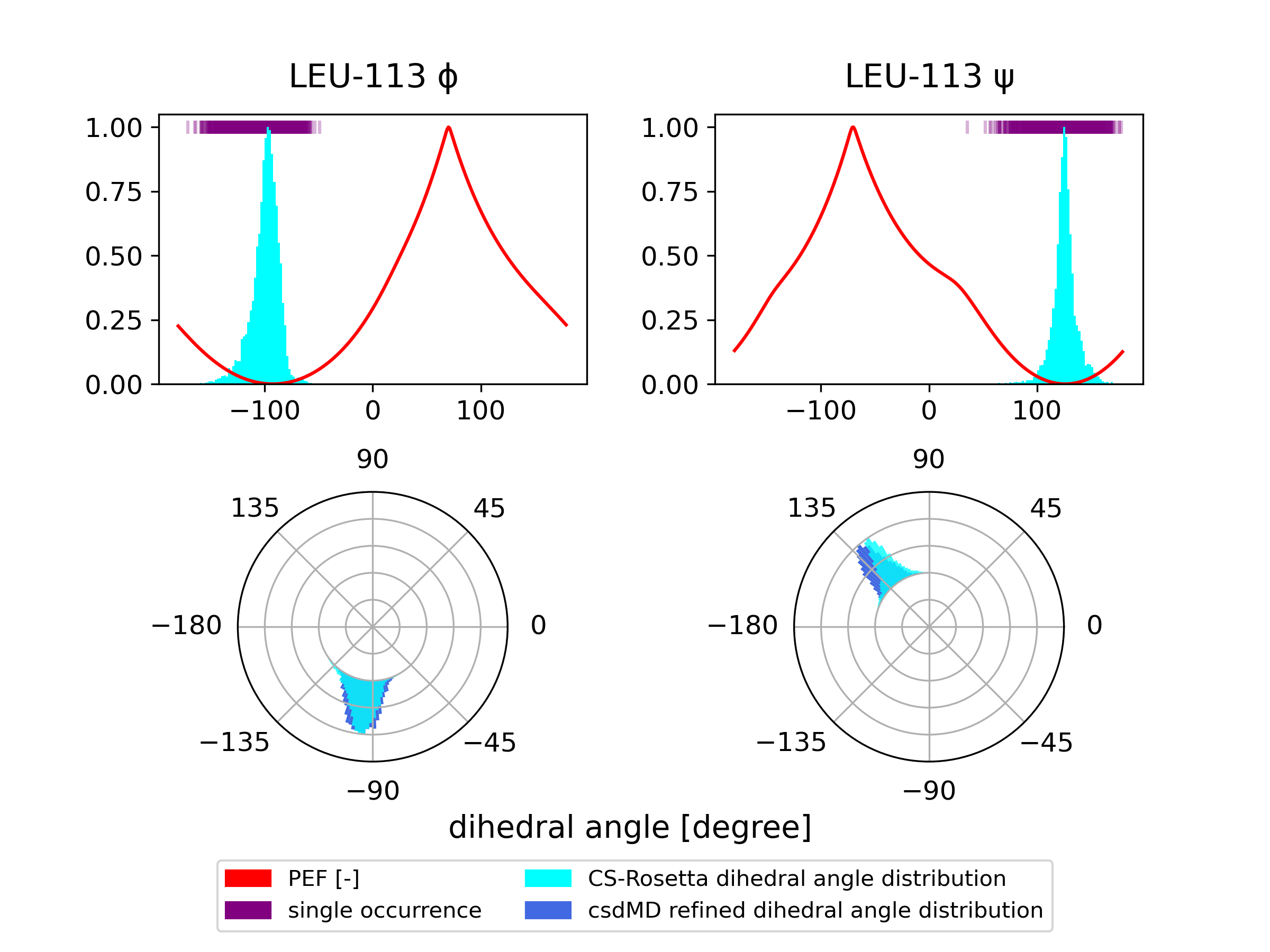

Supplement: Supplementary file 1 [file ijms-24-12101-s001.zip › KRAS-G12C-GDP-Mg-free_angle_figures/113-LEU.png]

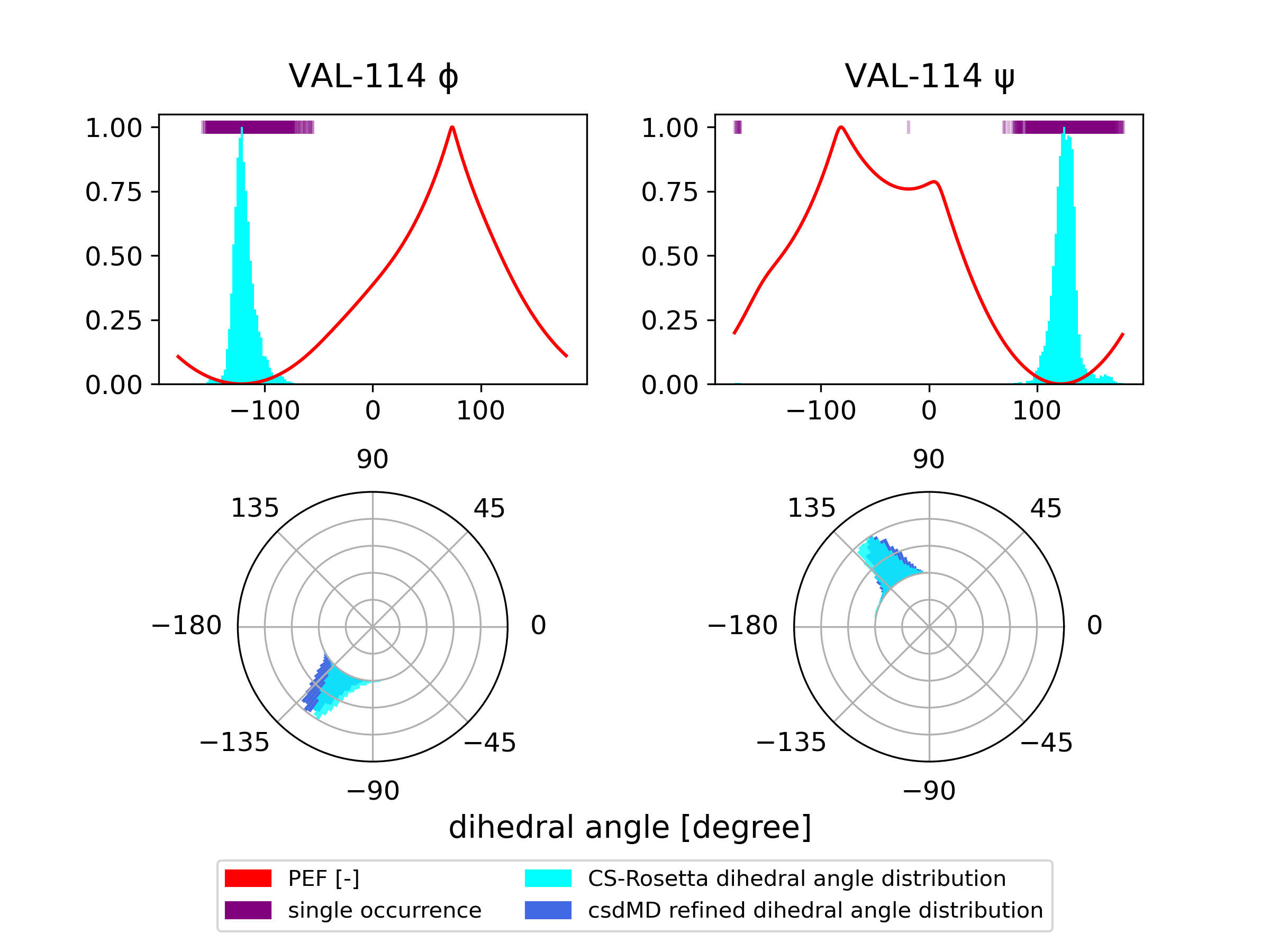

Supplement: Supplementary file 1 [file ijms-24-12101-s001.zip › KRAS-G12C-GDP-Mg-free_angle_figures/114-VAL.png]

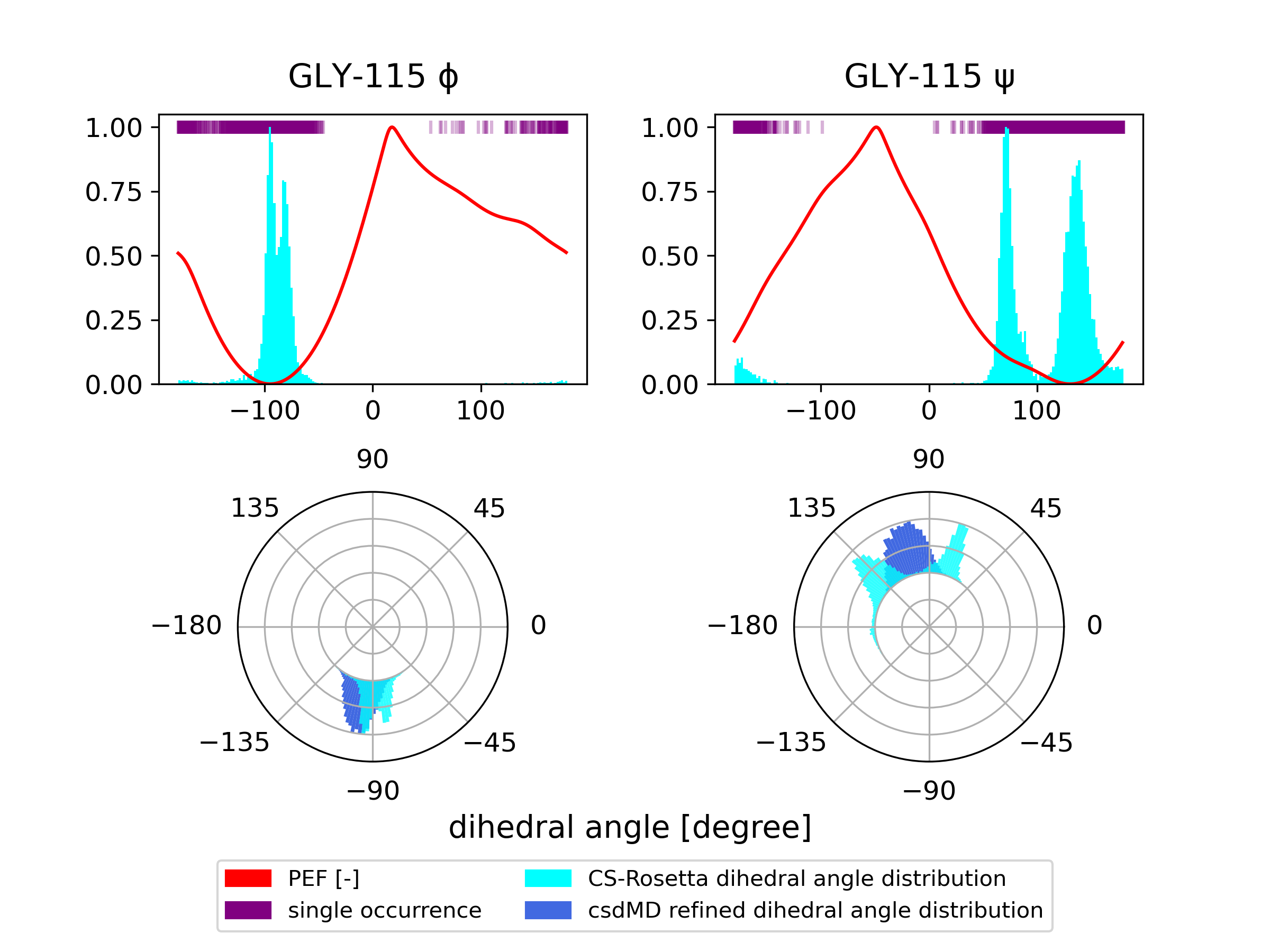

Supplement: Supplementary file 1 [file ijms-24-12101-s001.zip › KRAS-G12C-GDP-Mg-free_angle_figures/115-GLY.png]

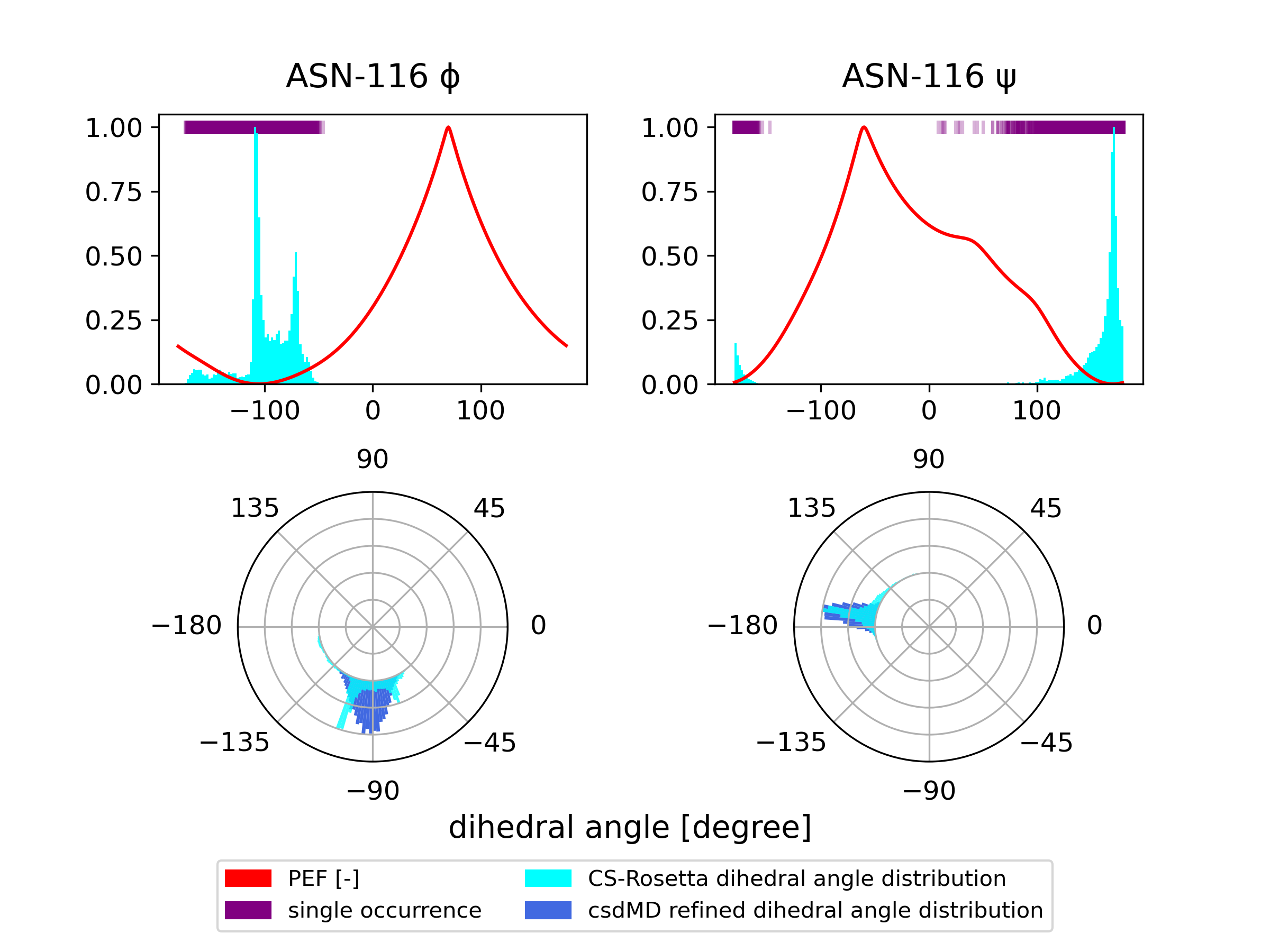

Supplement: Supplementary file 1 [file ijms-24-12101-s001.zip › KRAS-G12C-GDP-Mg-free_angle_figures/116-ASN.png]

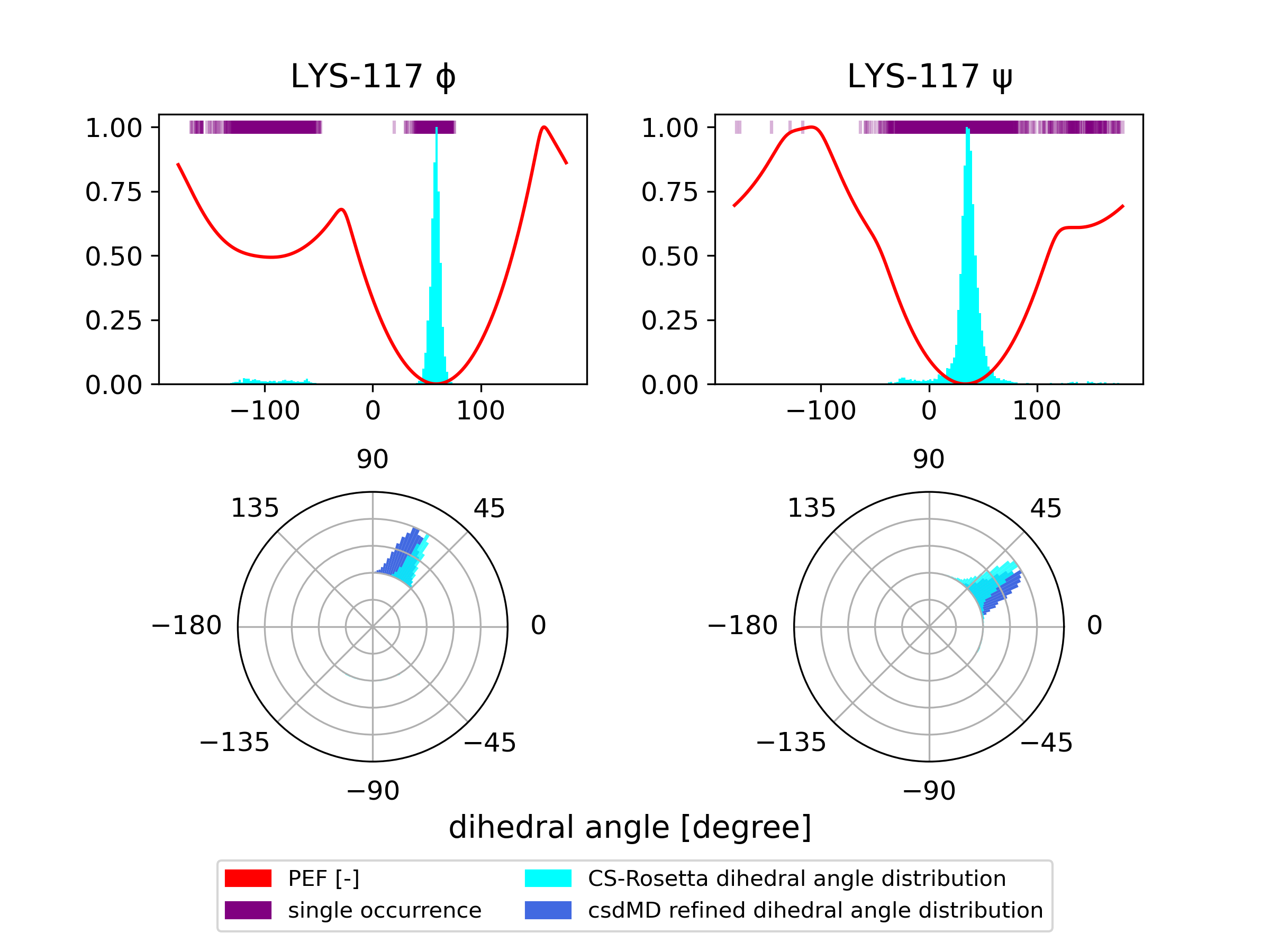

Supplement: Supplementary file 1 [file ijms-24-12101-s001.zip › KRAS-G12C-GDP-Mg-free_angle_figures/117-LYS.png]

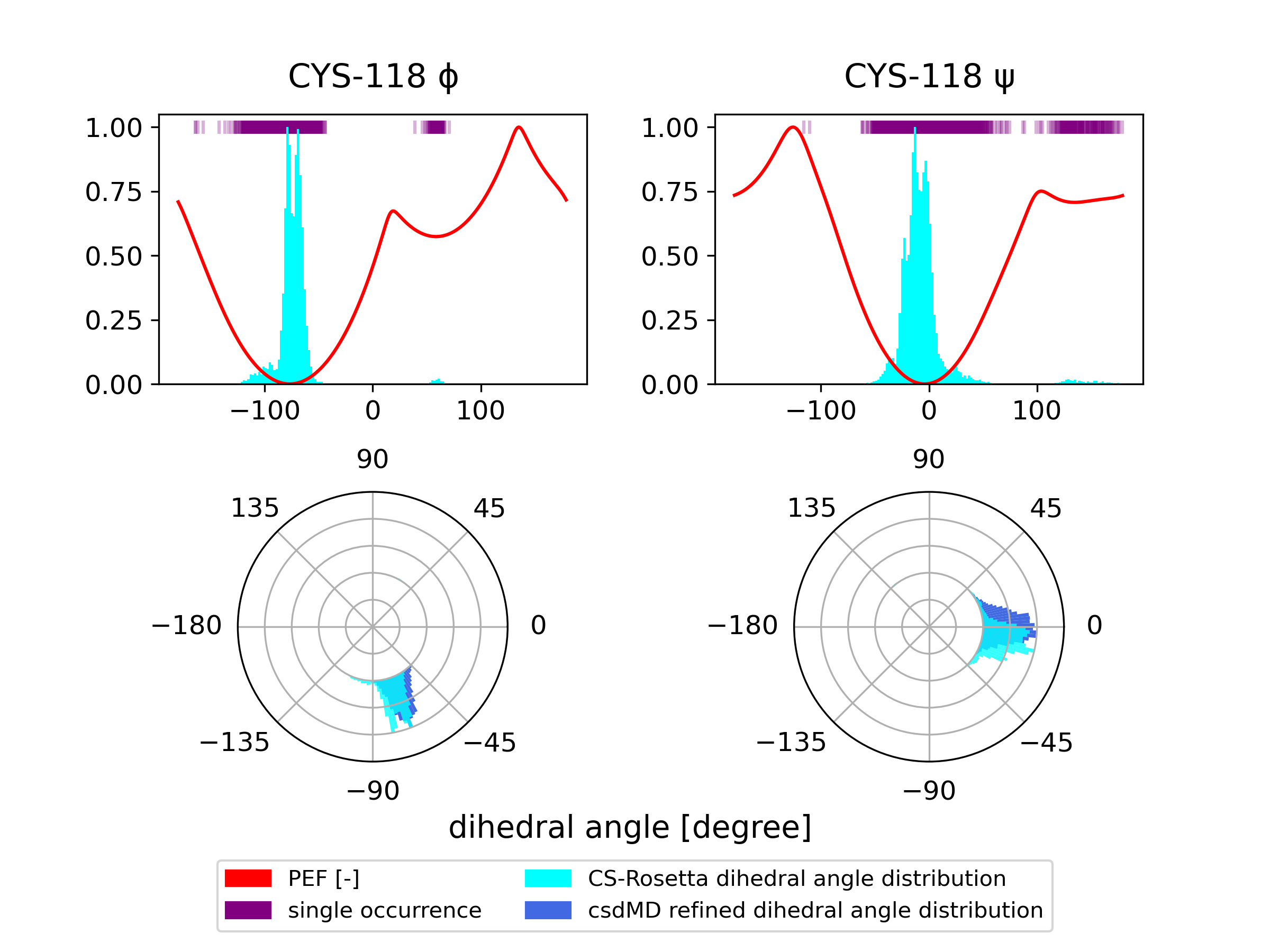

Supplement: Supplementary file 1 [file ijms-24-12101-s001.zip › KRAS-G12C-GDP-Mg-free_angle_figures/118-CYS.png]

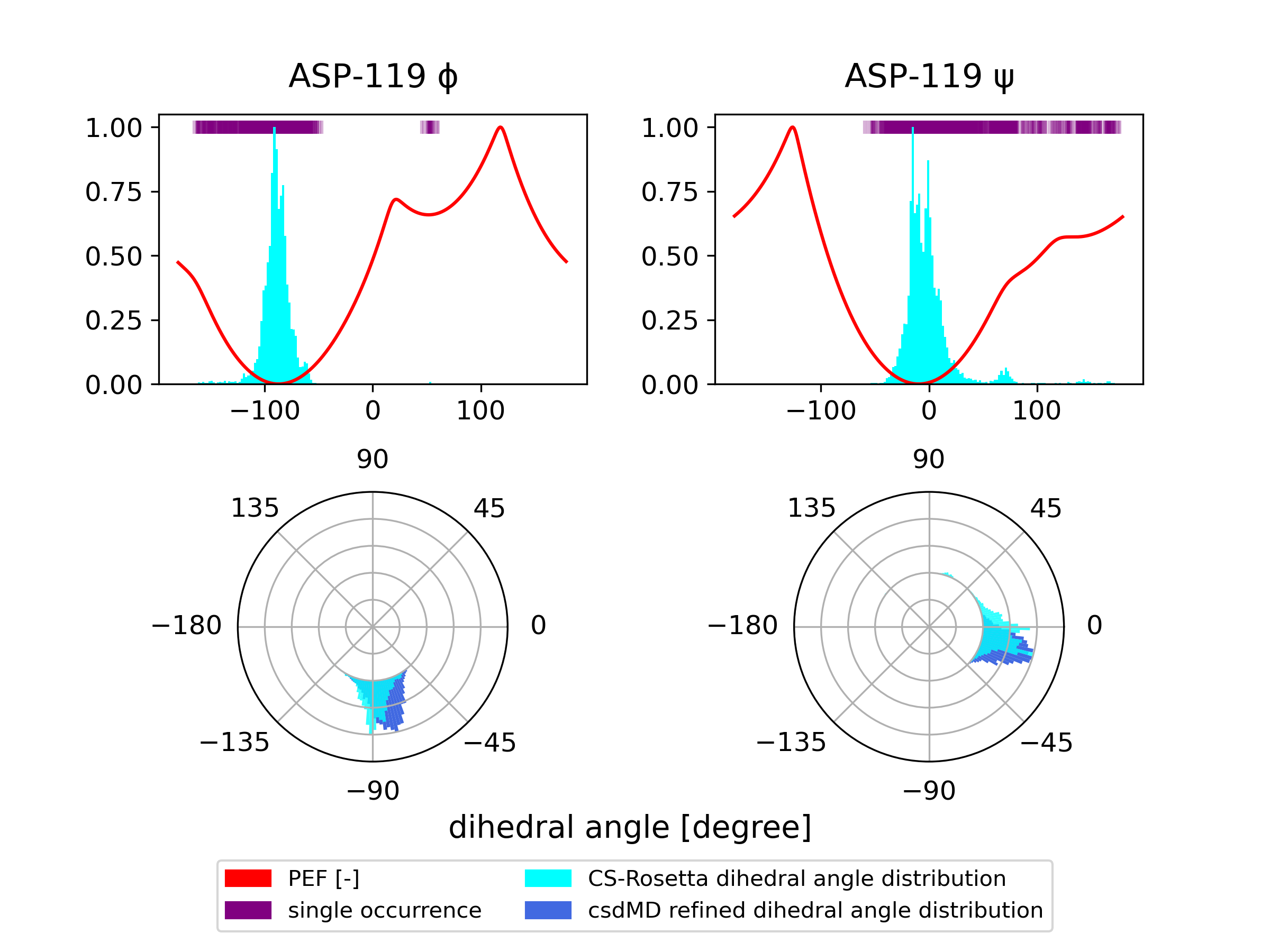

Supplement: Supplementary file 1 [file ijms-24-12101-s001.zip › KRAS-G12C-GDP-Mg-free_angle_figures/119-ASP.png]

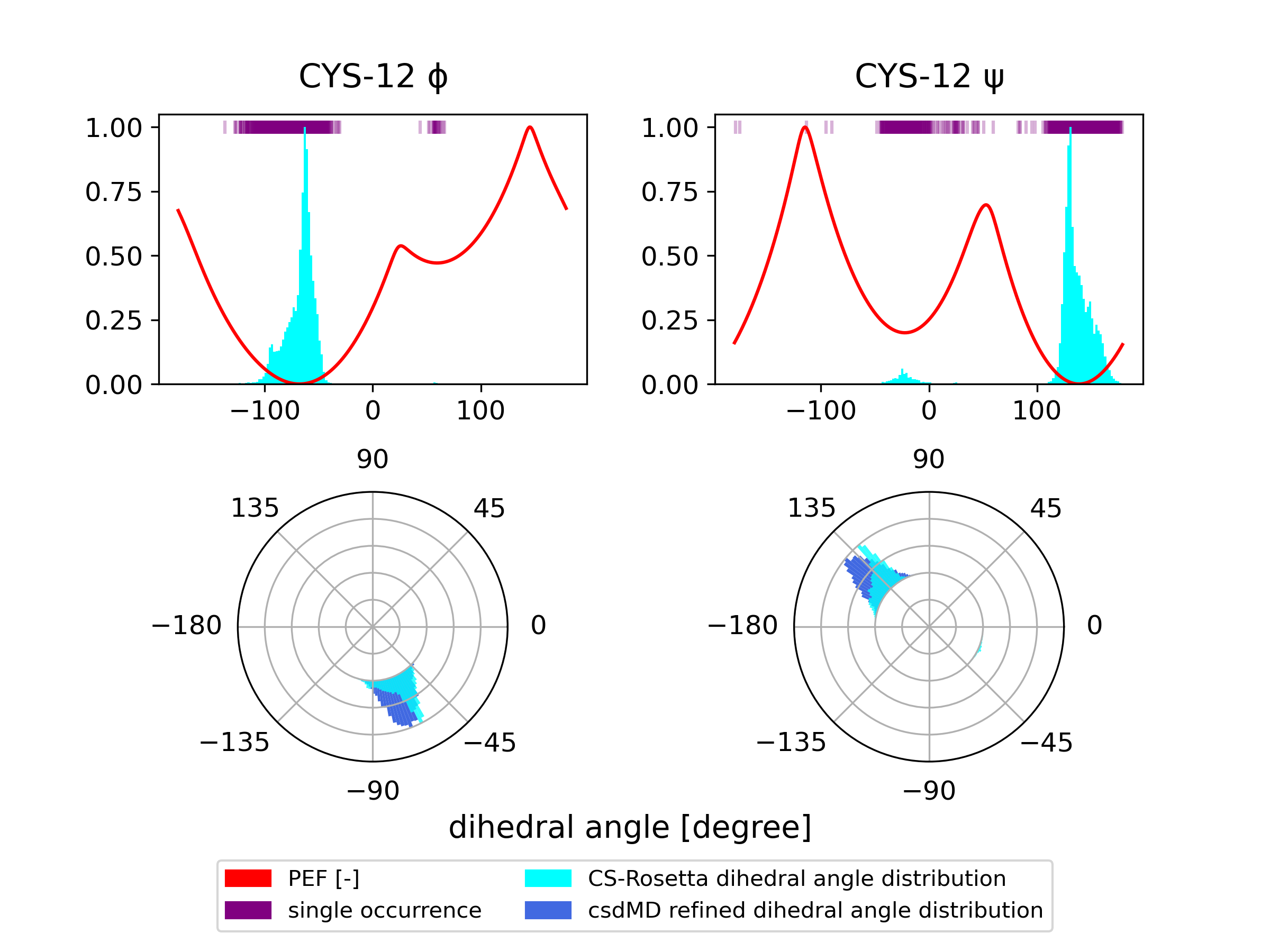

Supplement: Supplementary file 1 [file ijms-24-12101-s001.zip › KRAS-G12C-GDP-Mg-free_angle_figures/12-CYS.png]

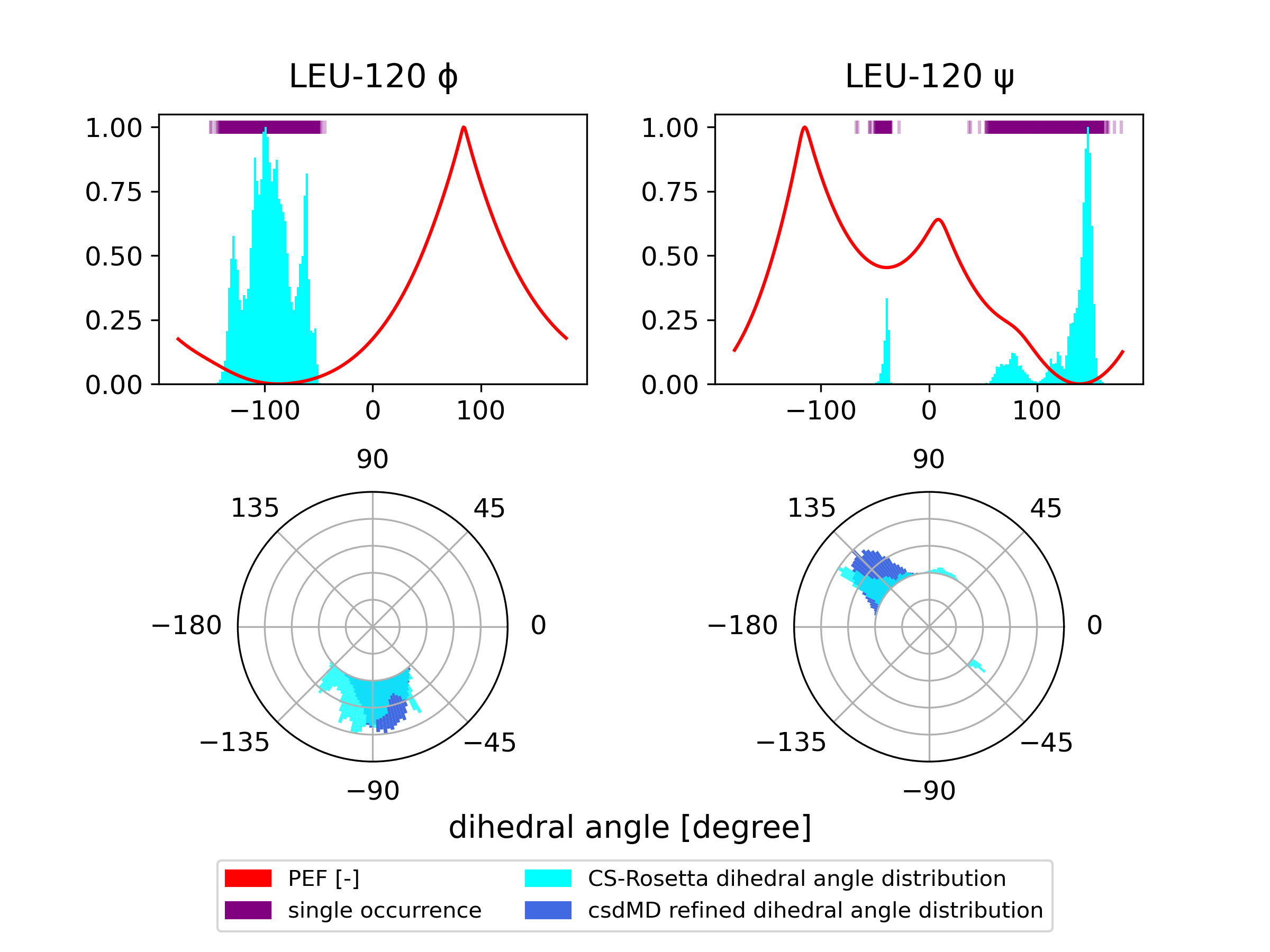

Supplement: Supplementary file 1 [file ijms-24-12101-s001.zip › KRAS-G12C-GDP-Mg-free_angle_figures/120-LEU.png]

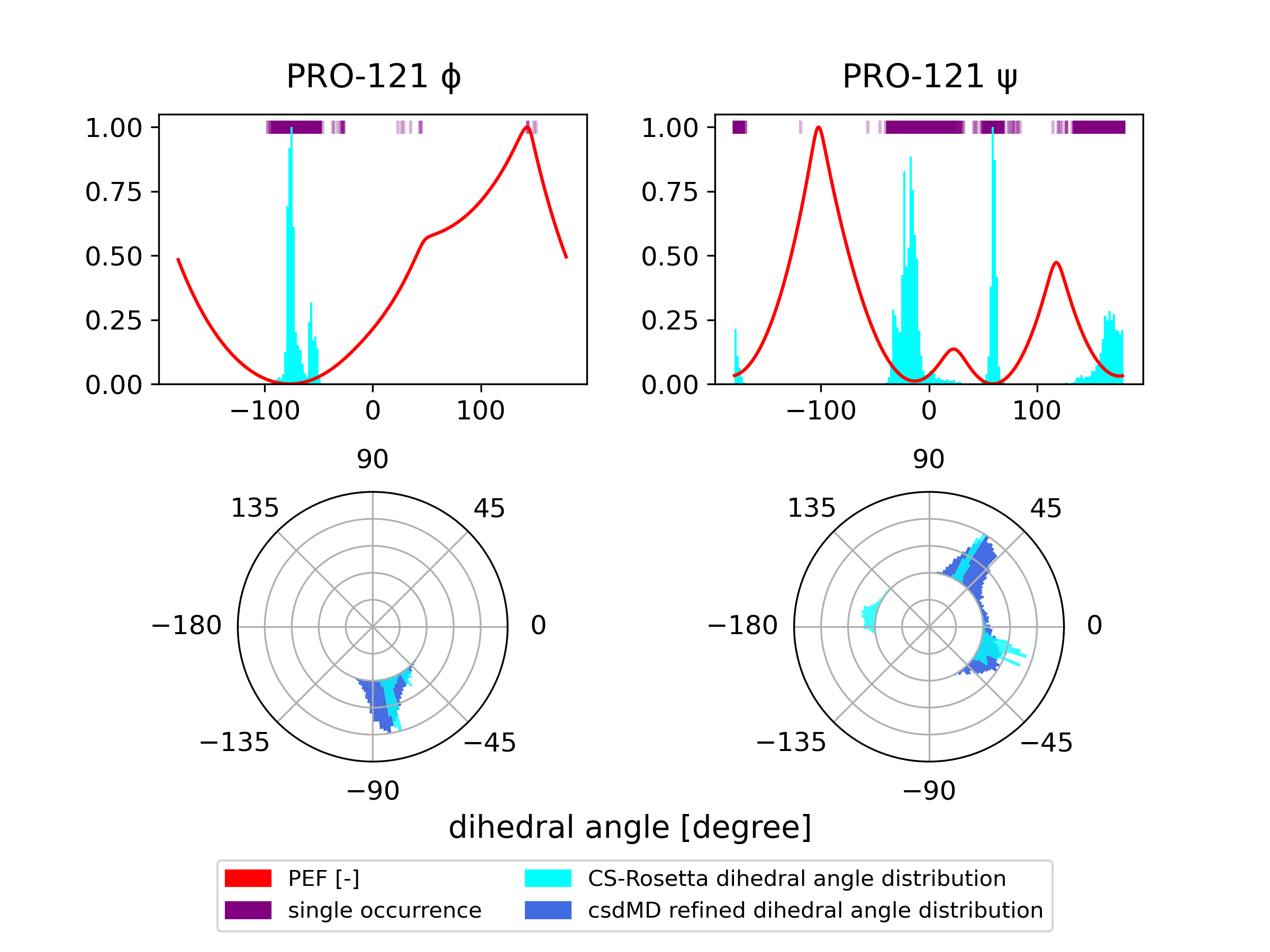

Supplement: Supplementary file 1 [file ijms-24-12101-s001.zip › KRAS-G12C-GDP-Mg-free_angle_figures/121-PRO.png]

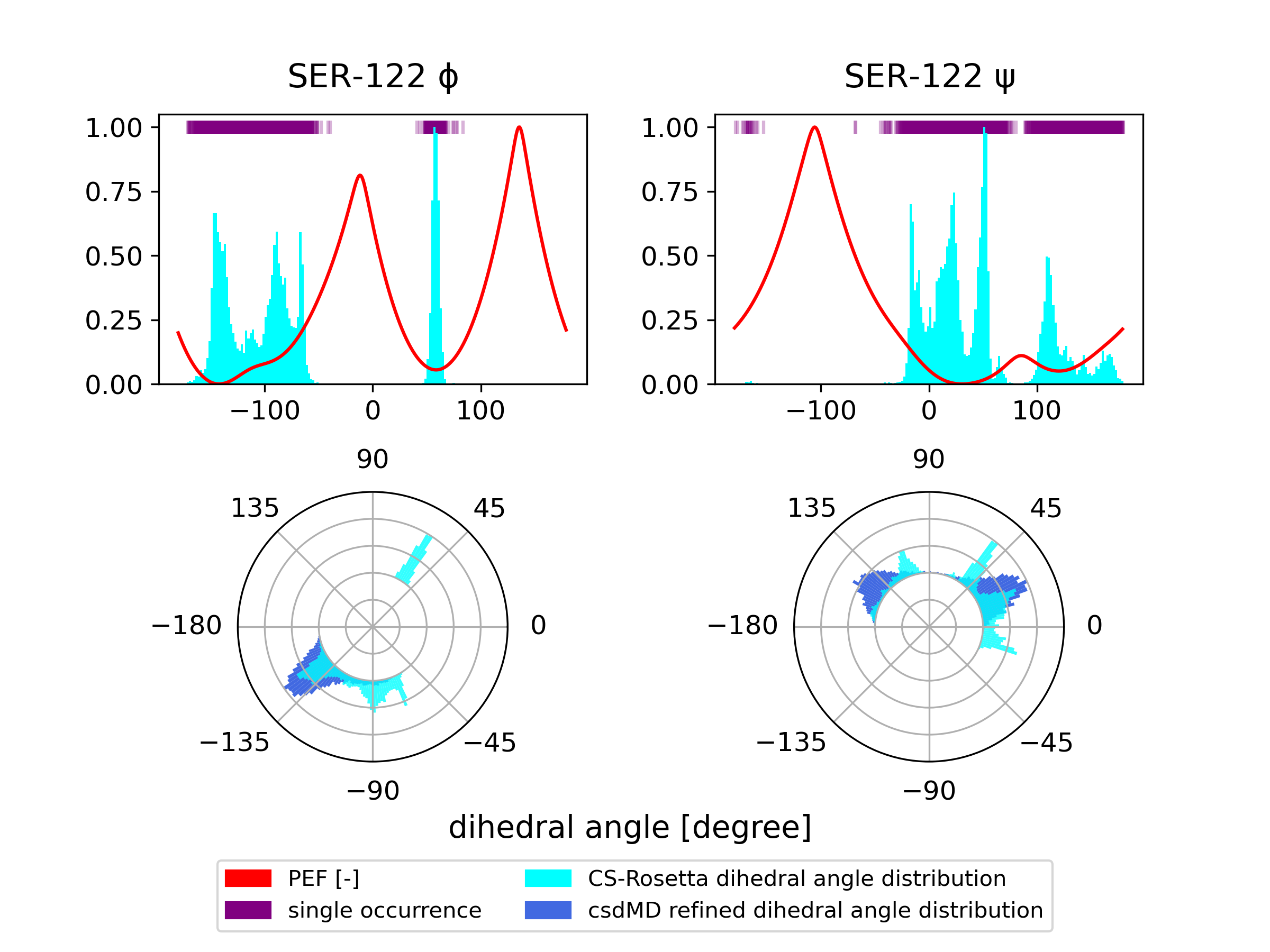

Supplement: Supplementary file 1 [file ijms-24-12101-s001.zip › KRAS-G12C-GDP-Mg-free_angle_figures/122-SER.png]

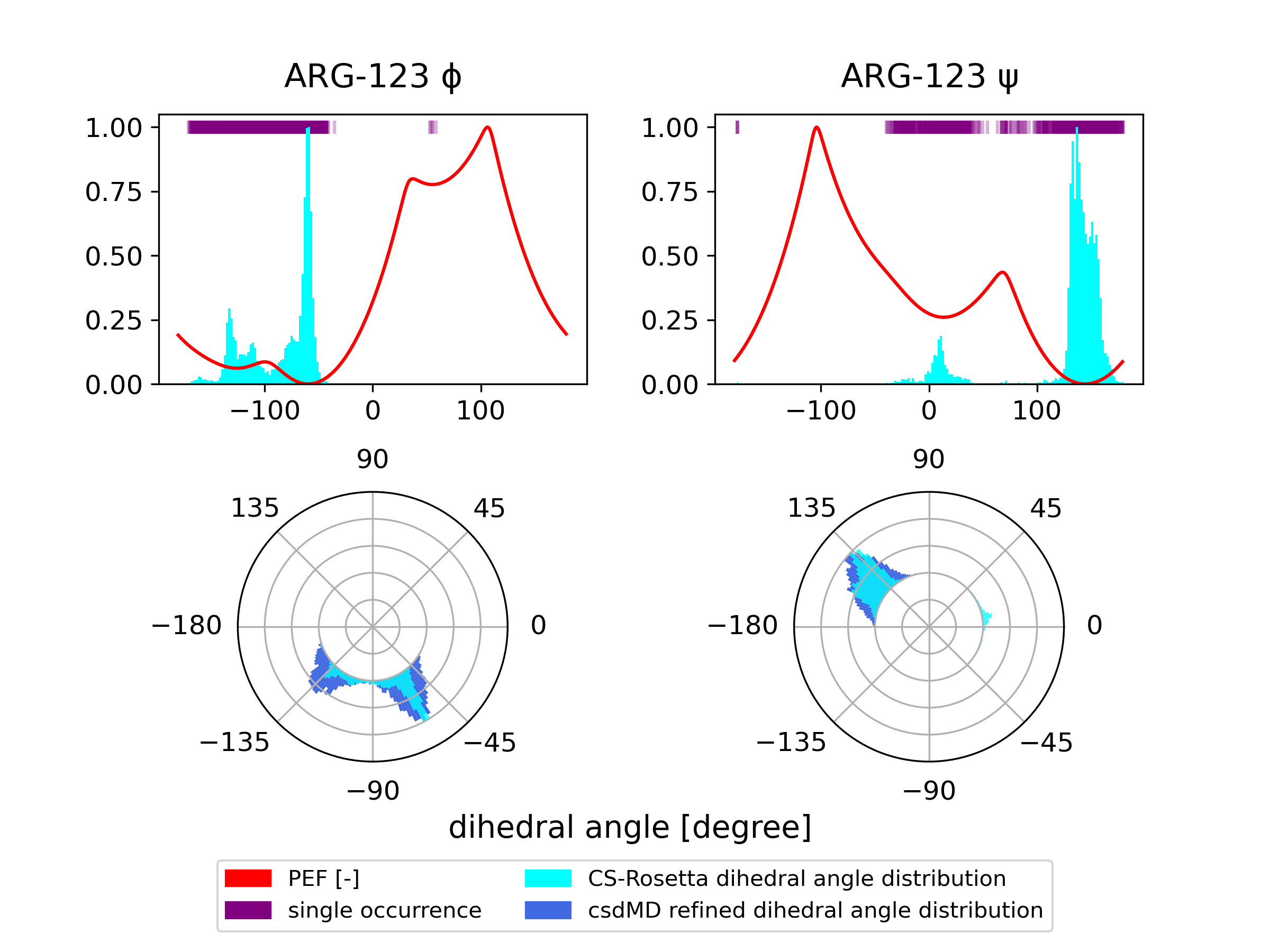

Supplement: Supplementary file 1 [file ijms-24-12101-s001.zip › KRAS-G12C-GDP-Mg-free_angle_figures/123-ARG.png]

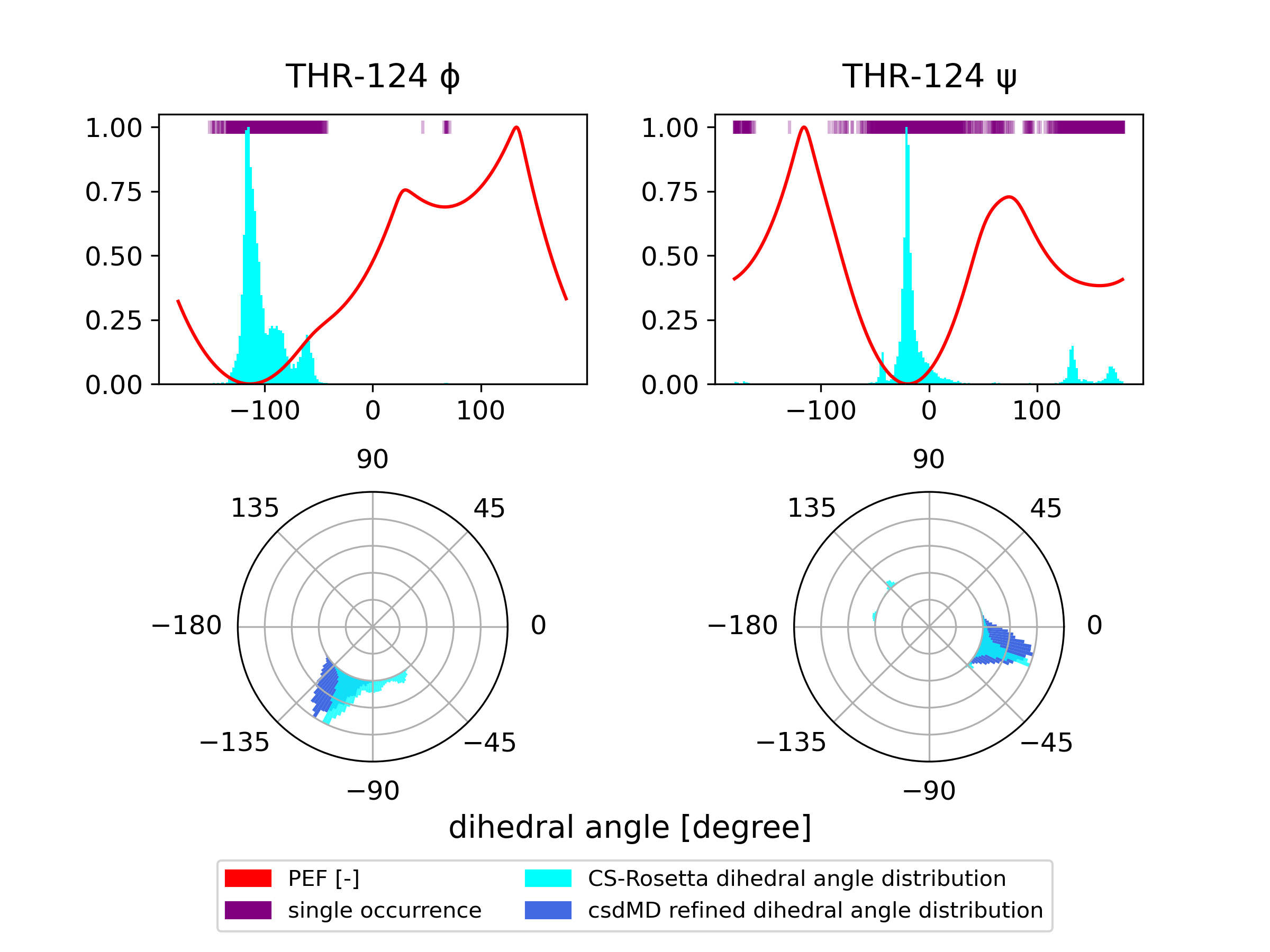

Supplement: Supplementary file 1 [file ijms-24-12101-s001.zip › KRAS-G12C-GDP-Mg-free_angle_figures/124-THR.png]

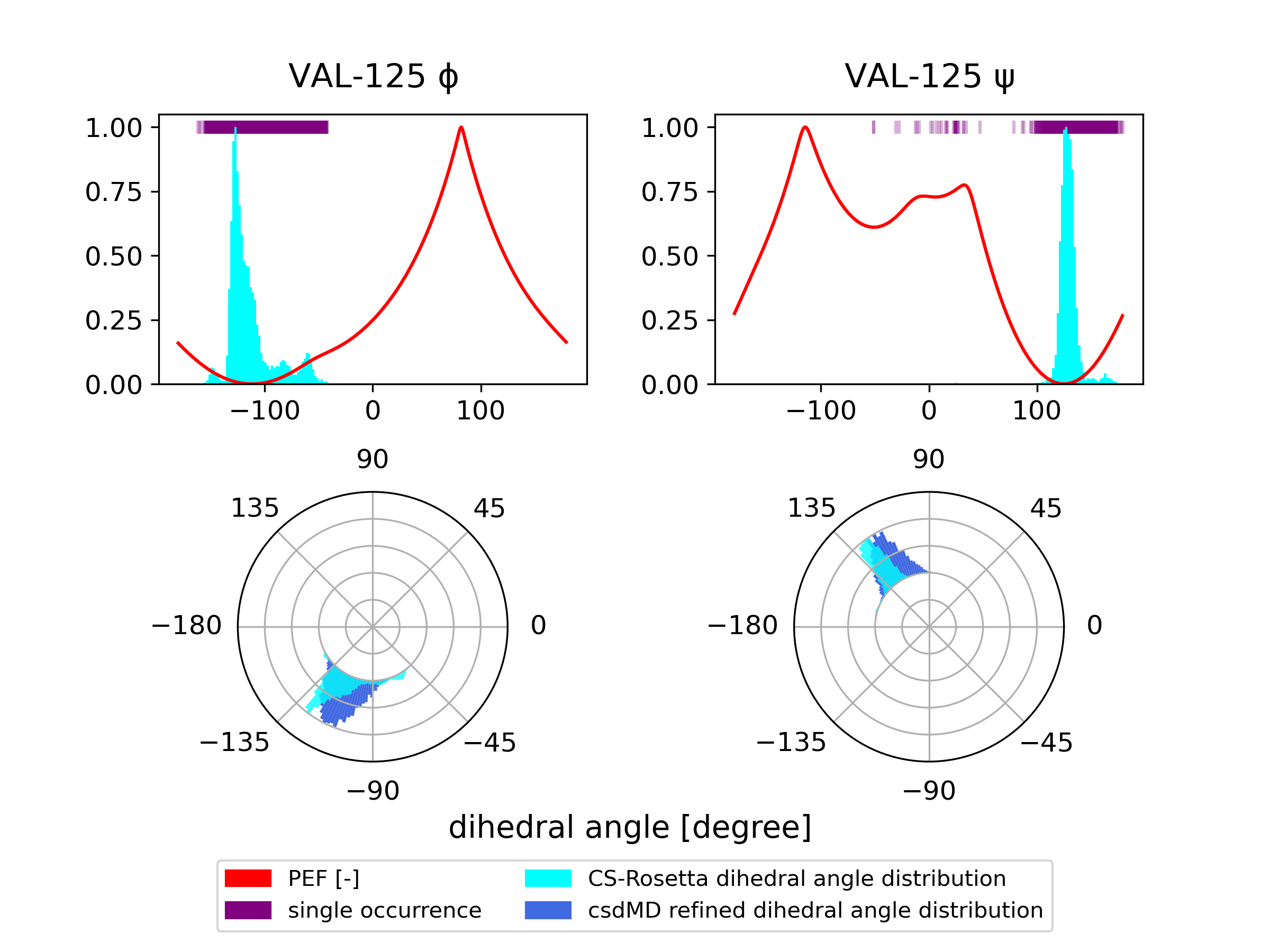

Supplement: Supplementary file 1 [file ijms-24-12101-s001.zip › KRAS-G12C-GDP-Mg-free_angle_figures/125-VAL.png]

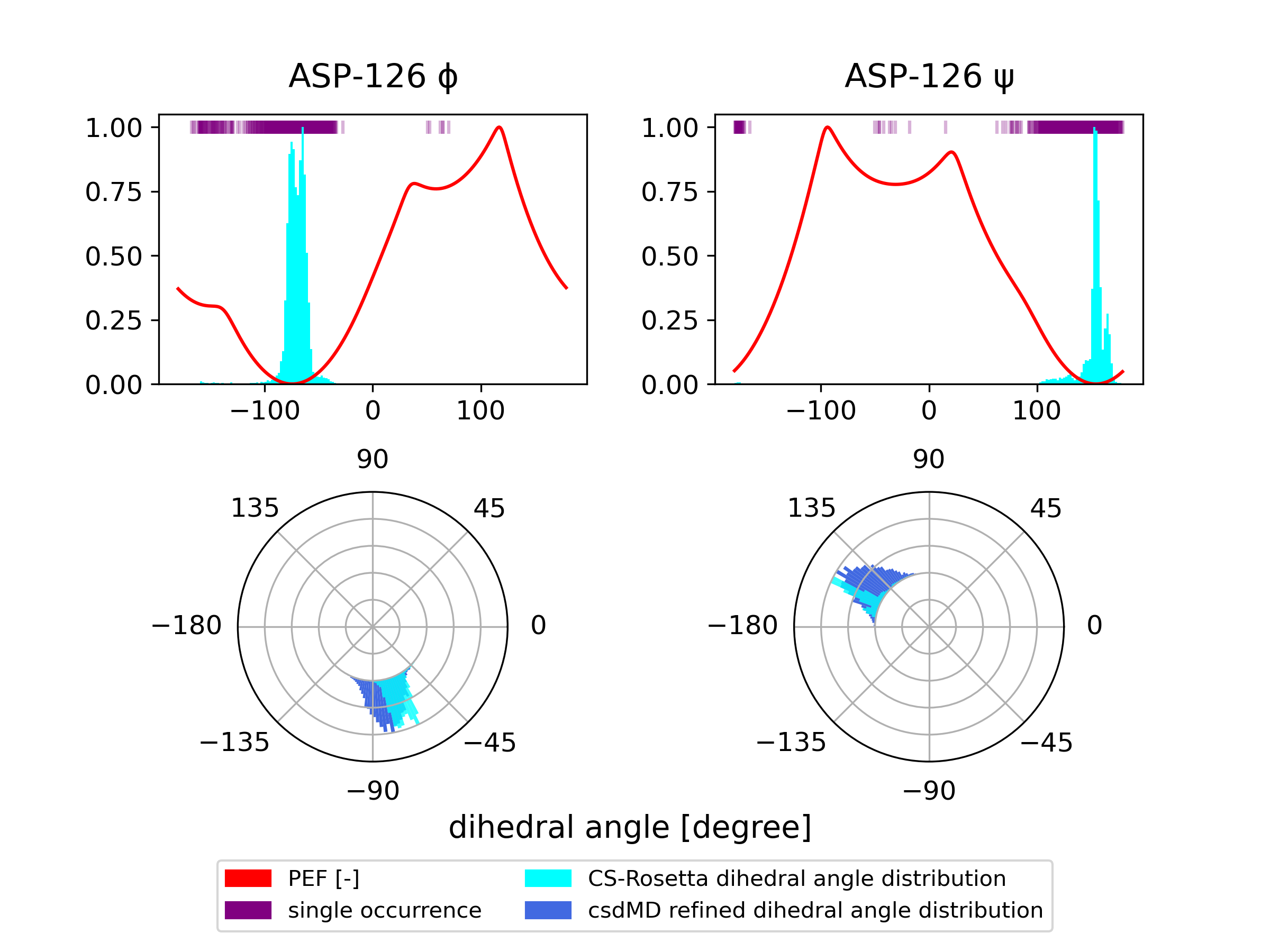

Supplement: Supplementary file 1 [file ijms-24-12101-s001.zip › KRAS-G12C-GDP-Mg-free_angle_figures/126-ASP.png]

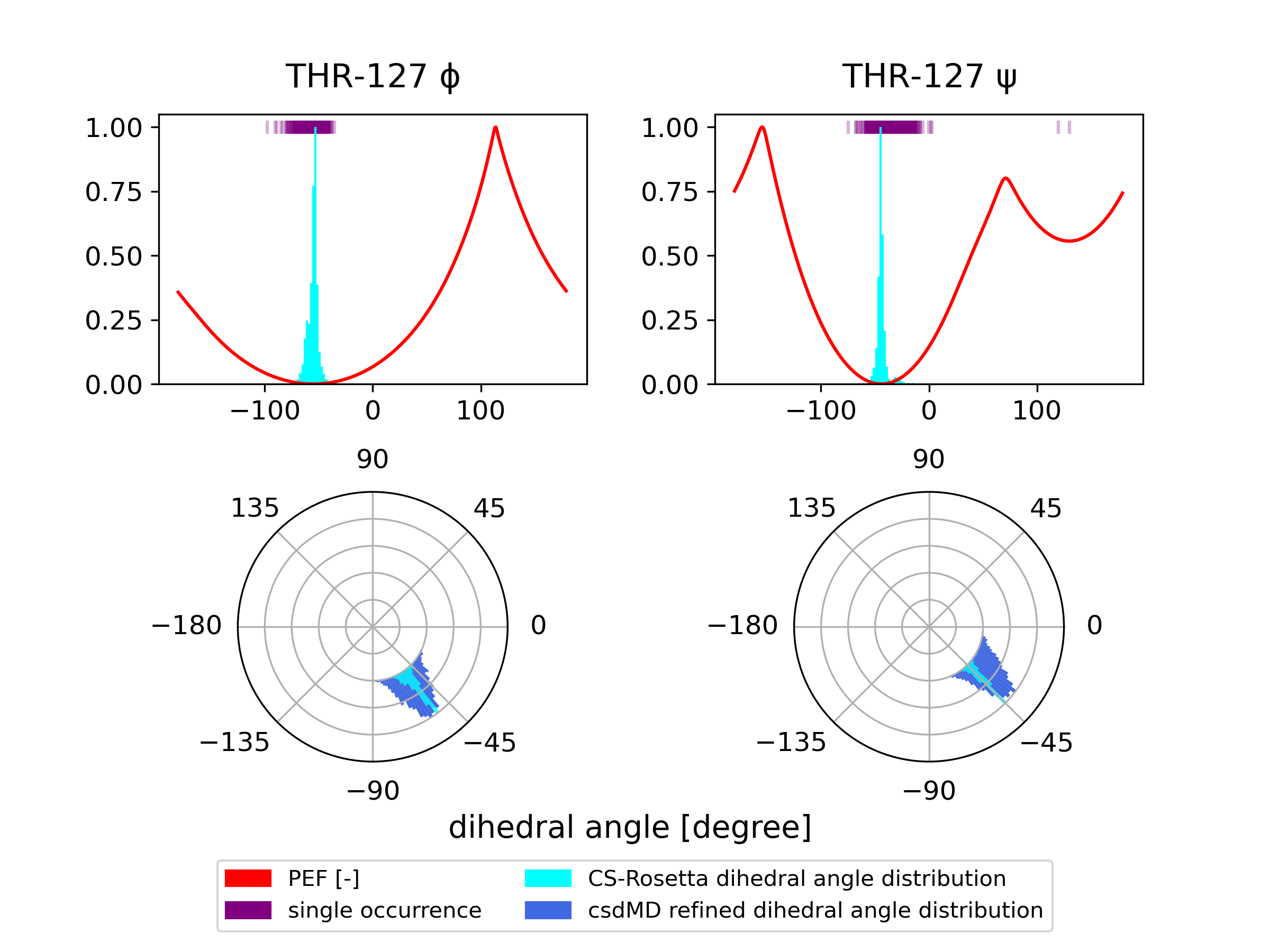

Supplement: Supplementary file 1 [file ijms-24-12101-s001.zip › KRAS-G12C-GDP-Mg-free_angle_figures/127-THR.png]

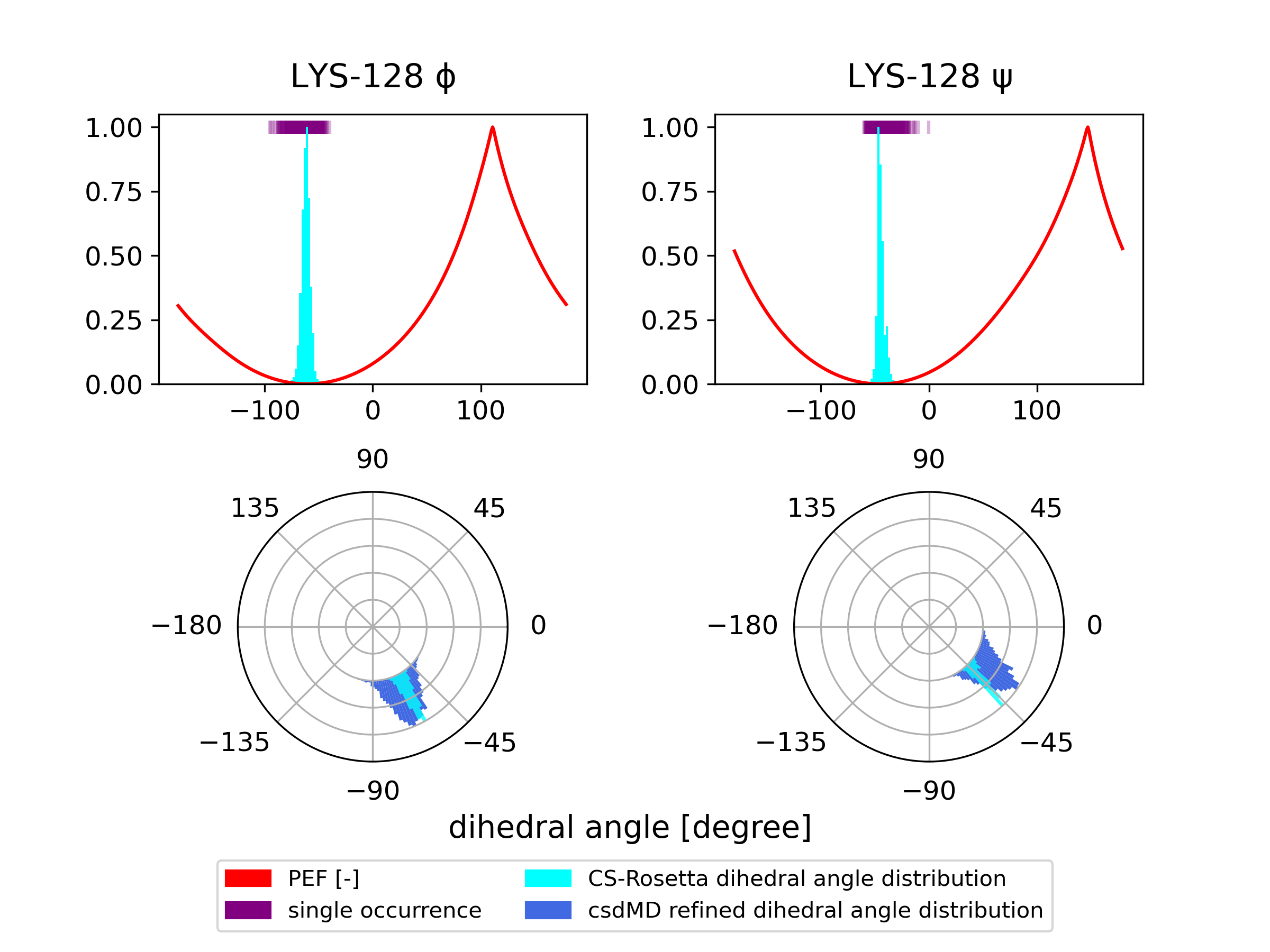

Supplement: Supplementary file 1 [file ijms-24-12101-s001.zip › KRAS-G12C-GDP-Mg-free_angle_figures/128-LYS.png]

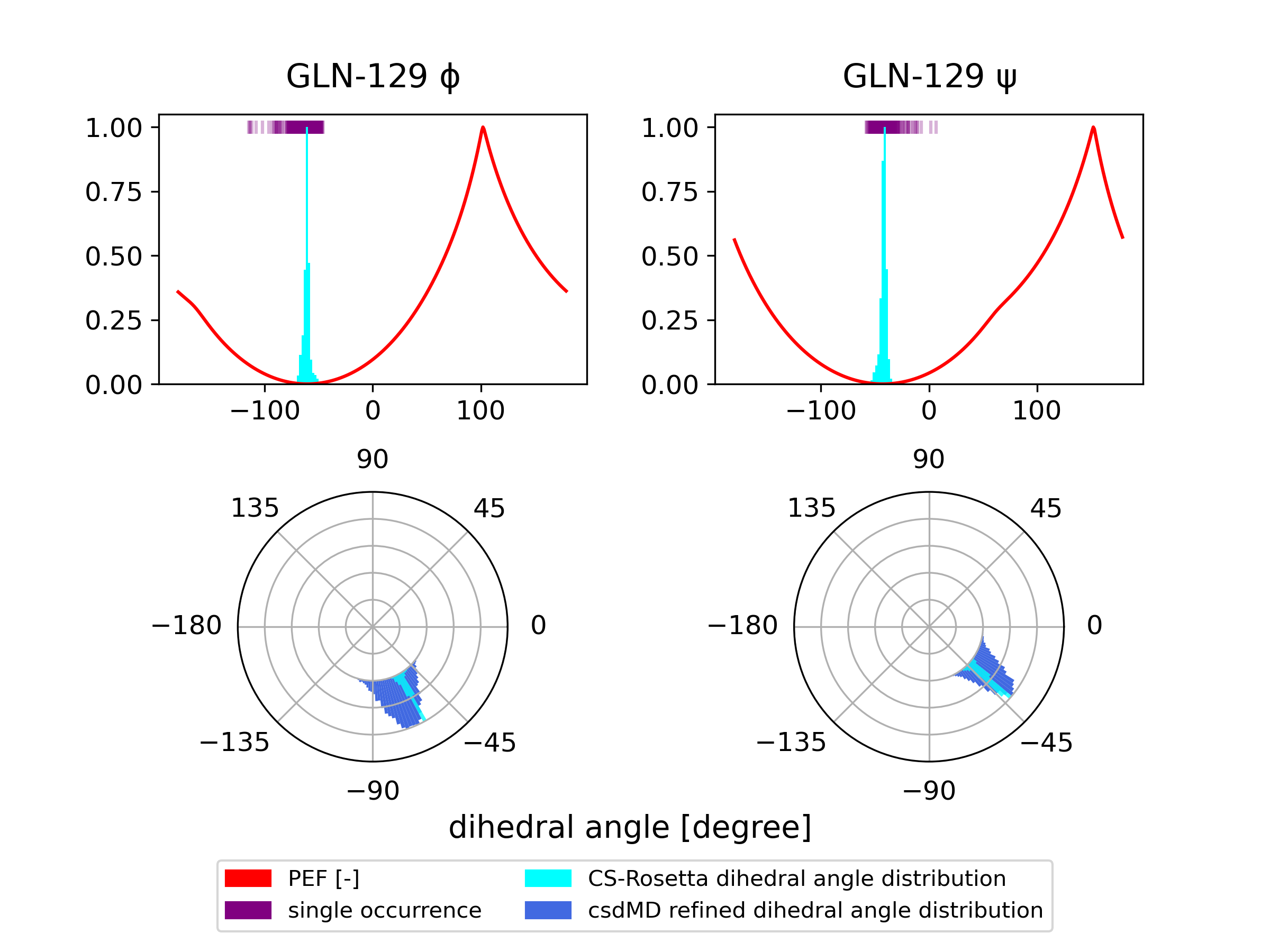

Supplement: Supplementary file 1 [file ijms-24-12101-s001.zip › KRAS-G12C-GDP-Mg-free_angle_figures/129-GLN.png]

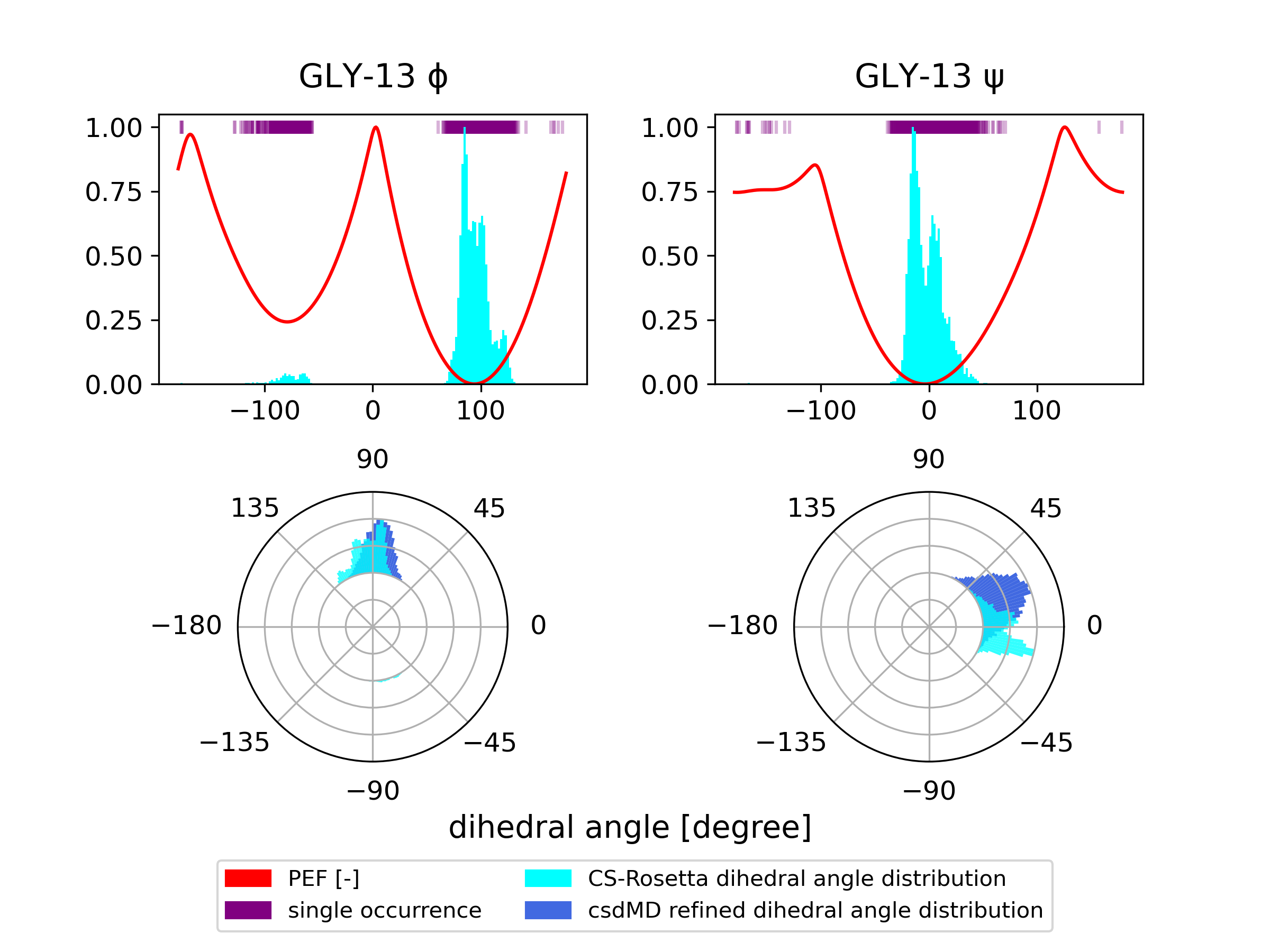

Supplement: Supplementary file 1 [file ijms-24-12101-s001.zip › KRAS-G12C-GDP-Mg-free_angle_figures/13-GLY.png]

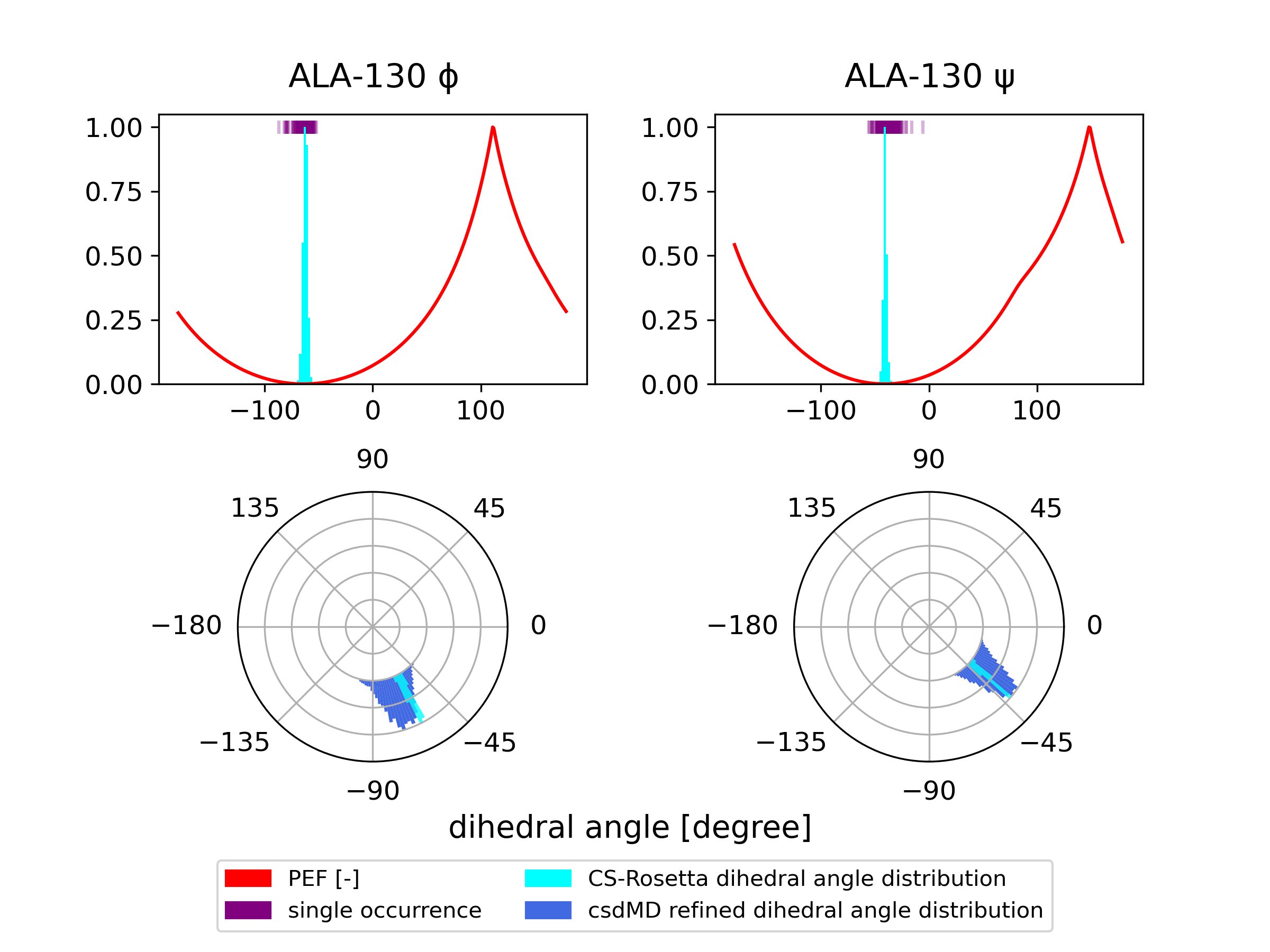

Supplement: Supplementary file 1 [file ijms-24-12101-s001.zip › KRAS-G12C-GDP-Mg-free_angle_figures/130-ALA.png]

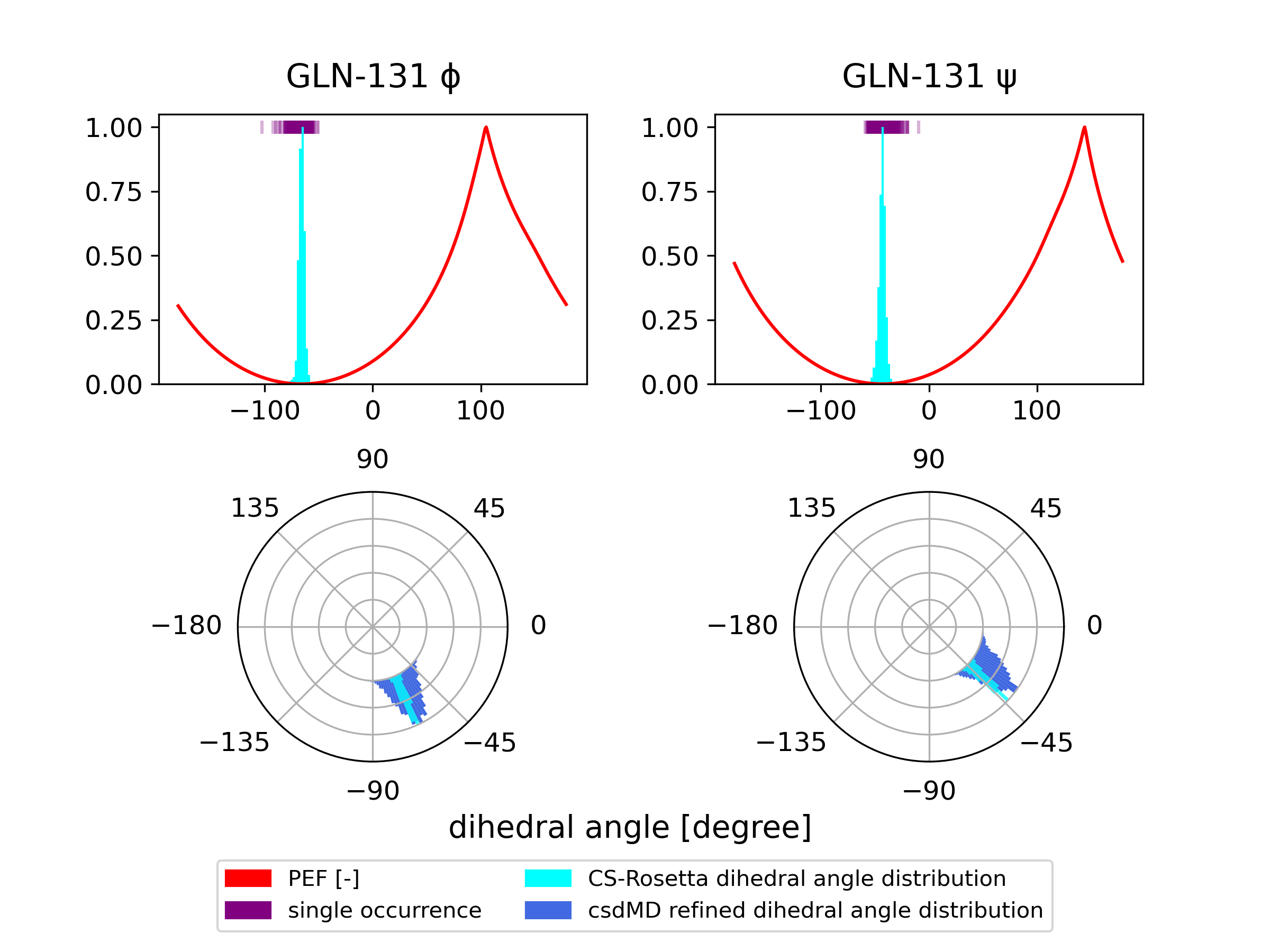

Supplement: Supplementary file 1 [file ijms-24-12101-s001.zip › KRAS-G12C-GDP-Mg-free_angle_figures/131-GLN.png]

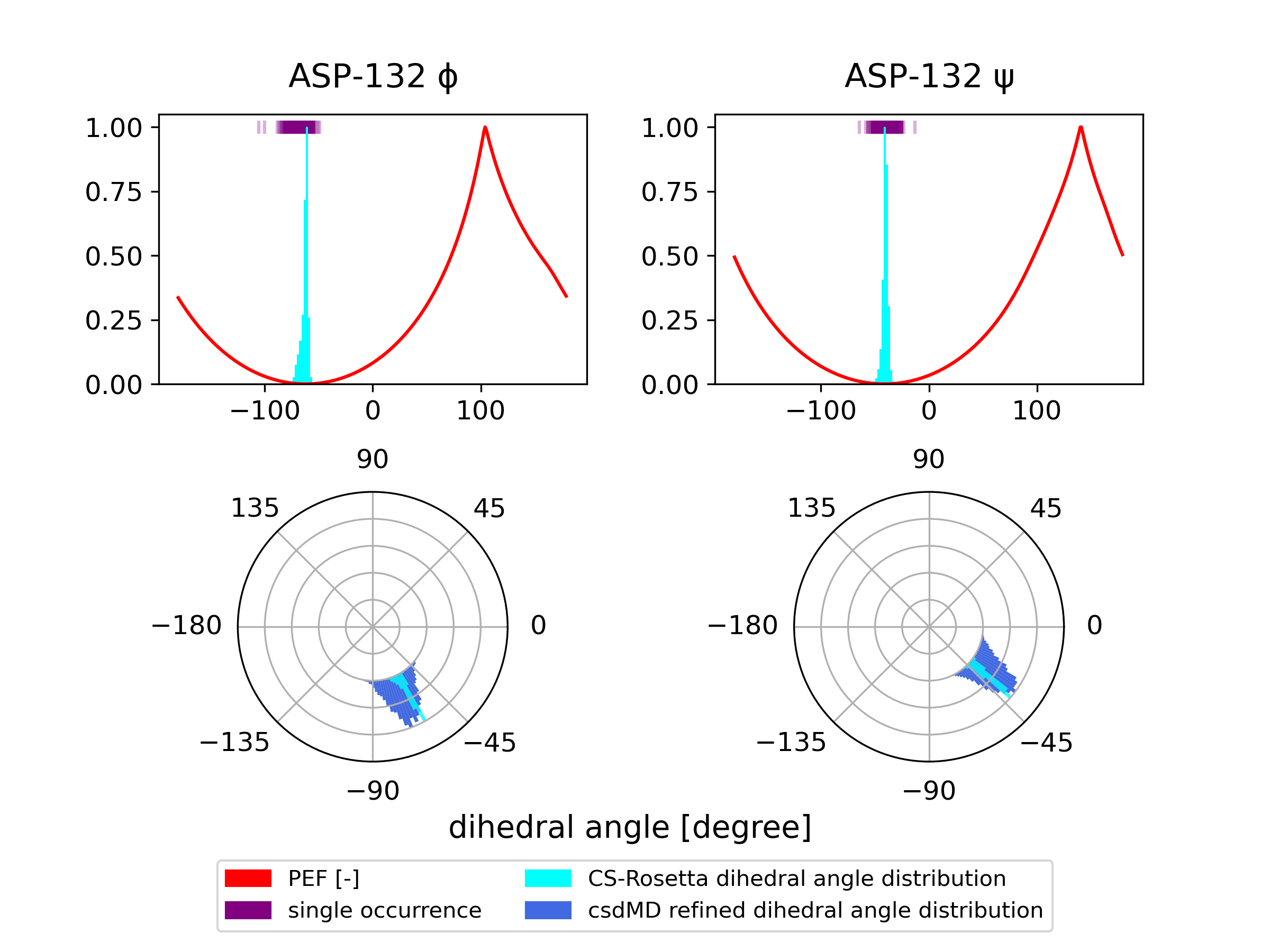

Supplement: Supplementary file 1 [file ijms-24-12101-s001.zip › KRAS-G12C-GDP-Mg-free_angle_figures/132-ASP.png]

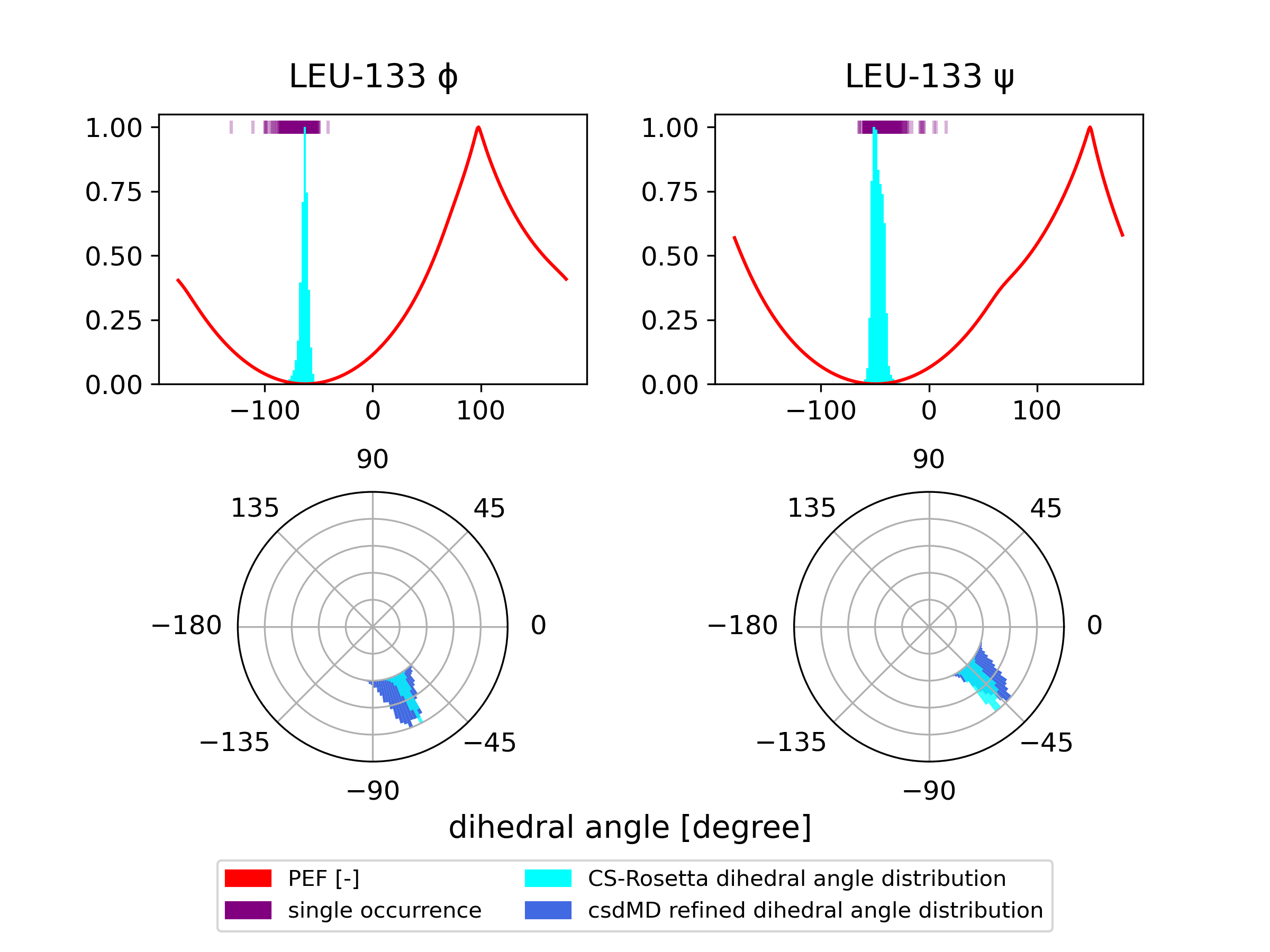

Supplement: Supplementary file 1 [file ijms-24-12101-s001.zip › KRAS-G12C-GDP-Mg-free_angle_figures/133-LEU.png]

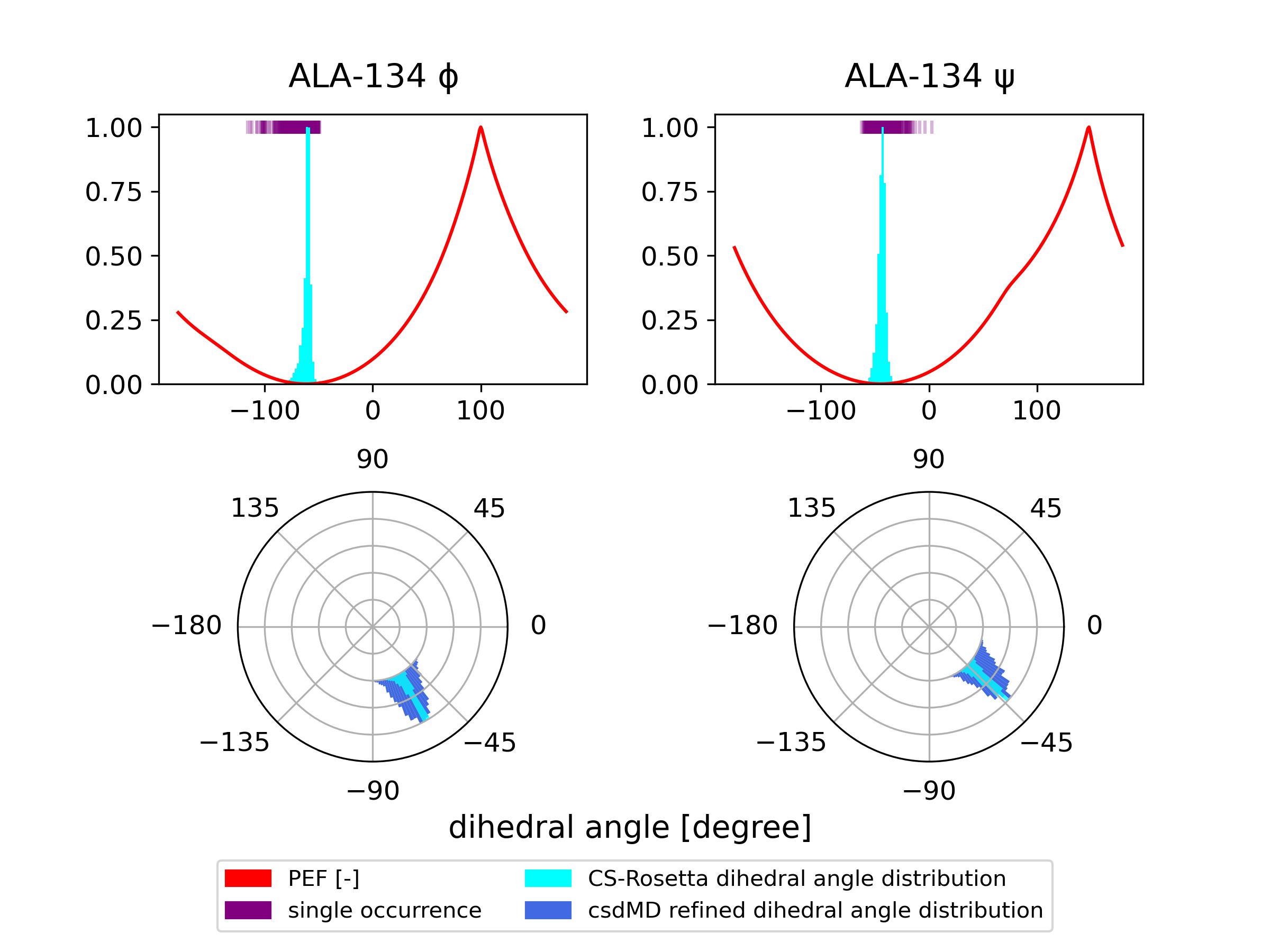

Supplement: Supplementary file 1 [file ijms-24-12101-s001.zip › KRAS-G12C-GDP-Mg-free_angle_figures/134-ALA.png]

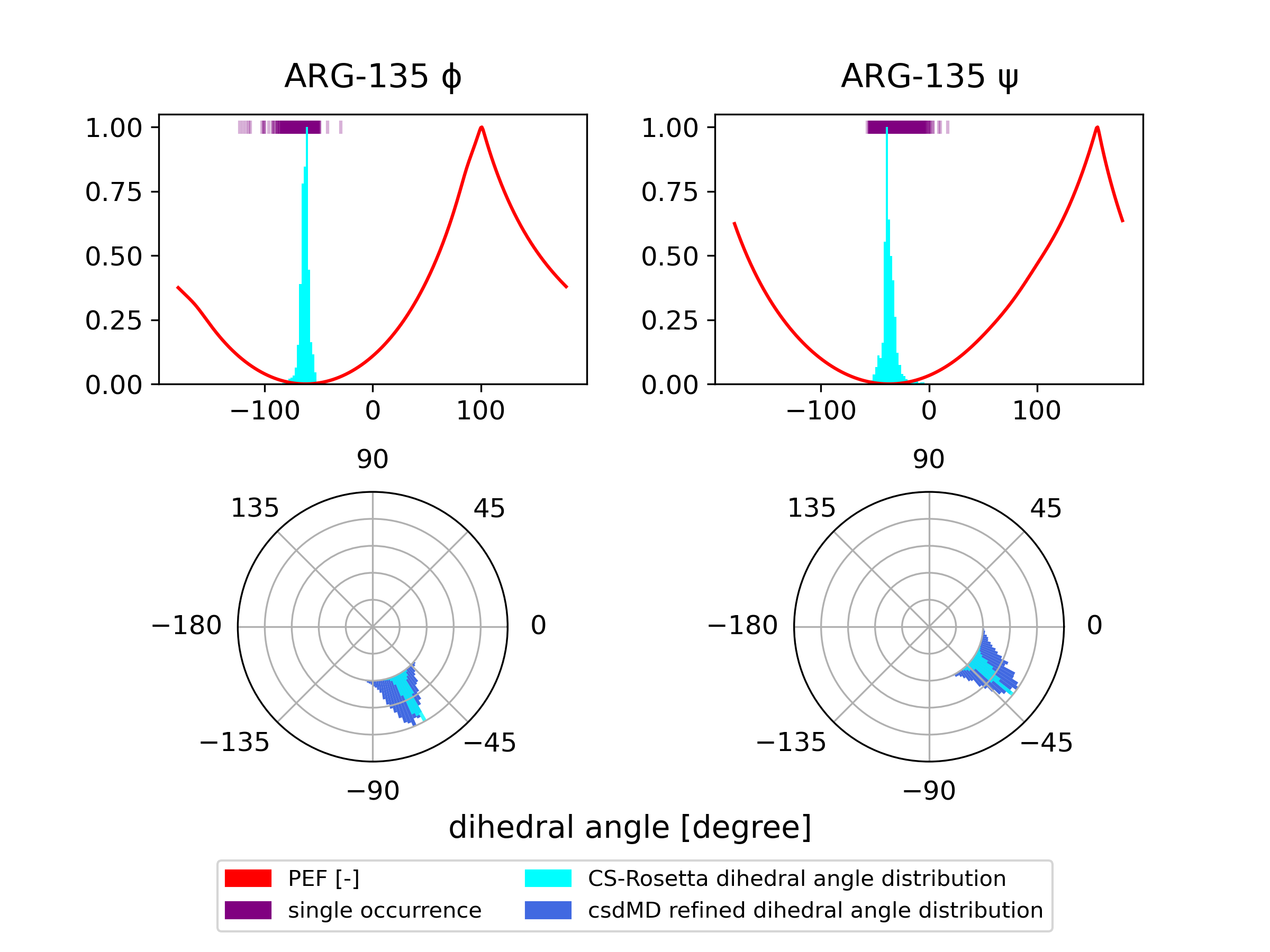

Supplement: Supplementary file 1 [file ijms-24-12101-s001.zip › KRAS-G12C-GDP-Mg-free_angle_figures/135-ARG.png]

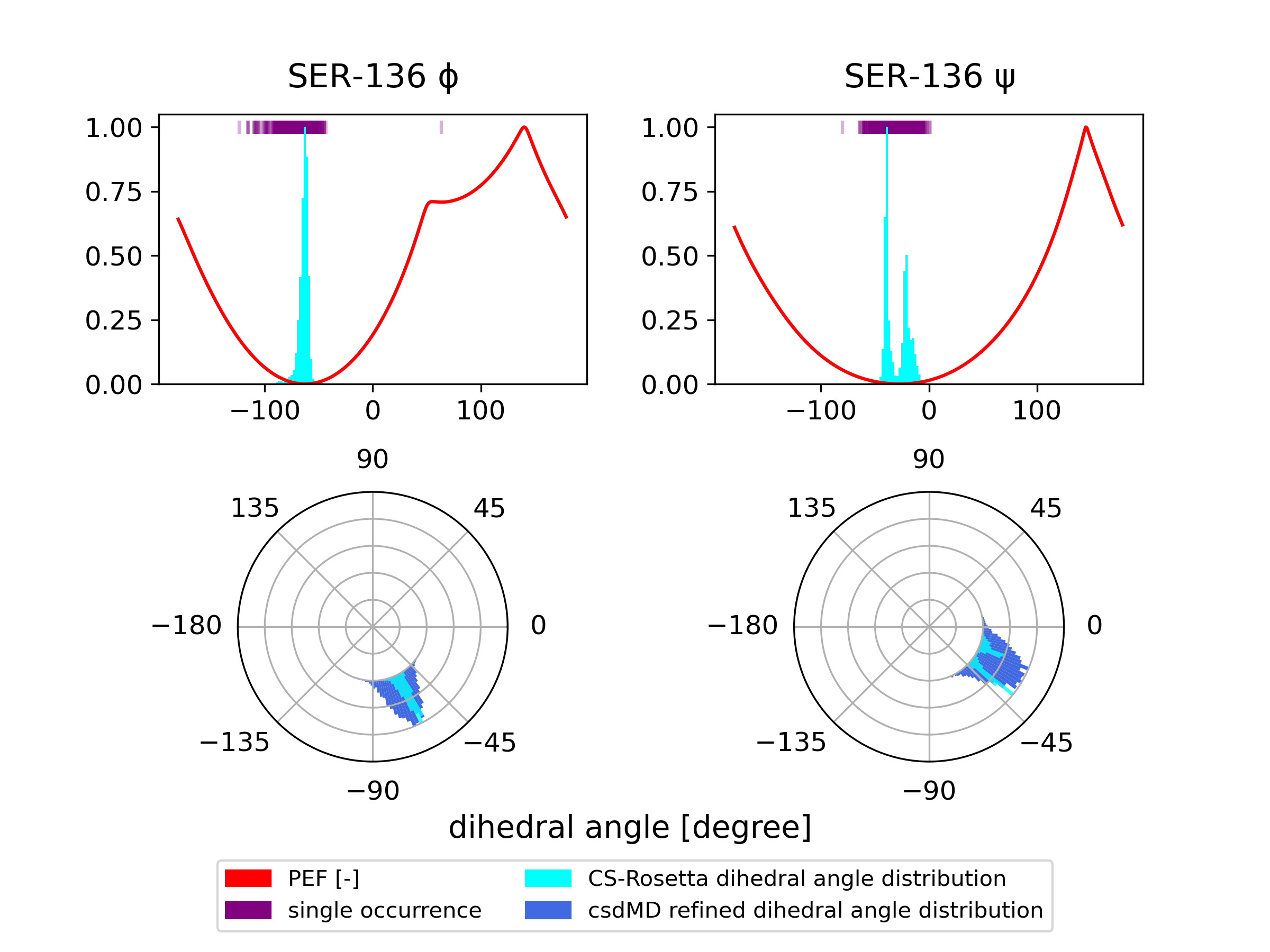

Supplement: Supplementary file 1 [file ijms-24-12101-s001.zip › KRAS-G12C-GDP-Mg-free_angle_figures/136-SER.png]

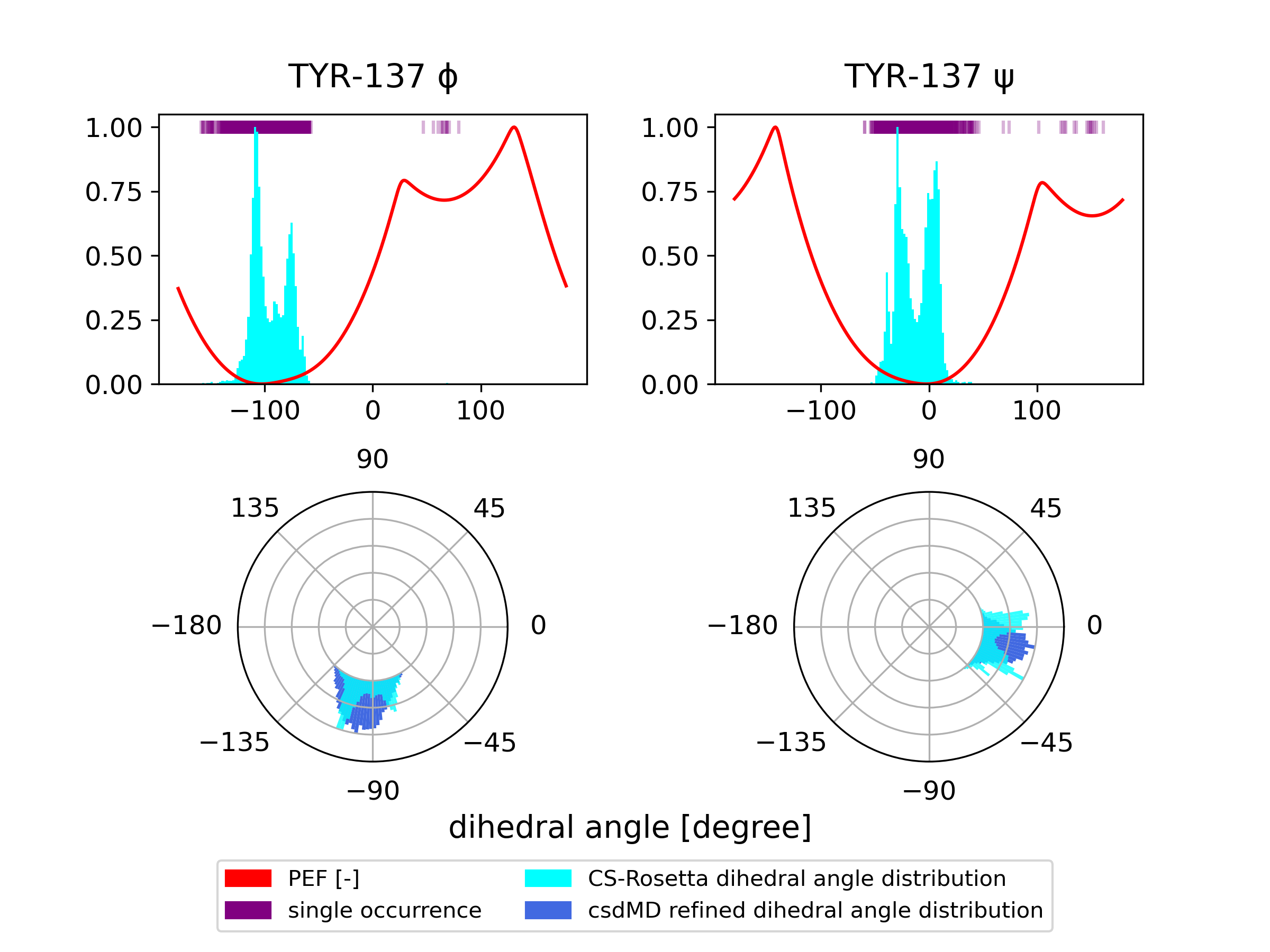

Supplement: Supplementary file 1 [file ijms-24-12101-s001.zip › KRAS-G12C-GDP-Mg-free_angle_figures/137-TYR.png]

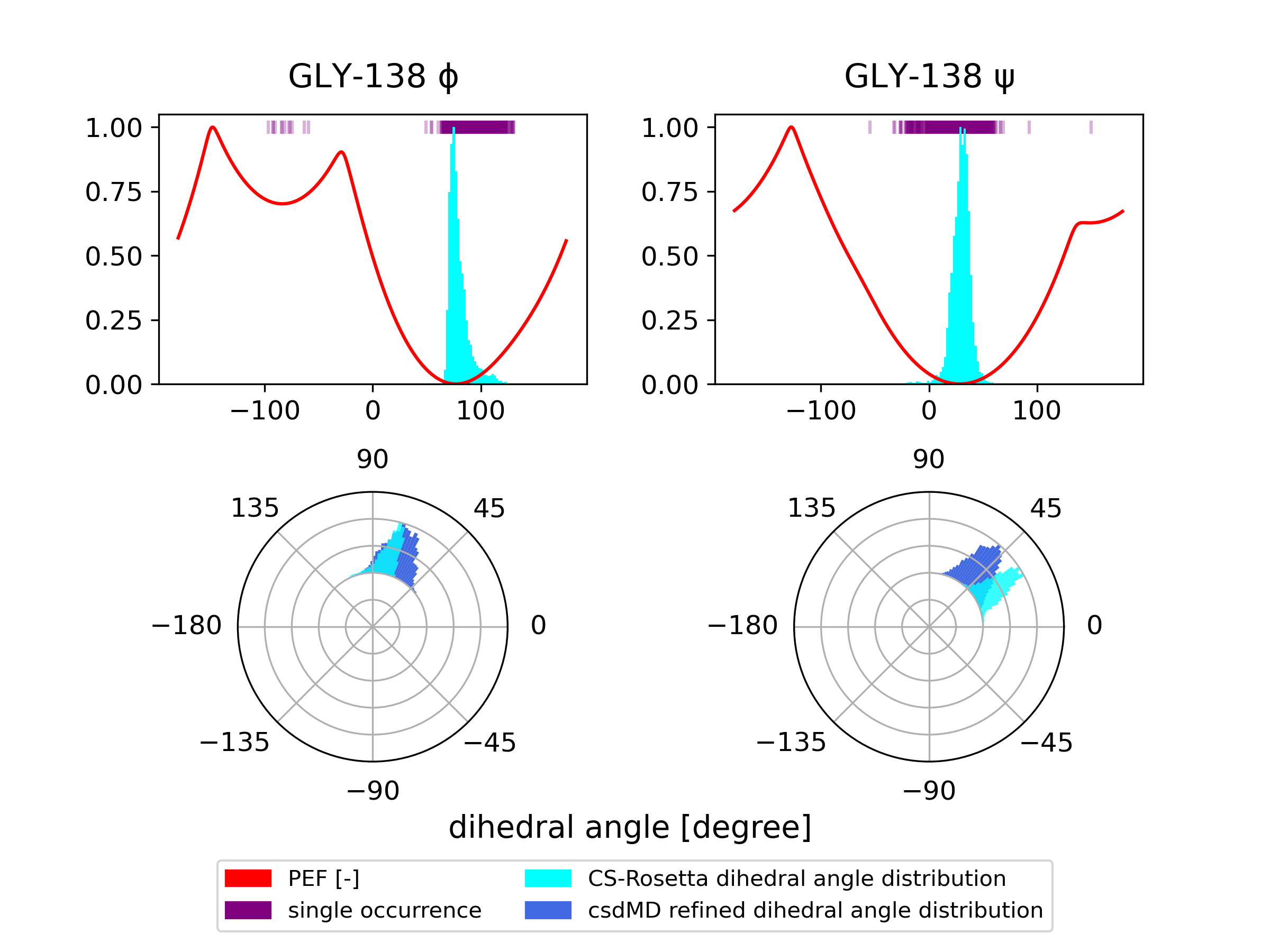

Supplement: Supplementary file 1 [file ijms-24-12101-s001.zip › KRAS-G12C-GDP-Mg-free_angle_figures/138-GLY.png]

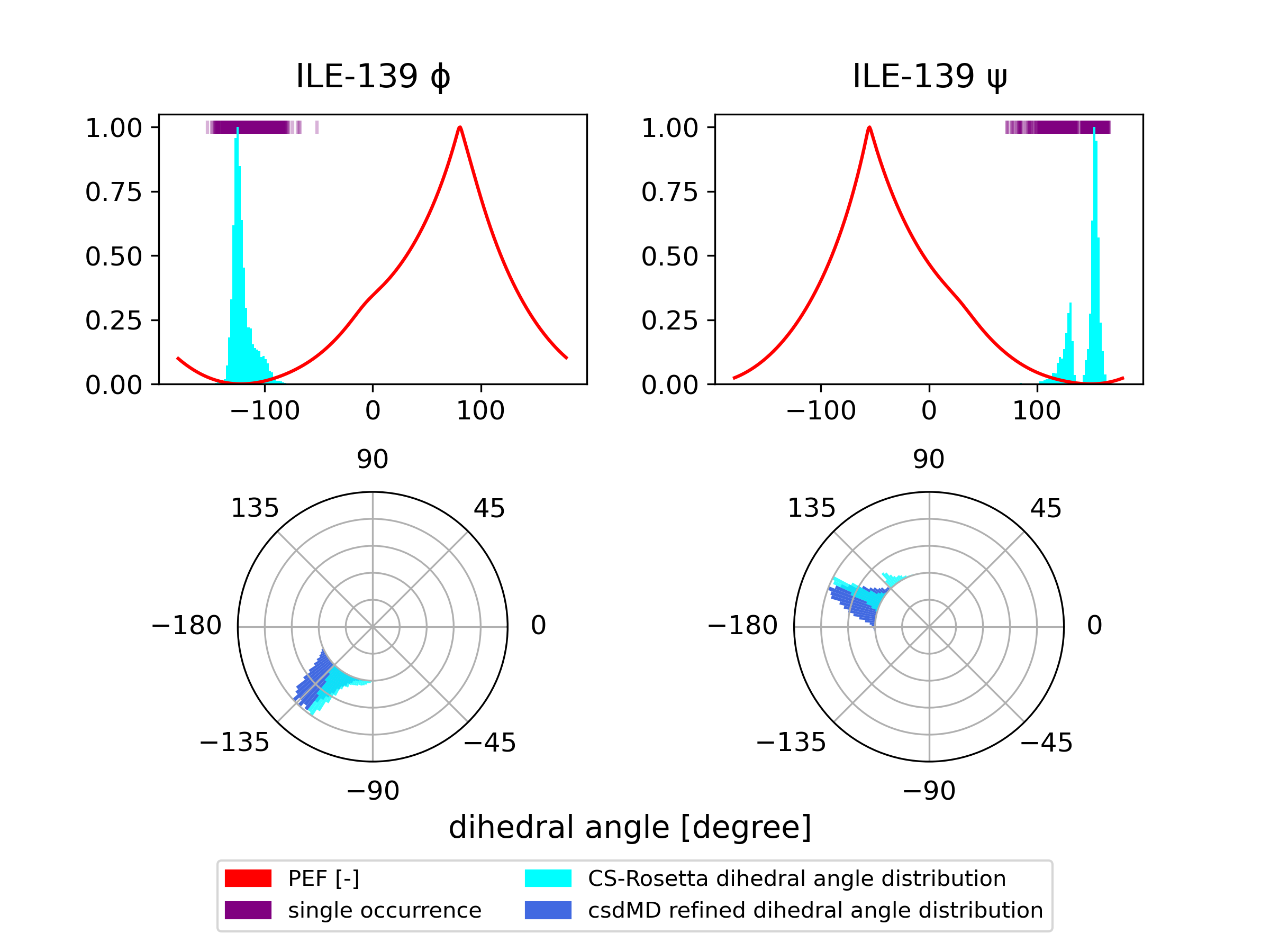

Supplement: Supplementary file 1 [file ijms-24-12101-s001.zip › KRAS-G12C-GDP-Mg-free_angle_figures/139-ILE.png]

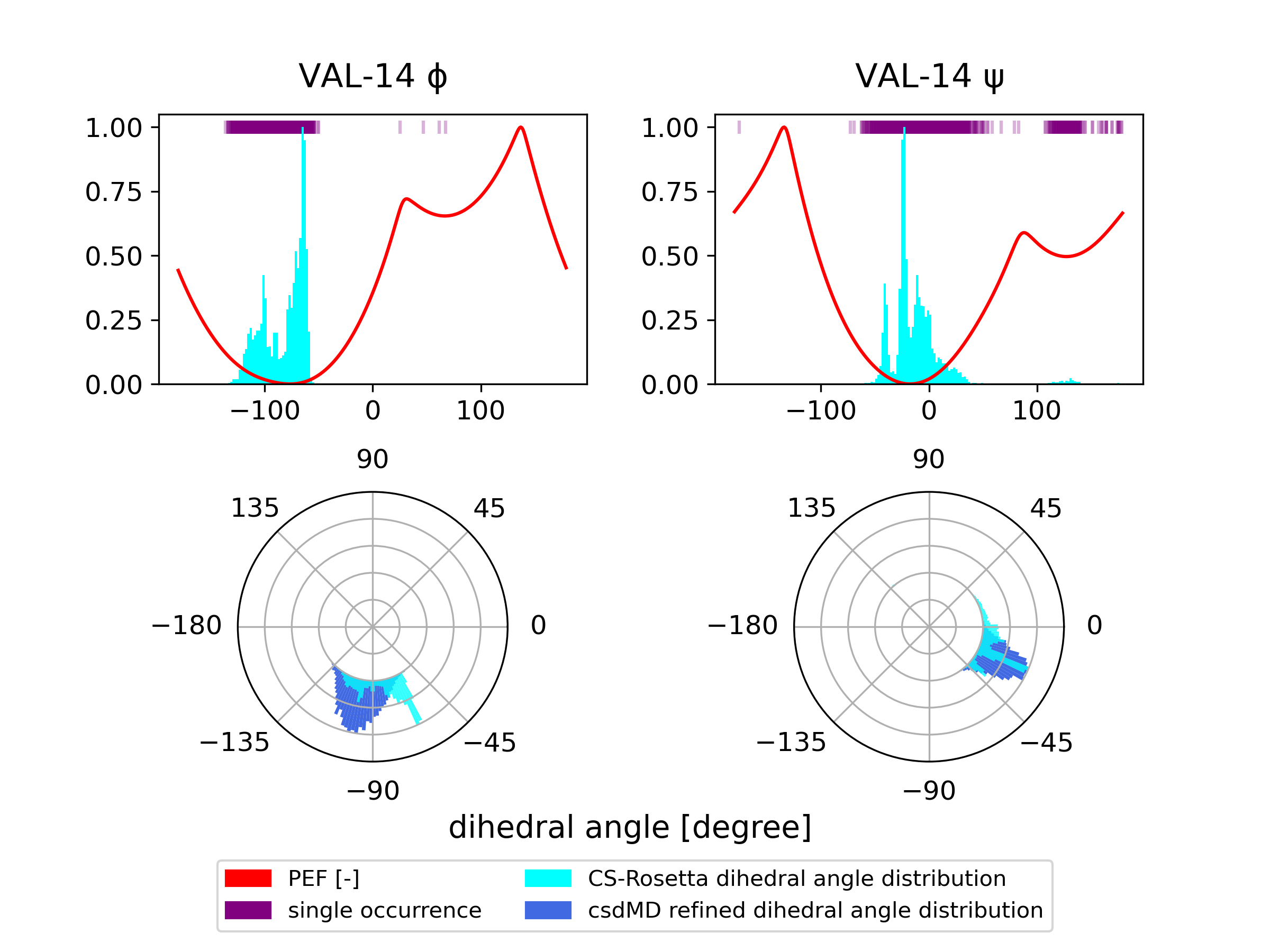

Supplement: Supplementary file 1 [file ijms-24-12101-s001.zip › KRAS-G12C-GDP-Mg-free_angle_figures/14-VAL.png]

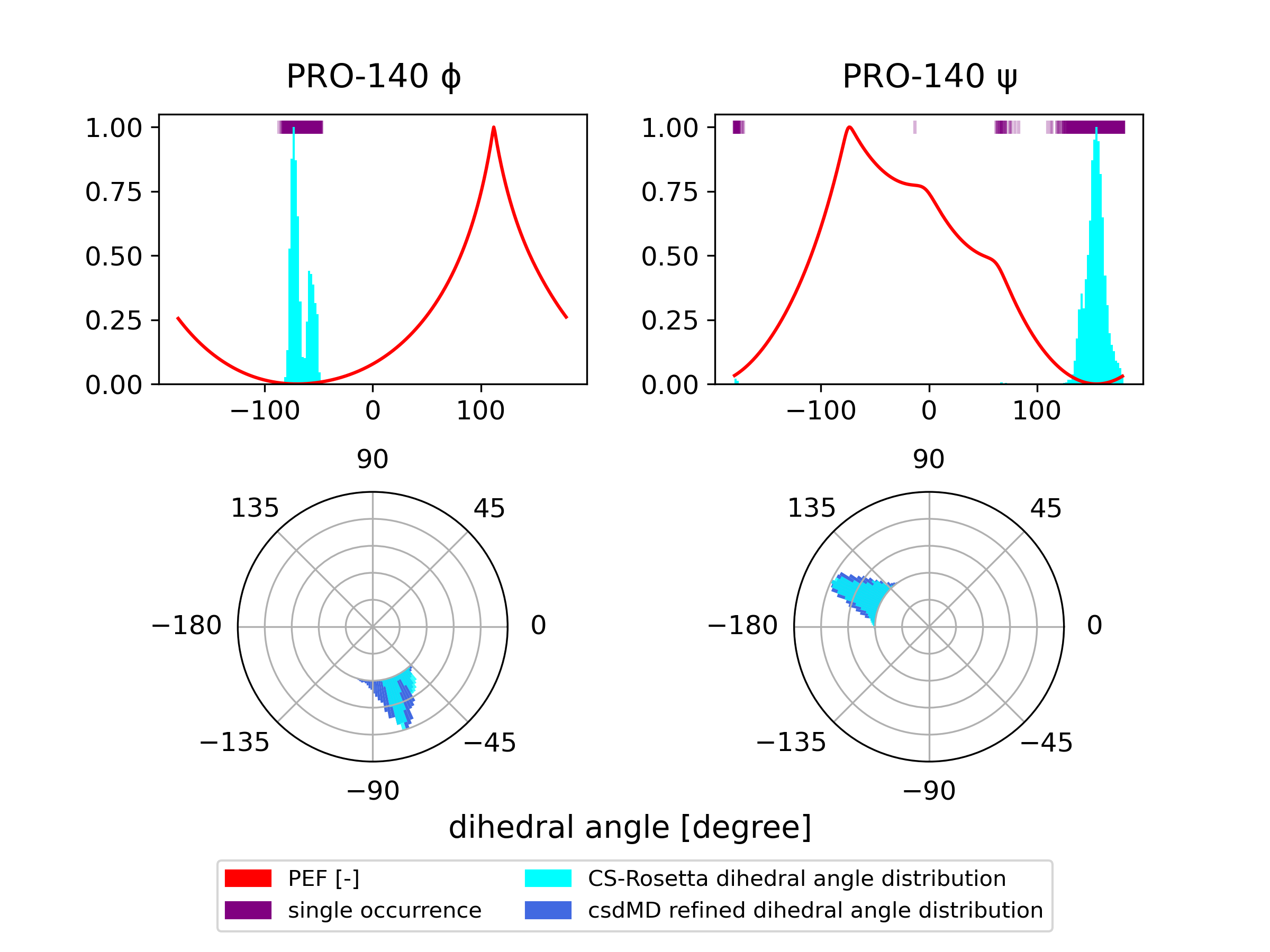

Supplement: Supplementary file 1 [file ijms-24-12101-s001.zip › KRAS-G12C-GDP-Mg-free_angle_figures/140-PRO.png]

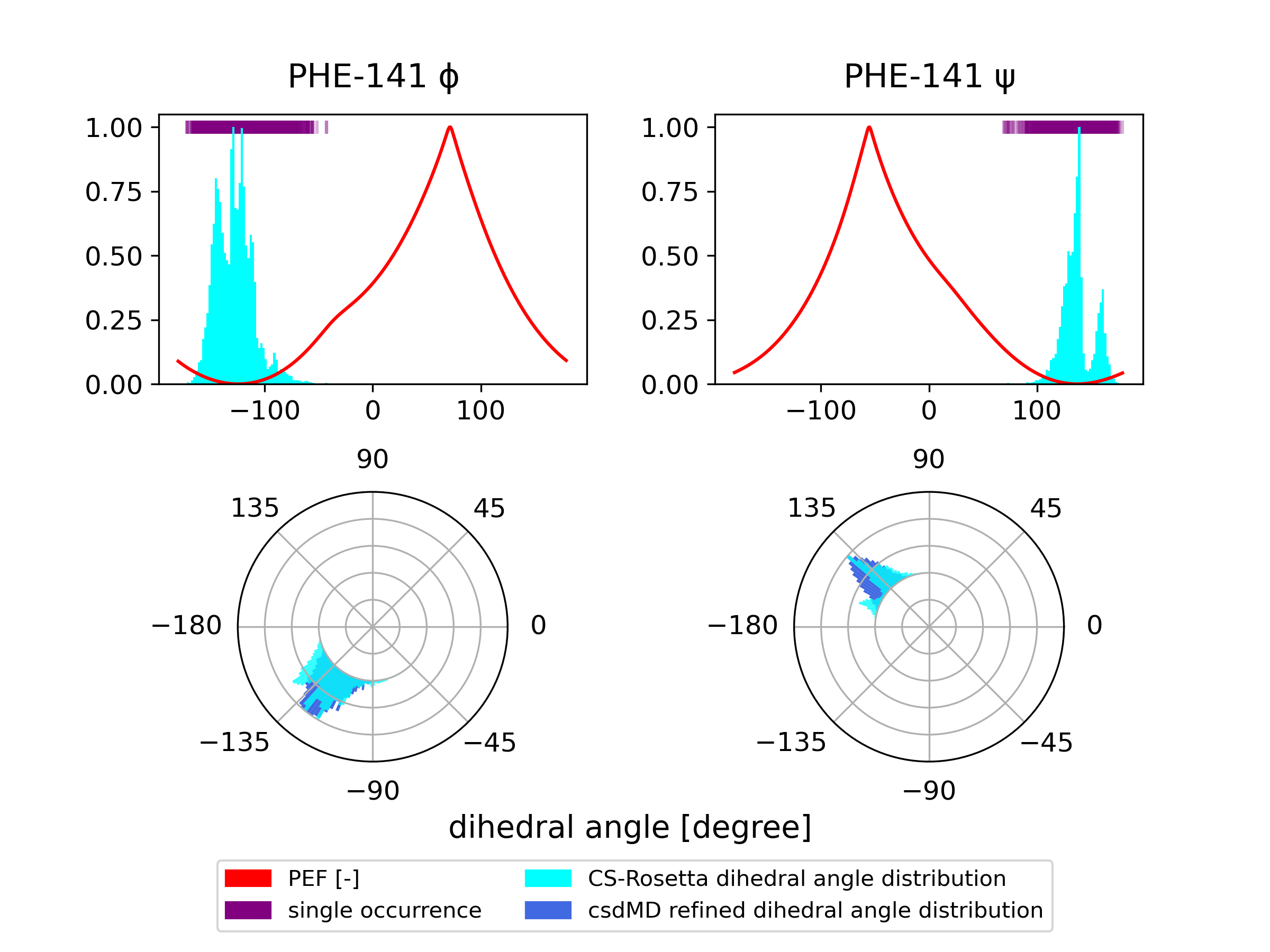

Supplement: Supplementary file 1 [file ijms-24-12101-s001.zip › KRAS-G12C-GDP-Mg-free_angle_figures/141-PHE.png]

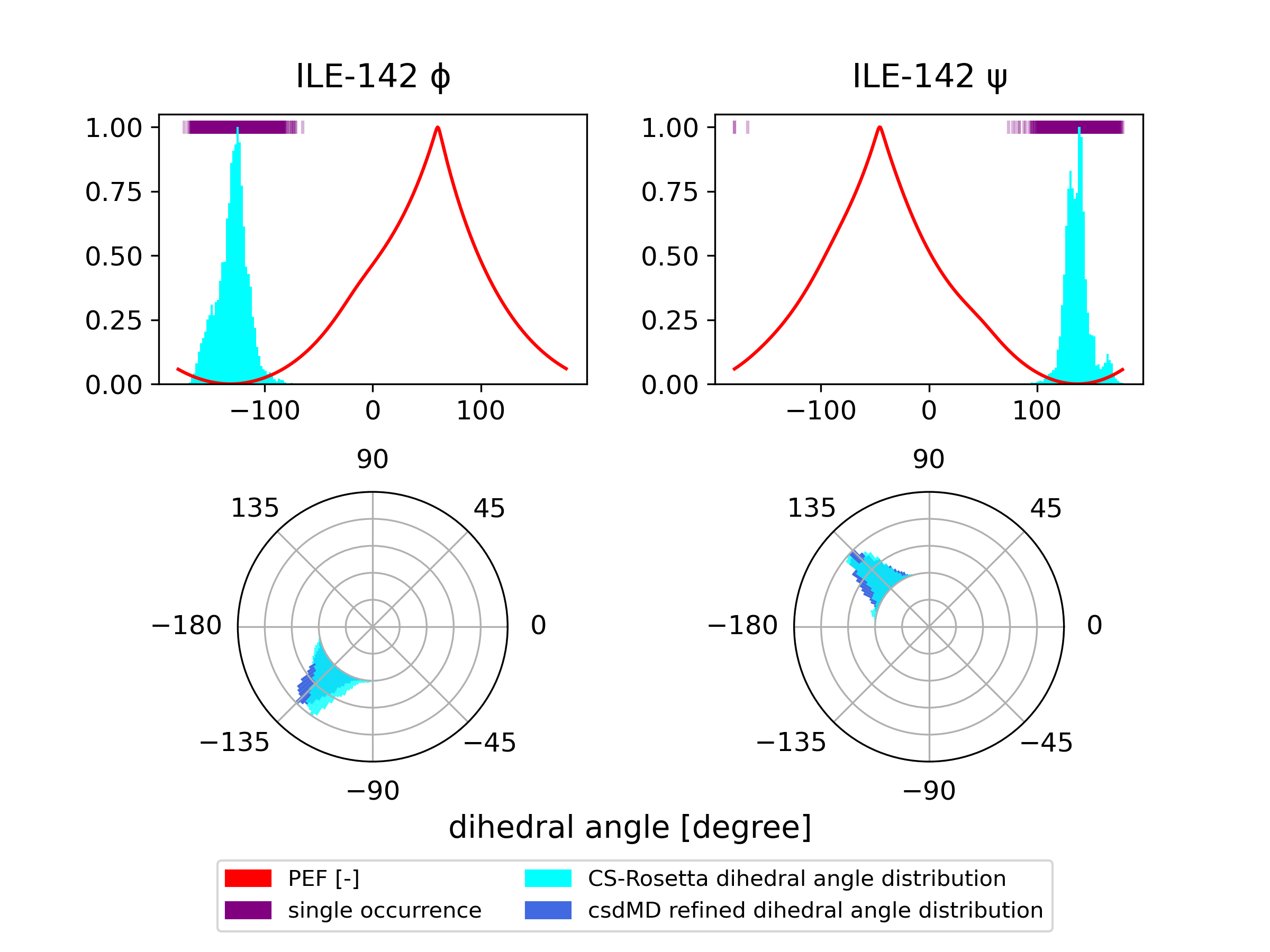

Supplement: Supplementary file 1 [file ijms-24-12101-s001.zip › KRAS-G12C-GDP-Mg-free_angle_figures/142-ILE.png]

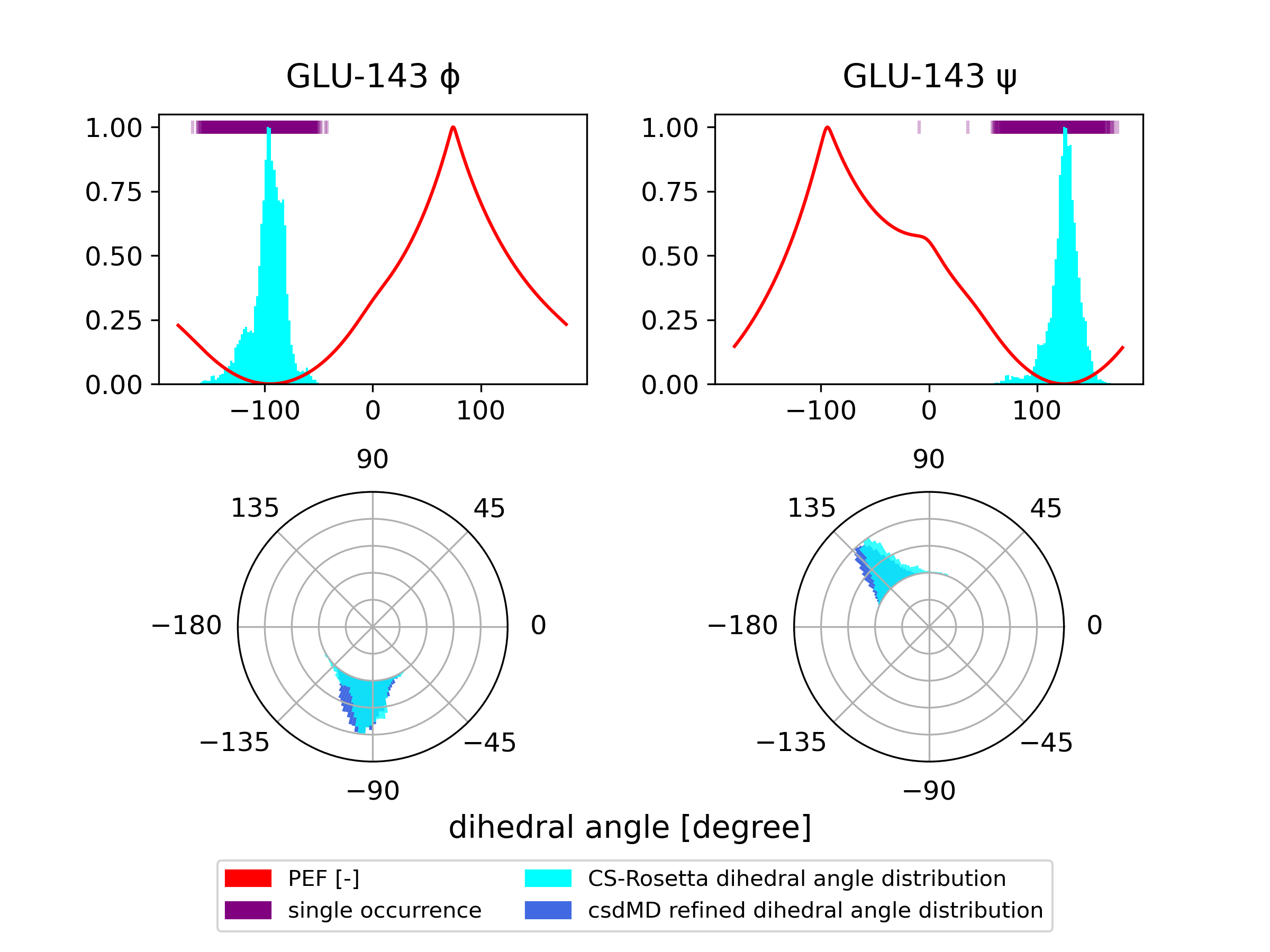

Supplement: Supplementary file 1 [file ijms-24-12101-s001.zip › KRAS-G12C-GDP-Mg-free_angle_figures/143-GLU.png]

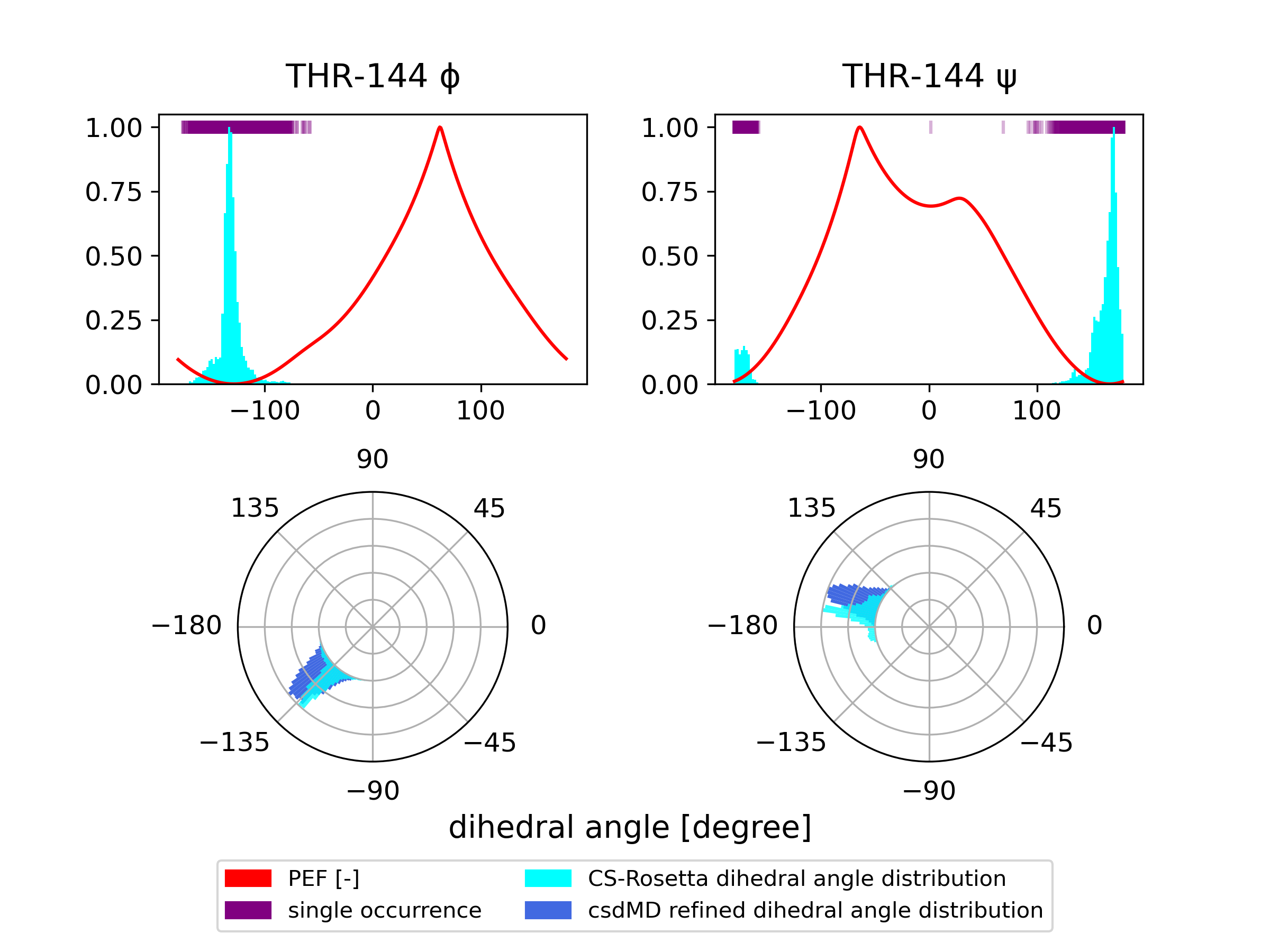

Supplement: Supplementary file 1 [file ijms-24-12101-s001.zip › KRAS-G12C-GDP-Mg-free_angle_figures/144-THR.png]

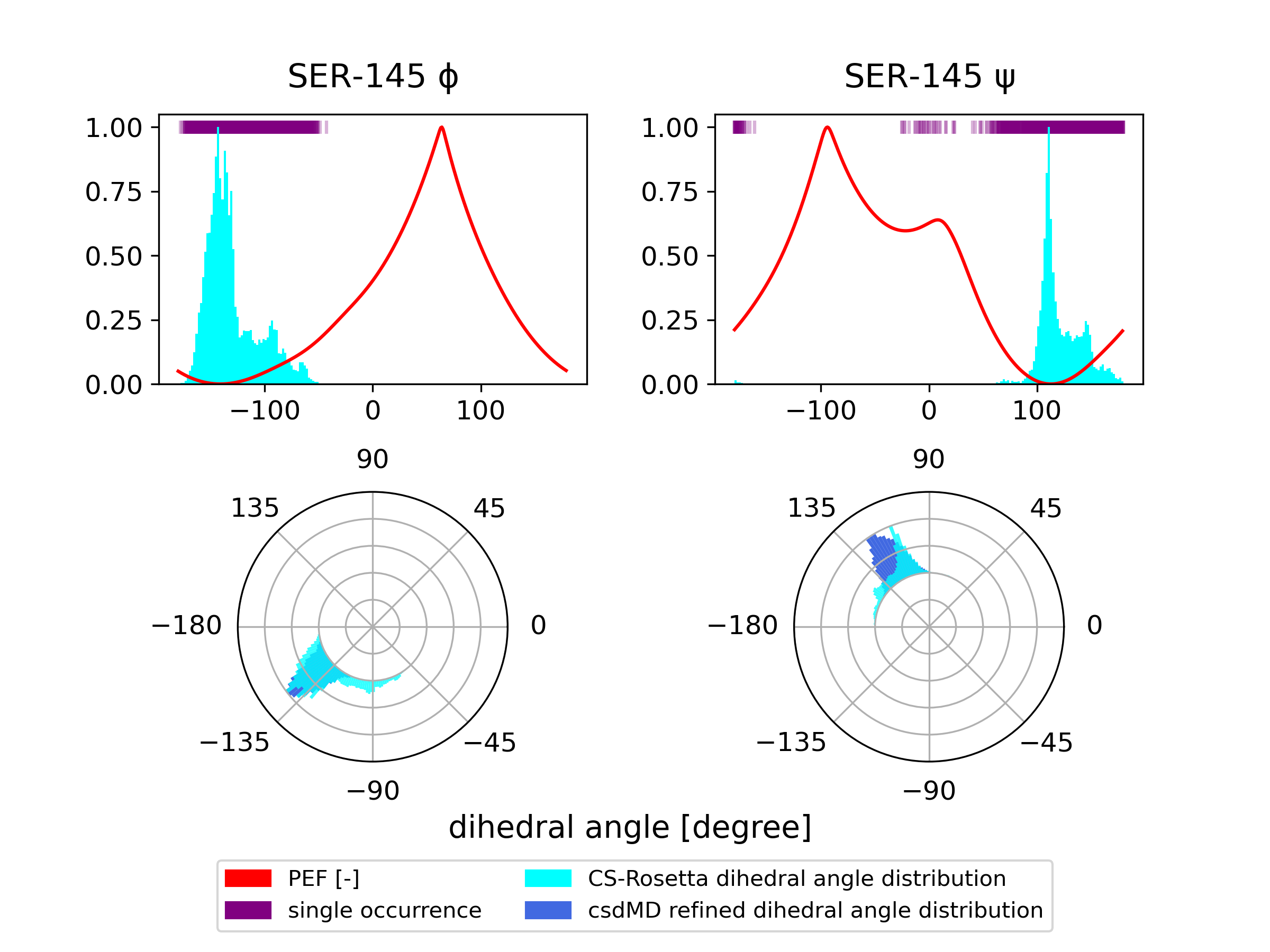

Supplement: Supplementary file 1 [file ijms-24-12101-s001.zip › KRAS-G12C-GDP-Mg-free_angle_figures/145-SER.png]

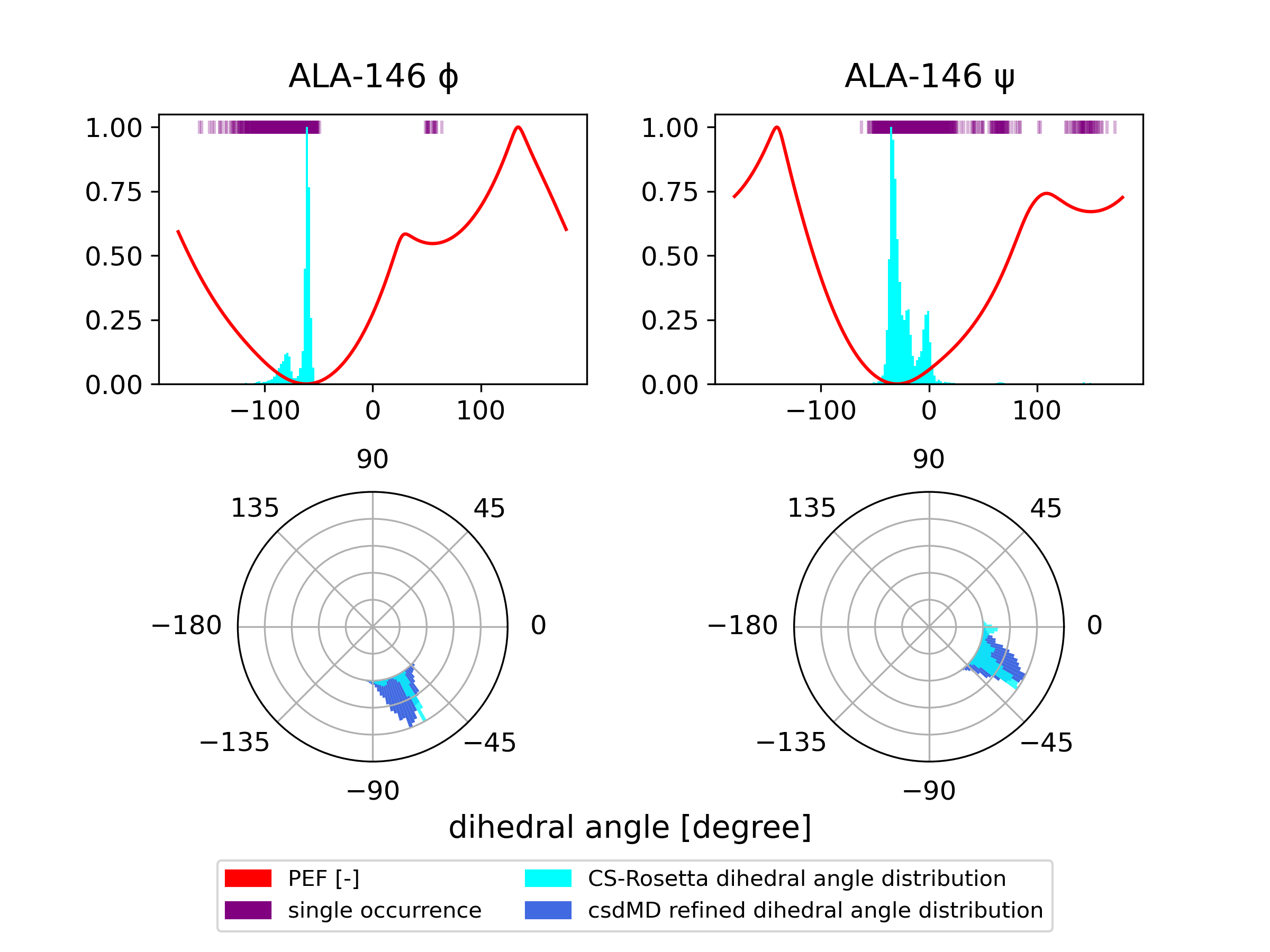

Supplement: Supplementary file 1 [file ijms-24-12101-s001.zip › KRAS-G12C-GDP-Mg-free_angle_figures/146-ALA.png]

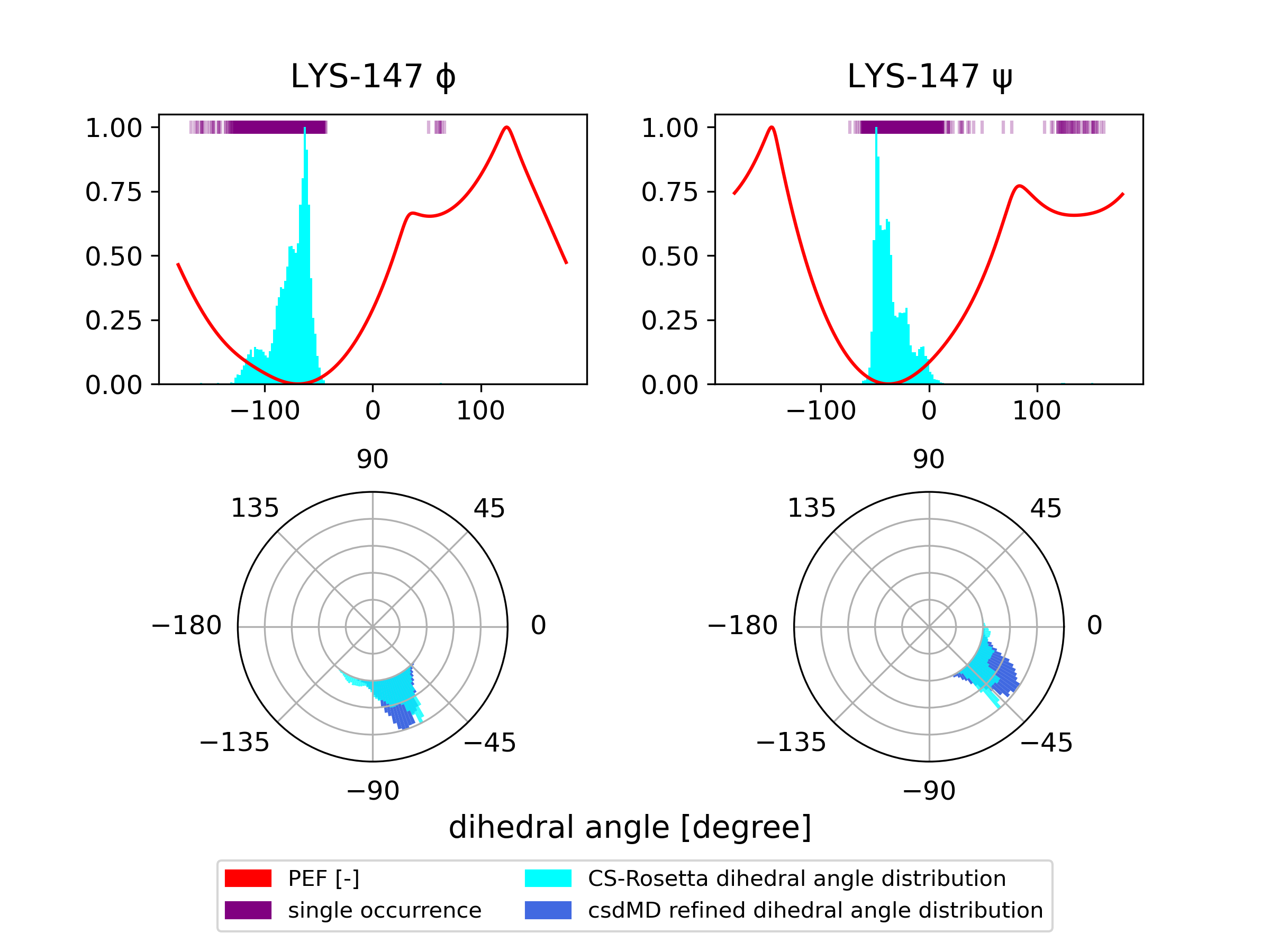

Supplement: Supplementary file 1 [file ijms-24-12101-s001.zip › KRAS-G12C-GDP-Mg-free_angle_figures/147-LYS.png]

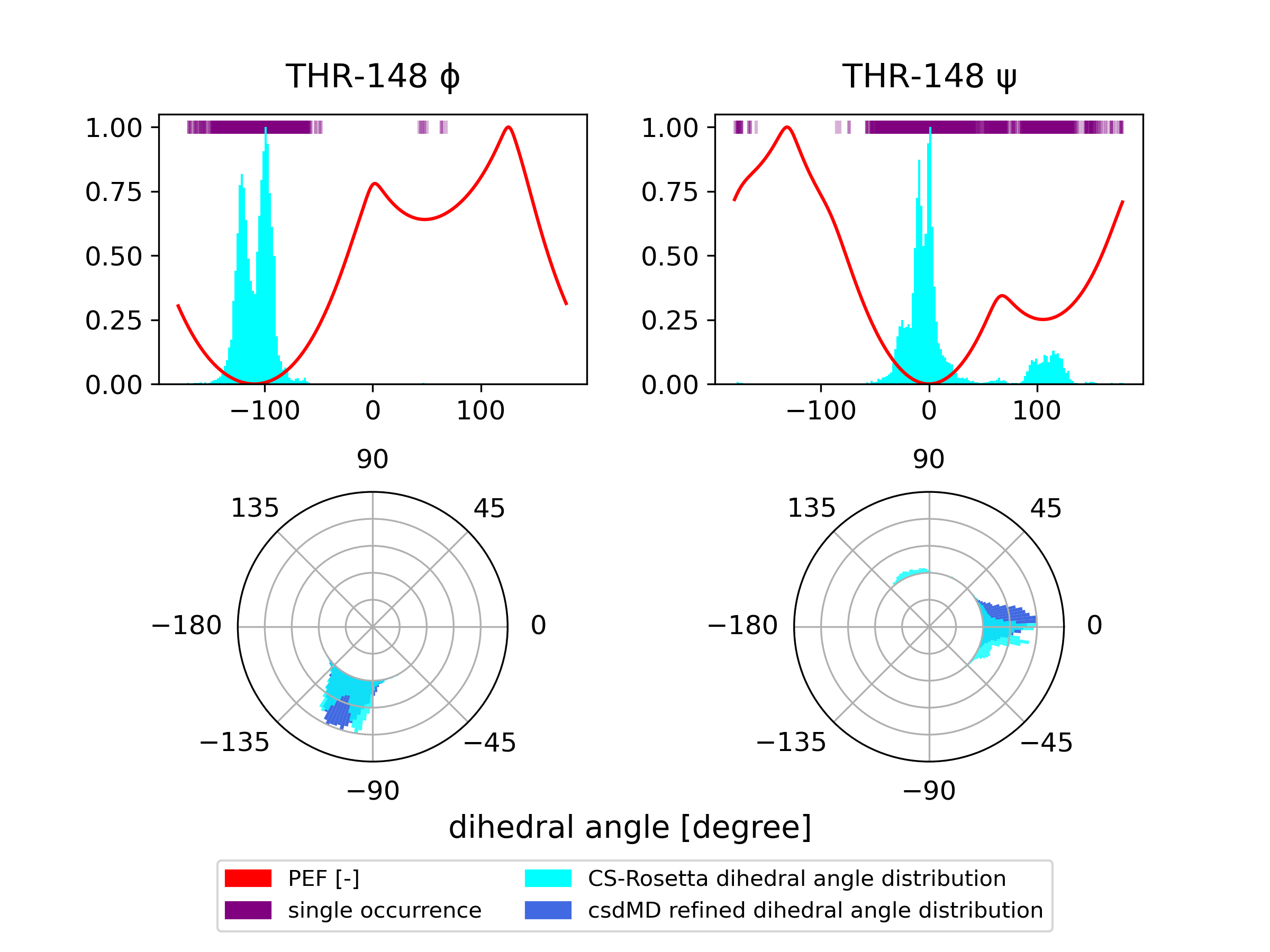

Supplement: Supplementary file 1 [file ijms-24-12101-s001.zip › KRAS-G12C-GDP-Mg-free_angle_figures/148-THR.png]

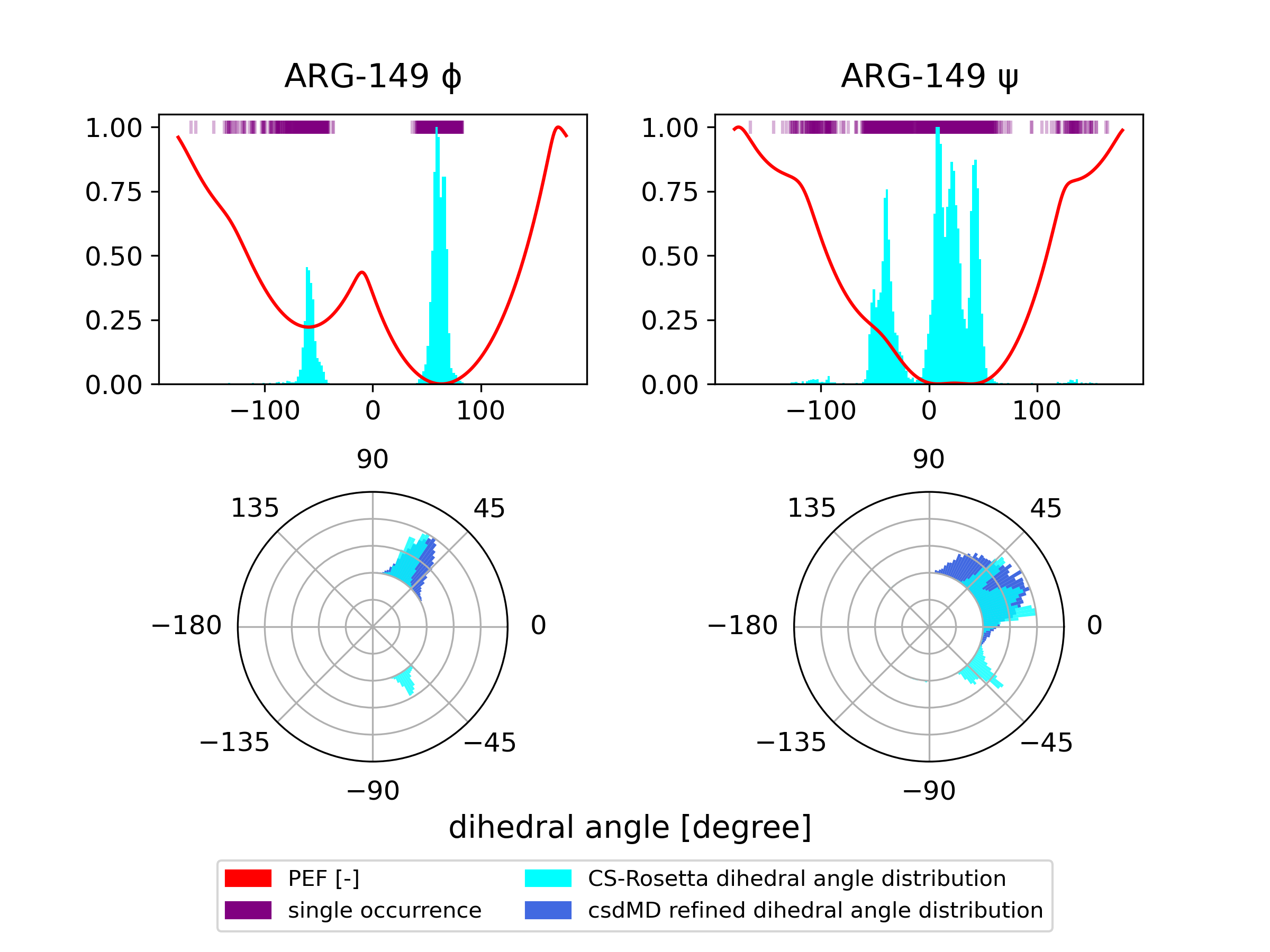

Supplement: Supplementary file 1 [file ijms-24-12101-s001.zip › KRAS-G12C-GDP-Mg-free_angle_figures/149-ARG.png]

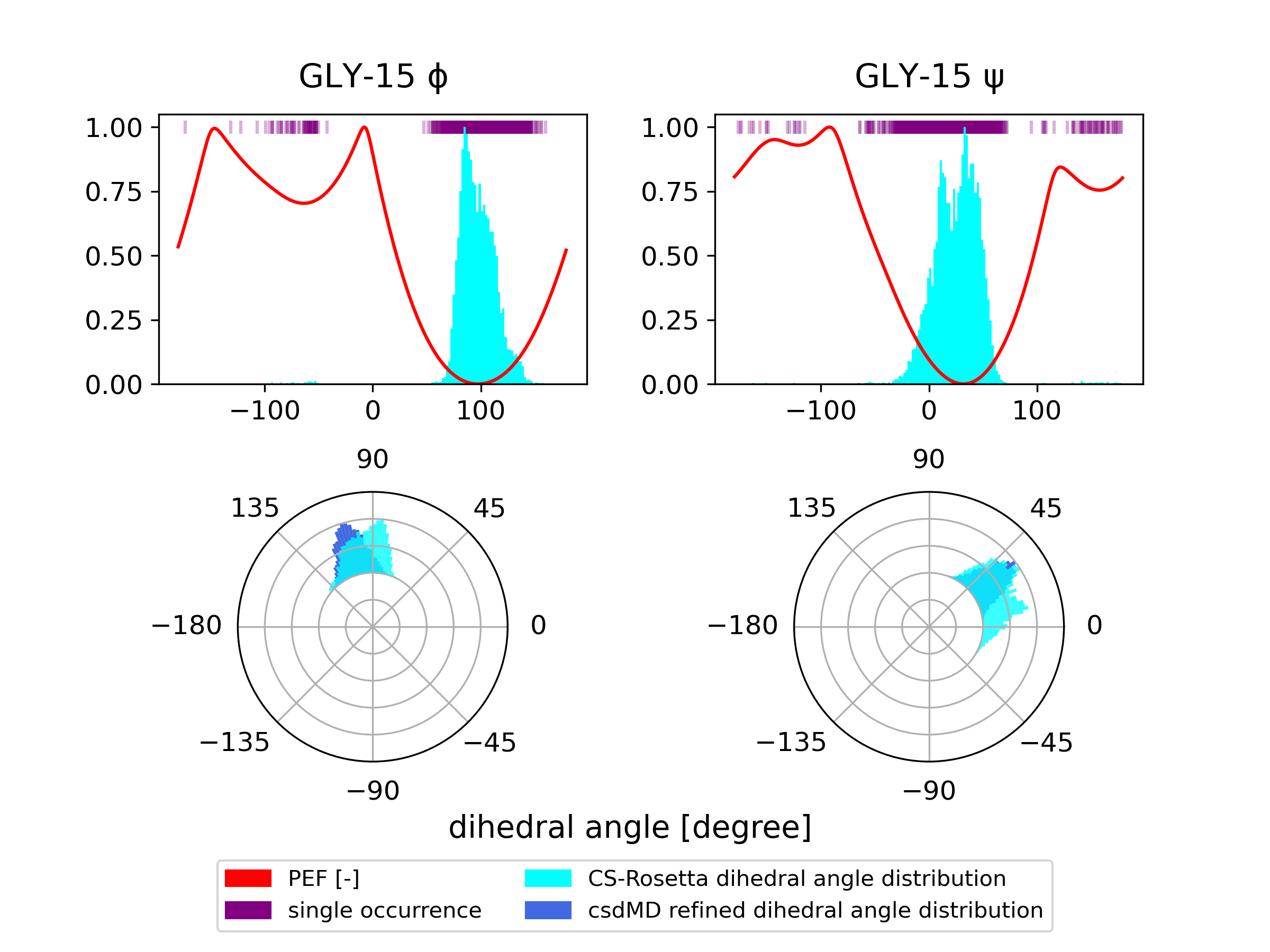

Supplement: Supplementary file 1 [file ijms-24-12101-s001.zip › KRAS-G12C-GDP-Mg-free_angle_figures/15-GLY.png]

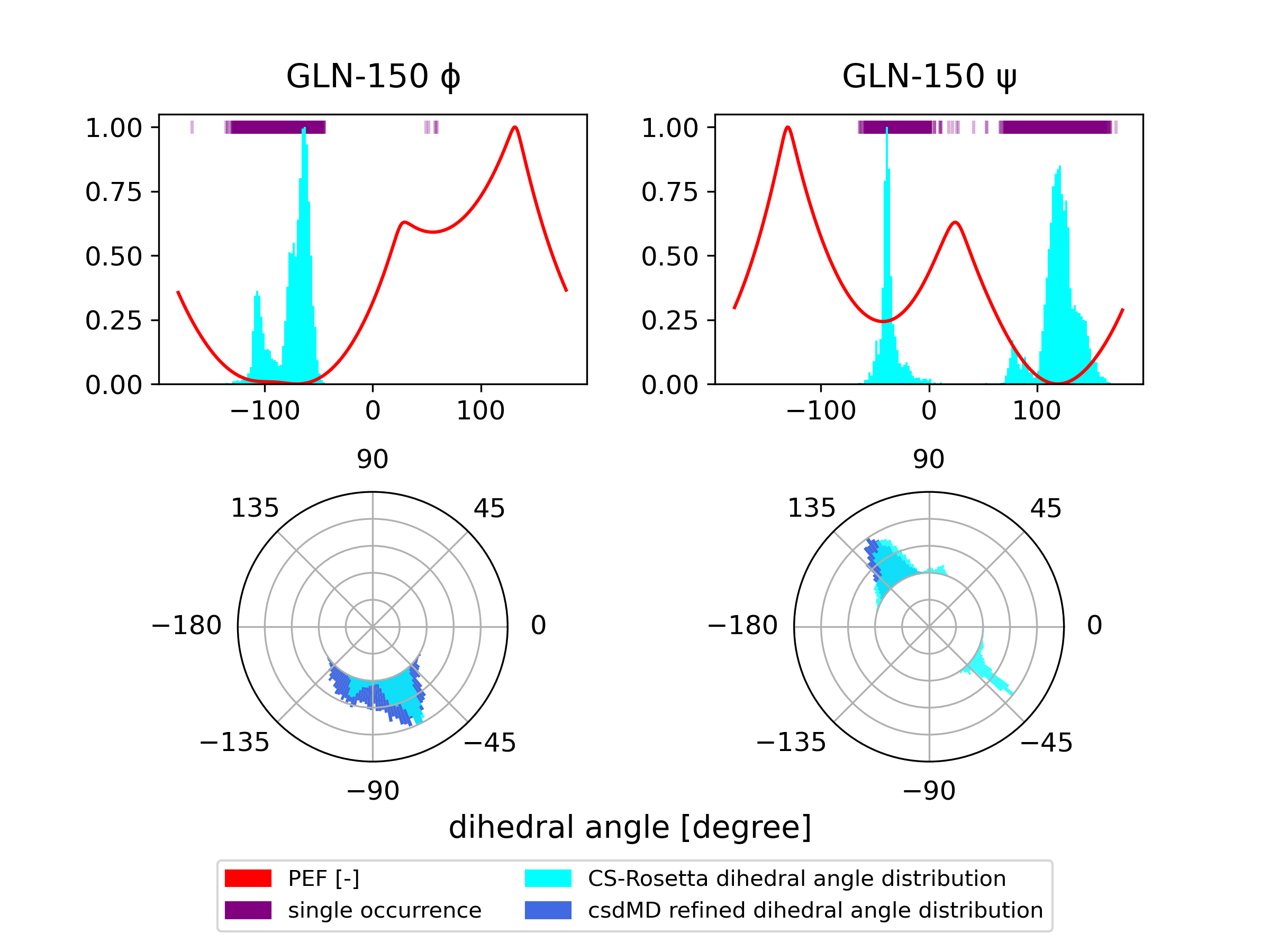

Supplement: Supplementary file 1 [file ijms-24-12101-s001.zip › KRAS-G12C-GDP-Mg-free_angle_figures/150-GLN.png]

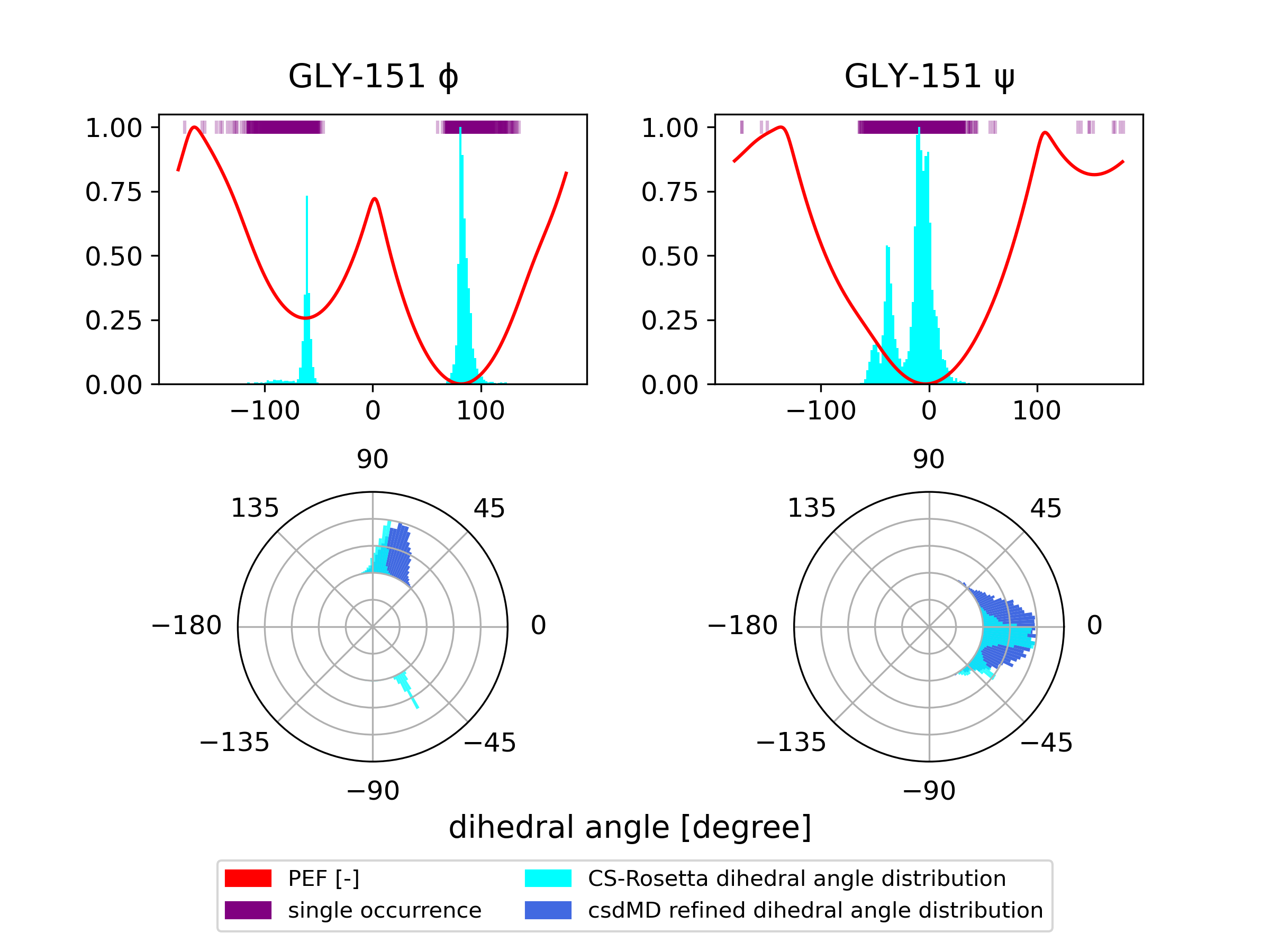

Supplement: Supplementary file 1 [file ijms-24-12101-s001.zip › KRAS-G12C-GDP-Mg-free_angle_figures/151-GLY.png]

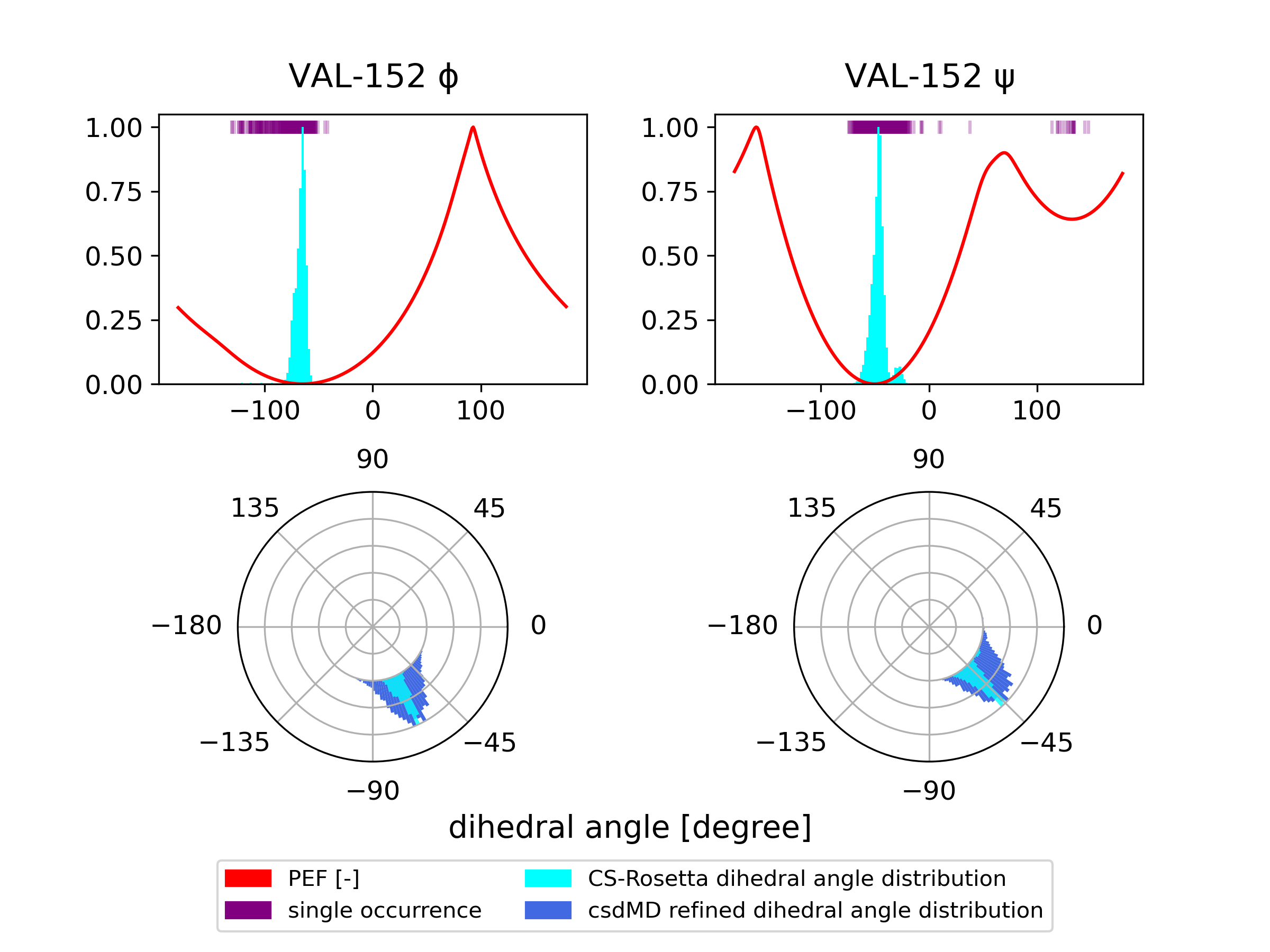

Supplement: Supplementary file 1 [file ijms-24-12101-s001.zip › KRAS-G12C-GDP-Mg-free_angle_figures/152-VAL.png]

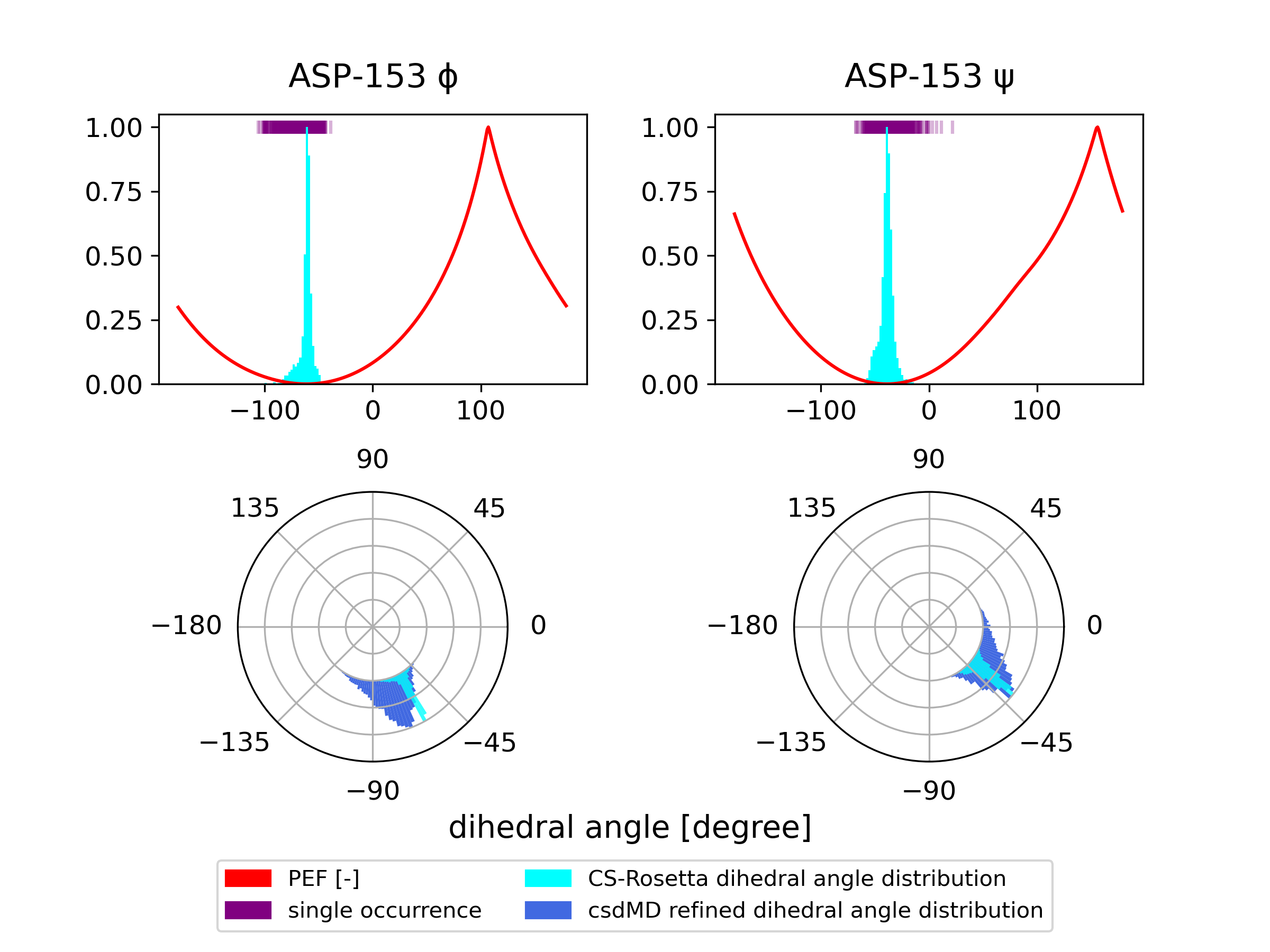

Supplement: Supplementary file 1 [file ijms-24-12101-s001.zip › KRAS-G12C-GDP-Mg-free_angle_figures/153-ASP.png]

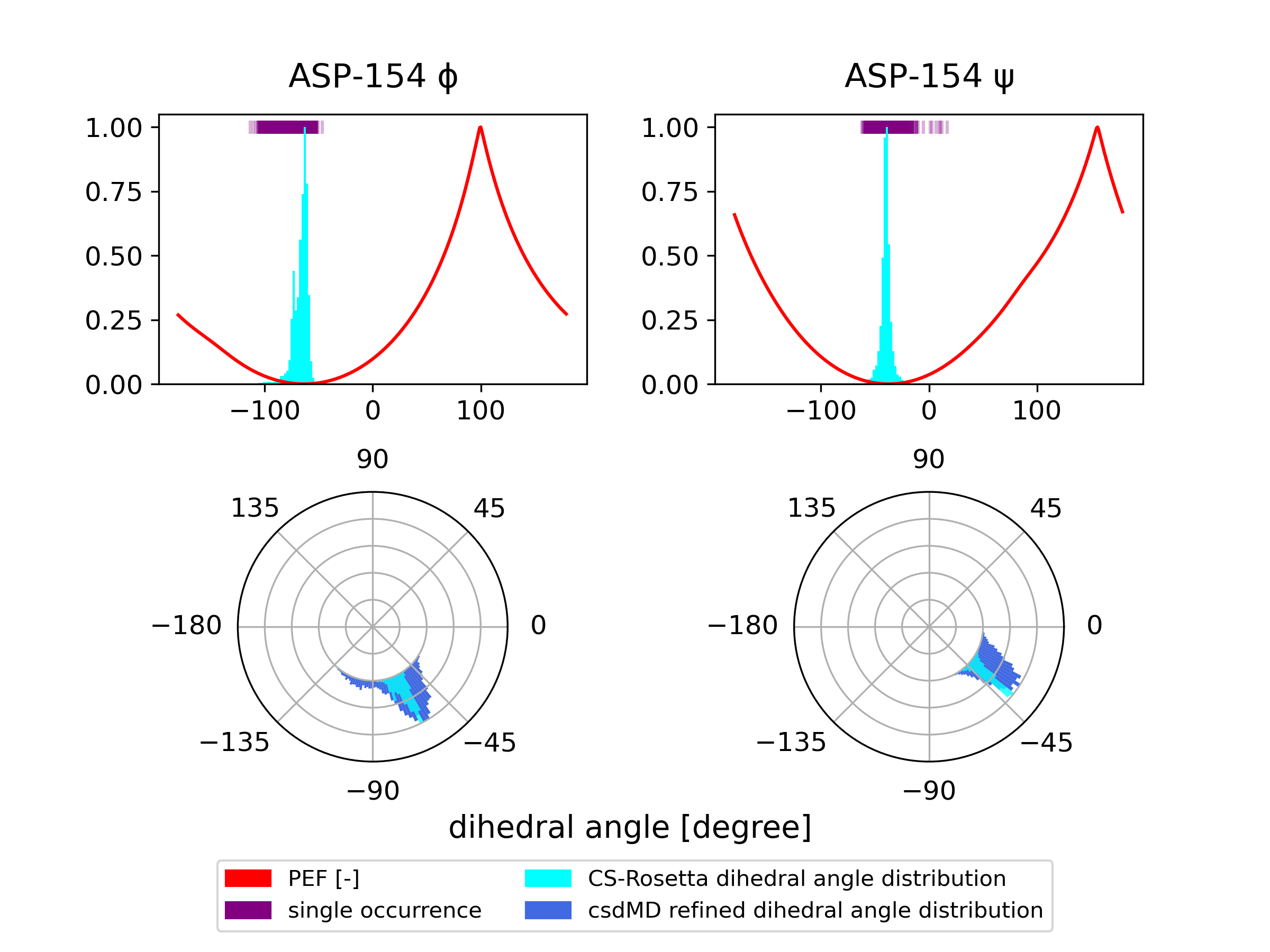

Supplement: Supplementary file 1 [file ijms-24-12101-s001.zip › KRAS-G12C-GDP-Mg-free_angle_figures/154-ASP.png]

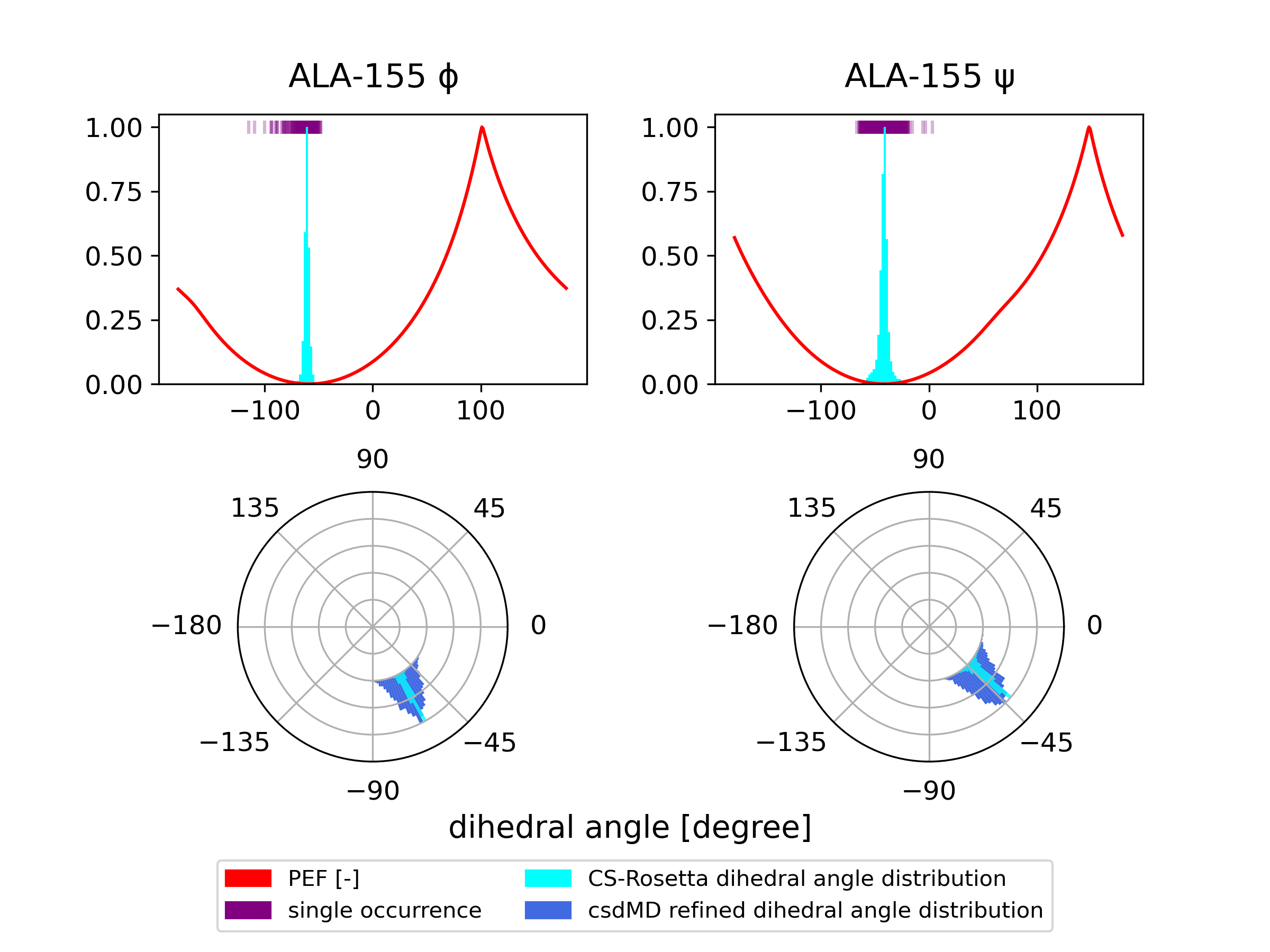

Supplement: Supplementary file 1 [file ijms-24-12101-s001.zip › KRAS-G12C-GDP-Mg-free_angle_figures/155-ALA.png]

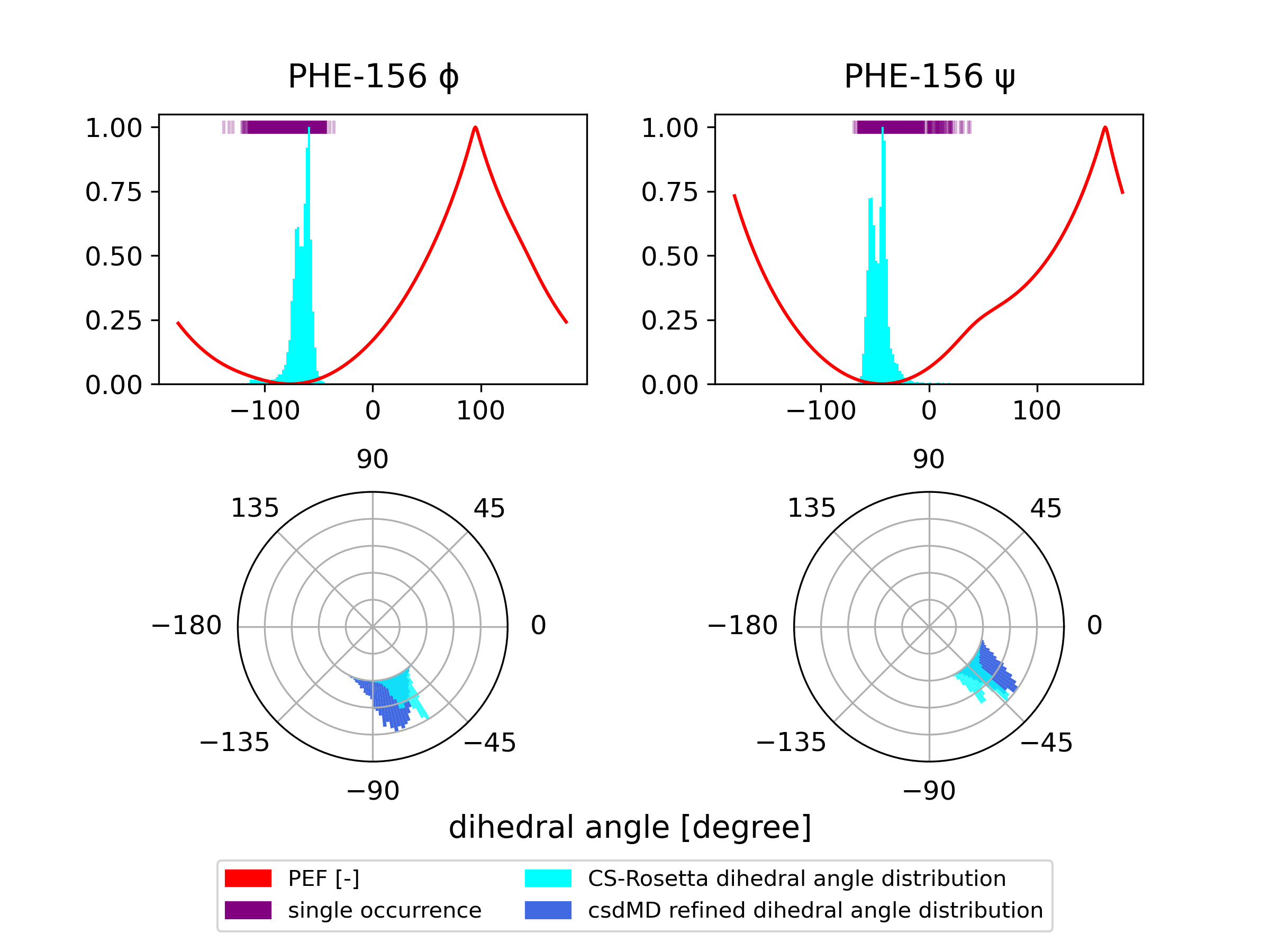

Supplement: Supplementary file 1 [file ijms-24-12101-s001.zip › KRAS-G12C-GDP-Mg-free_angle_figures/156-PHE.png]

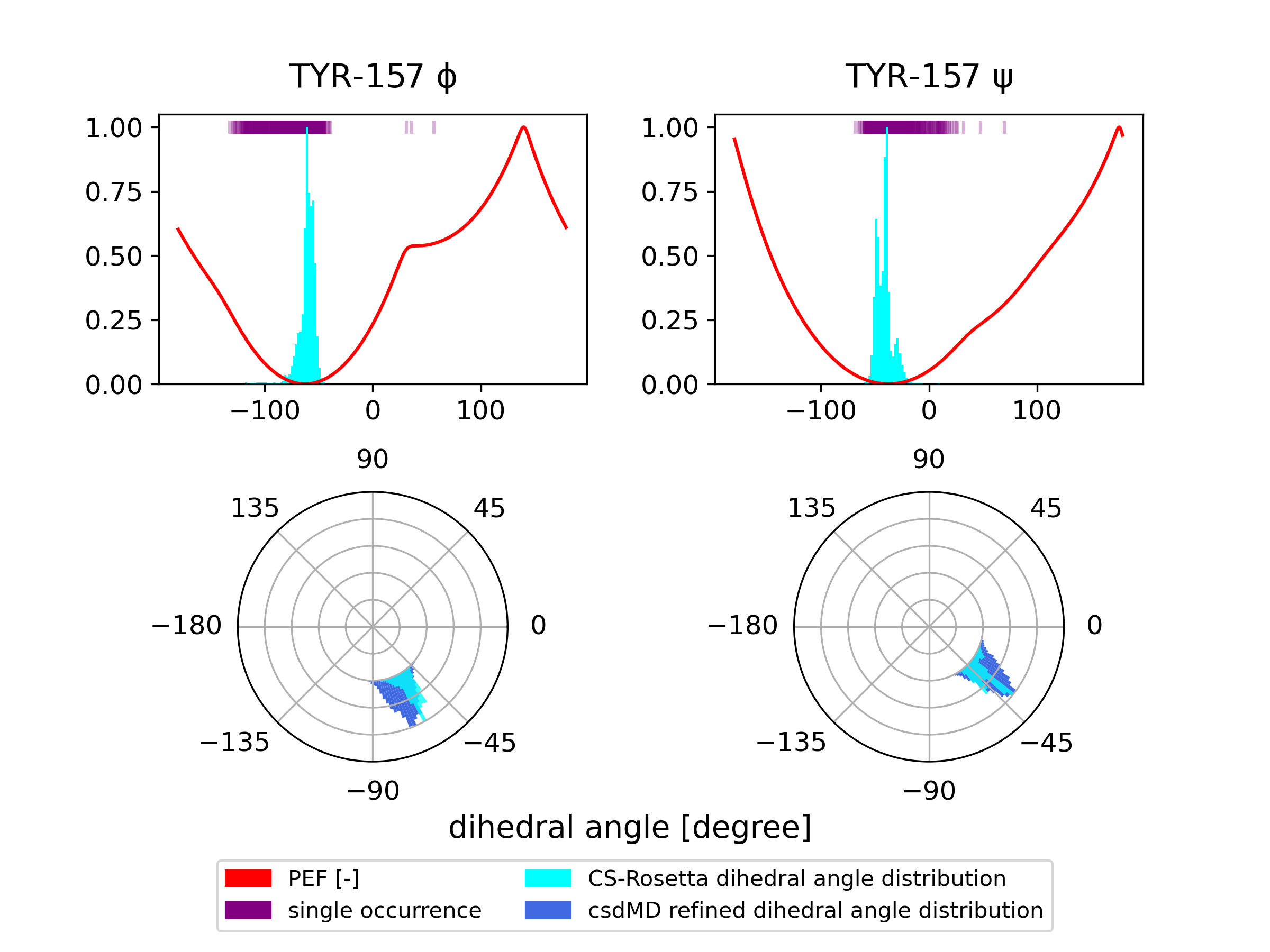

Supplement: Supplementary file 1 [file ijms-24-12101-s001.zip › KRAS-G12C-GDP-Mg-free_angle_figures/157-TYR.png]

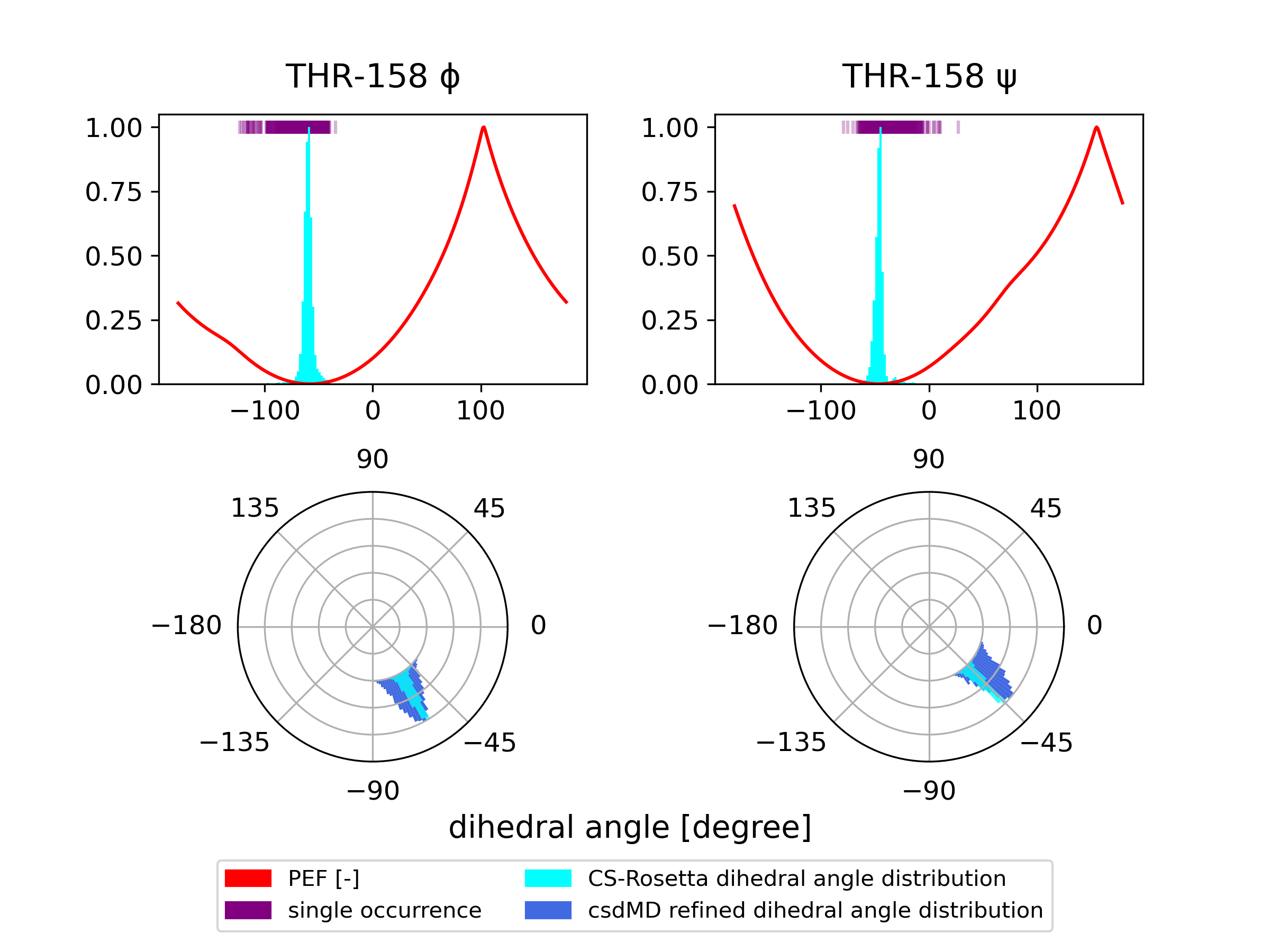

Supplement: Supplementary file 1 [file ijms-24-12101-s001.zip › KRAS-G12C-GDP-Mg-free_angle_figures/158-THR.png]

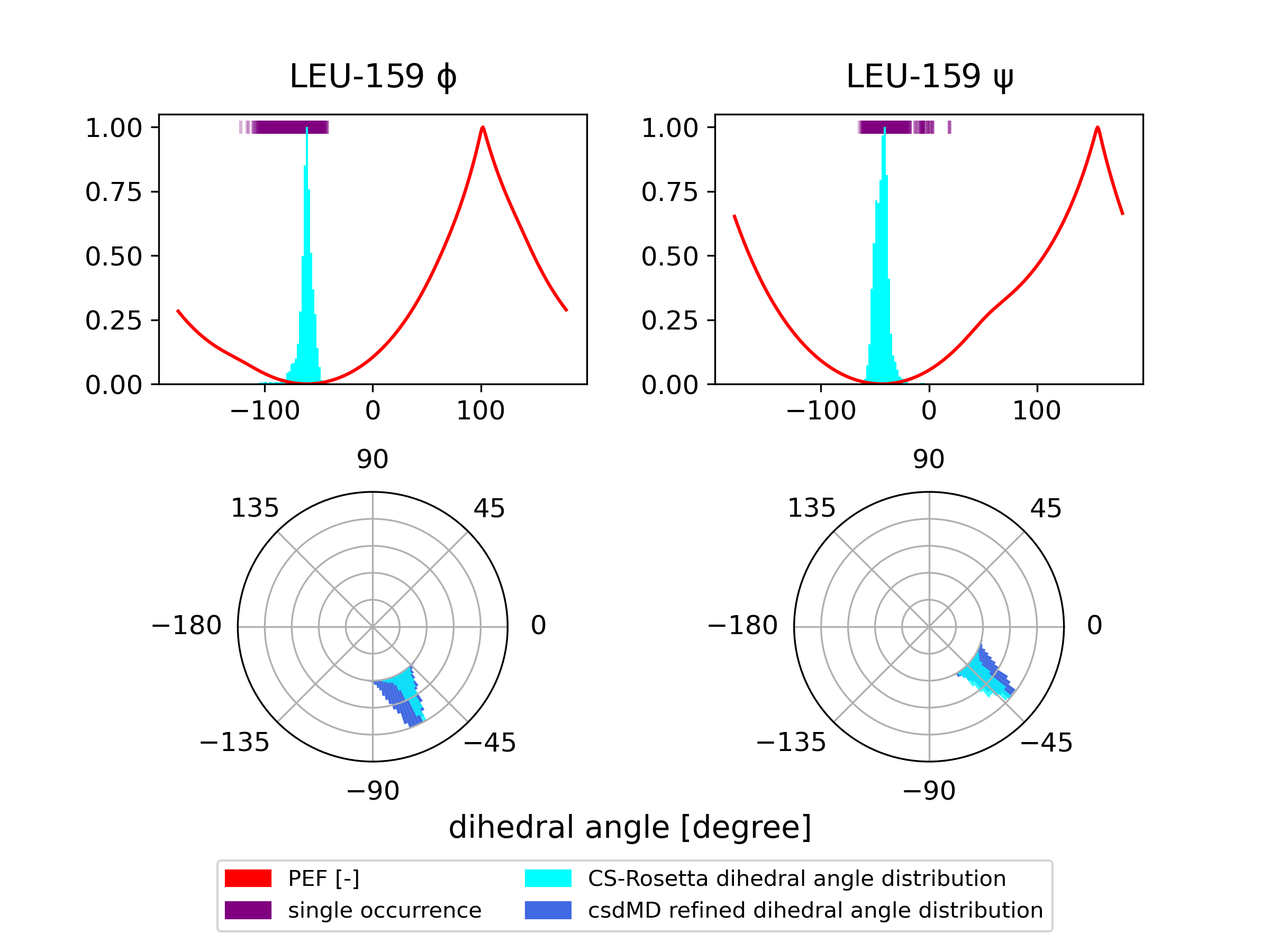

Supplement: Supplementary file 1 [file ijms-24-12101-s001.zip › KRAS-G12C-GDP-Mg-free_angle_figures/159-LEU.png]

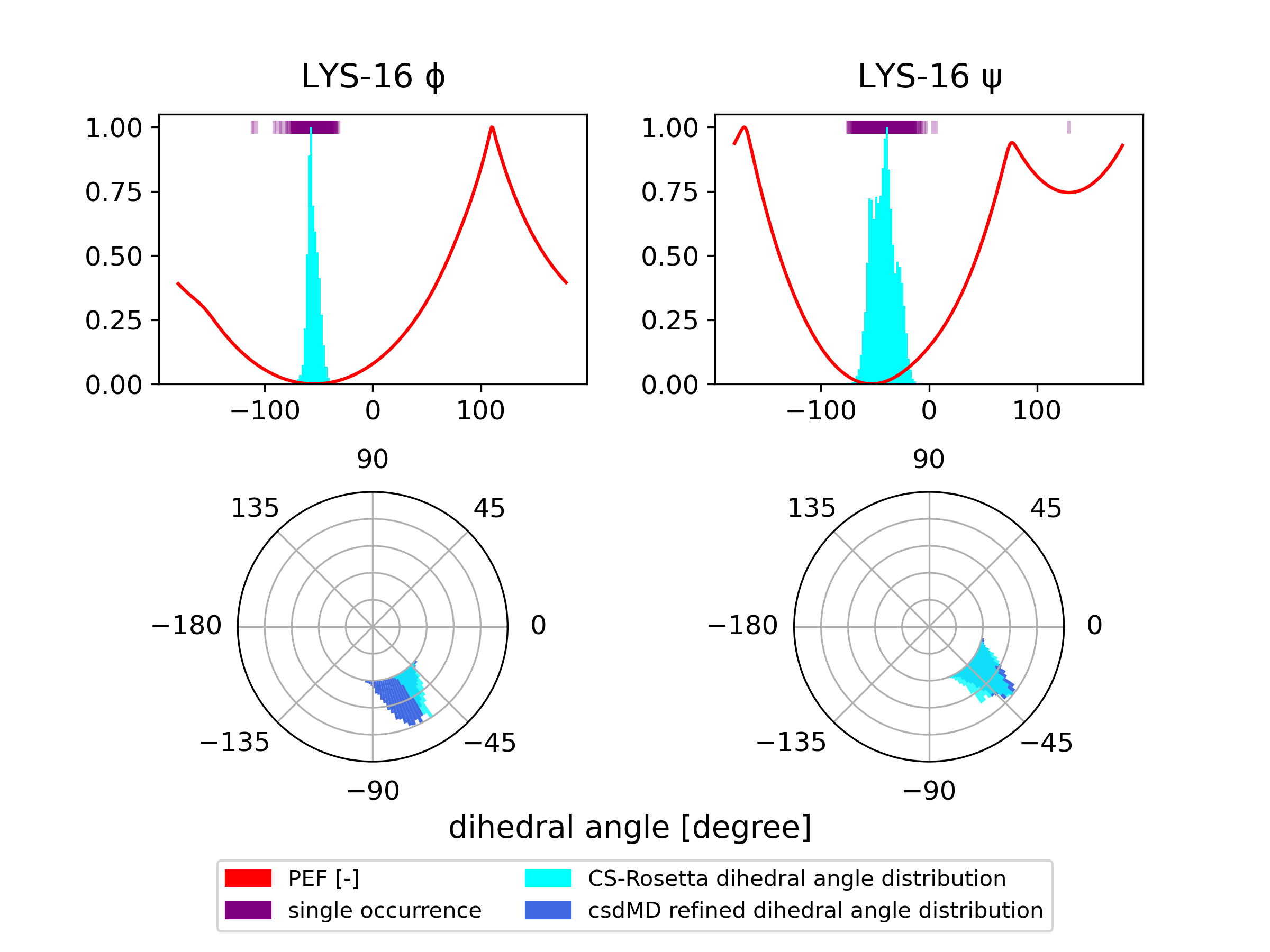

Supplement: Supplementary file 1 [file ijms-24-12101-s001.zip › KRAS-G12C-GDP-Mg-free_angle_figures/16-LYS.png]

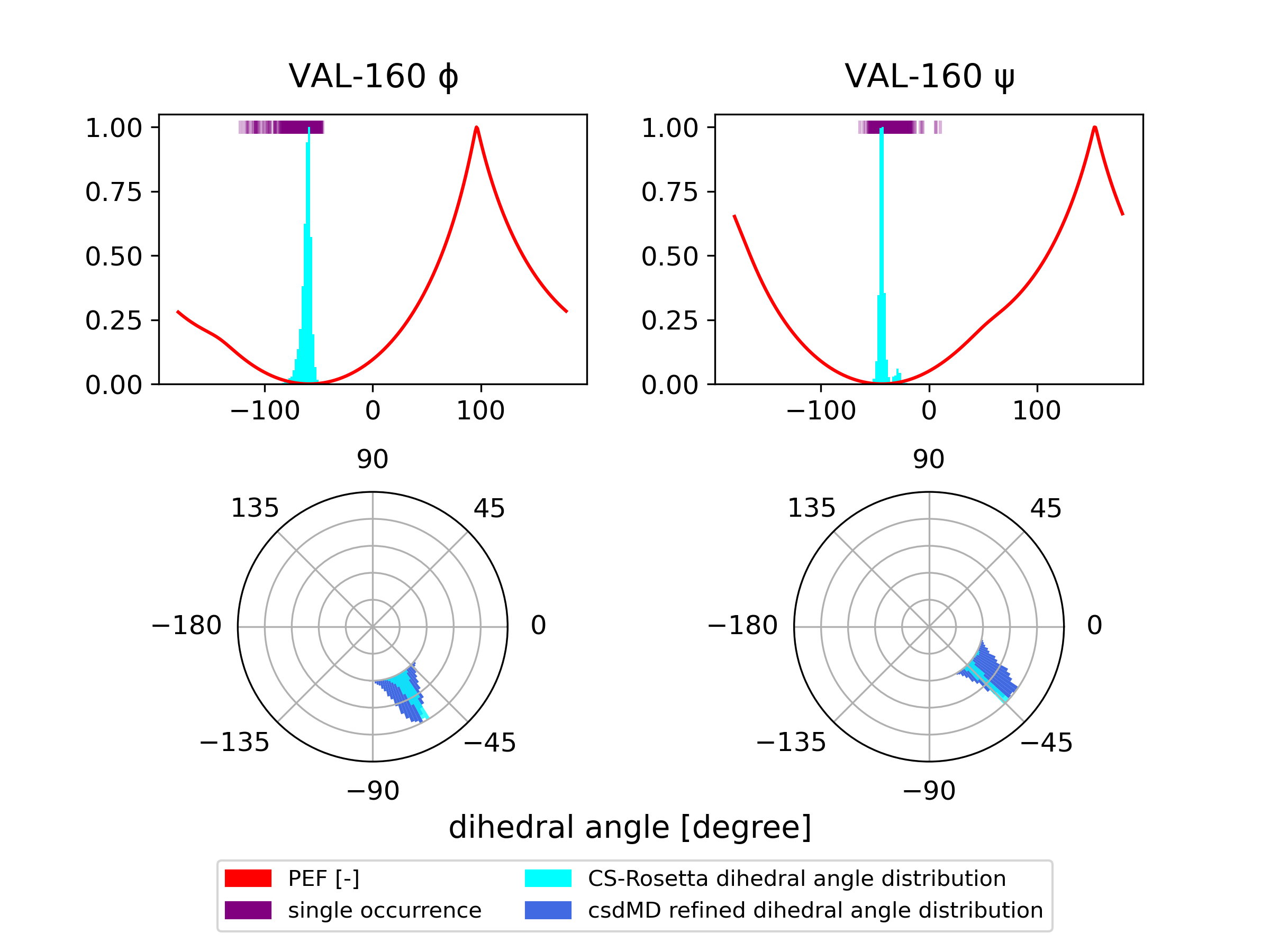

Supplement: Supplementary file 1 [file ijms-24-12101-s001.zip › KRAS-G12C-GDP-Mg-free_angle_figures/160-VAL.png]

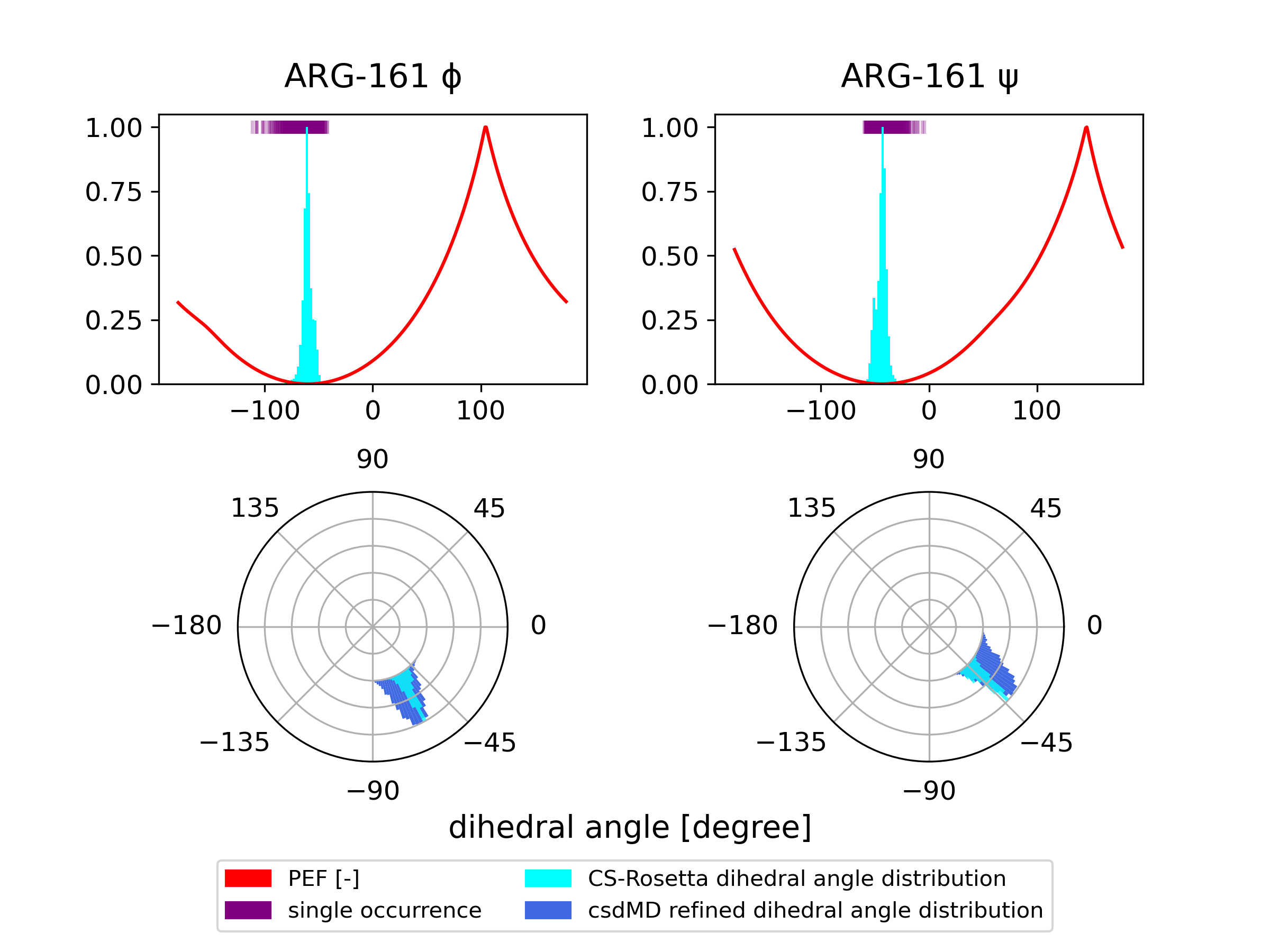

Supplement: Supplementary file 1 [file ijms-24-12101-s001.zip › KRAS-G12C-GDP-Mg-free_angle_figures/161-ARG.png]

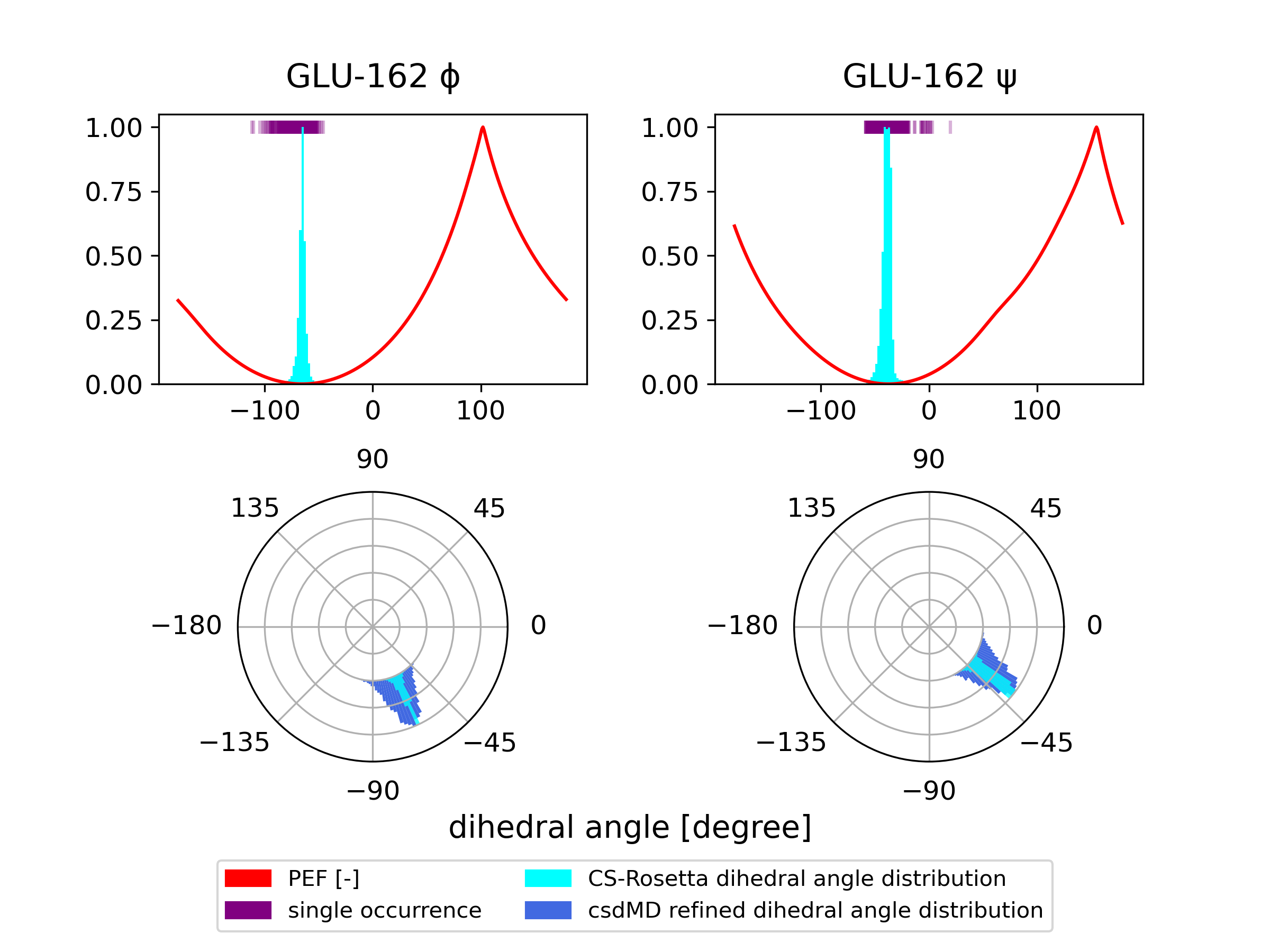

Supplement: Supplementary file 1 [file ijms-24-12101-s001.zip › KRAS-G12C-GDP-Mg-free_angle_figures/162-GLU.png]

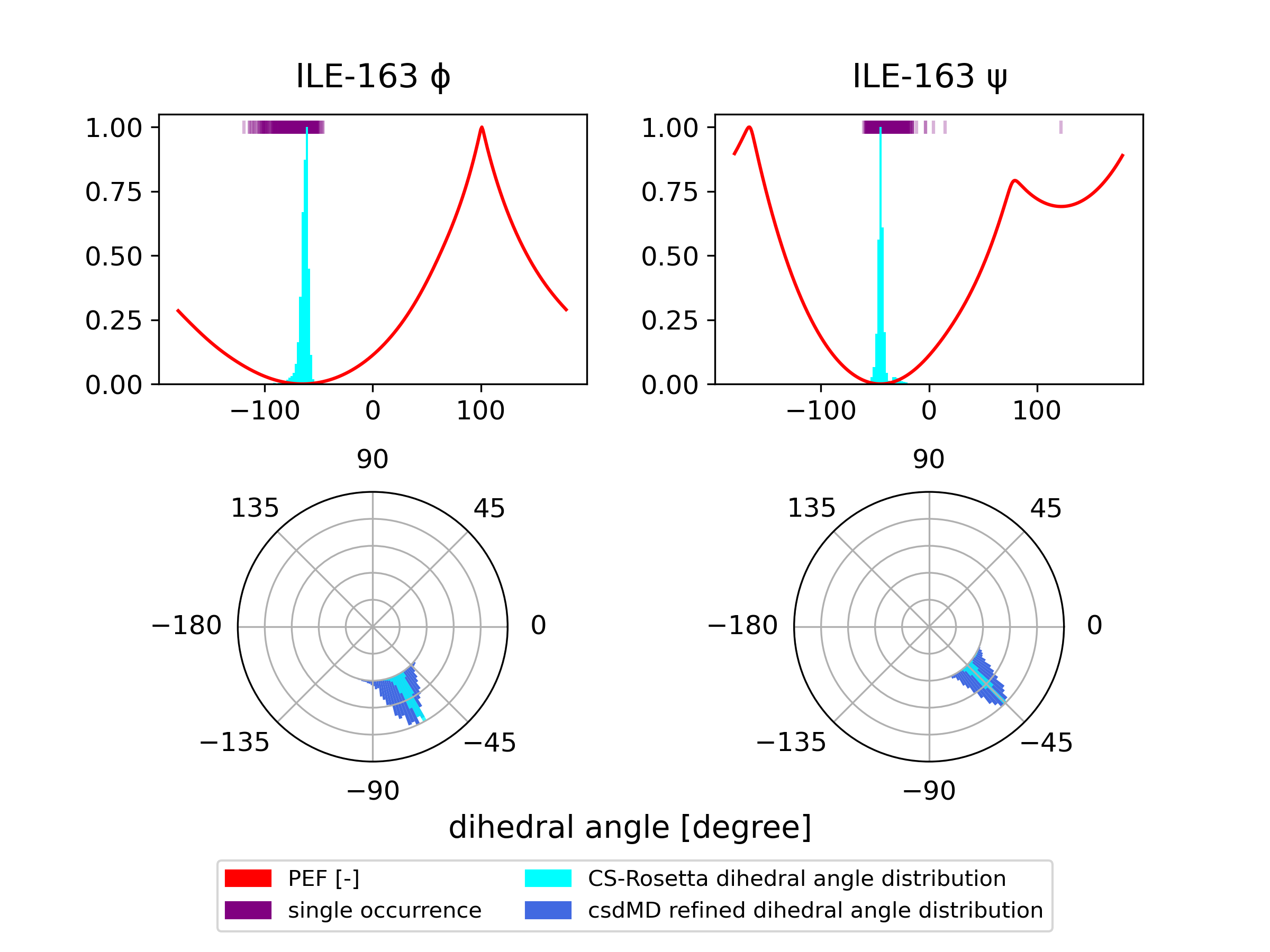

Supplement: Supplementary file 1 [file ijms-24-12101-s001.zip › KRAS-G12C-GDP-Mg-free_angle_figures/163-ILE.png]

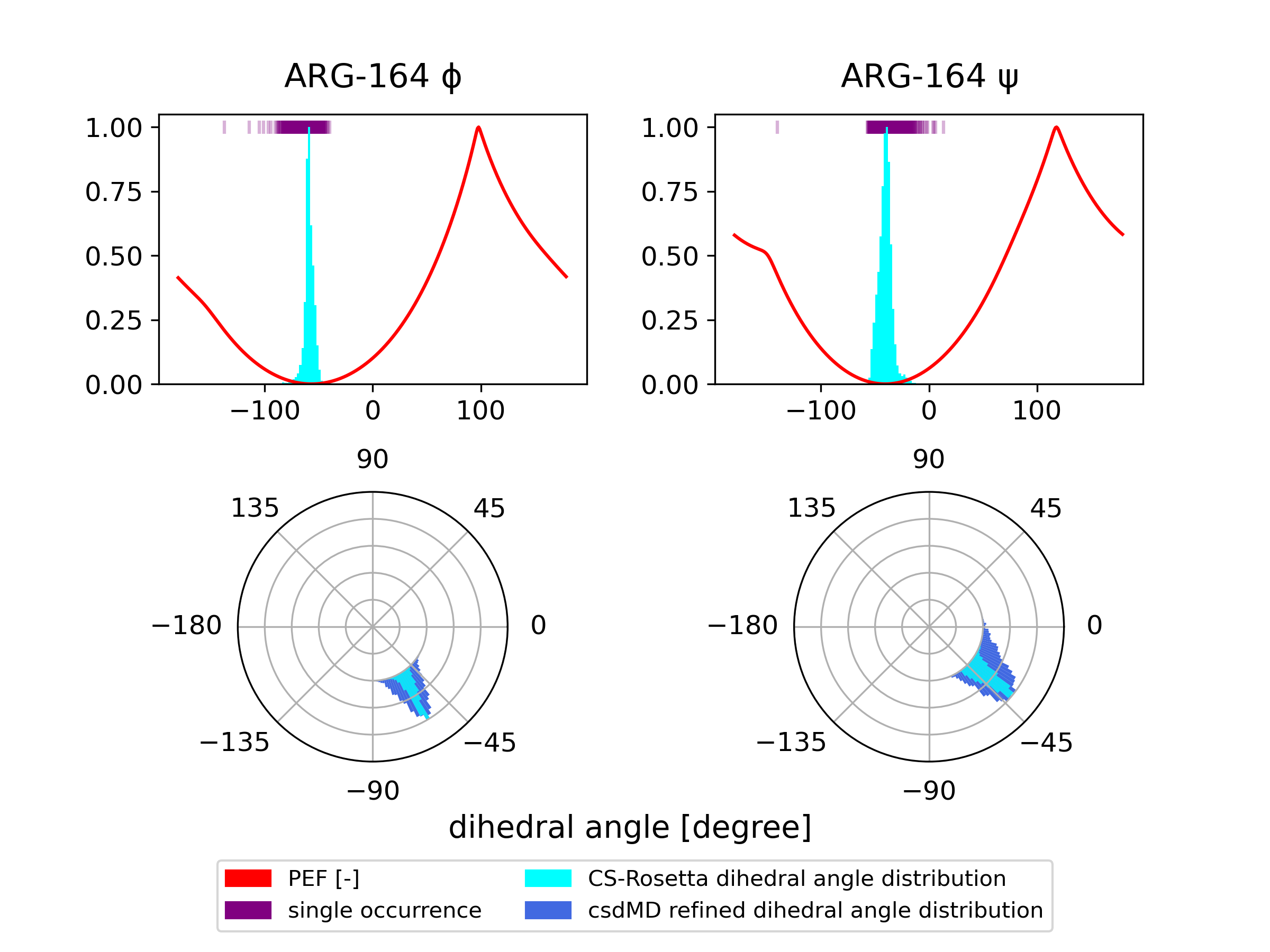

Supplement: Supplementary file 1 [file ijms-24-12101-s001.zip › KRAS-G12C-GDP-Mg-free_angle_figures/164-ARG.png]

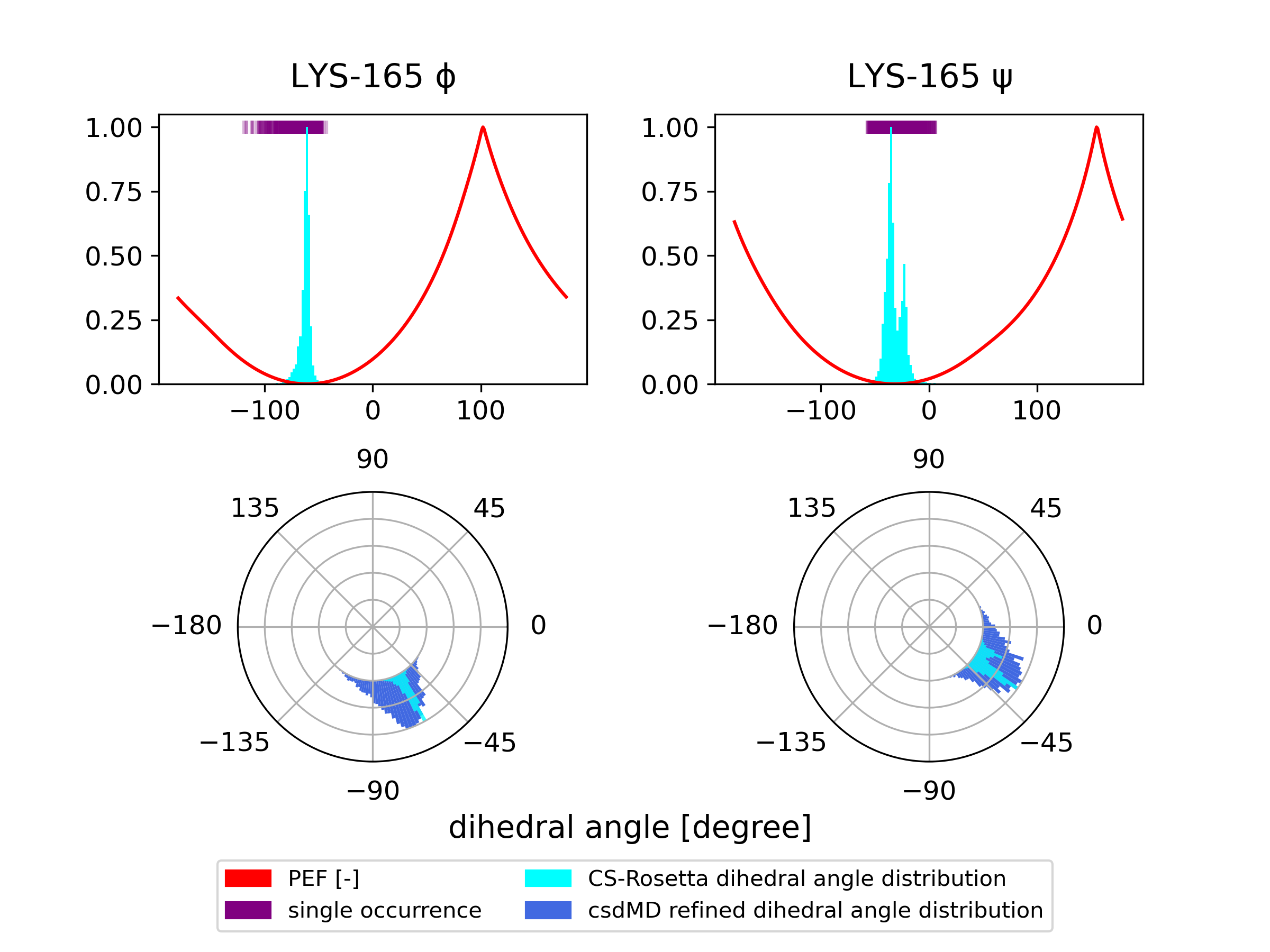

Supplement: Supplementary file 1 [file ijms-24-12101-s001.zip › KRAS-G12C-GDP-Mg-free_angle_figures/165-LYS.png]

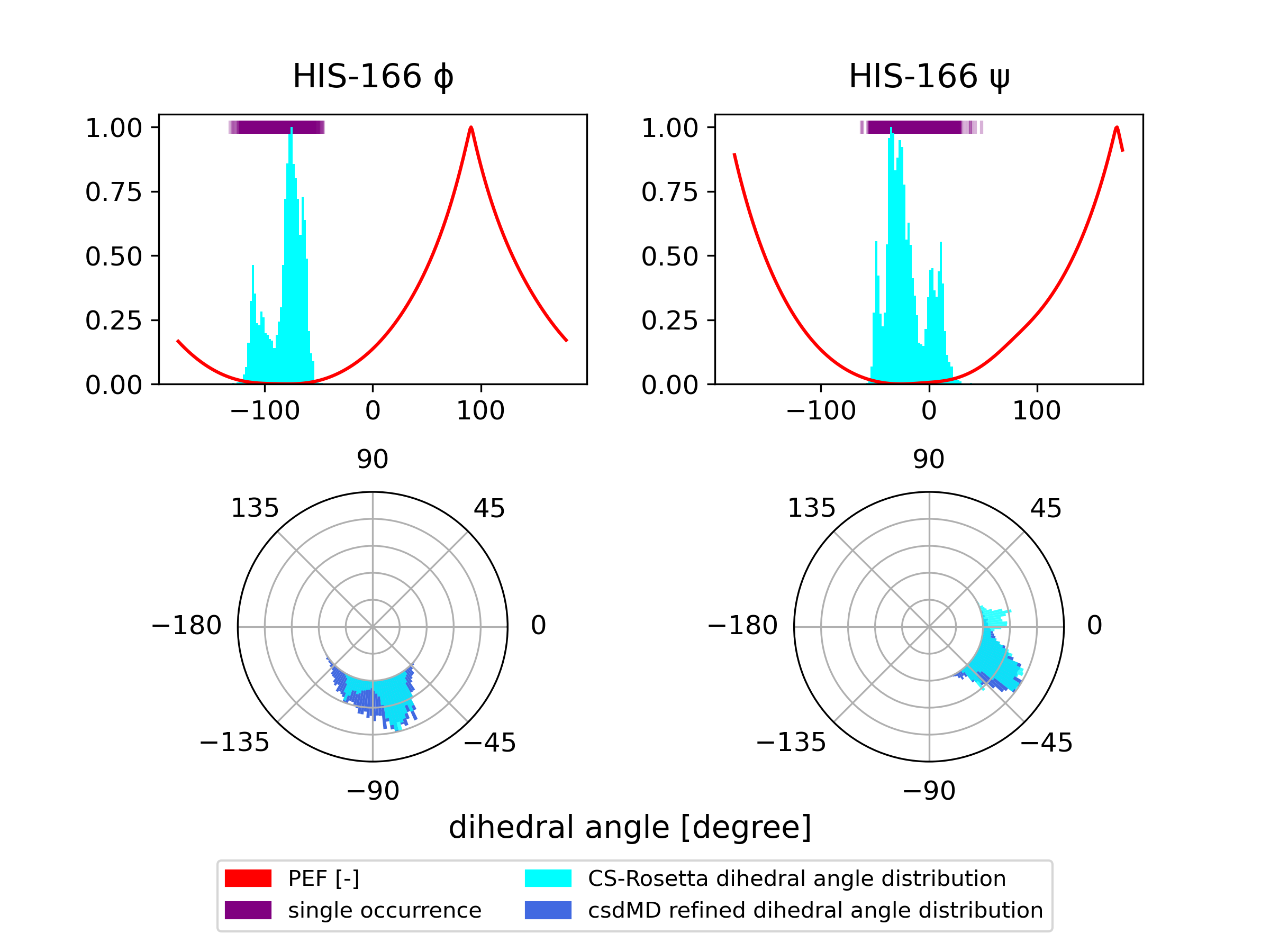

Supplement: Supplementary file 1 [file ijms-24-12101-s001.zip › KRAS-G12C-GDP-Mg-free_angle_figures/166-HIS.png]

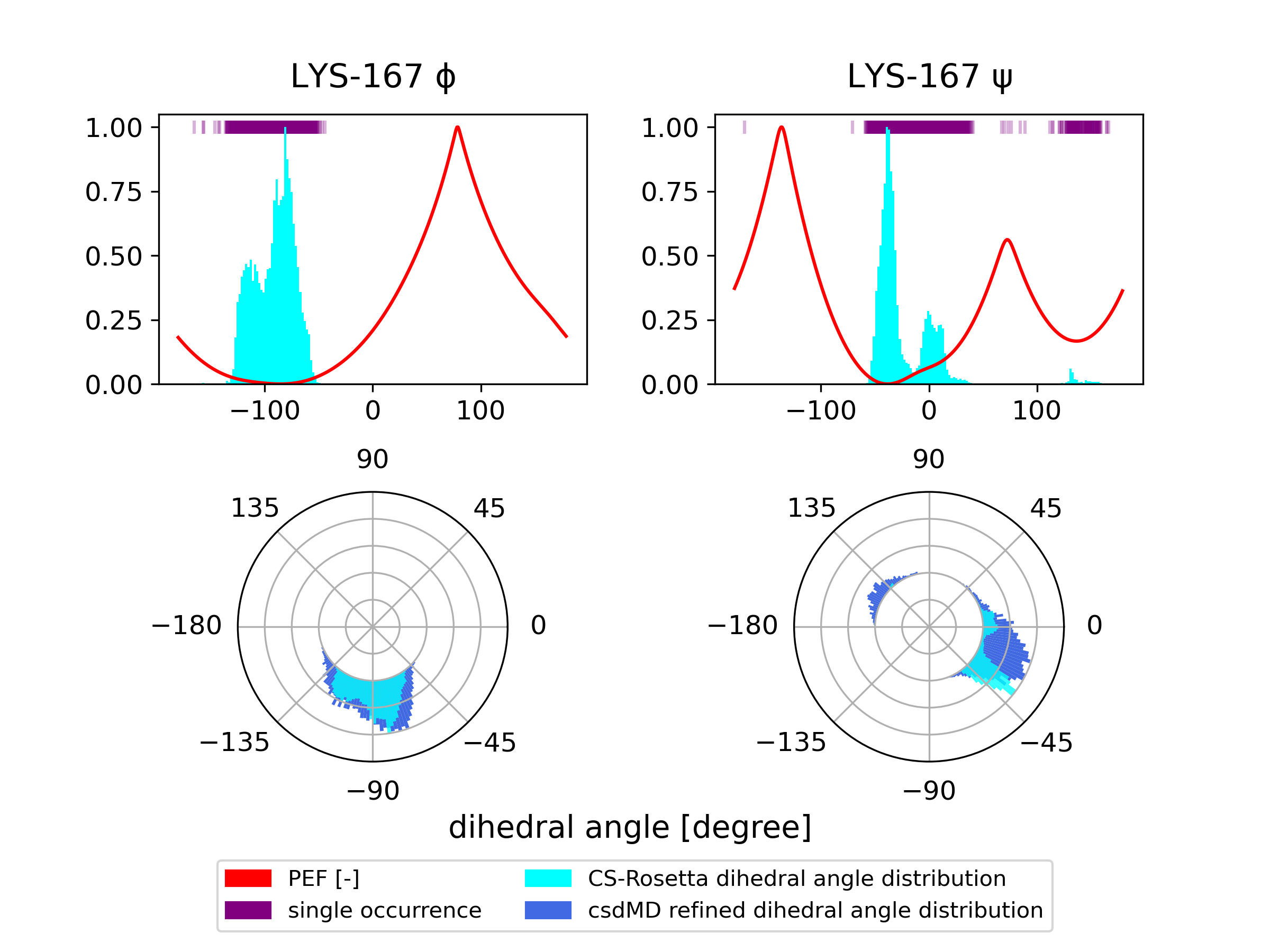

Supplement: Supplementary file 1 [file ijms-24-12101-s001.zip › KRAS-G12C-GDP-Mg-free_angle_figures/167-LYS.png]

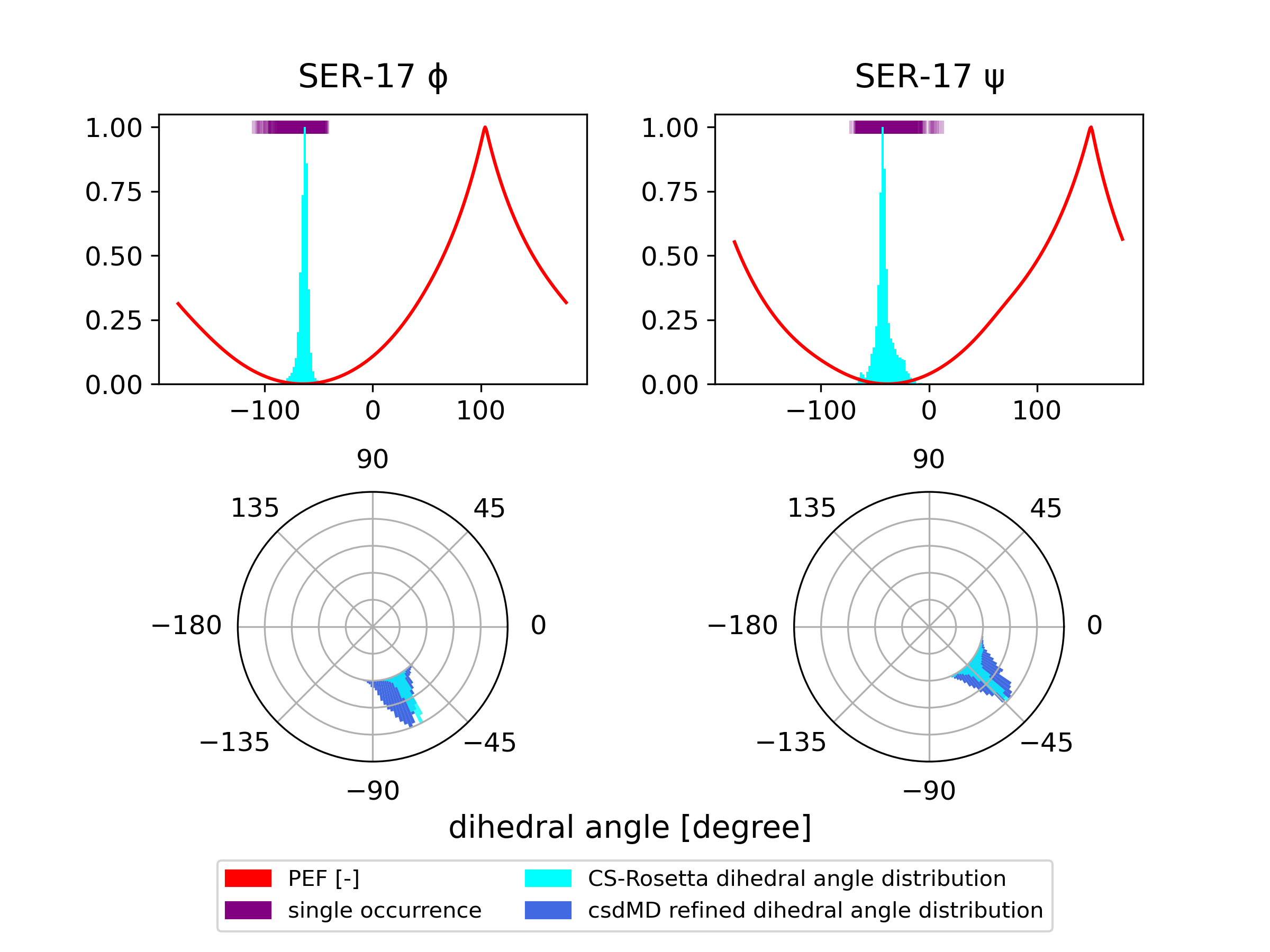

Supplement: Supplementary file 1 [file ijms-24-12101-s001.zip › KRAS-G12C-GDP-Mg-free_angle_figures/17-SER.png]

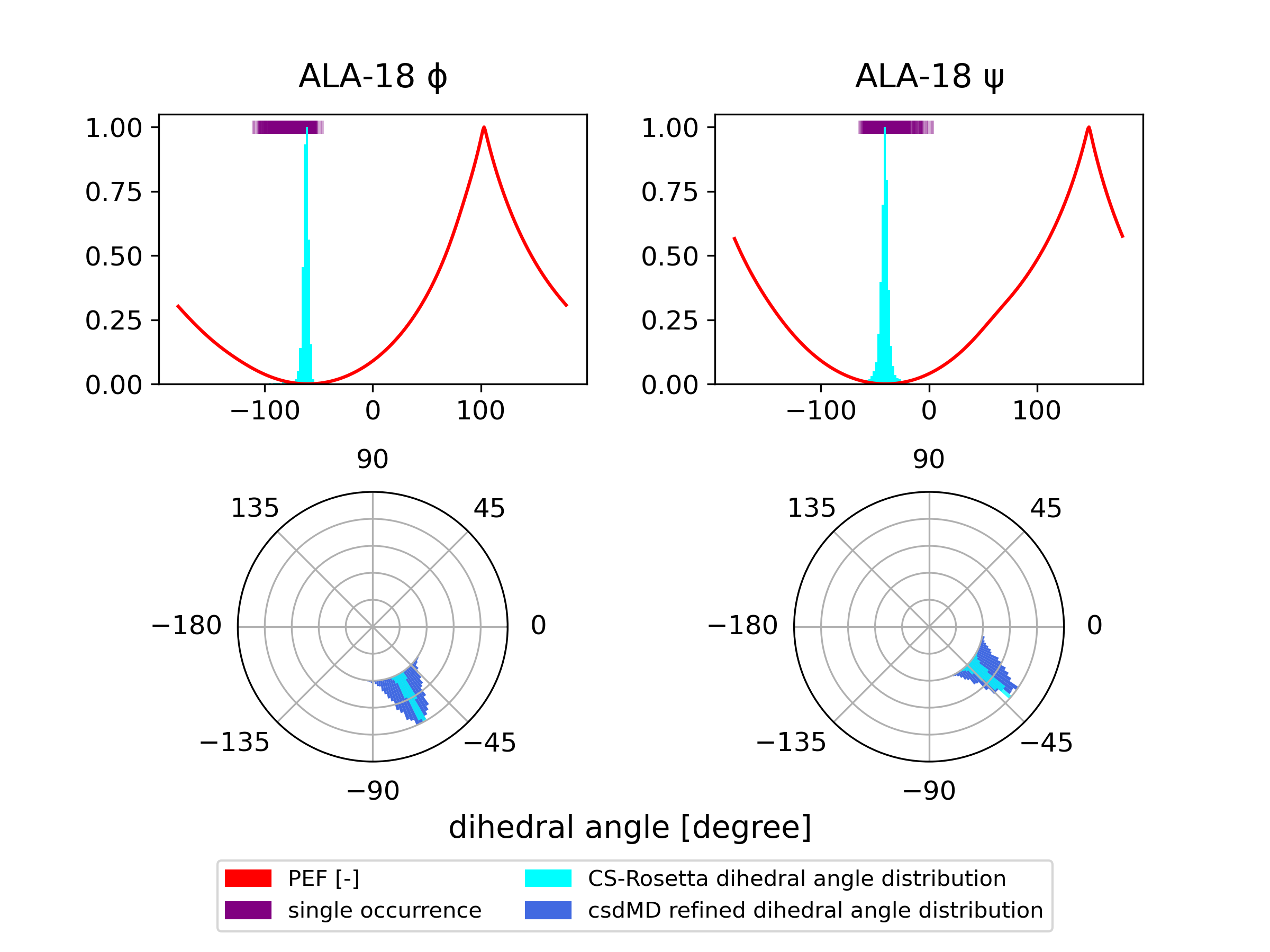

Supplement: Supplementary file 1 [file ijms-24-12101-s001.zip › KRAS-G12C-GDP-Mg-free_angle_figures/18-ALA.png]

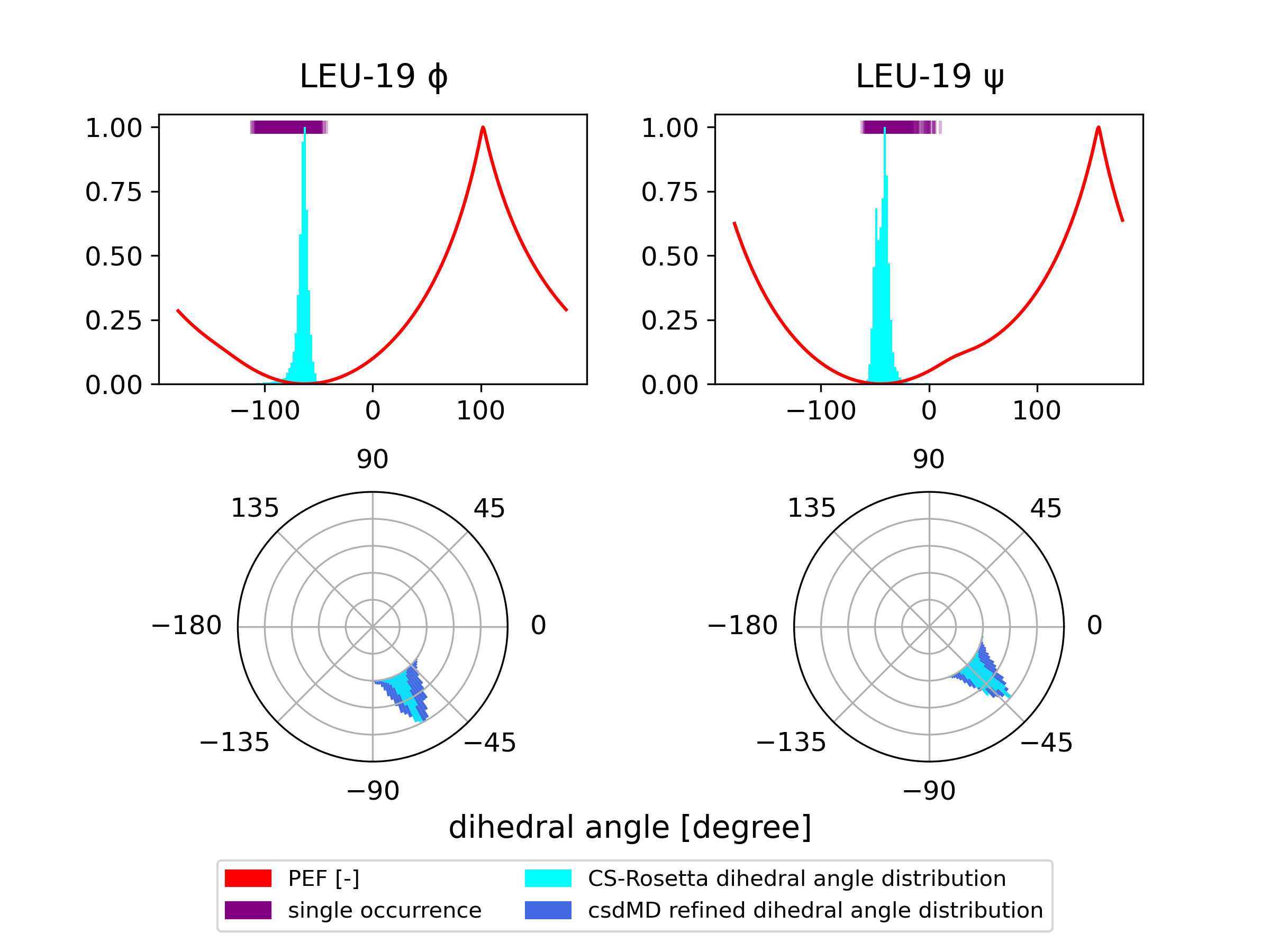

Supplement: Supplementary file 1 [file ijms-24-12101-s001.zip › KRAS-G12C-GDP-Mg-free_angle_figures/19-LEU.png]

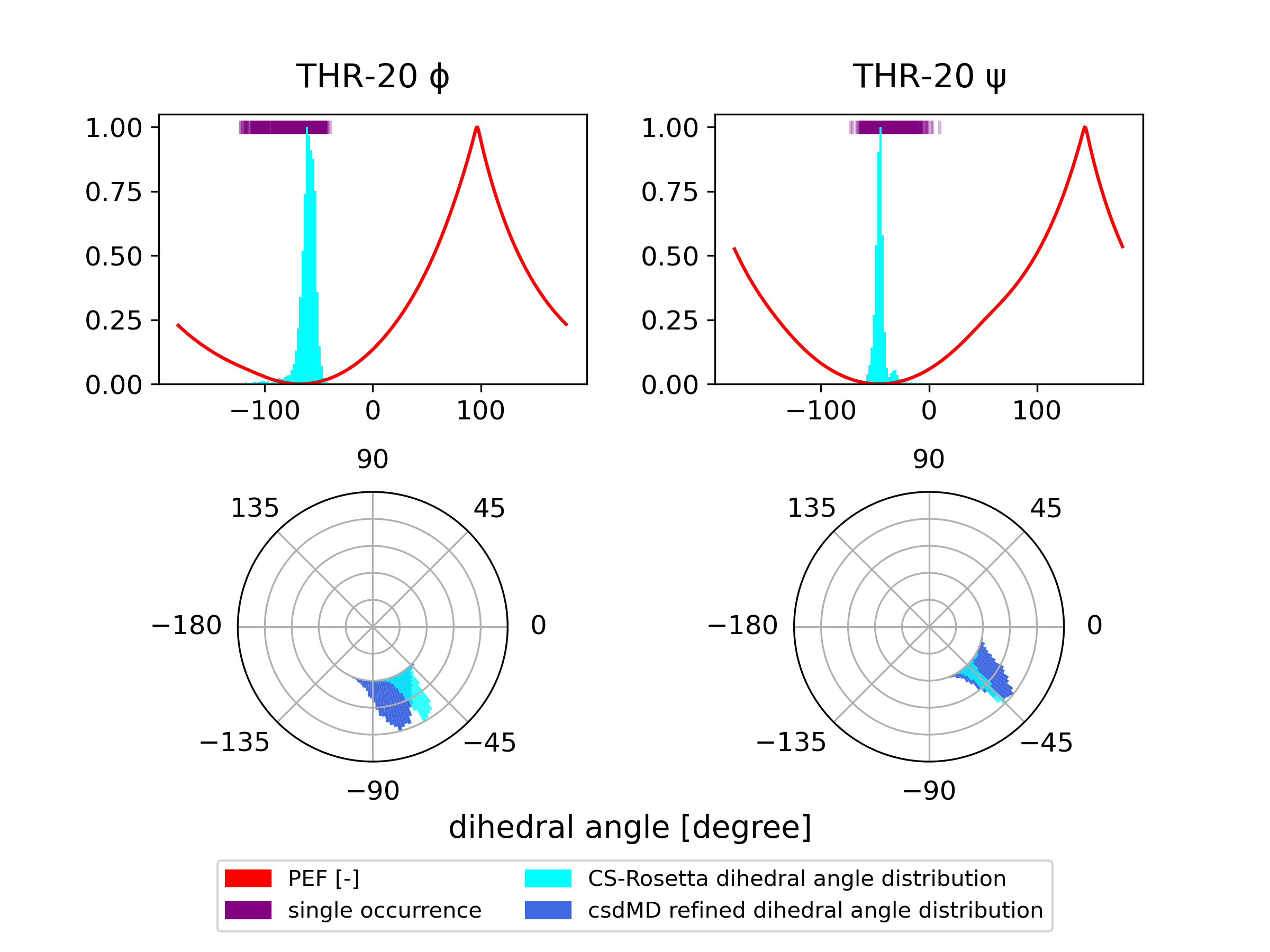

Supplement: Supplementary file 1 [file ijms-24-12101-s001.zip › KRAS-G12C-GDP-Mg-free_angle_figures/20-THR.png]

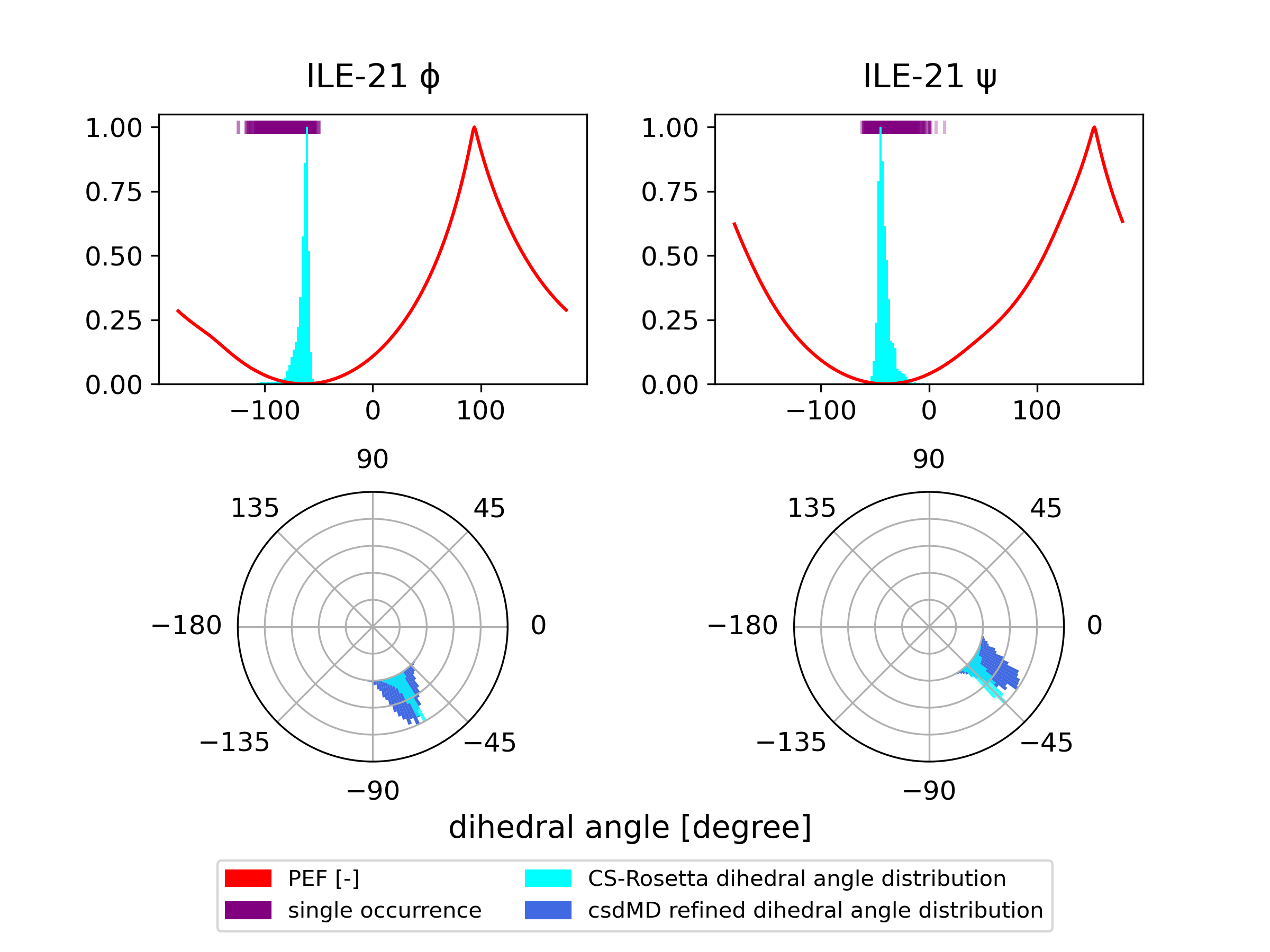

Supplement: Supplementary file 1 [file ijms-24-12101-s001.zip › KRAS-G12C-GDP-Mg-free_angle_figures/21-ILE.png]

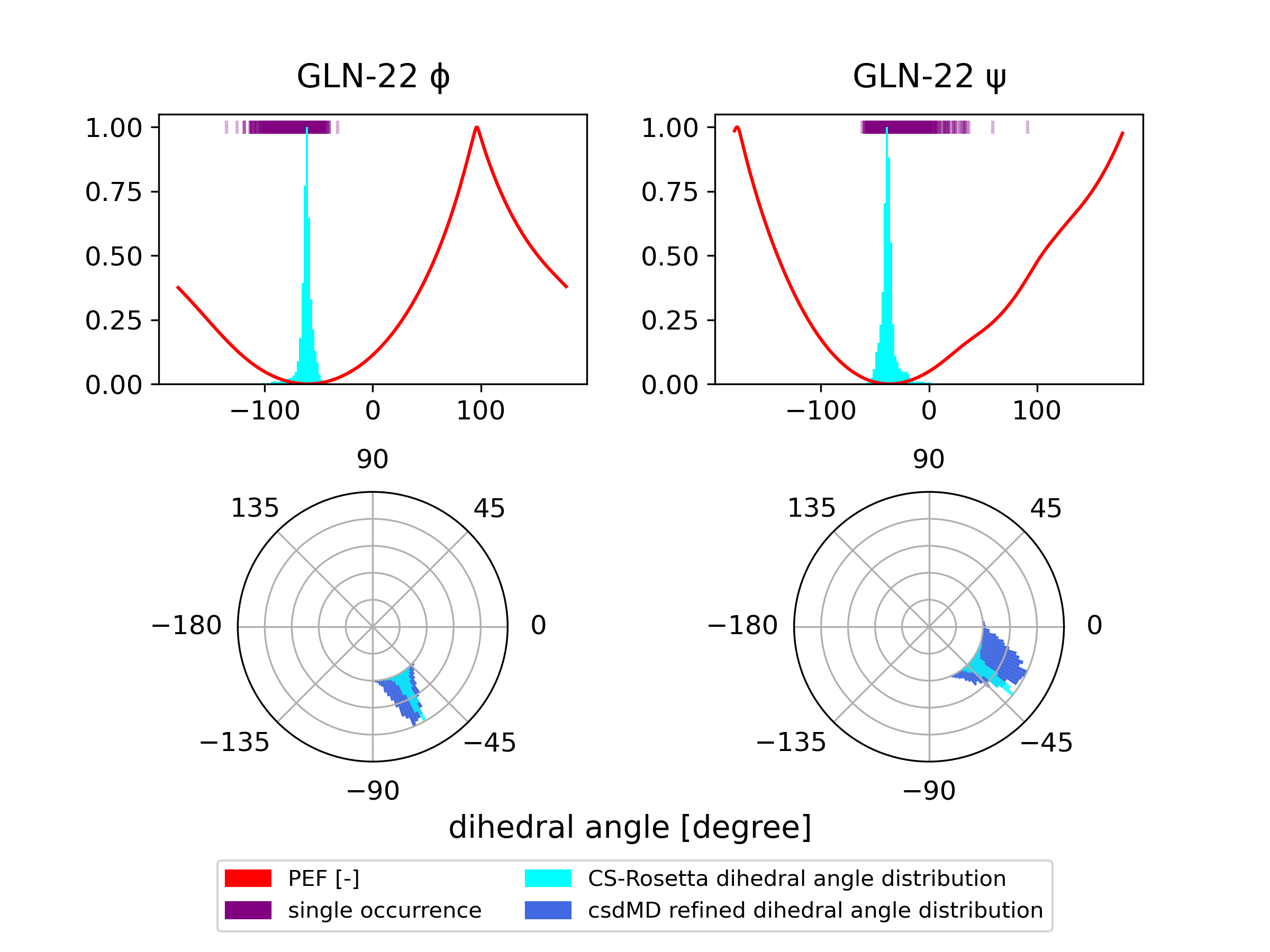

Supplement: Supplementary file 1 [file ijms-24-12101-s001.zip › KRAS-G12C-GDP-Mg-free_angle_figures/22-GLN.png]

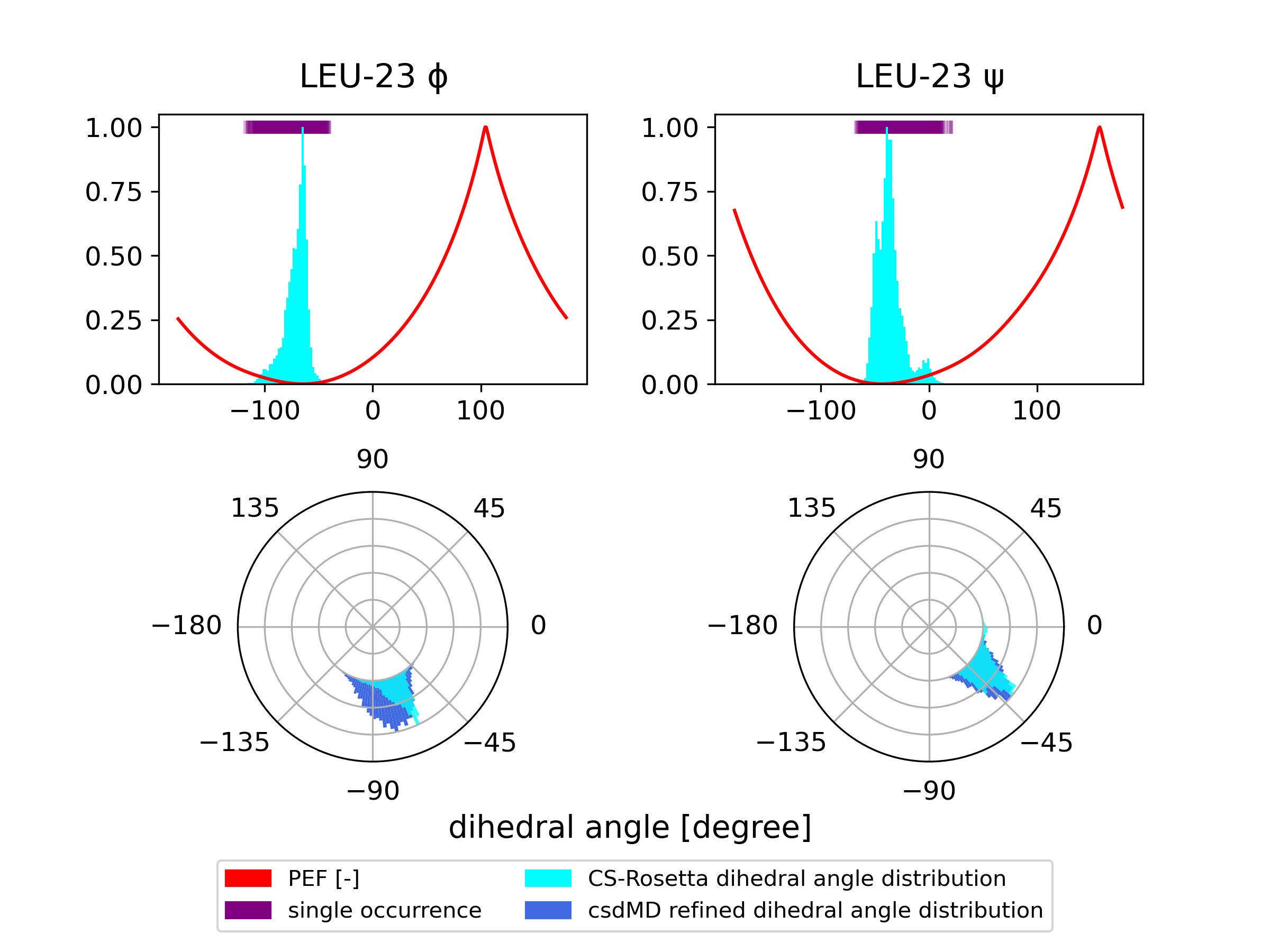

Supplement: Supplementary file 1 [file ijms-24-12101-s001.zip › KRAS-G12C-GDP-Mg-free_angle_figures/23-LEU.png]

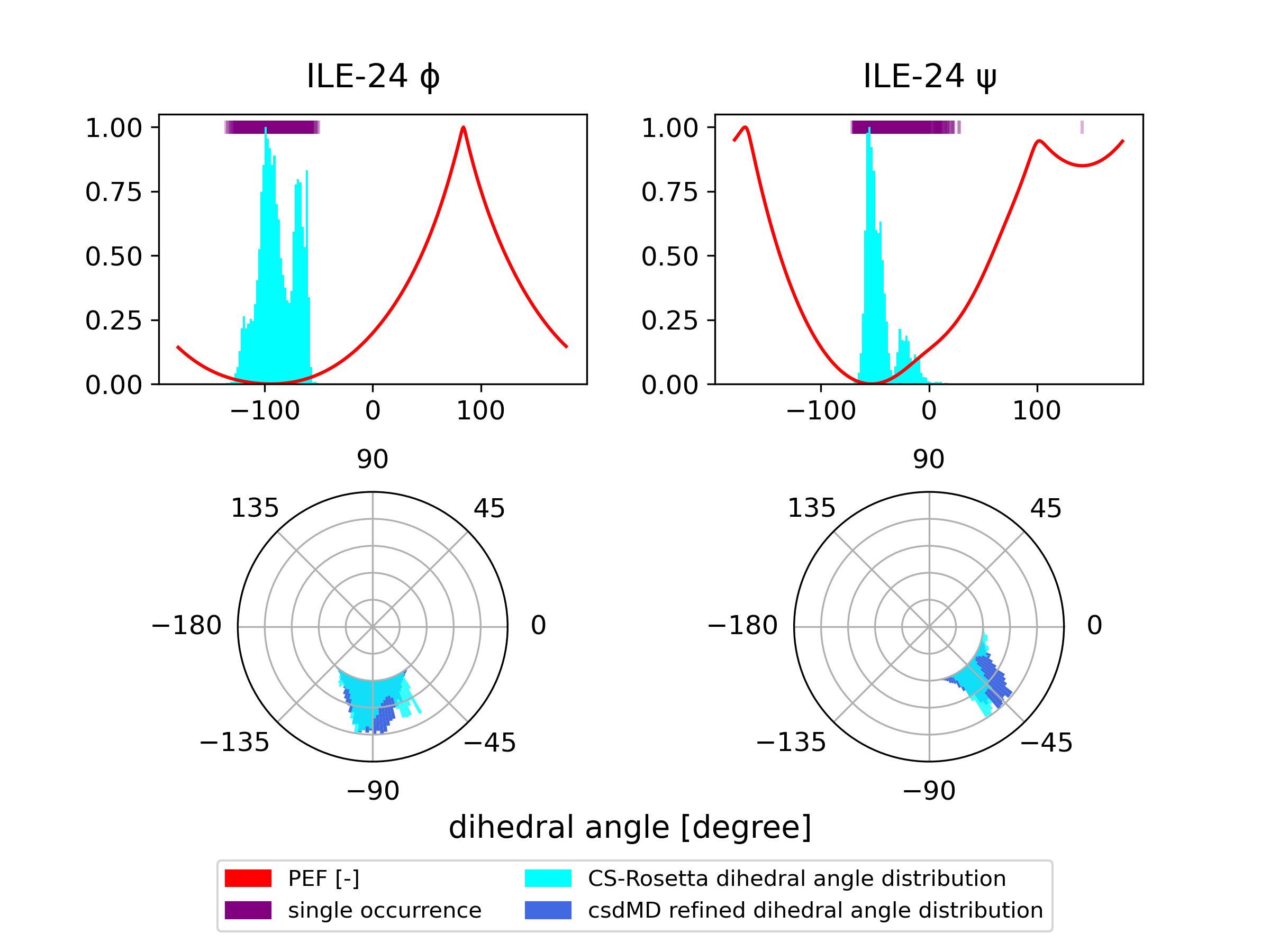

Supplement: Supplementary file 1 [file ijms-24-12101-s001.zip › KRAS-G12C-GDP-Mg-free_angle_figures/24-ILE.png]

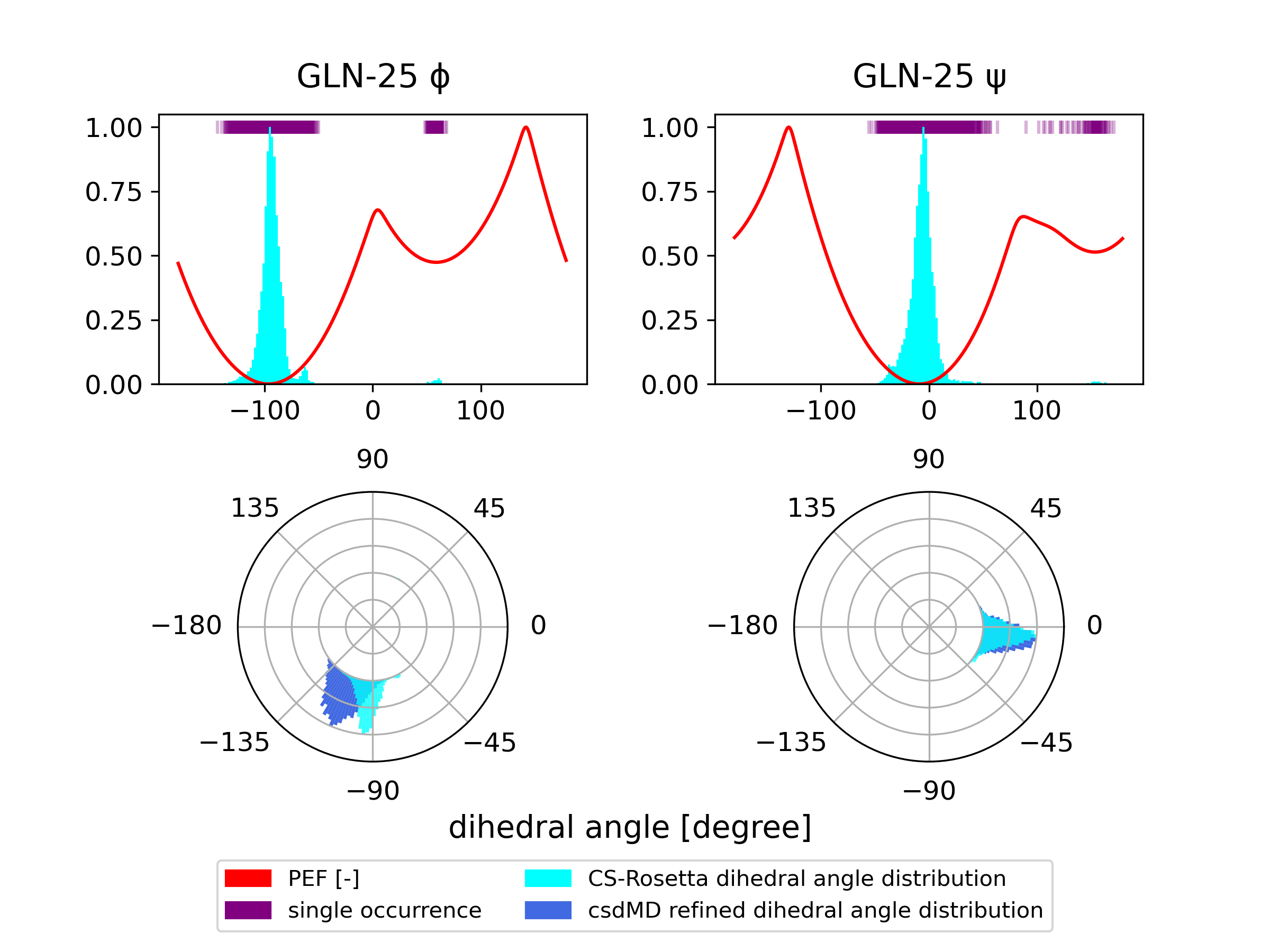

Supplement: Supplementary file 1 [file ijms-24-12101-s001.zip › KRAS-G12C-GDP-Mg-free_angle_figures/25-GLN.png]

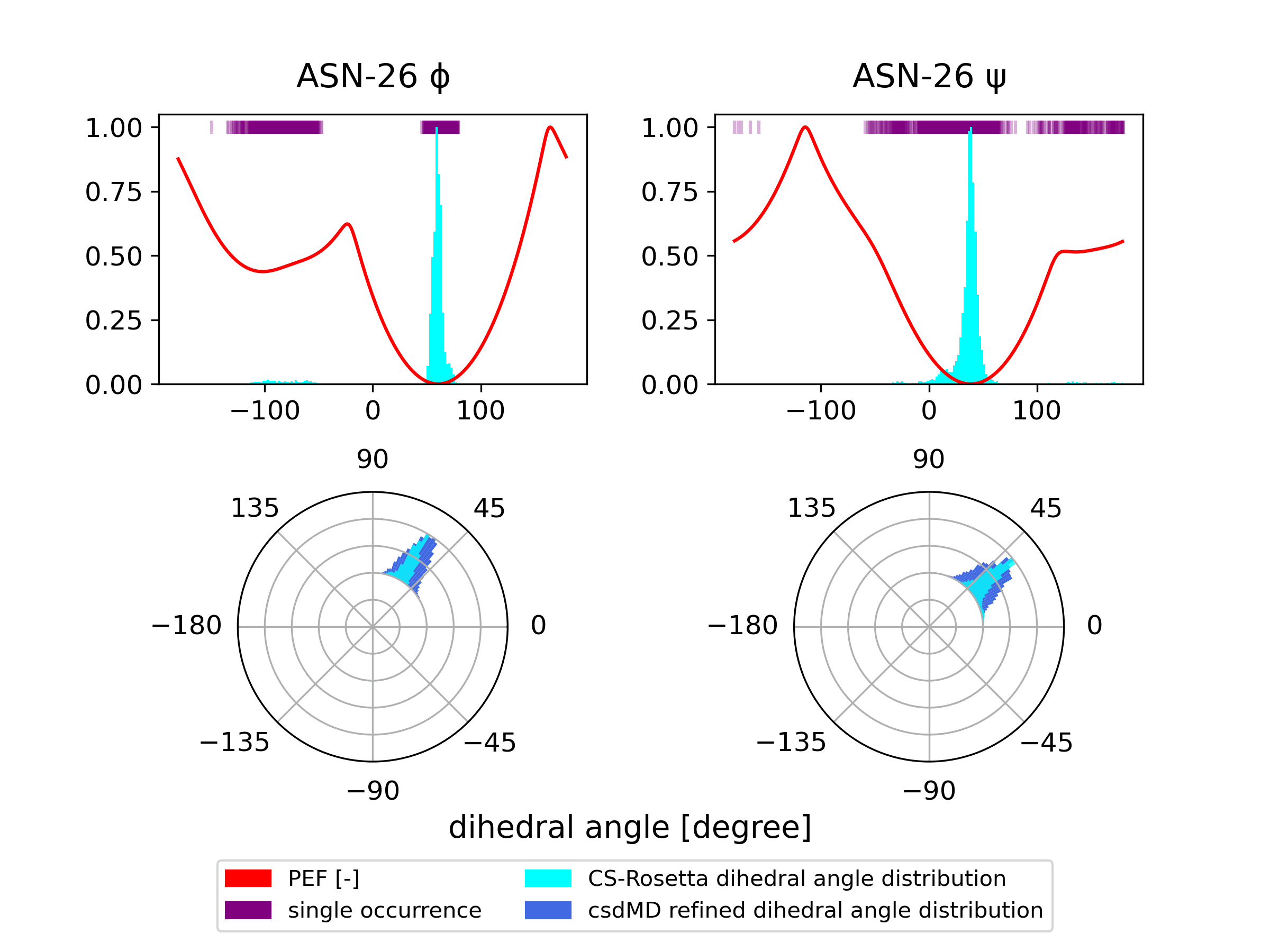

Supplement: Supplementary file 1 [file ijms-24-12101-s001.zip › KRAS-G12C-GDP-Mg-free_angle_figures/26-ASN.png]

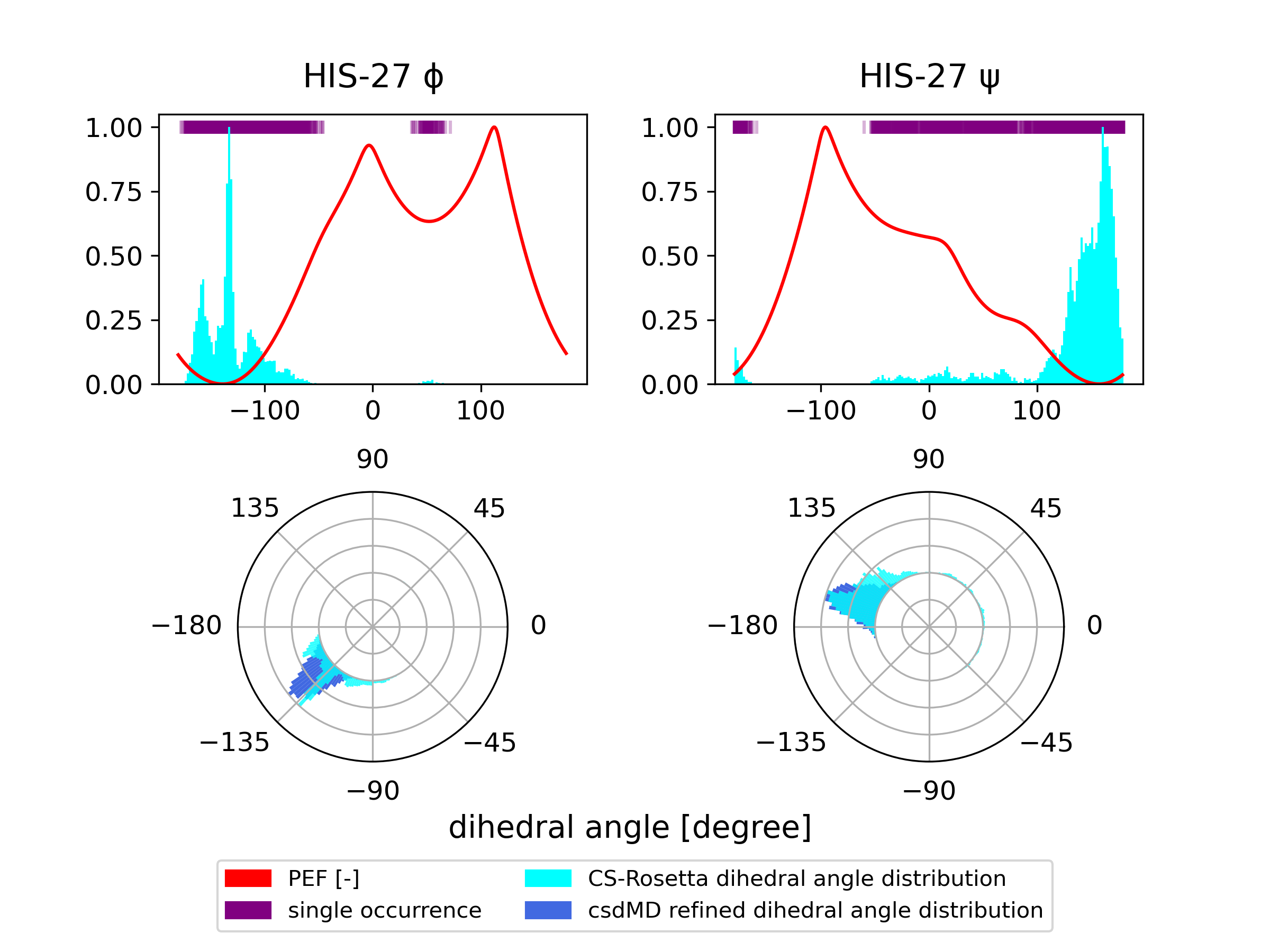

Supplement: Supplementary file 1 [file ijms-24-12101-s001.zip › KRAS-G12C-GDP-Mg-free_angle_figures/27-HIS.png]

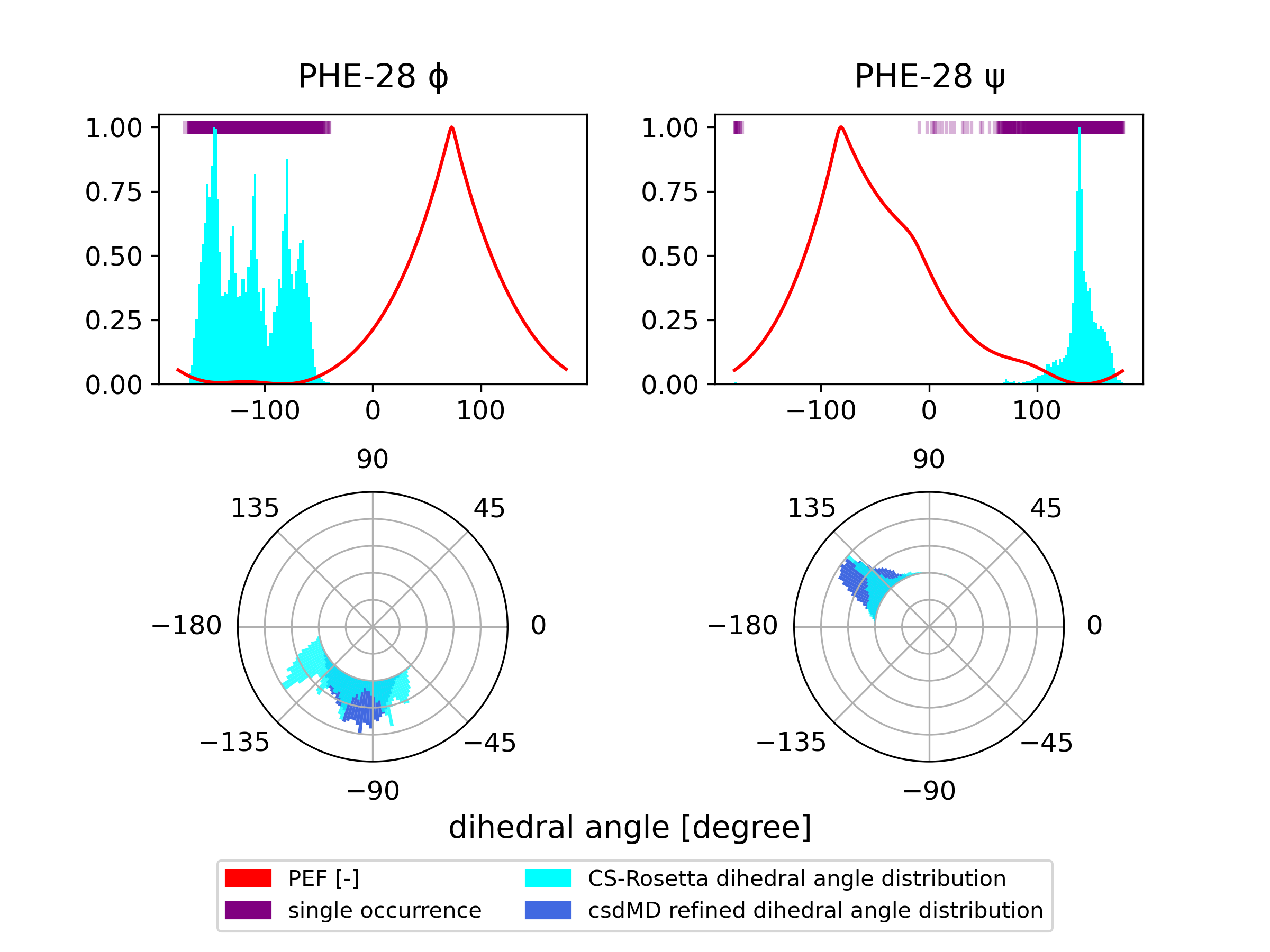

Supplement: Supplementary file 1 [file ijms-24-12101-s001.zip › KRAS-G12C-GDP-Mg-free_angle_figures/28-PHE.png]

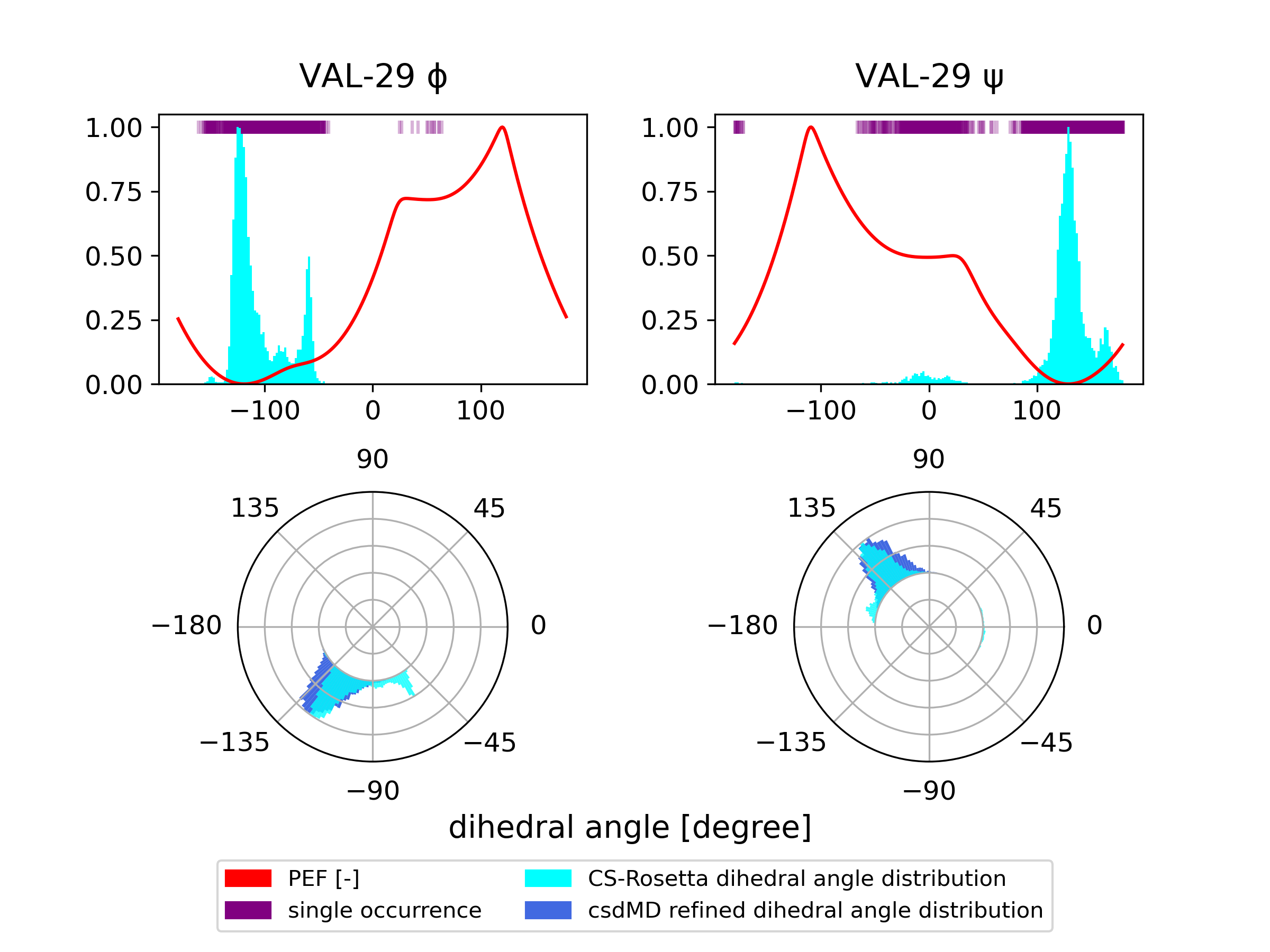

Supplement: Supplementary file 1 [file ijms-24-12101-s001.zip › KRAS-G12C-GDP-Mg-free_angle_figures/29-VAL.png]

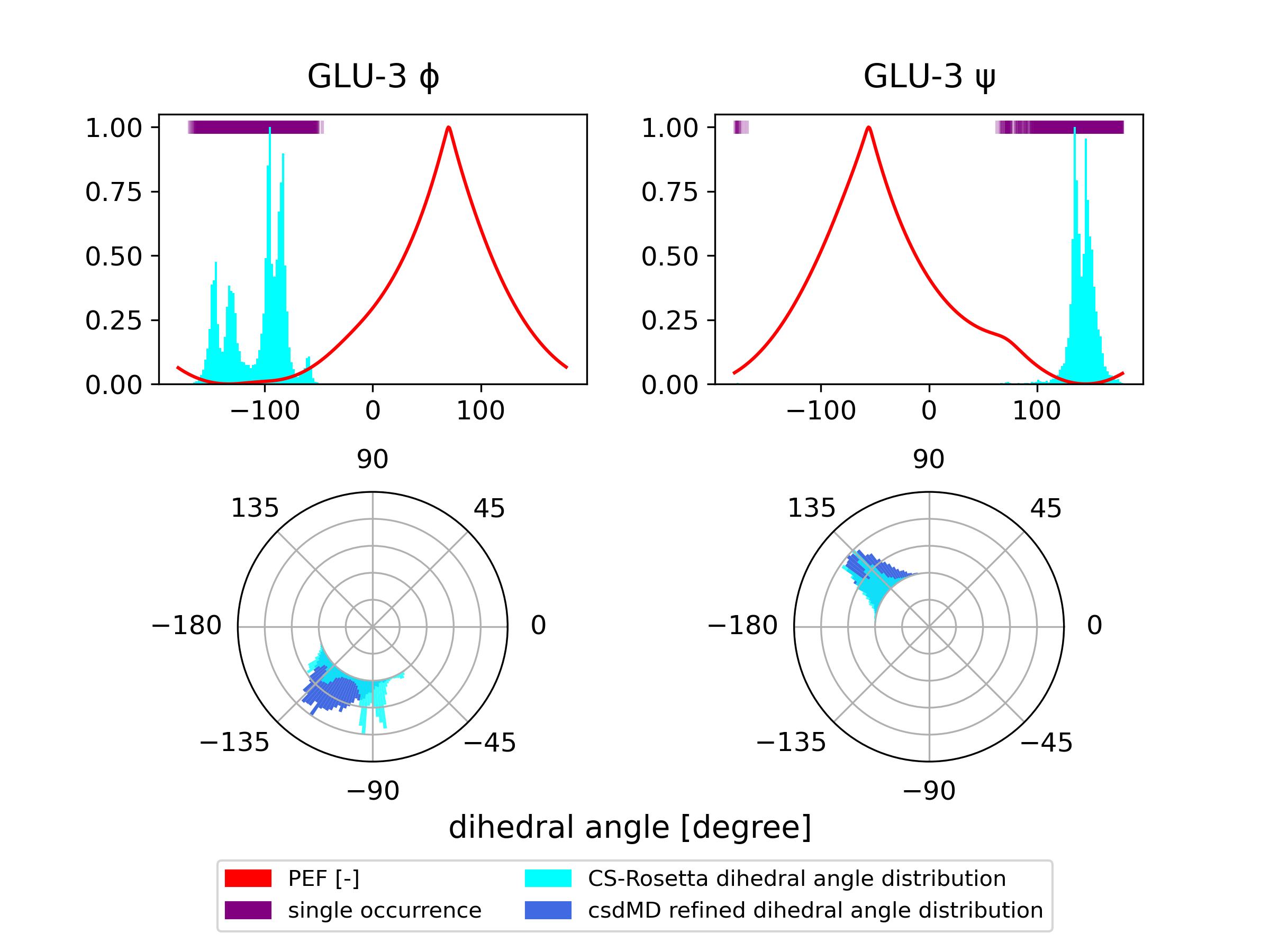

Supplement: Supplementary file 1 [file ijms-24-12101-s001.zip › KRAS-G12C-GDP-Mg-free_angle_figures/3-GLU.png]

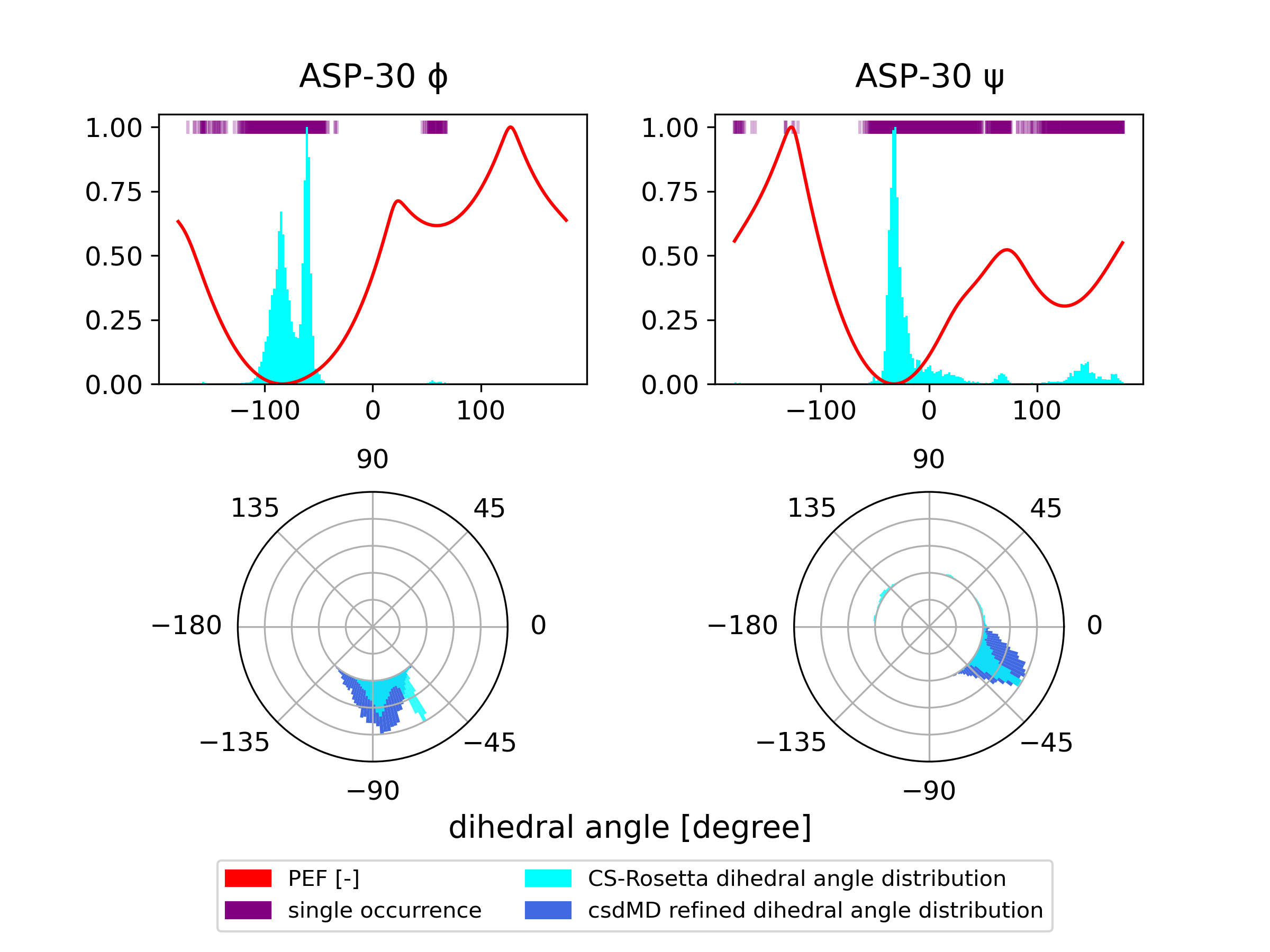

Supplement: Supplementary file 1 [file ijms-24-12101-s001.zip › KRAS-G12C-GDP-Mg-free_angle_figures/30-ASP.png]

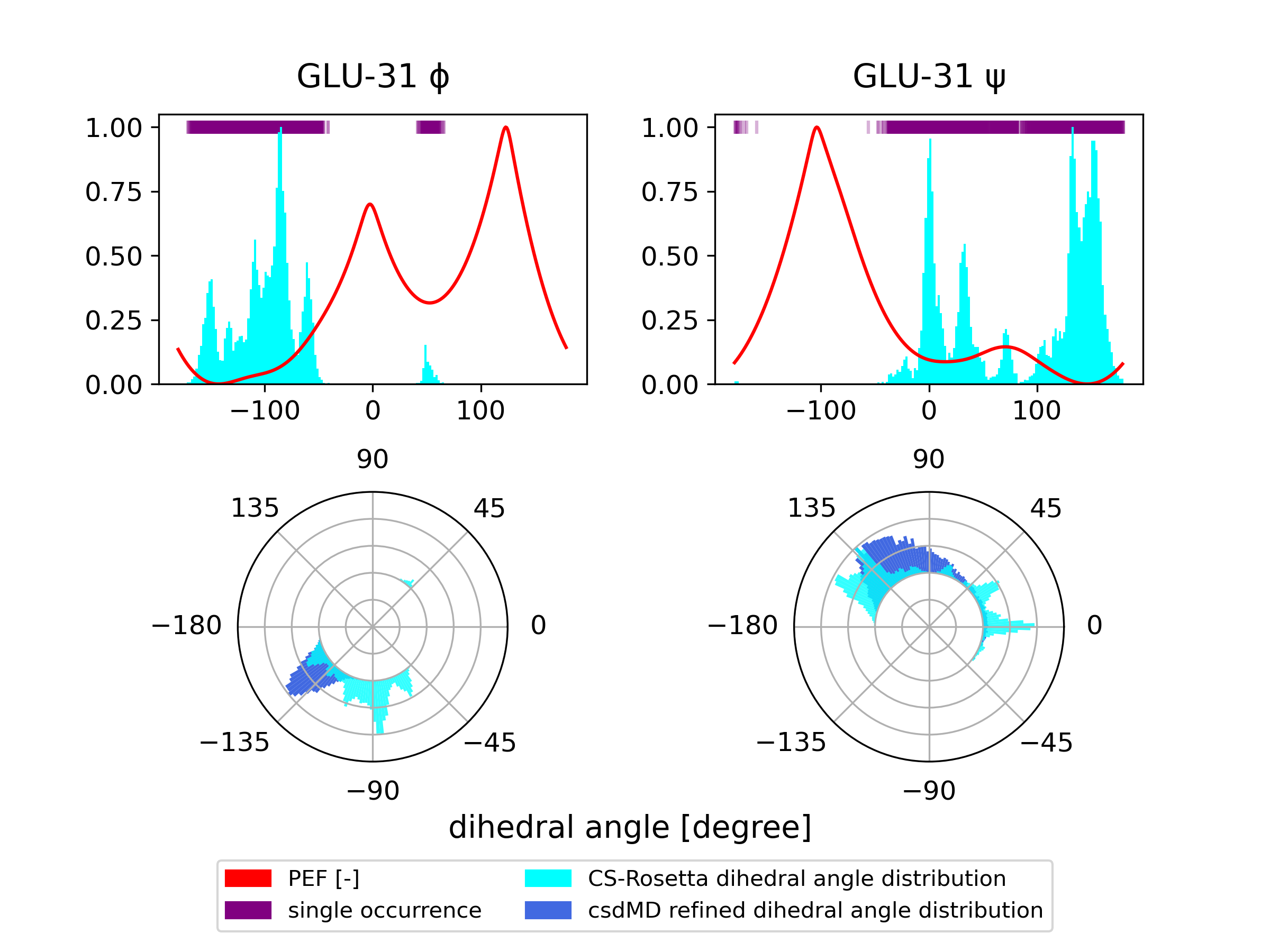

Supplement: Supplementary file 1 [file ijms-24-12101-s001.zip › KRAS-G12C-GDP-Mg-free_angle_figures/31-GLU.png]

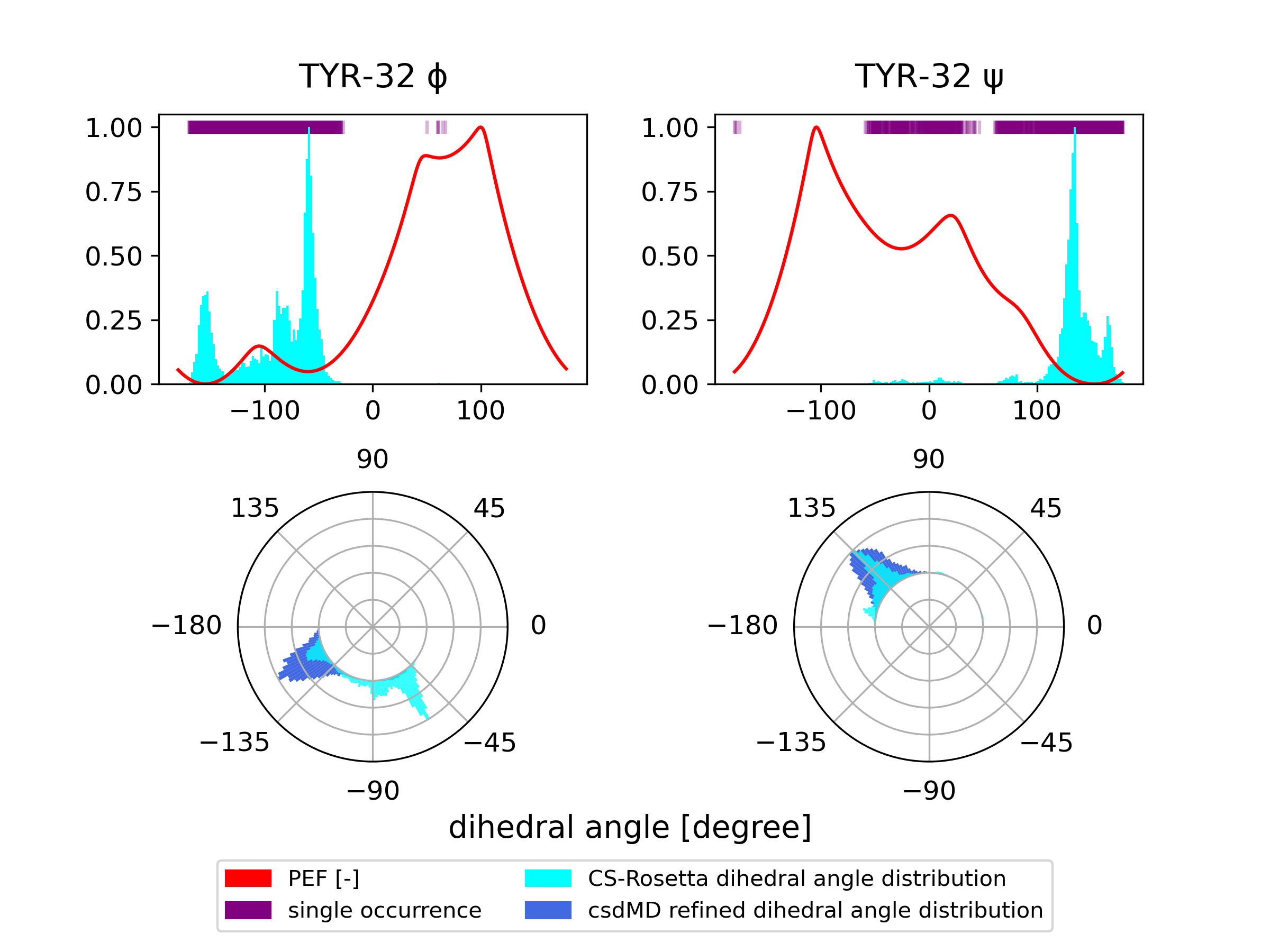

Supplement: Supplementary file 1 [file ijms-24-12101-s001.zip › KRAS-G12C-GDP-Mg-free_angle_figures/32-TYR.png]

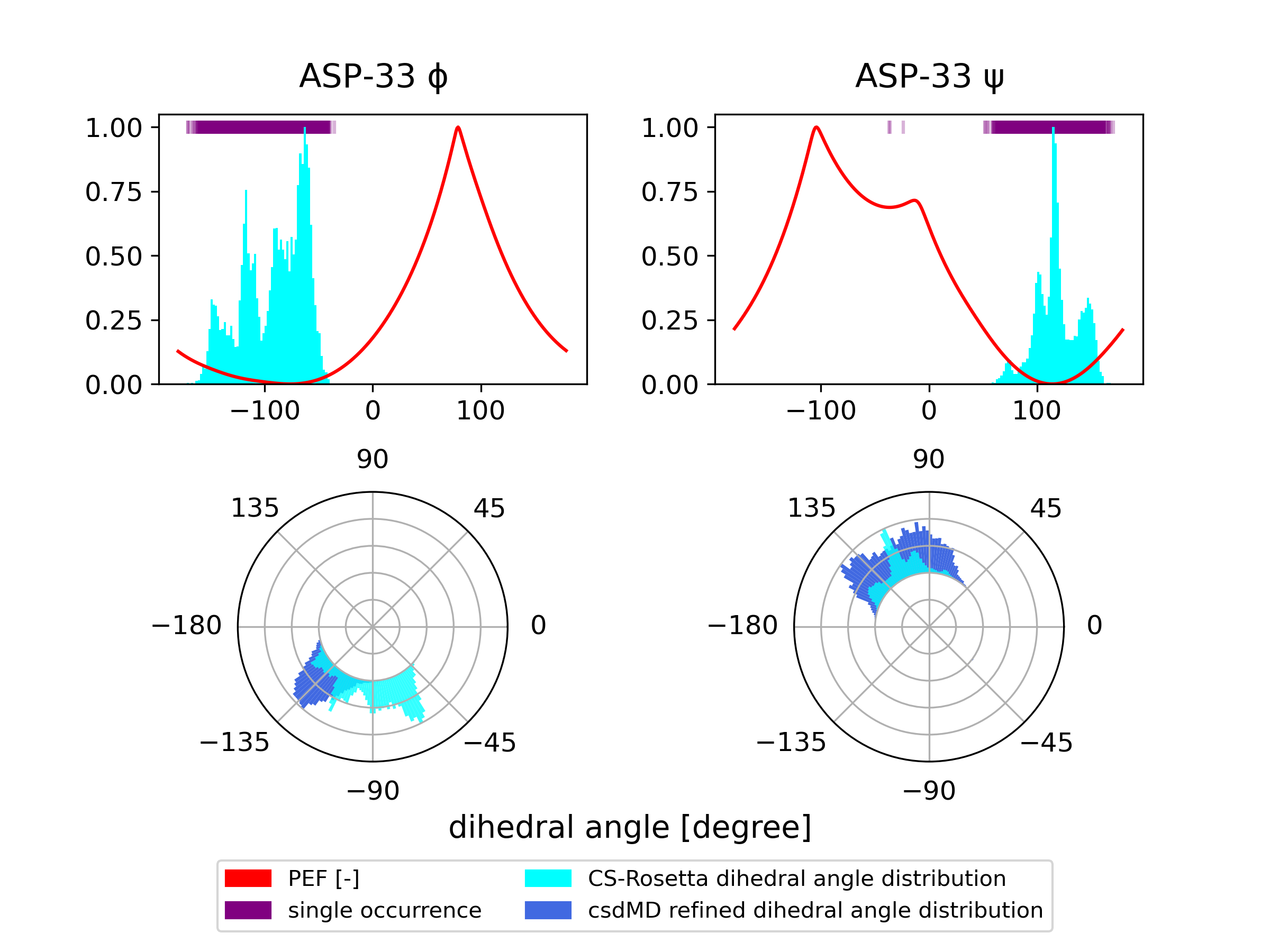

Supplement: Supplementary file 1 [file ijms-24-12101-s001.zip › KRAS-G12C-GDP-Mg-free_angle_figures/33-ASP.png]

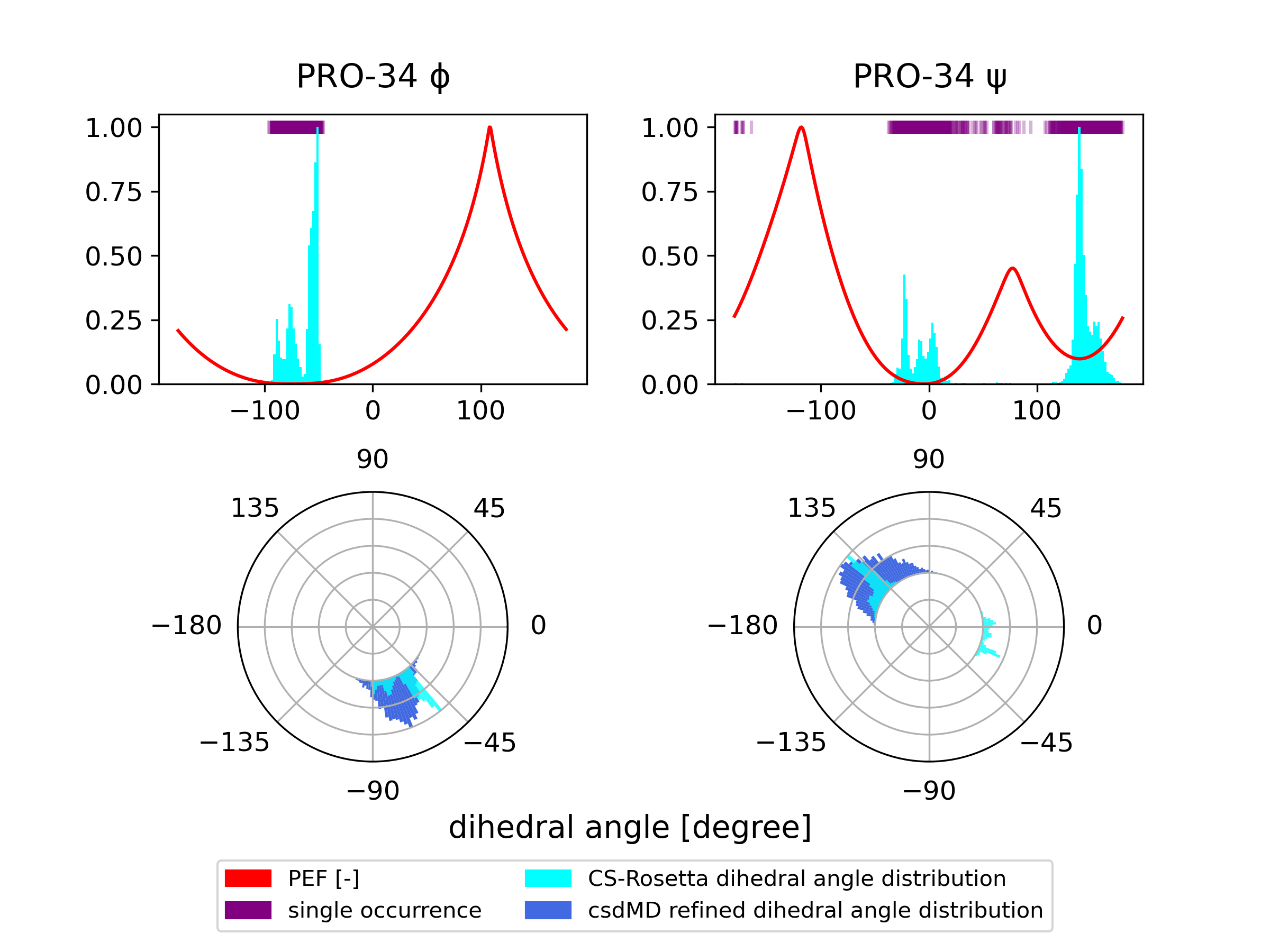

Supplement: Supplementary file 1 [file ijms-24-12101-s001.zip › KRAS-G12C-GDP-Mg-free_angle_figures/34-PRO.png]

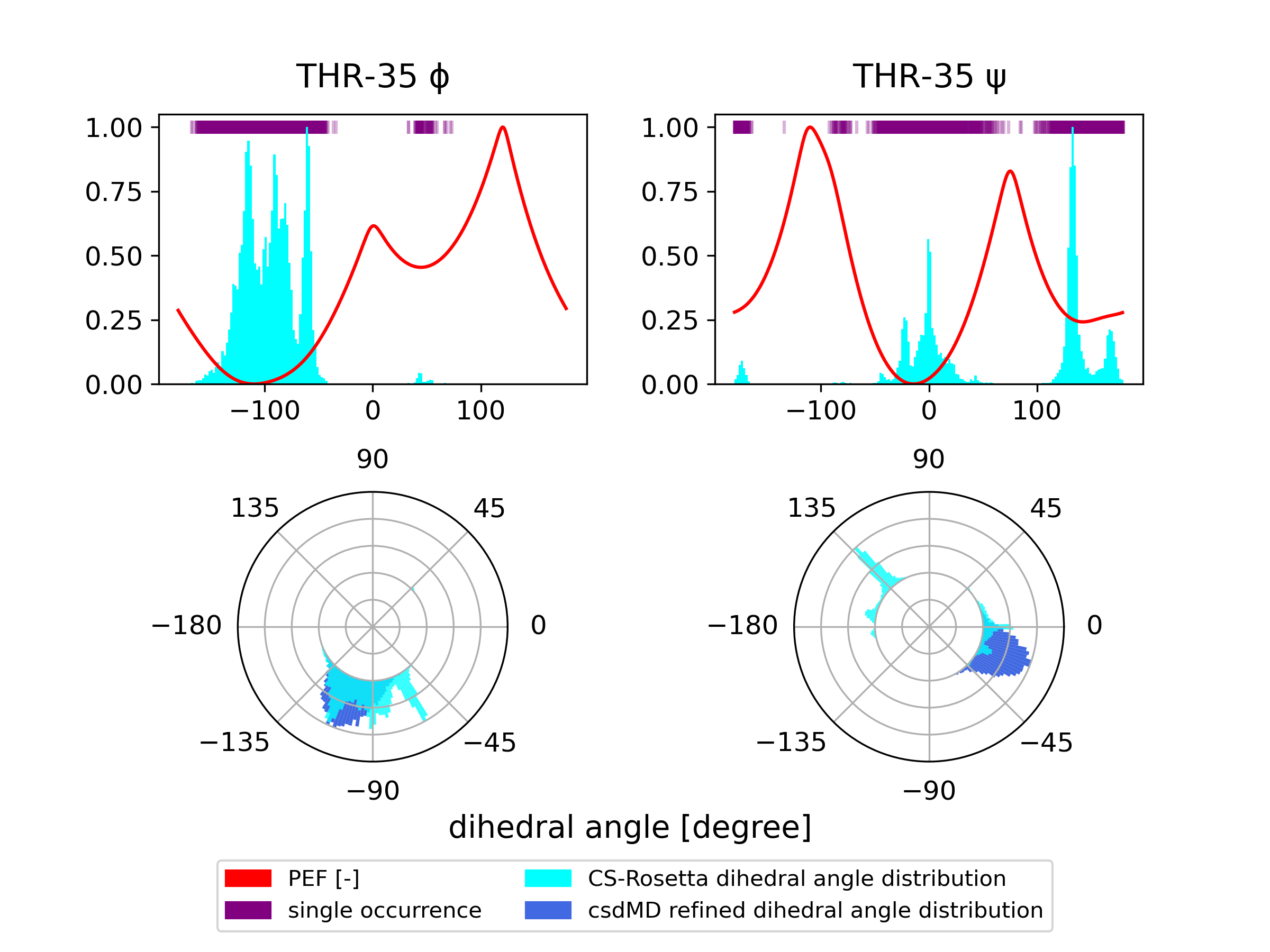

Supplement: Supplementary file 1 [file ijms-24-12101-s001.zip › KRAS-G12C-GDP-Mg-free_angle_figures/35-THR.png]

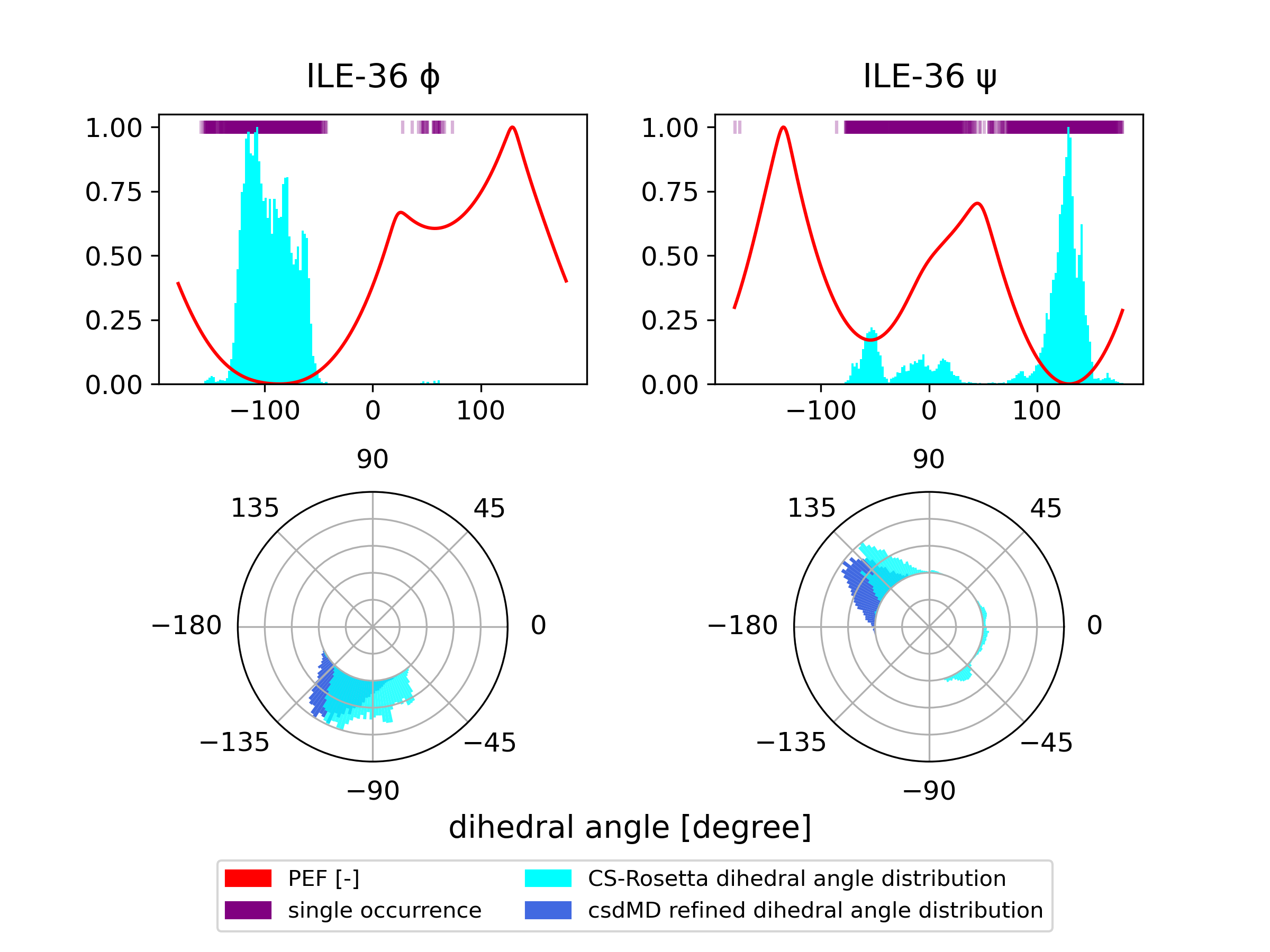

Supplement: Supplementary file 1 [file ijms-24-12101-s001.zip › KRAS-G12C-GDP-Mg-free_angle_figures/36-ILE.png]

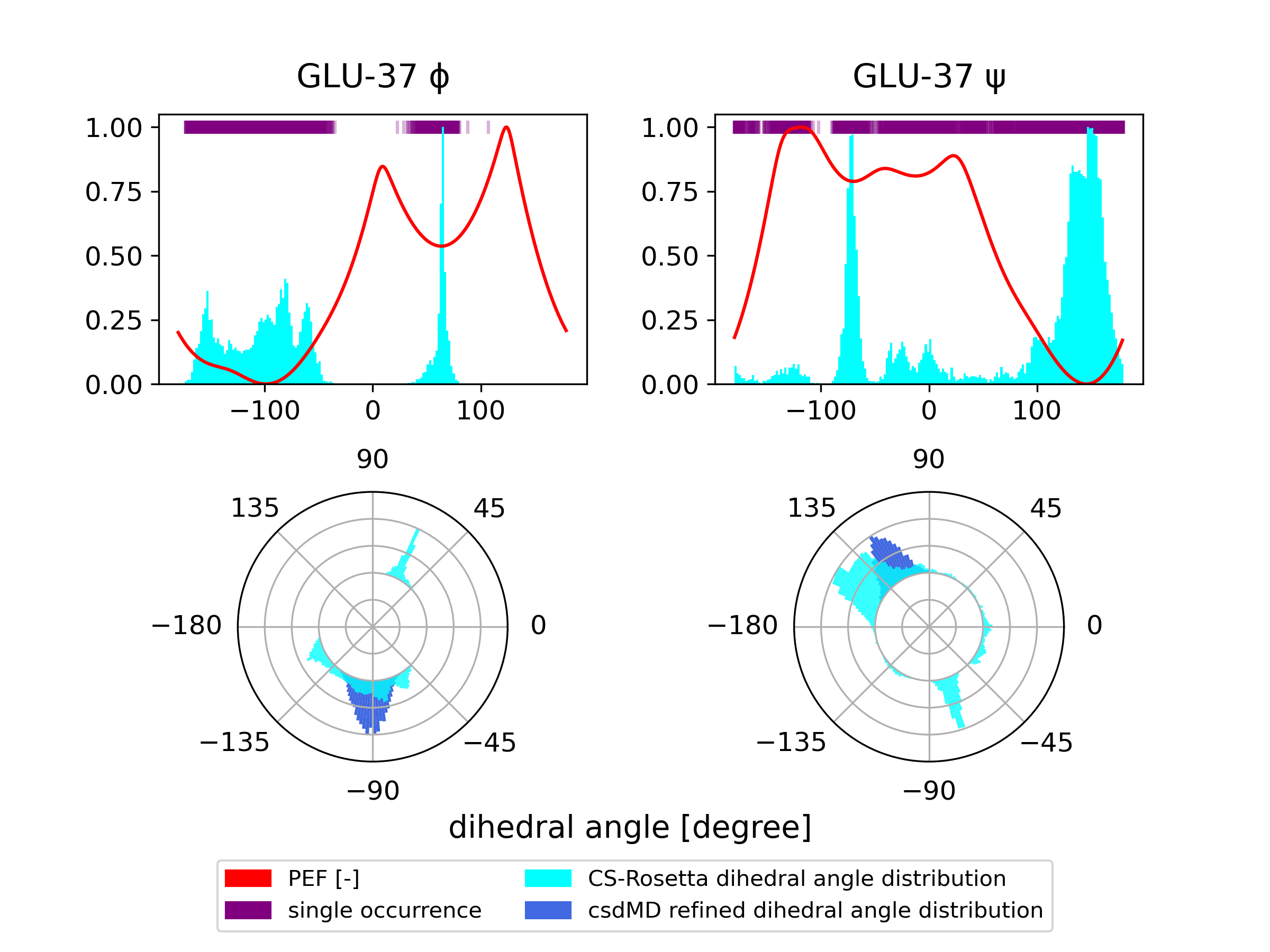

Supplement: Supplementary file 1 [file ijms-24-12101-s001.zip › KRAS-G12C-GDP-Mg-free_angle_figures/37-GLU.png]

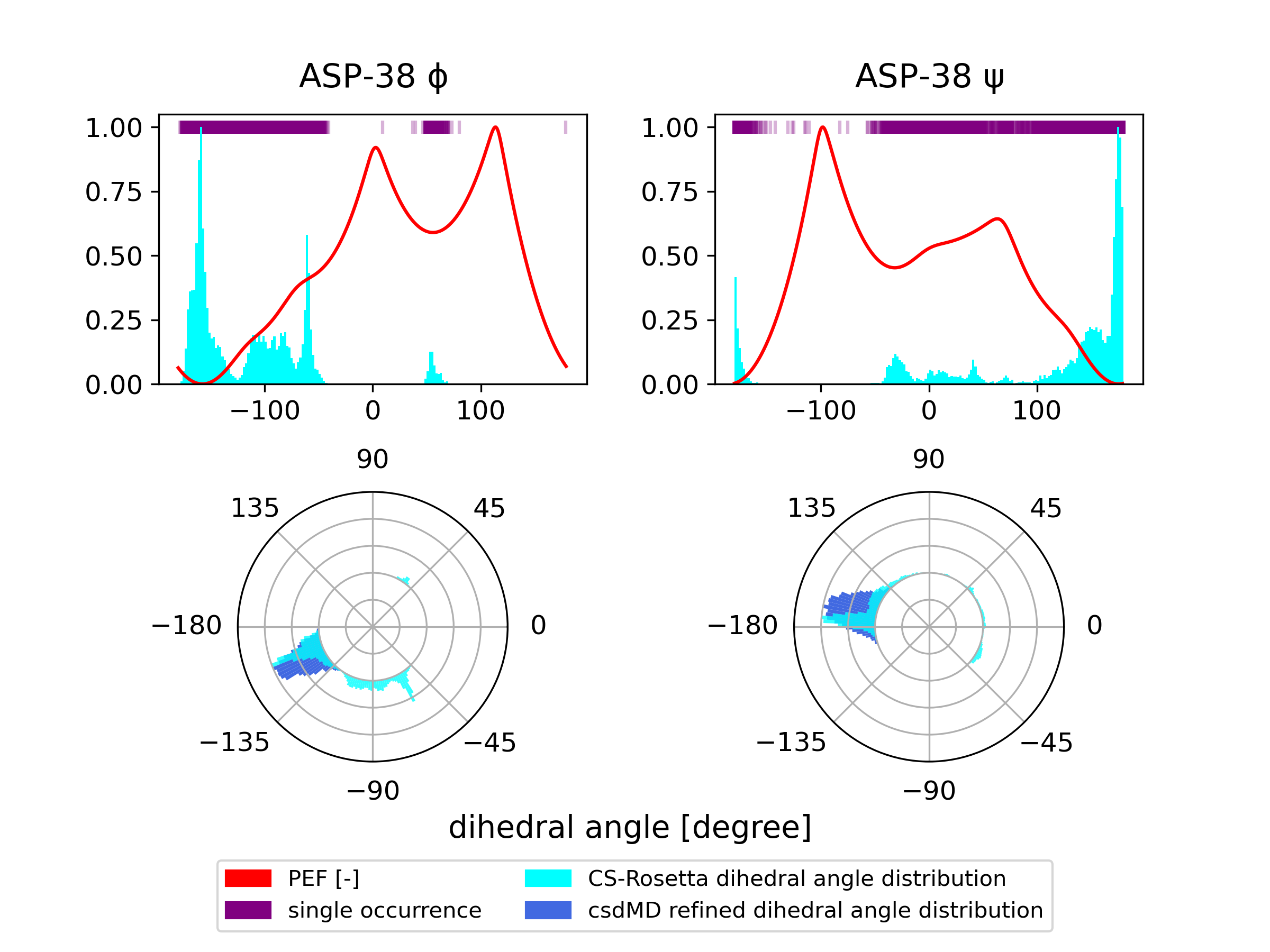

Supplement: Supplementary file 1 [file ijms-24-12101-s001.zip › KRAS-G12C-GDP-Mg-free_angle_figures/38-ASP.png]

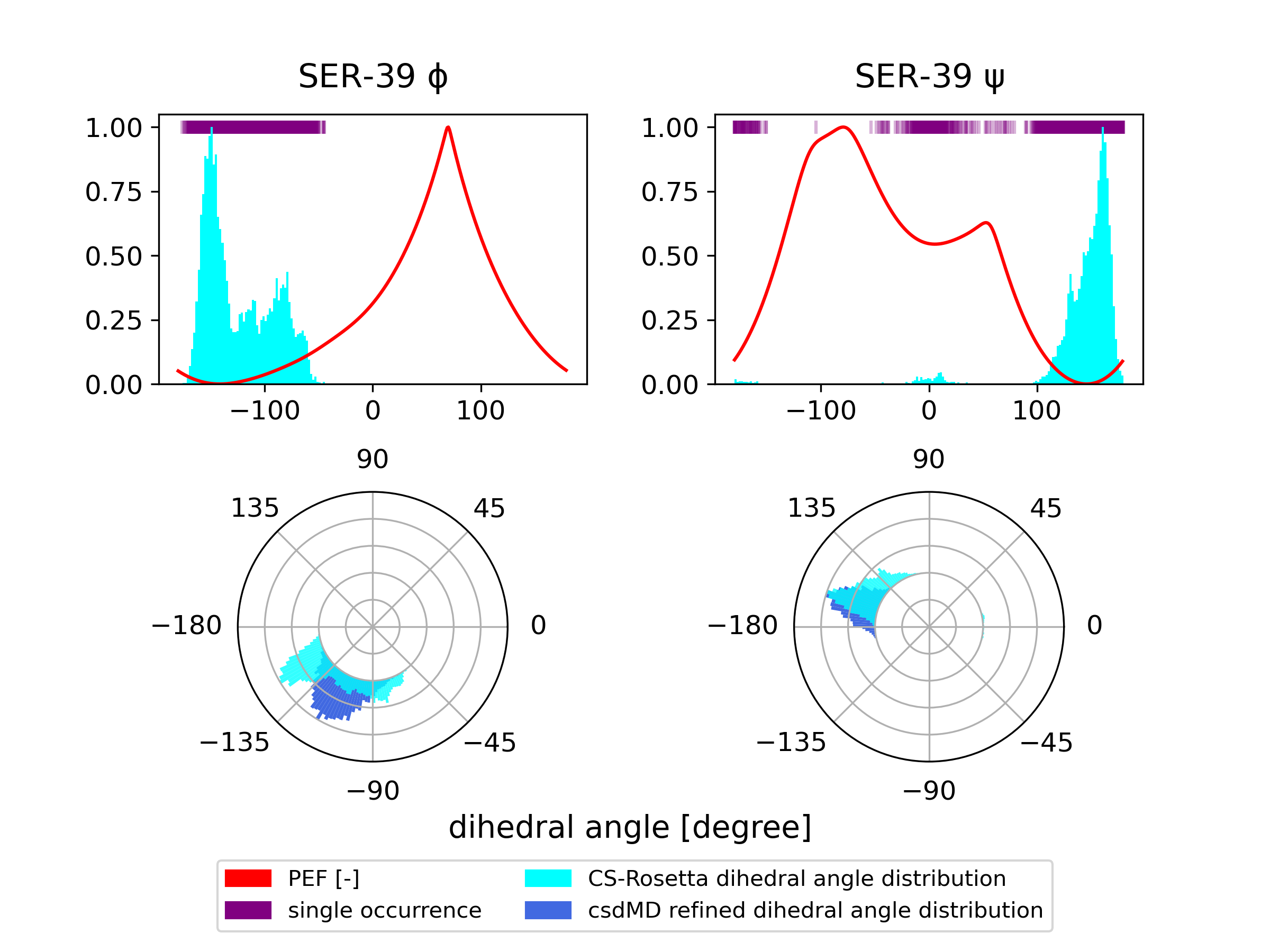

Supplement: Supplementary file 1 [file ijms-24-12101-s001.zip › KRAS-G12C-GDP-Mg-free_angle_figures/39-SER.png]

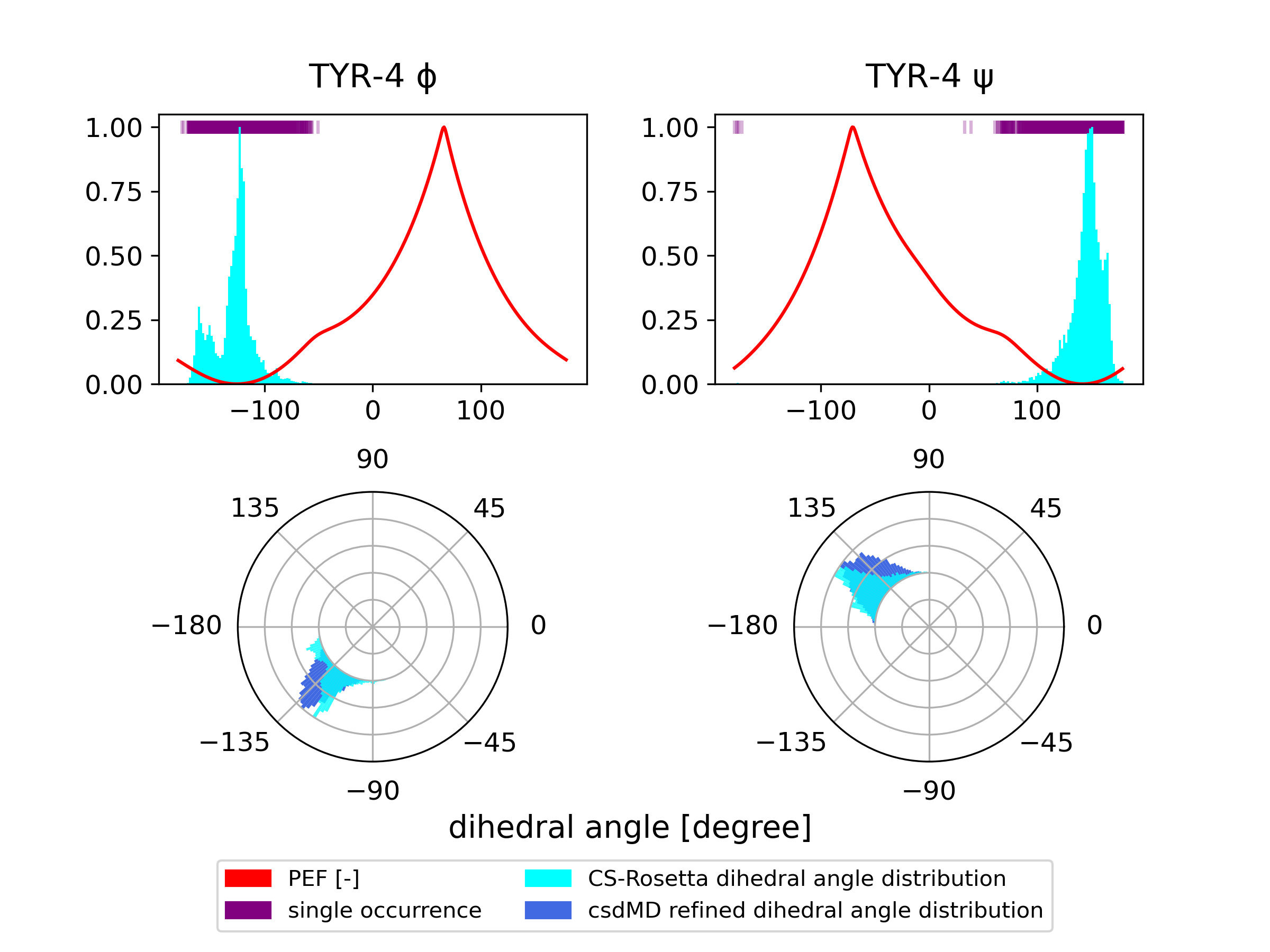

Supplement: Supplementary file 1 [file ijms-24-12101-s001.zip › KRAS-G12C-GDP-Mg-free_angle_figures/4-TYR.png]
