# Supplementary material for: NMR-Chemical-Shift-Driven Protocol Reveals the Cofactor-Bound, Complete Structure of Dynamic Intermediates of the Catalytic Cycle of Oncogenic KRAS G12C Protein and the Significance of the Mg2+ Ion
Source: Int J Mol Sci. 2023 Jul 28;24(15):12101. doi: 10.3390/ijms241512101 (PMC10418480; doi:10.3390/ijms241512101)
Supplement: Supplementary file 1 [file ijms-24-12101-s001.zip › Supplementary.pdf]

International Journal of *Molecular Sciences*

MDPI

NMR-Chemical-Shift-Driven Protocol Reveals the Cofactor-Bound, Complete Structure of Dynamic Intermediates of the Catalytic Cycle of Oncogenic KRAS G12C Protein and the Significance of the Mg^{2+} Ion

Márton Gadanecz [1,2], Zsolt Fazekas [1,2], Gyula Pálfy [1,3,4], Dóra Karancsiné Menyhárd [1,3] and András Perczel [1,3,*]

1 Laboratory of Structural Chemistry and Biology, Institute of Chemistry, Eötvös Loránd University, Pázmány Péter stny. 1/A, H-1117 Budapest, Hungary; marton.gadanecz@ttk.elte.hu (M.G.); dora.k.menyhard@ttk.elte.hu (D.K.M.)
2 Hevesy György PhD School of Chemistry, Eötvös Loránd University, Pázmány Péter stny. 1/A, H-1117 Budapest, Hungary
3 ELKH-ELTE Protein Modeling Research Group, Eötvös Loránd Research Network (ELKH), Pázmány Péter stny. 1/A, H-1117 Budapest, Hungary
4 Department of Biology, Institute of Biochemistry, ETH Zürich, 8093 Zürich, Switzerland
* Correspondence: perczel.andras@ttk.elte.hu

Supplementary Material

Table S1. All interacting atoms between the apo-protein, the GDP ligand and the Mg^{2+} cofactor are listed below, among which the atomic distances are between 2.1 and 3.0 Å in the reference crystal structure (4OBE).

Interacting atom group 1	Interacting atom group 2	Interacting atom group 1	Interacting atom group 2
Gly-13 N	GDP O3B	Pro-34 O	water-3 – Mg^{2+}
Lys-16 NZ	GDP O2B	Ile-36 O	water-2 – Mg^{2+}
Ser-17 OG	Mg^{2+}	Asp-57 OD1	water-4 – Mg^{2+}
Ala-18 N	GDP O1A	Thr-58 O	water-4 – Mg^{2+}
Phe-28 benzyl	GDP guanosine	Asp-119 OD1/OD2	GDP N1/N2
Asp-30 O	GDP O2	GDP O2A	water-1 – Mg^{2+}
Tyr-32 O	water-1 – Mg^{2+}	GDP O3B	water-3 – Mg^{2+}
Asp-33 O	water-2 – Mg^{2+}	Mg^{2+}	waters-1 to 4

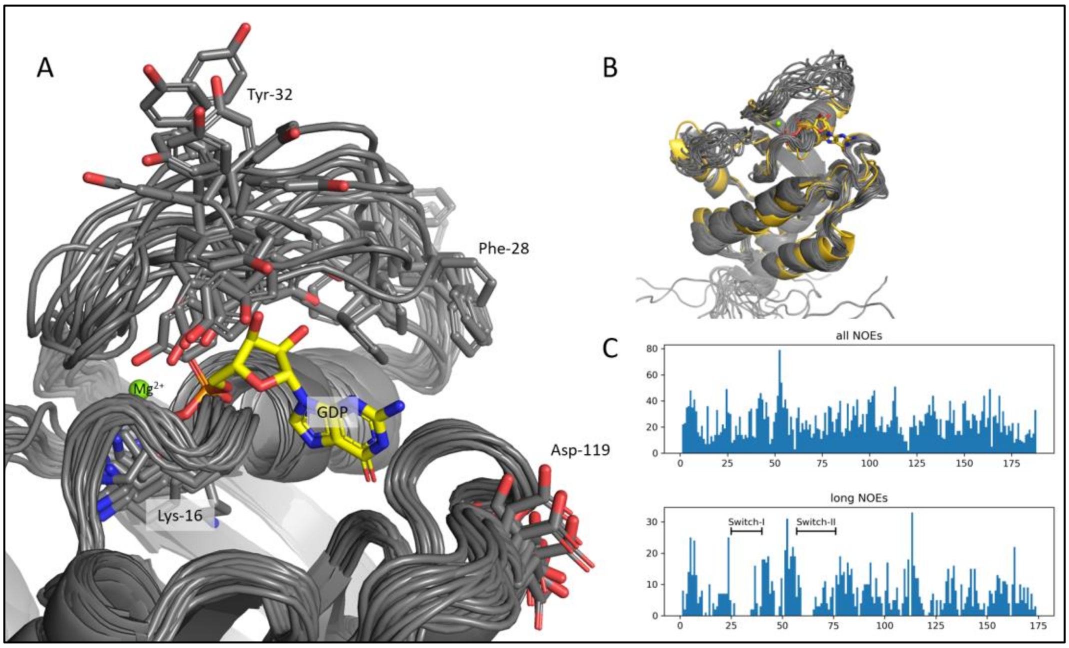

Figure S1. The 7KYZ NMR ensemble is shown in gray and the 4OBE crystal structure is shown in gold, as a reference. **A**) The nucleotide binding-pocket of the 7KYZ models, where the sidechains of some problematic amino acids of 7KYZ (gray) are represented as sticks and are labelled. The GDP and the Mg^{2+} ion are from 4OBE (gold), but they are not part of the NMR models. **B**) The superimposed full 3D structures of KRas-G12C. **C**) Distribution of NOE restraints depending on the residue position. The total number of distance restraints is 2278, from which 677 are derived from long NOE cross-peaks (where the given amino acids are more than three residues apart).

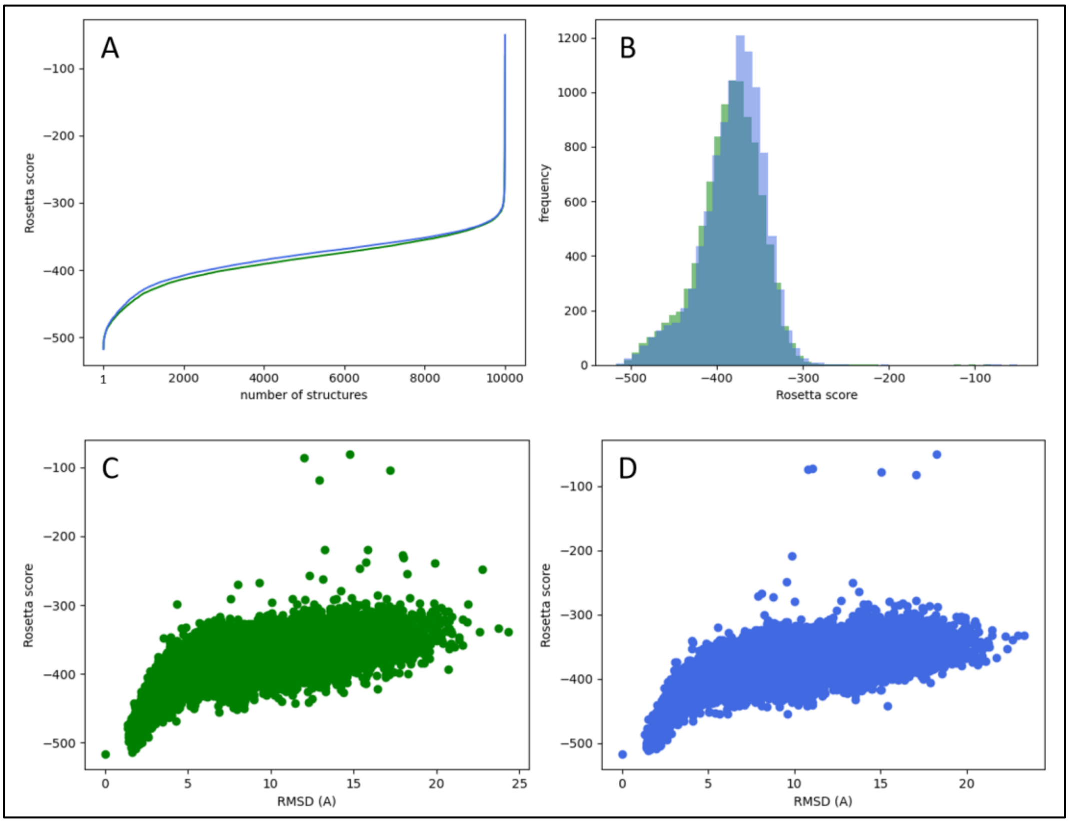

Figure S2. CS-Rosetta scores are shown for the KRAS-G12C/GDP-Mg^{2+} in green and the Mg^{2+}-free KRAS-G12C/GDP in blue. **A**) The Rosetta score distribution and **B**) the frequency of occurrence. **C** and **D**) The Rosetta scores of each model as a function of the heavy atom RMSD (in Å), compared to the best structure around the Mg^{2+}-bound and the Mg^{2+}-free CS-Rosetta ensembles, in green and blue, respectively.

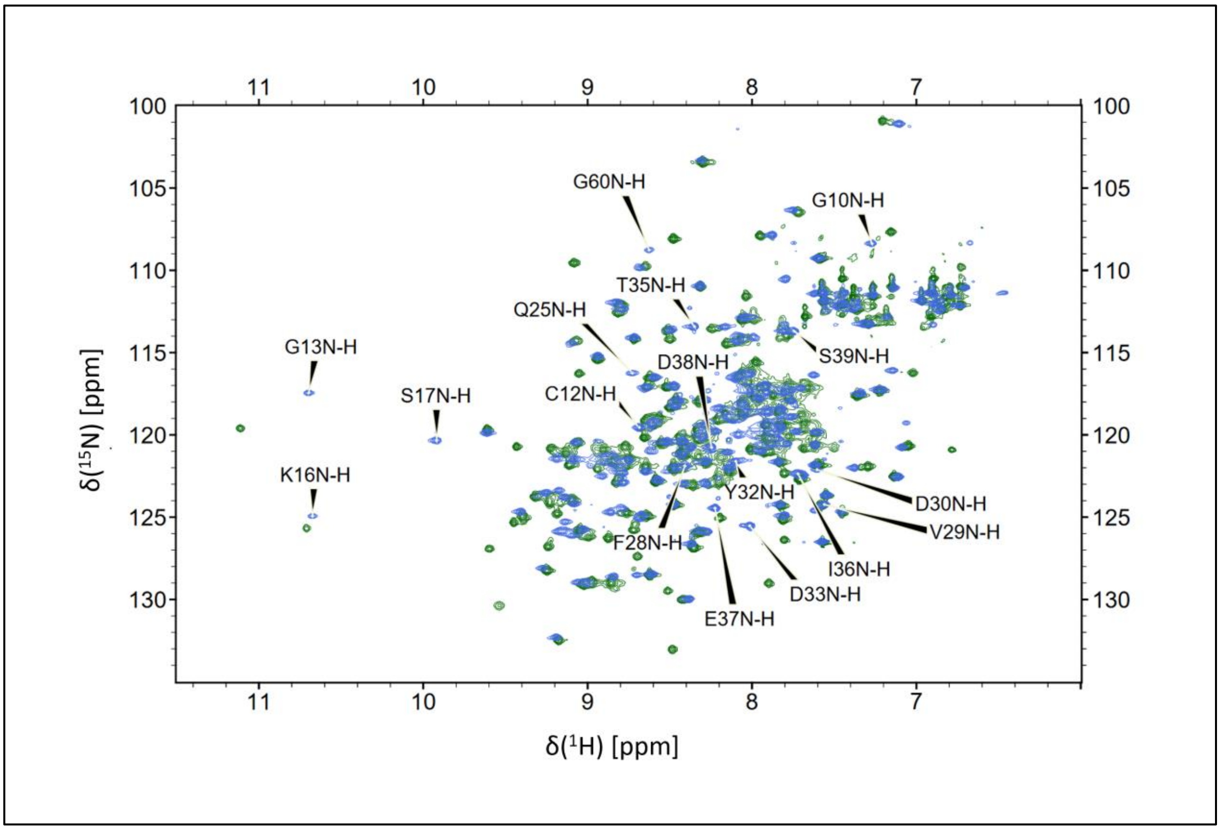

Figure S3. Overlaid 2D ^{1}H-^{15}N HSQC spectra of the KRAS-G12C/GDP-Mg^{2+} (green) and the Mg^{2+}-free KRAS-G12C/GDP (blue) systems. The assignment of some peaks with large chemical shift changes is shown (BMRB entry: 52009).

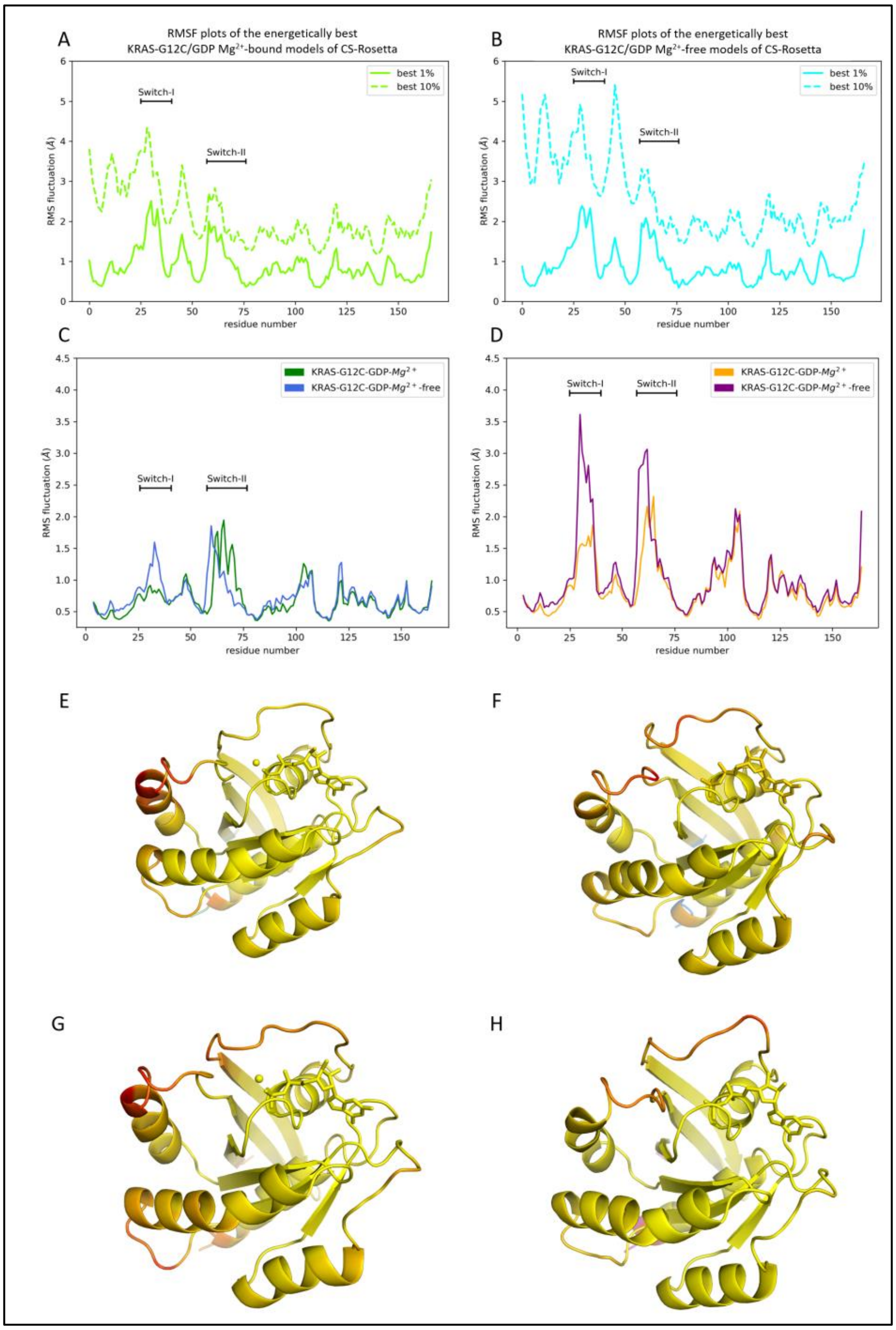

Figure S4. Root mean square fluctuations (RMSF) were calculated for the main chain and Cβ atoms of the P-loop, Switch-I and Switch-II regions (residues 10-48, plus 56-76). **A** and **B**) Root mean square fluctuation (RMSF) values of the best CS-Rosetta models. The best 0.1% (solid line), 5% (dotted line) and 10% (dashed line) are shown according to the CS-Rosetta score for the Mg^{2+}-bound (green) and Mg^{2+}-free (blue) ensembles. **C**) RMSF for csdMD simulations for the Mg^{2+}-free KRAS-G12C/GDP with our NMR-based modified force field. **D**) RMSF values of the KRAS-G12C/GDP-Mg^{2+} system obtained in the MD simulations using the original force field; **E**) KRAS-G12C/GDP-Mg^{2+} simulated with the NMR-based modified force field (csdMD). **F**) Mg^{2+}-free KRAS-G12C/GDP simulated with NMR-based modified force field (csdMD). **G**) KRAS-G12C/GDP-Mg^{2+} simulated with original force field (MD); **H**) Mg^{2+}-free KRAS-G12C/GDP simulated with original force field (MD).

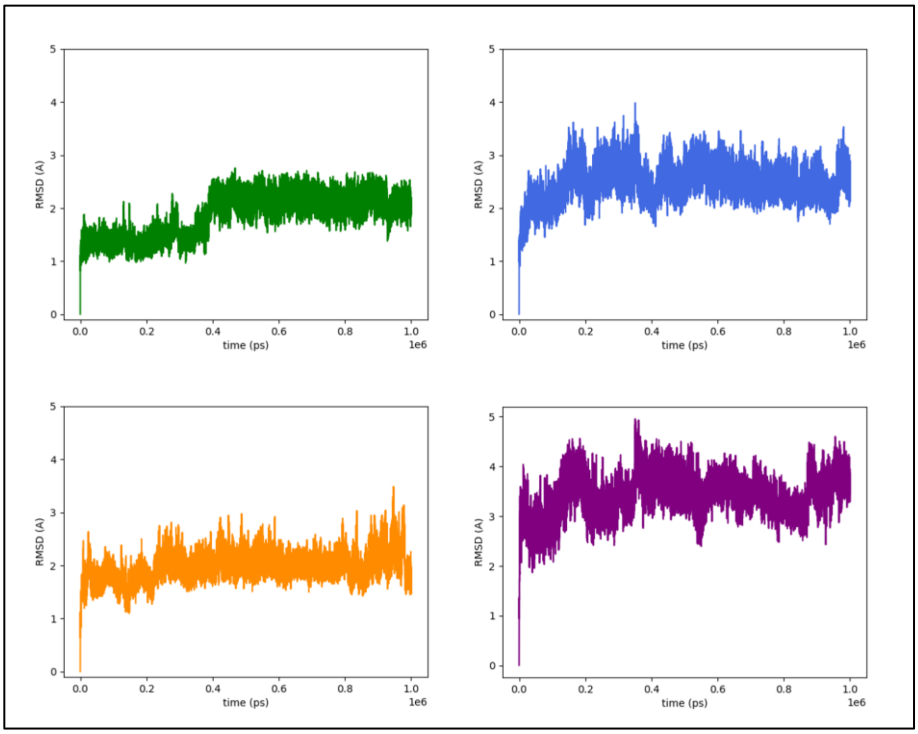

Figure S5. RMSD figures from MD simulations, starting from the best CS-Rosetta models, calculated for the main chain and Cβ atoms of the P-loop, Switch-I and Switch-II regions (residue 10-48, plus 56-76). KRAS-G12C/GDP-Mg^{2+} RMSDs are in green and orange, and Mg^{2+}-free KRAS-G12C/GDP RMSDs in blue and purple. The RMSDs for the simulations performed using our NMR-based modified force field are shown in green and blue, while the original unmodified force field was applied for the simulations whose RMSDs are colored orange and purple.

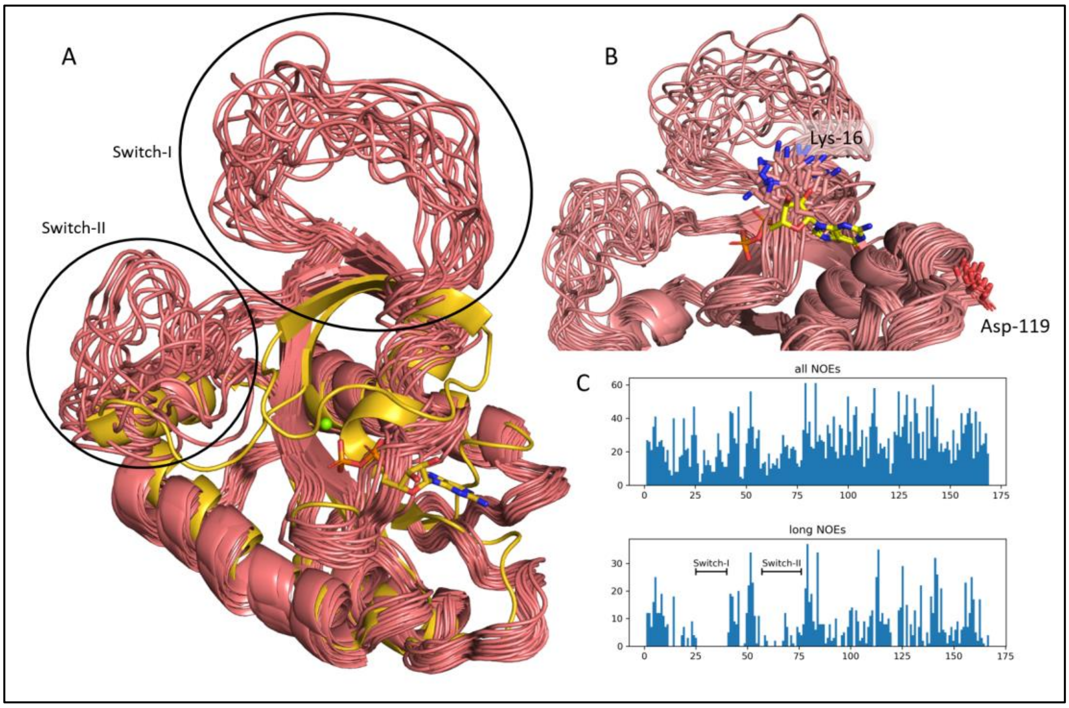

Figure S6. NOE-based structure determination with ARTINA. **A**) The best 20 structures are shown (as salmon cartoon) and the reference crystal structure (PDB ID: 4OBE, with gold color). **B**) The nucleotide binding-pocket is shown, where the sidechains of the Lys-16 and Asp-119 amino acids are shown as sticks and are labelled. The GDP ligand is shown as yellow sticks from the 4OBE crystal structure, but is not present in the ARTINA ensemble. **C)** Distribution of the NOEs depending on the residue number from the ARTINA calculations. The total number of distance restraints is 2200, and the number of long NOE cross-peaks (where the given amino acids are more than three residues apart) is 637.

Table S2. Comparison of quality metrics of the KRAS-G12C/GDP-Mg^{2+} structures determined with the csdMD method, the KRAS-G12C/GDP-Mg^{2+}-free structural ensembles of ARTINA and our csdMD refined models calculated with MolProbity.

* The median clashscores far all atoms are in the 99th percentile in all three cases. 100th percentile is the best among structures of comparable resolution; 0th percentile is the worst. For clashscore the comparative set of structures was selected in 2004, for MolProbity score in 2006.

^ MolProbity score combines the clashscore, rotamer, and Ramachandran evaluations into a single score, normalized to be on the same scale as X-ray resolution.

		**csdMD** KRAS-G12C/ GDP-Mg^{2+}			**csdMD** KRAS-G12C/ GDP-Mg^{2+}-free			**ARTINA** KRAS-G12C/ GDP-Mg^{2+}-free			
		average	median	SD	average	median	SD	average	median	SD	
All-Atom	Clashscore, all atoms:	1.92	1.83	0.80	1.75	1.65	0.69	1.15	1.12	0.40	*
Protein	Poor rotamers	4.1%	4.3%	1.4%	3.7%	3.6%	1.8%	15.5%	15.7%	2.5%	Goal: <0.3%
	Favored rotamers	87%	87%	3%	88%	88%	3%	65%	65%	3%	Goal: >98%
	Ramachandran outliers	0.64%	0.60%	0.57%	0.36%	0.00%	0.45%	0.81%	0.60%	0.68%	Goal: <0.05%
	Ramachandran favored	94%	94%	2%	95%	95%	2%	90%	89%	2%	Goal: >98%
	MolProbity score^	1.79	1.80	0.16	1.67	1.72	0.24	2.28	2.27	0.10	Goal: 0
